# Fluorescence Microscopy: a statistics-optics perspective

**Published:** 2023-05-05

**Authors:** Mohamadreza Fazel, Kristin S. Grussmayer, Boris Ferdman, Aleksandra Radenovic, Yoav Shechtman, Jörg Enderlein, Steve Pressé

**Affiliations:** 1 Department of Physics, Arizona State University, Tempe, Arizona, USA; 2 Center for Biological Physics, Arizona State University, Tempe, Arizona, USA; 3 Department of Bionanoscience, Faculty of Applied Science and Kavli Institute for Nanoscience, Delft University of Technology, Delft, Netherlands; 4 Russel Berrie Nanotechnology Institute and Department of Biomedical Engineering, Technion - Israel Institute of Technology, Haifa, Israel; 5 Laboratory of Nanoscale Biology, Institute of Bioengineering, Ecole Polytechnique Federale de Lausanne (EPFL), Lausanne, Switzerland; 6 III. Institute of Physics - Biophysics, Georg August University, Göttingen, Germany

## Abstract

Fundamental properties of light unavoidably impose features on images collected using fluorescence microscopes. Modeling these features is ever more important in quantitatively interpreting microscopy images collected at scales on par or smaller than light’s wavelength. Here we review the optics responsible for generating fluorescent images, fluorophore properties, microscopy modalities leveraging properties of both light and fluorophores, in addition to the necessarily probabilistic modeling tools imposed by the stochastic nature of light and measurement.

## I. INTRODUCTION

### A brief history of optics and statistics

A.

The ancient Greeks were divided over whether vision arose from rays entering or leaving the eyes [1, 2]. For instance, atomists believed that perception arose from a flux of atoms traveling through space to the eyes. Aristotle (384–322 BCE) later proposed the notion of ether serving as a medium for transmission of intrinsic qualities of objects to the eye rather than fluxes of atoms. An alternative formulation, advocated by Pythagoras (570–495 BCE) and Euclid (325–270 BCE), proposed the notion of ocular fire whose rays impassively scanned their surroundings. Following this logic, Euclid established a geometric optics to explain the perception of size and angle based on the geometry of these ocular rays. Along these same lines, the Chinese philosopher Mo Di (470–391 BCE) had established a geometrical optics very similar to Euclid’s to explain the formation of shadows and images in mirrors [3].

An amalgam of these ideas–where the fire originating from the eyes coalesced with sunlight coming into contact with another fire derived from objects to enable vision–was perhaps now demanded on philosophical grounds and promoted by Plato (427–347 BCE). In Ptolemy’s optics (100–170 CE), sunlight was a means to activate objects to emit rays which interacted with visual rays to give rise to perception. In this theory, Ptolemy explains perception based on the angular distribution, length, refraction and reflection of rays from the eye [1, 4].

Although these early Greek theories appear manifestly naive, notions of geometric optics served as a clear starting point to Medieval Arabs who took a decidedly more phenomenological approach. For example, inspired by Euclid’s geometric optics, Al-Kindi (801–873 CE) demonstrated that visual rays travel in straight lines by simple experiments on shadows [1]. This early progress was followed by insights from Ibn al-Heytham–latinized as Alhazen (965–1040 CE)–who reasoned that eyesight is derived from light rays directed toward the eyes from objects [1, 5]. He further developed experiments to study both refractive and reflective properties of light rays on boundaries, lenses and spherical mirrors [1, 5].

The distribution of Latin translations of Alhazen’s *Book of Optics* amongst other ancient works, ultimately sparked a Renaissance that presages the onset of modern optics in Europe. From the democratization of knowledge driven by the indefatigable Gutenberg presses followed refractive telescopes attributed to the Dutch spectaclemakers Zacharias Janssen (1585–1638 CE) and Hans Lippershey (1570–1619 CE) and reflecting telescopes attributed to Issac Newton (1643–1727 CE) [6]. In contrary to telescopes, there is uncertainty regarding the original inventor of microscope, it is however often credited to Zacharias Janssen [7].

From the very start, the world of microscopy and biology were intertwined: the Dutch businessman and scientist Antonie van Leeuwenhoek (1632–1732 CE) exploited his microscope to single-handedly discover bacteria, sperm cells, and red blood cells amongst other actors dominating the microscopic realm [7]. Little, in this regard, has changed throughout history with sizes, features, and other optical properties of the Natural world motivating the design of modern microscopes. In this regard, *compound* microscopes [8], also credited to Janssen and foreshadowing our multi-lens microscopes, provided improved magnification and were widely used by Robert Hooke (1635–1703 CE) [8], author of the first book on microscopes *Micrographia*.

Now taken for granted, successive properties of light–including diffraction, refraction, reflection as well as light’s particulate nature–were each individually leveraged in microscope development with diffraction through an aperture first reported by the Italian Jesuit Francesco Maria Grimaldi (1618–1663 CE), followed by a number of discoveries culminating in Maxwell’s (1831–1879 CE) electromagnetic theory, and theories on light’s quantization [9, 10] due to Planck (1858–1947 CE) and Einstein (1879–1955 CE).

Throwing the remarkable later progress in microscopy development–including phase imaging–to the wind for sake of expediency, we interrupt history to pause at fluorescence microscopy which has dominated the scene of the last half century as smaller scales, demanding increased contrast between background and object of interest, have drawn our attention [11]. At such scales, the stochastic properties of light, intrinsic to quantum mechanics, dictate our ability to interpret fluorescence microscopy data and brings us back to the primary focus of this review: fluorescence microscopy from a statisticsoptics perspective.

Modeling the stochastic properties of light isn’t an exercise in mitigating the recurring nuisance of shot noise. It is, instead, fundamental to how we draw insights at the scales fluorescence microscopy has made available. In fact, a fluorescent photon’s emission time, its absorption time, emission wavelength, and detection location, *i.e.*, where a photon is detected on an image plane, are all random variables. These random variables themselves are drawn from probability distributions. In the classical limit, the probability density for locating photons is proportional to the time-averaged energy flux given by Poynting’s theorem [12], introduced by John Henry Poynting (1852–1914 CE). For point-like sources of light, *e.g.*, fluorophores, the normalized spatial distribution, coinciding with a slice orthogonal to the propagation direction, is termed the Point Spread Function (PSF). This inherent randomness in a photon’s location, reporting only probabilistically on a fluorescent object of interest, and detected imperfectly now introduces multiple levels of stochasticity between the object whose properties we care to characterize and measurement output. This, unavoidably, introduces statistical concepts–including latent variables and hierarchical probabilistic models–in the quantitative modeling of imaging systems.

The manipulation of hierarchical dependencies between random variables then requires what is known today as Bayes’ theorem. The theorem is attributed to its namesake Thomas Bayes (1702–1761 CE) and was popularized by Pierre-Simon de Laplace (1749–1827 CE) who introduced and codified, through seminal texts on probability [13, 14], probabilistic modeling to the Sciences [15].

Before we return to microscopy, we now take a brief detour to discuss statistical modeling relevant to our future applications.

### B. Introduction to statistical modeling

The electromagnetic force carrying particle, the photon, is intrinsically both wave-like and particulate. While the continuous spatial distributions over a photon’s potential location, *e.g.*, its PSF determined by the physics of diffraction, are dictated by the photon’s wave properties, photon detections themselves are necessarily pointillistic and probabilistic.

As such, even before considering other sources of stochasticity like detection, a quantitative picture of microscopy demands, at its most fundamental level, an exposition of the theory of statistical sampling.

Here, we first lay out the main concepts for probabilistic modeling. We then discuss the concept of likelihoods and Bayesian inference key to the statistical frameworks introduced throughout this review.

#### 1. Basic concepts and notation

Stochasticity in a system arises either from the inherent random nature of the physical system or measurement noise or both. Both are relevant to modeling in quantitative microscopy and thus we minimally require two layers of stochasticity: at the level of Poisson shot noise of photon and at the level of detection; see Appendix A. Soon, we will also see that stochasticity can also arise from the behavior of fluorescent labels as well.

For this reason, we begin by defining the requisite notions of a random variable. A random variable, R, represents a collection of possible options, either numeric or non-numeric, following the statistics of a probability distribution P.

As such, we often write

(1) R∼P

where the above reads “the random variable R is sampled from the probability distribution P”. We then denote r a particular realization of R and p(r) the probability density associated with the probability distribution P.

Generally, the probability distribution itself depends on parameters, ϑ. To make such dependency explicit, we may write p(r;ϑ) and P(ϑ).

For example, the location at which the photon is detected is itself a random variable, R, sampled from a distribution centered at the emitting molecule’s location, $r_{0}$. As such, we write

(2) $$R∼U\left(r_{0}\right),$$

(3) $$p\left(r;r_{0}\right)=U\left(r;r_{0}\right),$$

where $\vartheta \equiv r_{0}$, and p(r;ϑ) is the probability density, *i.e*., the PSF, from which r is drawn.

It is sometimes of interest to compute the probability of obtaining a value from a subset η of the possible values (r∈η), given by

(4) $${𝒫}_{\eta }=\int_{\eta }\;drP\left(r;\vartheta \right).$$

By definition if η is the entire set of options then ${𝒫}_{\eta }=1$. For instance, the probability of a photon reaching a pixel is given by the integral of the PSF over the pixel area 𝒜

(5) $${𝒫}_{\text{pix}\;}=\int_{𝒜}\;U\left(r;r_{0}\right)dr.$$

In probabilistic modeling, we often work with many random variables, $R_{1},R_{2},\ldots ,R_{N}$, at once. For this reason, we often write the joint density

(6) $$p\left(r_{1:N};\vartheta \right)=p\left(r_{1},r_{2},\ldots ,r_{N};\vartheta \right).$$

The density of any individual $r_{n}$ is then obtained by integrating the joint density with respect to $r_{1:n-1}$ and $r_{n+1:N}$

(7) $$p\left(r_{n};\vartheta \right)=\int \;dr_{1:n-1}dr_{n+1:N}p\left(r_{1:N};\vartheta \right),$$

where we integrate over all possible values. This integration, termed a marginalization, results in a marginal density, $p\left(r_{n};\vartheta \right)$. Marginalization is often useful as we wish to compute, say, the probability of an emitter (a fluorescently labeled molecule or dye) diffusion coefficient irrespective (and thus integrating over) its exact location. This is later explored in, *e.g.*, Fig. 44.

If random variables $R_{1:N}$ are independent and identically distributed *iid*, then Eq. 6 assumes the simpler form

(8) $$R_{1},R_{2},\ldots ,R_{N}\overset{iid}{∼}P(\vartheta )$$

with the understanding that the joint density decomposes into the product of independent densities $p\left(r_{1};\vartheta \right),p\left(r_{2};\vartheta \right),\ldots ,p\left(r_{N};\vartheta \right)$.

For example, *iid* random variables include photon arrival times following pulsed excitation for a static distribution of molecules or mixtures of molecules with no change in lifetime over time; *e.g.*, as later explore in the box IV C.

In general random variables are not independent, *e.g.*, the position of a molecule in time. For example, the state of a system may either depend on its state at a previous time point either exactly or by approximation. This dependency, explored in the context of fluorophore dynamics in Sec. II C is termed the Markov assumption.

In this case, we say that values that can be ascribed to $R_{2}$ depend on the realization of a preceding random variable $r_{1}$. This dependency is often expressed as

(9) $$R_{2}|r_{1},\vartheta ∼P\left(r_{1},\vartheta \right),$$

which reads “the random variable $R_{2}$ given the parameters ϑ and realization (or “conditioned on”) $r_{1}$ of $R_{1}$ is sampled from the probability distribution $P\left(r_{1},\vartheta \right)$. The density we associate to this probability distribution then reads $p\left(r_{2}|r_{1},\vartheta \right)$ and is referred to as a conditional density.

As is customary in physics, we will now denote both random variables and their realizations with lower case letters. The distinction between both notions will be implied by the context.

In the most general setting, a random variable $R_{N}$ can depend on many other random variables $R_{1:N-1}$ with associated conditional density $p\left(r_{N}|r_{1:N-1},\vartheta \right)$. Such conditionals will become useful as we build hierarchical models relating random variables across all of our boxed environments.

Finally, we end with a note in constructing conditional densities such as

(10) $$p\left(r_{1:2}\right)=p\left(r_{2}|r_{1}\right)p\left(r_{1}\right)$$

where $r_{1}$ or $r_{2}$ may, in full generality, coincide with sets of random variables. The expression above can then be used to derive Bayes’ formula, also termed Bayes’ theorem, of central importance in this review from the observation that $p\left(r_{1:2}\right)=p\left(r_{2:1}\right)$ and thus

(11) $$p\left(r_{1}|r_{2}\right)p\left(r_{2}\right)=p\left(r_{2}|r_{1}\right)p\left(r_{1}\right).$$

#### 2. Likelihood

We are now in a position to introduce the object at the heart of quantitative analysis of microscopy data: the likelihood. The likelihood is a probability distribution over those random variables coinciding with K experimental observations, $w_{1:K}$, conditioned on $w_{1:K}$. The likelihood’s density is therefore written as $p\left(w_{1:K}|\vartheta \right)$ where $w_{1:K}=\left\{w_{1},w_{2},\ldots ,w_{K}\right\}$. It is also convenient to denote this set using the overbar, $\overset{‾}{w}$.

The term likelihood follows from the notion that $p\left(w_{1:K}|\vartheta \right)$ is a likelihood of observing the sequence of observations $w_{1:K}$ under the assumptions of the model (*i.e.*, calibrated values for parameters ϑ of a particular model). Indeed, all box environments will contain likelihoods for each statistical framework presented.

Often as the parameters are themselves unknown, we ask what values for these parameters make the likelihood of the observed sequence $w_{1:N}$ the greatest. The parameter values maximizing the likelihood are called estimators and are denoted by $\overset{ˆ}{\vartheta }$. For example, we can ask what values of the excited lifetime (assuming one lifetime component) make an observation sequence largest; *e.g.*, box IV C.

For practical reasons, it is common to work with, and maximize, the likelihood’s logarithm $ℒ\left(w_{1:K}|\vartheta \right)=log\left(p\left(w_{1:K}|\vartheta \right)\right)$, sometimes termed log-likelihood, rather than the likelihood itself, *e.g.*, see Sec. VC. This is because the logarithm is monotonic with the original function and the logarithm avoids numerical underflow typical of small probability densities arising when K grows.

Within a Maximum Likelihood Estimation (MLE) framework, ϑ are treated as fixed (deterministic) parameters and the data, $w_{1:K}$, are understood as realized random variables.

While the MLE yields a single value (estimator) for the parameters of interest, it may be possible to capture the uncertainty around the parameter estimate by computing the likelihood’s breadth around its maximum. The breadth is often estimated as

(12) $$\sigma_{\vartheta_{l}}^{2}={\left[𝒬(\vartheta {)}^{-1}\right]}_{ll},$$

where l counts the elements of the model parameter set, ϑ. Here, 𝒬(ϑ) is the Fisher information matrix defined as [16, 17]

(13) $${𝒬}_{ll^{\prime }}(\vartheta )=E\left[{\left.\frac{\partial^{2}ℒ\left(w_{1:K}|\vartheta \right)}{\partial \vartheta_{l}\vartheta_{l^{\prime }}}\right|}_{\overset{ˆ}{\vartheta }}\right]$$

where E denotes expected value of the expression inside the parenthesis. As the breadth of Eq. 12 sets a minimum bound on the variance to which a MLE can be pinpointed it is sometimes termed the Cramer Rao Lower Bound (CRLB).

As may be evident, MLE-based approaches fail when the likelihood has multiple degenerate maxima or, more importantly, when the model (let alone the model parameters) are unknown. What is more, even assuming a model form, the likelihood only provides a point estimate not a full distribution over the putative parameter values.

It is for all these reasons that we now turn to a more general Bayesian paradigm.

In this more general setting, we will use the likelihood as described above to construct the distribution over the parameters of interest given the observed data, $p\left(\vartheta |w_{1:K}\right)$. The latter object is termed the posterior and is central to Bayesian inference.

#### 3. Posterior

In working with likelihoods, the data is understood as random variables and parameters, ϑ, as fixed but to be determined. By contrast, in a Bayesian setting both data and parameters are treated as random variables. In particular, the data are random variables already realized and whose values are used to construct the probability, $p\left(\vartheta |w_{1:K}\right)$, over the unknown random variables, ϑ. This Bayesian paradigm allows us to properly propagate uncertainty from all sources that arise from the particular application, such as, detector noise, camera intensity pixelation, motion aliasing, and photon shot noise.

The posterior is constructed from the likelihood by invoking Bayes’ formula, Eq. 11,

(14) $$p\left(\vartheta |w_{1:K}\right)=\frac{p\left(w_{1:K}|\vartheta \right)p(\vartheta )}{p\left(w_{1:K}\right)},$$

where the normalization reads

(15) $$p\left(w_{1:K}\right)=\int \;d\vartheta p\left(w_{1:K}|\vartheta \right)p\left(\vartheta \right).$$

Here, p(ϑ) quantifies any prior knowledge about the system parameters (prior to the data). In practice, it is a largely “flat” distribution between the upper and lower bounds permitted on values of the parameters.

In summary, Bayes’ formula provides a clear recipe by which to update our prior distribution using the set of observations, $w_{1:K}$, encoded in the likelihood, to arrive at the posterior $p\left(\vartheta |w_{1:K}\right)$. As we will see in all applications, likelihoods can generally be constructed from knowledge of the microscopy technique and the physics of the problem while priors are normally imposed as largely flat distributions. The broad question then arises: Can we determine whether the posterior is peaked at some value of ϑ More concretely, what does our posterior look like?

Unfortunately, posteriors rarely attain a simple, analytic form, on account of the measurement effects and optical physics informing the likelihood. As such, values of ϑ are typically numerically sampled from posteriors using Monte Carlo methods. For example, as we later discuss in the context of confocal microscopy, *e.g.*, Sec. IV C, we will see that ϑ includes quantities such as diffusion coefficients, emission rates, and emitter locations. As posteriors are thus often multi-variate, a common textbook Monte Carlo strategy, loosely, involves sampling one random variable at a time in a scheme termed Gibbs sampling [18].

Whether sampling a posterior exactly or numerically, via Gibbs sampling, it is often computationally convenient to judiciously select the functional form of priors over parameters. Indeed, some prior forms play a special role in Bayesian modeling by having the unique mathematical property that, when multiplied by the likelihood, result in a posterior of the same form as the original prior (albeit with updated parameters). As such, we often speak of conjugate prior-likelihood pairs or, for succinctness, conjugate priors when such priors can be identified. While we will not dwell on specialized notions of Bayesian inference, we make the reader aware that computational efficiency is what makes it possible to include measurement noise details at marginal added computational cost whilst improving the spatiotemporal resolution of any fluorescence analysis method. Indeed, whenever possible, specialized Monte Carlo schemes (from Gibbs sampling [18], to Metropolis-Hastings [19, 20], to slice sampling [21], and beyond [22, 23]) used across all applications discussed herein benefit from any computational advantage thrown their way.

#### 4. Bayesian non-parametrics

From Eq. 14, we see that constructing a posterior demands a mathematical, *i.e.* “parametric”, form of the likelihood. However, for most practical cases, we often do not know what competing models describe a given data set. We also know, and can demonstrate by way of examples, that the more complicated we make a model, the larger its likelihood, *i.e.*, we over-fit the data.

Compromising between data under- and over-fitting is at the heart of the fundamental model selection problem. From the onset, progress in model selection has been critical, for instance, in clustering problems where the number of clusters (*i.e.*, the model) are unknown [24–27]. Indeed, the model selection problem manifests itself across microscopy applications. For example: determining the number of molecules within a diffraction-limited spot (*i.e.*, the model) explored in boxs II C; or determining the number of lifetime components in lifetime imaging explored in box IV C.

While heuristically comparing a fixed set of models to resolve model selection—for example by relying on information criteria [28], and other tools introduced as post-processing steps—is computationally advantageous, such an approach presents theoretical problems.

For example, it is often only in limited cases where we can successfully exhaustively enumerate models. For example, how many emitters in each frame across a stack of frames can we consider in any wide-field tracking application? Even if we can enumerate them, how do we assign probabilities to these competing models given the data?

Answers to these questions, outside the realm of the Natural Sciences, led to the formal development of Bayesian Non-Parametrics (BNPs) [29] alongside Monte Carlo tools to sample from the resulting non-parametric posteriors, including Reversible Jump Markov Chain Monte Carlo (RJMCMC) [30].

In short, BNPs treat model and parameter estimation on the same footing [27, 31, 32] and construct non-parametric posteriors over both models and their associated parameters.

In particular, within a non-parametric treatment, we consider *a priori* an infinite number of competing models. We place priors on these models alongside their associated parameters just as we place priors on parameters alone within the regular (parametric) Bayesian paradigm.

One catch is that BNPs are limited to a particular class of models termed nested models. Fortuitously, many models we consider across microscopy applications belong to this class. Briefly, nested models include all models that can be generated from a more general model by setting parameters to different values (including zero) with the most general model itself being infinite dimensional. For example, a two state model used in analyzing a Förster Resonance Energy Transfer (FRET) time trace, later explored in greater depth in Sec. II A, follows from a three state model where transitions to the third state are all set to zero. Other examples of nested models we will explore include: 1) the number of molecules in a diffraction-limited spot (boxes II C and V B 3); 2) the number of lifetime components in lifetime imaging (box IV C); and perhaps less intuitively 3) the spatial 2D lifetime map, of all competing two-dimensional lifetime maps, obtained from scanning confocal lifetime imaging (box IV C).

These examples were intentionally enumerated. They allow us to introduce three commonly used non-parametric priors used in constructing non-parametric posteriors. In the order in which these examples are listed, we have: the beta-Bernoulli process prior [33–37]; the Dirichlet process prior [25–27]; and the Gaussian Process (GP) prior [38, 39].

The beta-Bernoulli process prior is used when we try to estimate the number of discrete elements contributing to the data. These could be, for example, the number of emitters in an image frame or a confocal spot, *e.g.*, box IV C. Within a BNP paradigm, we assign a Bernoulli variable (binary random variable), $b_{m}$ called a load, to each discrete element (molecule). Considering as many as M loads (and letting M eventually tend to infinity), the unknowns appearing in ϑ are augmented to include $b_{1:M}$. Thus ϑ for the single spot confocal would now include the diffusion coefficient, emission rate, molecular locations, as well as loads, $b_{1:M}$.

When multiplying the likelihood by the appropriate beta-Bernoulli prior process, we may then construct a posterior whose parameters we wish to sample include the loads. The resulting posterior is, in turn, often sampled using Monte Carlo techniques to determine which loads are sampled mostly as 0’s (and thus coincide with molecules not warranted by the data) or coincide with 1’s (and thus coincide with molecules warranted by the data). The number of molecules from each Monte Carlo draw from the posterior are then determined by summing all loads.

We now turn, much more briefly, to the subsequent two non-parametric priors. For instance, the Dirichlet process prior is used when we wish to assign probabilities to an infinite number of components. For example, when we wish to determine to what degree each unique chemical species contributes photons in a lifetime experiment (box IV C). Ideally, based on Monte Carlo sampling of the non-parametric posterior (obtained from the product of the likelihood and the Dirichlet process prior), we would find which of the infinite species introduced in modeling contribute non-negligibly to the data.

Finally, GP priors are used in estimating smooth functions. Smooth functions of interest in microscopy include, for example, fluorophore density maps explored in Sec. IV C or even smooth background when the number of emitters are large and discretely localizing each is infeasible.

Each of these maps consists of an infinite set of correlated random variables, *i.e.*, values of the map at every point in space. Draws from the posterior then assign values to each point on the map. In practice, the number of map points whose value we wish to deduce is kept finite and limited to a fixed number of points typically over a uniform mesh grid termed inducing points [39]. The value of map on a finer spatial grid can then be obtained by interpolation given the spatial correlation function already informing the GP prior.

Having now introduced key notions from statistics, we turn to microscopy.

### C. Basic characteristics of fluorescence microscopy

All microscopes use light, one way or another, to interact with the sample under observation. Indeed, bright-field, dark-field, or even phase contrast imaging differ from each other in details pertaining to which part of the excitation or detection arms are altered or blocked to create images at the microscope objective.

**FIG. 1:**
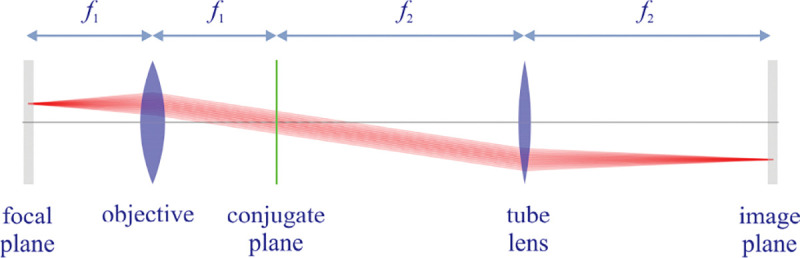
Schematic of an infinity-corrected wide-field microscope consisting of an ideal objective lens with focal length $f_{1}$ and an ideal tube lens with focal length $f_{2}$. We show the light propagation from a point source in the focal plane (sample space) to the image point in image space. The plane between the lenses a distance $f_{1}$ away from the objective lens and $f_{2}$ from the tube lens is called the conjugate plane (green vertical line). Here the light from any point source on the focal plane crosses through the same lateral position. By considerations of geometric proportion, it can be seen that the ratio of lateral displacement of the image point to lateral displacement of the source point is equal to the ratio of the focal lengths, $f_{2}/f_{1}$. This ratio is the microscope’s magnification ℳ.

However, these microscopes are limited in their ability to discern contrast at the molecular and even supramolecular length scales at which life unravels. At such scales, we exploit fluorescence microscopy in which a sample contains fluorophores later detailed in Sec. II. When excited, fluorophores emit light that can be selectively filtered from the excitation beam and used to form an image. In its simplest form, a fluorescence microscope is a two-lens system, consisting of an objective with small focal length $f_{1}$ and a tube lens with long focal length $f_{2}$; see Fig. 1.

In modern research microscopes, the objective converts the diverging spherical wavefront emitted by a point emitter in the focal plane in sample space into a planar wavefront. The planar wavefront is then reconverted by the tube lens into a spherical wavefront converging into a point on the image plane in an infinity-corrected microscope.

The two most important characteristics of a microscope are its magnification and its resolution, *i.e.*, how well sample features are resolved.

From Fig. 1, the system’s magnification is understood as given by the ratio $f_{2}/f_{1}$ (from the proportionality of vertical to horizontal distances). However, the magnification of an optical microscope today is of secondary importance as images are recorded with array detectors, such as Complementary Metal-Oxide Semiconductor (CMOS) or Electron Multiplying Charge-Coupled Device (EMCCD) cameras with varying pixel size; see Sec. A. This is by contrast to visually inspecting a sample where our eyes’ light sensitive receptor sizes, namely rod and cone cells, are fixed. For such wide-field microscopes equipped with a camera, the detector’s physical pixel size divided by the microscope’s magnification set an upper bound on the image quality. This effective pixel size should be at least two times smaller than the microscope’s optical resolution (Nyquist criterion).

This leads us to the second important microscope characteristic: its resolution. The microscope resolution is limited by the diffraction of light and light collection by objective lenses among multiple other factors. These two effects lead to a fundamental resolution limit of approximately half of the wavelength. As such, if the emitted light’s wavelength were to be far smaller than typical dimensions of the molecular species of interest, then our review article would stop here and textbooks would be replenished with real life images reminiscent of David Goodsell’s artistic renderings of life inside the cell [40]. However, this is not the case.

**FIG. 2:**
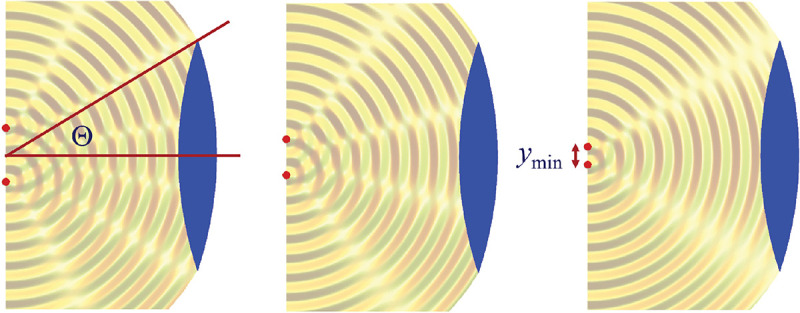
Visualization of the diffraction limit of resolution. Here, we show interference patterns of two coherently emitting point emitters, shown by red dots, for three different distances between emitters across panels. The closer the emitters are positioned with respect to each other, the larger the angular positions of the destructive interference lanes (directions of zero light intensity). At a critical distance, shown in the right panel, the first lane of destructive interference is positioned at the half angle Θ of light collection of the objective, and the objective lens receives a continuous wavefront absent intensity minima appearing as a single emitter wavefront.

We will discuss more thoroughly resolution of different microscope modalities shortly though we start with a heuristic albeit useful visualization of a fundamental microscope’s optical resolution limit; see Fig. 2. Here we show the (far-field) light intensity distribution of two coherent point sources, designated by red dots, before an objective lens. As both point sources are assumed to emit light coherently, the resulting intensity distribution shows characteristic lanes of constructive and destructive interference. When the distance between the two point emitters, y, is gradually reduced (from left to right panels in Fig. 2), the two symmetric lanes of destructive interference (directions of zero light intensity) closest to the optical axis migrate towards higher emission angles, until they reach the objective lens’ edge. At that point, the objective detects only light of a continuous spherical wavefront absent any zero-intensity minima within its light detection cone (with half angle Θ), similar to what the objective would see from a single point emitter.

Simple trigonometry dictates that the path difference between 1) the first emitter, and the edge of the lens, and 2) the second emitter, and the same edge of the lens is y sin Θ. In doing so, we assumed that the separation of the lens, and emitters is much larger than y in the far-field limit. The first destructive interference lane therefore occurs at angle Θ if the path difference is half the wavelength, *i.e.*, $y_{min}sin\;\Theta =\lambda /2n$, where λ is the vacuum emission wavelength, and n is the sample’s refractive index. As such, the wavelength in the sample is λ/n. From this result follows Abbe’s famous resolution limit, first formulated by Ernst Abbe (1840–1905 CE) in 1873 [41], as

(16) $$y_{min}=\frac{\lambda }{2n\;sin\;\Theta }=\frac{\lambda }{2NA},$$

where NA is the objective’s numerical aperture.

**FIG. 3:**
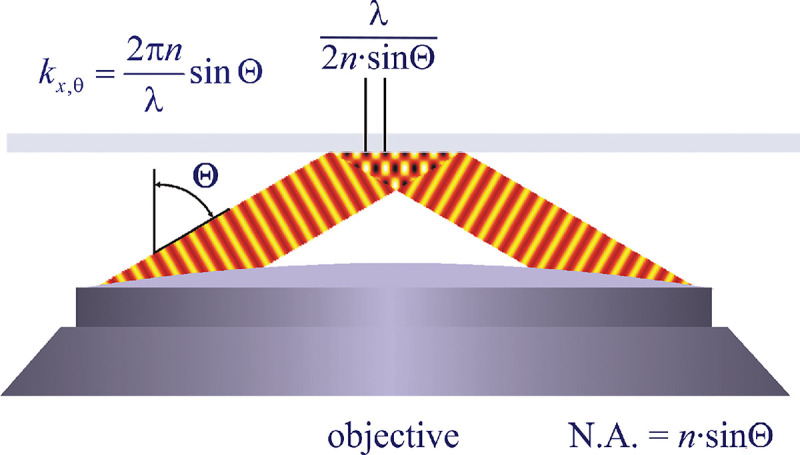
Lateral resolution limit of a CLSM. The resolution is determined by the highest lateral spatial frequency contained in a focused bright spot. This is generated by the interference of two rays traveling from the edges of the objective to the focal point with the highest possible incidence angle Θ with respect to the optical axis as shown. The associated wave vectors are of equal magnitude, 2πn/λ, where λ is the vacuum wavelength. The corresponding lateral components, $k_{x,\theta }$, of these wave vectors are of equal magnitude given by $k_{x,\theta }=2\pi n\;sin\Theta /\lambda$, and opposite directions resulting in a difference of 4πnsinΘ/λ. As such, the interference of the two beams leads to a periodic interference pattern in the lateral direction with periodicity λ/2nsinΘ, equal to the lateral resolution limit of a CLSM.

A similar simplified consideration can also be applied toward understanding the spatial resolution of a Confocal Laser Scanning Microscope (CLSM). In a CLSM, the sample is scanned with a tightly focused laser beam, and the excited fluorescence light is collected by the microscope optics, focused through a confocal pinhole to suppress out-of-focus light, and finally detected with a point detector (usually silicon-based photo-diode, or photo-electron multiplier tube); see Sec. IV B 1. The recorded fluorescence light intensity as a function of scan position is then used to reconstruct an image. The fundamental advantage of a CLSM as compared to a wide-field imaging microscope is its optical sectioning capability, *i.e.*, its capability to record true three-dimensional sample images, later detailed when considering the Optical Transfer Functions (OTF) of both microscope types. Neglecting momentarily a CLSM’s confocal detection volume, then its lateral resolution is determined by how tightly a laser beam can be focused into an excitation spot. In a mathematically more precise manner, one asks what the tightest spatial intensity modulation is that is still present in a diffraction-limited focus. The answer is given by Fig. 3, which shows that the tightest modulation is achieved by the interference of the two light rays exiting the objective at the highest possible angle, which is exactly the half angle of light detection Θ of the objective. As can be seen, the spatial periodicity of this intensity modulation is again given by Abbe’s formula, Eq. 16, only with the emission wavelength now replaced by the excitation wavelength (usually shorter than the emission wavelength due to the spectral Stokes shift of fluorescence emission with respect to excitation; see Sec. II for details).

**FIG. 4:**
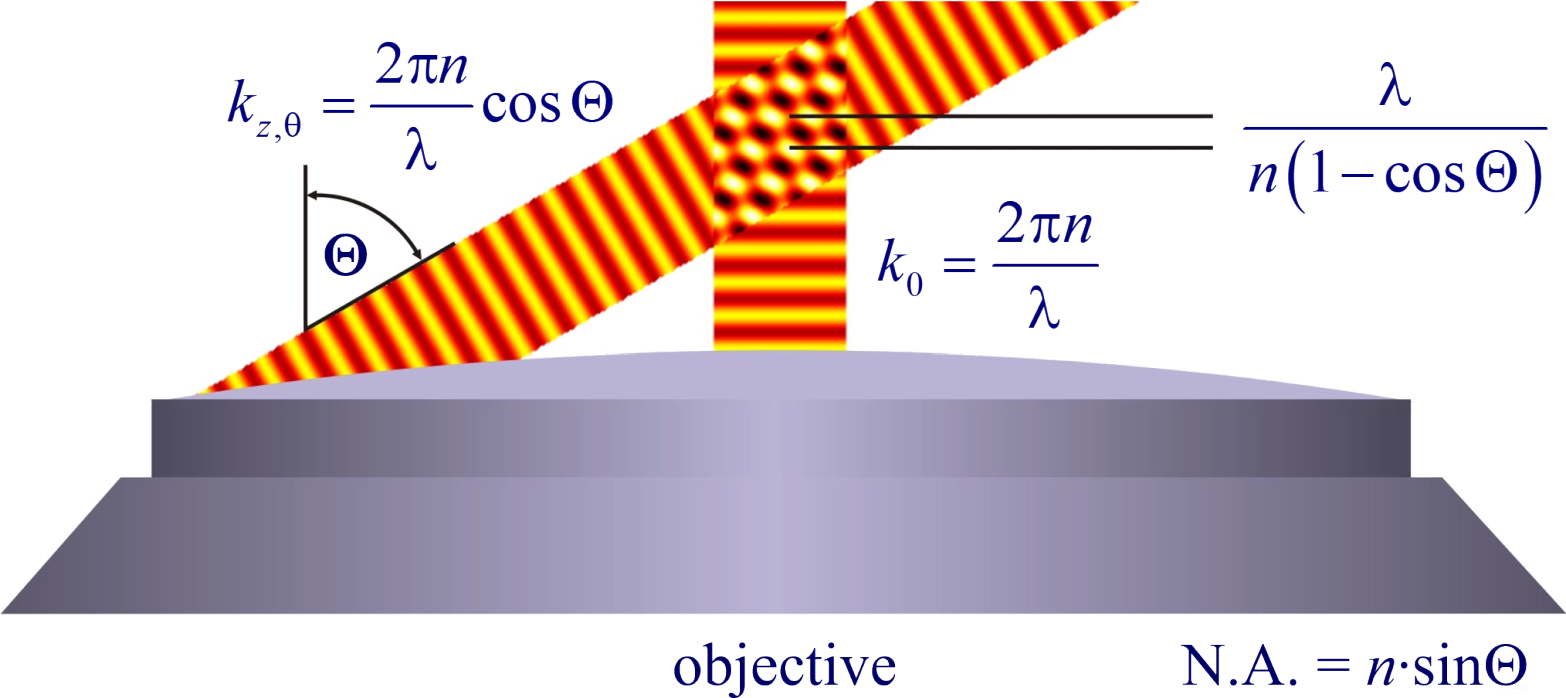
Axial resolution of a CLSM: Similar to the lateral resolution, the axial resolution is determined by the tightest spatial modulation of light that can be generated along the optical axis. This is achieved by interfering an axially propagating beam with one traveling at highest possible incidence angle. The axial component of the wave vector of the former is equal to the full wave vector length $k_{0}=2\pi n/\lambda$, and the axial component for the latter is $k_{z,\Theta }=2\pi n\;cos\Theta /\lambda$. The resulting interference therefore leads to a spatial intensity modulation along the optical axis with periodicity λ/n(1-cosΘ) which is the axial resolution limit of a CLSM.

In a similar vein, we can also obtain the axial resolution limit of a (confocal laser scanning) microscope, by asking what the tightest spatial intensity modulation is that can be achieved when focusing light through the objective. The answer is presented in Fig. 4, where this tightest modulation is now generated by the interference of an axial light ray with a light ray traveling at the highest possible incidence angle Θ. This directly yields the axial resolution limit of an optical microscope, complementary to Abbe’s lateral resolution limit, and is given by

(17) $$z_{min}=\frac{\lambda }{n(1-cos\Theta )}\approx \frac{2n\lambda }{(NA{)}^{2}},$$

where the approximation on the right hand side is valid only for small values of the numerical aperture.

**FIG. 5:**
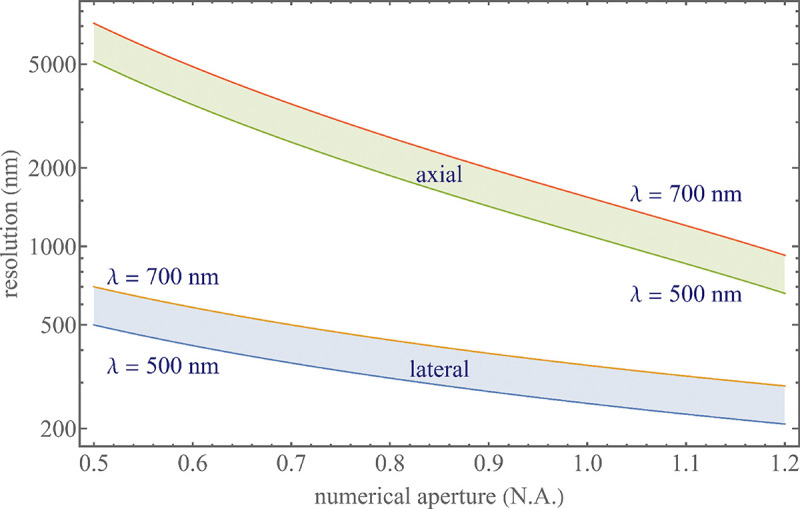
Lateral and axial resolution of diffraction-limited optical microscopy using a water immersion objective (designed for imaging in water with refractive index 1.33) as a function of numerical aperture NA and wavelength.

To get a better idea about the actual numbers for the lateral and axial resolution limit of a diffraction-limited optical microscope with water immersion objective (*i.e.*, designed for imaging in water with refractive index 1.33), all the above results are summarized by Fig. 5 showing the lateral and axial resolution as a function of numerical aperture NA and for optical wavelengths across the visual spectrum.

While providing qualitative guidance on optical system design, the axial and spatial resolution expressions provided in Eq. 16–17 remain theoretical. In particular, such expressions provide an upper bound on the resolution otherwise limited by factors including crucial notions of stochastic noise and undesired out-of-focus light.

A final important note is warranted on light (information) collection efficiency and/or suppression of out-of-focus light from parts of the specimen outside the focal plane limiting light collection to a certain axial range, *i.e.*, optical sectioning. For this purpose, different variants of specialized sample illumination and fluorescence light detection techniques have been developed, such as the Total Internal Reflection Fluorescence (TIRF) microscopy [42], Super-critical Angle Fluorescence (SAF) microscopy [43, 44], Metal-Induced Energy Transfer (MIET) microscopy [45], confocal microscopy [46], Image Scanning Microscopy (ISM) [47, 48], 4pi microscope [49], two-photon microscopy [50], Structured Illumination Microscopy (SIM) [51], light-sheet microscopy [52, 53], and multi-plane microscopy [54, 55].

All the methods mentioned accomplish optical sectioning and/or enhance photon collection efficiency in improving image resolution and contrast. These techniques pushed the optical resolution to its very limits as dictated by Abbe’s diffraction barrier. However, it was not until the end of 20th century that this barrier was overcome to achieve spatial resolutions in far-field light microscopy far beyond the diffraction limit [56]. Research in this front is still ongoing leveraging advances in four main components of fluorescent microscopes: fluorescent emitters (*i.e.*, light sources); optical setups; detectors; and analysis. In what follows, we first discuss fluorescent light sources, including their basic characteristics and then proceed to review optics of different microscope modalities while presenting statistical frameworks throughout.

## II. FLUOROPHORES

Point fluorescent emitters or light sources, often molecules termed fluorophores, are key toward fluorescence imaging of labeled samples. Both conventional fluorescence imaging, as well as advanced microscopy at high spatial resolution, rely on tunable properties of fluorophores including emission rates, brightness, absorption and emission spectra, excited state lifetimes, and other photo-physical properties such as blinking and photo-bleaching [57]. Here, we will discuss quantum fluorophore properties, their statistical modeling, and relegate classical models to Sec. III D, where we derive their emission fields.

### A. Fluorophore properties

Most molecules do not naturally fluoresce in regimes detectable by modern detectors and cannot be easily excited without inducing photo-damage. Thus, one must often resort to specific fluorescence labeling [58] of biological samples, *e.g.*, biomolecules or organelles, in order to identify and investigate structures of interest against the vast background of proteins, nucleic acids, lipids, and small molecules within a cell.

While the addition of fluorescent labels introduces challenges, their intrinsic properties as well as non-linear response to light in themselves open windows of opportunity, *e.g.*, to study molecular interactions [59, 60], determine molecular copy numbers [61–63], and improve optical resolution [64, 65] as later detailed in Sec. V.

The most common labels include: fluorescent proteins [66]; organic dyes [67, 68], generally small organic molecules containing conjugated π-electron systems; and semiconductor quantum dots, inorganic nano-crystals with especially broad excitation spectra and narrow emission spectra [69] as compared to other labels. These fluorescent labels include a large variety of fluorophores with excitation and emission wavelength maxima spanning the near-infrared, visible and UV spectrum range [70, 71]. Less common fluorescent labels, referred to as exotic labels, include carbon nanorods, carbon dots, polymer dots, fluorocubes, and fluorescent defects in diamond or 2D materials [72–74]. Exotic labels provide an even larger color palette and unique photo-physical properties.

**FIG. 6:**
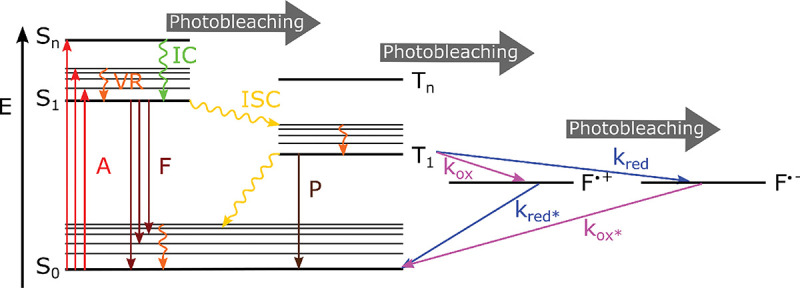
Simplified Jablonski diagram. The electronic ground state $S_{0}$, the singlet excited states $S_{n}$, the triplet excited states $T_{n}$, and radical cation $F^{\cdot +}$ or anion states $F^{\cdot -}$, along with possible transitions. The thick lines represent electronic energy levels, the thin lines vibrational energy levels, while rotational energy states are left unmarked. Here we denote: Absorption by A; Fluorescence by F; Phosphorescence by P; Vibrational Relaxation by VR; Internal Conversion by IC; Inter System Crossing by ISC; and rates of oxidation and reduction are $k_{ox}$ and $k_{\text{red}}$, respectively.

Basic fluorophore photo-physics is often captured by Jablonski diagrams. Fig. 6 shows an example of such a diagram for an organic dye. The diagram illustrates select transitions between different molecular energy and spin states. A more rigorous treatment of transition rules, molecular spectra, and interactions of light and matter, can be found in the books of Lakowicz [75] and Valeur *et al* [76].

A molecule in the (typically singlet) ground state is excited to a singlet excited state by absorbing a photon with a probability depending on the excitation light intensity and the molecule’s absorption cross-section linearly related to extinction coefficient [75]. The molar extinction coefficient $\epsilon_{\lambda }$ is a measure of how strongly a solution containing a mole of a fluorophore species absorbs (attenuates) light at a given wavelength expressed by the Lambert-Beer law [75]

(18) $$\epsilon_{\lambda }=\frac{A_{\lambda }}{c_{M}l}=\frac{log\left(I_{0\lambda }/I_{\lambda }\right)}{c_{M}l}$$

where $A_{\lambda }$ is the absorbance measured, $I_{0\lambda }$ is the initial light intensity with wavelength λ, and $I_{\lambda }$ is the light intensity after traveling the path length l through the solution with molar concentration $c_{M}$. As the Lambert-Beer law suggests, light absorption by a fluorophore depends on the excitation light wavelength and may be non-zero across wavelength range, leading to a broad absorption spectrum. This arises due to: closely spaced rotational levels associated with each vibrational level largely increasing the number of possible absorption bands (not shown in the simplified Jablonski diagram of Fig. 6); the solvation effect and electronic dephasing [77].

From $\epsilon_{\lambda }$, we immediately arrive at another important fluorophore property, namely the molecular brightness $B_{\lambda }$. To achieve high Signal to Noise Ratio (SNR), fluorescent labels with high molecular brightness, $B_{\lambda }=Q_{f}\epsilon_{\lambda }$, are desired, where $Q_{f}$ is fluorescence quantum yield. Here, $Q_{f}$ describes how many fluorescence photons are emitted relative to the number of absorbed photons. This is given by the ratio of the sum of radiative transitions to the total transitions, *i.e.*, the sum of transition rates corresponding to all transition paths out of the excited state,

(19) $$Q_{f}=\frac{\sum k_{f}}{\sum k_{f}+k_{\text{non-radiative}\;}},$$

where $k_{f}$ is the rate of fluorescence or radiative decay, and $k_{\text{non–radiative}}$ is the rate of non-radiative decay for all non-radiative decay processes.

Another fluorophore property related to the total transitions is the average time a fluorophore remains excited prior to emitting a photon given by

(20) $$\tau =\frac{1}{\sum k_{f}+k_{\text{non-radiative}}}.$$

Here, the average time τ, termed fluorescence lifetime, typically lasts on the order of a few nanoseconds for organic dyes. The fluorescence lifetime is a characteristic property of fluorophores in their unique environment tuned by pH, ion or oxygen concentration, molecular binding, or proximity dependent inter-molecular energy transfers primarily influencing the rate of non-radiative decay [75, 76]. As such, differences in fluorophore lifetimes can be employed to distinguish fluorophore species thereby broadening the appeal of Fluorescence Lifetime Imaging Microscopy (FLIM) [78, 79] in functional and multiplexed imaging of disparate fluorophores with otherwise overlapping spectra; see Sec. IV B 1.

As described above, many fluorophore properties are related to the number of possible transitions out of the excited state either non-radiatively or radiatively. For instance, upon fluorophore excitation, one such radiative transition occurs via rapid vibrational relaxation to the lowest energy level of the $S_{1}$ excited state followed by radiative decay to a vibrational ground state level with spontaneous fluorescence emission; see Fig. 6. The fluorescence emission is in general independent of the excitation wavelength (Kasha’s rule [80]) and shifted towards longer wavelengths (Stokes shift) as compared to excitation, due to fast internal conversion and vibrational relaxation to the lowest level of the $S_{1}$ excited state. Another radiative transition out of the excited state, of later interest, is stimulated emission. Typically, stimulated emission does not play a role at room temperature so long as the excitation intensity is low. However, this non-linear process is exploited in STimulated Emission Depletion (STED) super-resolution imaging [56] later described in Sec. VA1.

In addition to radiative transitions, several alternative non-radiative pathways are available for transition from the first singlet excited state, $S_{1}$, to the ground state. For instance, the molecule can also return to the ground state without emitting a photon by fast internal conversion to an electronic state of lower energy followed by further vibrational relaxation thereby dissipating the energy to the environment as heat.

An example of non-radiative transition, often employed in Single Molecule Localization Microscopy (SMLM) (see Sec. VB2) to create fluorophore blinking, is transition to the $T_{1}$ triplet state via inter-system crossing. Return from the triplet state to the ground state (phosphorescence) is typically delayed on account of a forbidden spin flip transition; see Fig. 6. As such, transitions to and from triplet, or further reduced/oxidized off-states (also referred to as bright and dark states, respectively) occur on longer timescales (0.1 ms to 100 ms timescale).

One way to control fluorophore switching between the triplet dark state to the bright states, *i.e.*, blinking, is by adjusting oxygen concentration within the solution. Upon reaction with oxygen dissolved in solution, fluorophores may transit from the triplet dark state (off-state) to the singlet ground state (on-state). This is because the oxygen molecule’s ground state is a triplet state (while its excited state is singlet), thus a fluorophore in a triplet state can transfer its energy to a ground state oxygen and return to its singlet ground state. However, another possible reaction between an oxygen molecule and a triplet fluorophore is an electron transfer. The latter, sometimes considered an undesirable phototoxic effect, results in irreversible photo-bleaching [81]. Photo-bleaching can occur from a variety of states as indicated in Fig. 6.

In some applications, such as the study of diffusion via particle tracking [82] and protein-protein interactions via FRET [83, 84], suppression of photo-bleaching and blinking are desired. One way to achieve this is by removal of oxygen from the solution and by depopulating the dark states via a system of reducing and oxidizing agents [85].

On the other hand, different dark states can be exploited to achieve super-resolution imaging [71, 86] by SMLM (see Sec. VB2) or by higher order statistical analysis of fluorophore fluctuations; see Sec. VB1. Many cyanine and rhodamine dyes can be reversibly photo-switched from a bright state to a dark state (blink) in a buffer containing enzymatic oxygen scavengers and a primary thiol such as β-mercaptoethylamine or β*-*mercaptoethanol [71, 86]. Alexa Fluor 647 is the organic dye of choice for state-of-the-art direct STochastic Optical Reconstruction Microscopy (dSTORM) imaging due to its high brightness and efficient switching behavior [87]. For several cyanines, *e.g.*, Cy5, it has been shown that thiolate anions covalently bind to the fluorophore [88], thereby disrupting the conjugated system. The dyes can also be chemically reduced by NaBH_4_ to a non-fluorescent form or synthesized in a caged form that can later be photo-activated [89, 90]. Rhodamine dyes can as well reversibly switch from a fluorescent to a non-fluorescent form by intra-molecular spirocyclization either spontaneously or driven by UV light. This has been exploited to generate sensors and switches or can be used for SMLM [71, 91].

Examples of SMLM include (fluorescence) Photo-Activated Localization Microscopy ((f)PALM) [92, 93], as well as derivatives such as single particle tracking PALM (sptPALM) [94]. In these applications, advanced fluorescent proteins have been engineered. These switch between fluorescent states through different photo-chemical reactions of the chromophore either reversibly (*e.g.*, on and off for Dronpa by cis-trans isomerization) or through photo-activated (*e.g.*, PA-GFP by decarboxylation) or photo-converted (*e.g.*, green to red wavelength for mEos by β-elimination) [66, 86].

More recently, studies of protein activity and SMLM have benefited from the discovery of a new class of ligandactivated fluorescent proteins [95]. The prototype UnaG binds the small molecule bilirubin via multiple noncovalent interactions and forms a fluorescent complex. The oxidized (and photo-bleached) ligand can detach from the protein, allowing a fresh bilirubin molecule to bind and act as a sensor for small molecules thereby reporting on protein activity [96].

In general, fluorescent proteins have the advantage that they can be genetically encoded, allowing fluorescent labeling of nearly arbitrary target proteins in living cells and organisms by creation of fusion constructs. However, this also means that the proteins have to undergo appropriate folding followed by chromophore maturation, *i.e.*, formation of a fluorescent molecule typically starting from three amino acids [66]. This process can take minutes to hours, may be incomplete and can impair the temporal accuracy of measurements of rapid processes such as gene expression dynamics [97]. However, the photo-physics and photo-chemistry of organic dyes and fluorescent proteins is often more complex than naively expected which can be, in turn, employed for a range of applications. For instance, organic dyes can experience a spectral blue shift upon high laser radiation [98, 99] useful in mechanistic studies of chemical reactions at the single molecule level [100], like the epoxidation of a double bond in conjugation to a BODIPY dye indicated by a spectral shift from substrate (green) to product state (orange) [101]. However, such spectral shifts may affect multi-color applications, *e.g.*, in super-resolution imaging or Single Particle Tracking (SPT) and is a problem in FRET experiments. Moreover, many proteins have additional dark states, *e.g.*, mEos cis-trans isomerization [102, 103], and organic dyes may have several conformations with different intensity levels, *e.g.*, Atto647N, with at least three identified states differing in fluorescent lifetimes [104] complicating quantitative single molecule read-outs.

### B. Förster resonance energy transfer

In the previous section, we discussed fluorophore properties involving radiative transitions or non-radiative transitions via interaction with the environment. Here, we continue by considering non-radiative transitions through inter-molecular energy transfer [76]. A few example of these transitions include: Photo-induced Electron Transfer (PET) [105], collisional quenching or FRET, Bioluminescence Resonance Energy Transfer (BRET) [106, 107], or the recently discovered Proximity-Assisted Photo-Activation (PAPA) [108]. Such transitions are distance dependent and thus have been leveraged to probe binding interactions or conformational changes. In what follows, we focus on FRET.

FRET is an inter-molecular energy transfer process widely used to measure molecular interactions and as a distance ruler for structural biology [75, 109]. It is a non-radiative energy transfer from a so called donor fuorophore to an acceptor fluorophore that can occur through dipole-dipole coupling with rate constant $k_{FRET}$ if the emission spectrum of the donor overlaps with the acceptor’s absorption spectrum [110].

The FRET efficiency $E_{FRET}$, *i.e.*, the probability for the energy transfer to occur, scales with the donoracceptor distance to the inverse 6*^th^* power and is 50% at the Förster radius $R_{0}$

(21) $$E_{FRET}\;=\frac{1}{1+{\left(\frac{r}{R_{0}}\right)}^{6}}\;\;=\frac{k_{FRET}}{\sum k_{f}+k_{\text{non-radiative}}}=1-\frac{\tau_{DA}}{\tau_{D}}$$

where $\tau_{DA}$ and $\tau_{D}$ are, respectively, the fluorescence lifetime of the donor in the presence and absence of the acceptor. For typical donor-acceptor pairs $R_{0}$ is a few nanometers [75] and depends on the spectral overlap of the donor emission with the acceptor absorption spectra and the relative orientation of the donor emission and acceptor absorption dipole moments

(22) $$R_{0}^{6}\;=\frac{9000\;ln10}{128\pi^{5}N_{A}n^{4}}\kappa^{2}Q_{f,D}\int I_{D}(\lambda )\epsilon_{A}(\lambda )\lambda^{4}d\lambda ,\;\;\kappa \;=3cos\theta_{D}cos\theta_{A}-cos\theta_{DA}.$$

Here, κ is the so-called orientation factor, $Q_{f,D}$ is the donor’s quantum yield in the absence of the acceptor, n is the solution’s refractive index, $I_{D}$ is the donor’s normalized fluorescence emission spectrum, $\epsilon_{A}$ is the acceptor’s molar extinction coefficient, $\theta_{DA}$ is the angle between the donor and acceptor transition moments, and $\theta_{D}$ and $\theta_{A}$ are the angles between these moments and the separation vector, respectively.

Ignoring angular dependence of energy transfer, as described in Eq. 22, can yield significant biases in inferred FRET distance assessments [111]. However, in practice, the dipoles are often freely and rapidly rotating (rapid compared to the de-excitation rate of the donor) leading to an average value of. $\kappa^{2}=\frac{2}{3}$

FRET can also occur between spectrally identical molecules (homo-FRET), and is then observed by measuring its effect on fluorescence polarization anisotropy [112]

(23) $$r=\frac{I_{∥}-GI_{⊥}}{I_{∥}+2GI_{⊥}}.$$

Here, $I_{∥/⊥}$ is the intensity measured when the polarizers in the detection path are aligned parallel/perpendicular to those in the excitation, and G is a correction factor for the difference in the instrument’s sensitivity to the two orthogonal polarization orientations.

Upon exposure to linearly polarized light, the excitation probability is highest for molecules whose absorption dipole moments are aligned parallel to the polarization vector of the exciting light. In most cases, the absorption and emission dipoles of a molecule are co-linear, so fluorescence emission remains polarized immediately after excitation. Fluorescence remains anisotropic unless the molecule rotates over the fluorescence lifetime or the excitation energy is transferred to a different molecule. Thus anisotropy or polarization measurements inform us on molecular parameters such as orientation, oligomerization or size, and environmental conditions like viscosity [112, 113]. Polarization can also be read out in super-resolution imaging, *e.g.*, using polarized light in illumination or detection and capturing polarized emission by implementing specifically engineered PSFs sensitive to polarization [114]; see Sec. VC.

Polarization, lifetime, FRET efficiency, or other photophysical markers we have discussed herein are only interesting in so far as their changes probabilistically report back on the kinetics of the underlying labeled molecule. We now turn to Markov models describing discrete molecular events to extract molecular kinetics from discrete photo-physical changes.

### C. Markov models for fluorophores

To help motivate the use of Markov modeling, we consider their applications in the analysis of FRET and the enumeration of fluorophores within a diffraction-limited Region Of Interest (ROI).

For example, observations from FRET experiments with photons individually recorded (at avalanche photodiodes abbreviated as APDs) include a set of photon arrival times along with a set of corresponding colors (wavelengths), designated by c=1,2, attributing photons to either donor or acceptor channels, respectively.

The set of photon arrival times (data) are either measured with respect to the start of the experiment, for continuous illumination [115], or with respect to the pulse immediately preceding a photon detection, such as in pulsed illumination [116]. Here, we assume continuous illumination where data consists of intervals between photon arrivals. We let K coincide with the total number of photons and denote the data with $\Delta t_{1:K}=\left\{\Delta t_{1},\ldots ,\Delta t_{K}\right\}$. The sets of inter-arrival times are then used to learn transition kinetics between system states comprised of molecular and label photo-physical states. For concreteness, we assume that molecular states coincide with conformational states of a typically large biomolecule.

To collect such data sets in typical FRET experiments, the donor is excited using an illumination laser and we assume, only for simplicity here though performed more generally in Ref. [117], that acceptors become excited exclusively through FRET. The rate of donor and acceptor emissions depends on their separation marking a conformational state and its corresponding FRET efficiency; see Sec. II B. In general, there might be many conformational states associated with different FRET efficiencies ($E_{FRET}$, Eq. 21) and these can be learned within a non-parametric paradigm [115, 117]. However, for simplicity here again, we presume two states termed high and low FRET designated by $\xi_{m},\;m=1,\;2$. We assume that photo-physical states determine whether the donor and acceptor are in the excited or ground state. Given that both donors and acceptors rarely reside simultaneously in their excited states, we only consider three possible photo-physical states: $f_{1}=(Ground,\;Ground)$, $f_{2}=(Excited,\;Ground)$, and $f_{3}=(Ground,\;Excited)$ where the first elements represent the donor’s state. The entire problem’s state space is then spanned by a set of states obtained from the tensor product of photo-physical and conformational states, which we term composite states. To facilitate the notation, we designate these composite states by $s_{m}\in \left\{\left(\xi_{1},f_{1}\right),\left(\xi_{1},f_{2}\right),\left(\xi_{1},f_{3}\right),\left(\xi_{2},f_{1}\right),\left(\xi_{2},f_{2}\right),\left(\xi_{2},f_{3}\right)\right\}$ with m=1:6.

We are now in a position to write a generative model required in constructing the likelihood used in the analysis of FRET experiments. To do so, we start from the rate matrix

(24) $$K=\left[\begin{array}{cccc} 0 & k_{s_{1}\to s_{2}} & \ldots & k_{s_{1}\to s_{6}}\\ k_{s_{2}\to s_{1}} & 0 & \ldots & k_{s_{2}\to s_{6}}\\ \vdots & \vdots & \ddots & \vdots \\ k_{s_{6}\to s_{1}} & k_{s_{6}\to s_{2}} & \ldots & 0 \end{array}\right]$$

where self-transitions are, by definition, disallowed and $k_{s_{m}\to s_{m^{\prime }}}$ is the transition rate from state $s_{m}$ to $s_{m^{\prime }}$. Furthermore, elements of the rate matrix coinciding with simultaneous conformational and photo-physical transitions are set to zero owing to their rarity.

The non-zero matrix elements therefore coincide with: 1) transitions between the two FRET conformational states $\left(k_{\xi_{1}\to \xi_{2}},k_{\xi_{2}\to \xi_{1}}\right)$ while the photo-physical states remain fixed; or 2) transitions between different photo-physical states while conformational states remain fixed. To be more precise, photo-physical transitions include donor excitation $\left(k_{s_{1}\to s_{2}}=k_{ex}\right)$, donor radiative relaxation $\left(k_{s_{2}\to s_{1}}=k_{d}\right)$, acceptor relaxation $\left(k_{s_{3}\to s_{1}}=k_{a}\right)$, FRET transition when in $\xi_{1}(k_{s_{2}\to s_{3}}=k_{FRET}^{\left(1\right)})$, and FRET transition when in $\xi_{2}(k_{s_{5}\to s_{6}}=k_{FRET}^{\left(2\right)})$. As such, written explicitly, the rate matrix for this simple case reads

(25) $$K=\left[\begin{array}{cccccc} 0 & k_{ex} & 0 & k_{\xi_{1}\to \xi_{2}} & 0 & 0\\ k_{d} & 0 & k_{FRET}^{1} & 0 & k_{\xi_{1}\to \xi_{2}} & 0\\ k_{a} & 0 & 0 & 0 & 0 & k_{\xi_{1}\to \xi_{2}}\\ k_{\xi_{2}\to \xi_{1}} & 0 & 0 & 0 & k_{ex} & 0\\ 0 & k_{\xi_{2}\to \xi_{1}} & 0 & k_{d} & 0 & k_{FRET}^{2}\\ 0 & 0 & k_{\xi_{2}\to \xi_{1}} & k_{a} & 0 & 0 \end{array}\right].$$

Observations only occur when either the donor or acceptor emits radiatively. As such, the system may visit intermediate states between photon emissions such as undergo conformational transitions. For a perfect detector, *e.g.,* ignoring detector dead time [117] and assuming complete detection efficiency (otherwise $k_{\text{ex}}$ assumes an effective excitation rate), the photon inter-arrival time is thus the total time the system spends avoiding radiative transitions.

Now to construct the likelihood for a FRET data set (inter-photon arrival times and detection channels), we begin by illustrating how such data set can be obtained from a generative model. To do so, we first designate the state of the composite system at time $t_{n}$ as $s\left(t_{n}\right)$. Next, following the notation introduced in Sec. I B (see Eq. 9), a state trajectory is constructed following Gillespie algorithm [118] by first selecting to what state we move to and then deciding when this transitions occurs

(26) $$s\left(t_{n+1}\right)|s\left(t_{n}\right)∼\;\text{Categorical}\;\left(\frac{k_{s(t)\to s_{1}}}{k_{s\left(t_{n}\right)}},\ldots ,\frac{k_{s(t)\to s_{6}}}{k_{s\left(t_{n}\right)}}\right)$$

(27) $$\delta t_{n}∼Exponential\left(k_{s\left(t_{n}\right)}\right).$$

Here, $\delta t_{n}=t_{n+1}-t_{n}$ is the time the system spends in state $s\left(t_{n}\right)$, and $k_{s\left(t_{n}\right)}$ is the escape rate out of $s\left(t_{n}\right)$, *i.e.*, sum of rates pointing out of $s\left(t_{n}\right)$. The categorical distribution introduced is treated here as the generalization of the Bernoulli albeit with more than two outcomes.

Here, Eqs. 26–27 taken together constitute what is called a generative model, *i.e.*, a model both helpful in generating the data but also in constructing the likelihood.

This generative model can indeed be further generalized to include imperfect detectors, dead time, and other artefacts such as direct acceptor excitation and cross-talk [117, 119–121].

Now we have a modeling choice. For instance, we may learn the trajectory in composite state space (states occupied across time points) and kinetic rates populating the rate matrix [116, 122]. Alternatively, as is more commonly done, we may marginalize (sum) (see Eq. 7) over all trajectories and only learn the kinetic rates.

As it is most common, we select the latter path and sum over all possible (non-radiative) paths between observations. To achieve this, we use the master equation [117, 123, 124]

(28) $$\frac{d}{dt}P(t)=P(t)G$$

describing the evolution of the probability vector P(t) collecting the probabilities of occupying different states at time t. Here, G, the generator matrix, is related to the rate matrix as follows

(29) $$G=K-\left[\begin{array}{cccc} k_{s_{1}} & 0 & \ldots & 0\\ 0 & k_{s_{2}} & \ldots & 0\\ \vdots & \vdots & \ddots & \vdots \\ 0 & 0 & \ldots & k_{s_{6}} \end{array}\right]$$

where the diagonal matrix has the same size as K and its non-zero elements coincide with the escape rates.

From the master equation, we can obtain a propagator matrix Q collecting transition probabilities over an infinitesimal period ε

(30) $$Q=\exp\left[G\varepsilon \right].$$

Therefore, given the probability vector at time t-ε,P(t-ε), the probability vector at time t is given by

(31) P(t)=P(t-ε)Q.

As such, given the initial probability vector $P_{\text{in}}$, we can find this vector at an arbitrary time by dividing the time interval into N small periods of ε

(32) $$P=P_{\text{in}\;}Q_{1}\ldots Q_{N}$$

where $Q_{1}=\ldots =Q_{N}=Q$ in the absence of observations.

However, in the presence of observations, the propagators in Eq. 32 are modified according to the transitions monitored [117]. For example, observation of no photon over the *n*th period ε signifies no radiative transitions allowing us to set $k_{a}=k_{d}=0$ for this period, which in turn results in a modified propagator, designated by $Q_{n}^{\text{non}}$. Furthermore, a photon arrival, indicating a radiative transition, forces non-radiative transition rates to be zero leading to a modified propagator $Q_{k}^{\text{rad}}$ for the *k*th photon over an infinitesimal period ε.

The likelihood of a given set of observations is now given in terms of these modified propagators [117, 125]

(33) $$P\left(\Delta t_{1:K}|K,P_{\text{in}\;}\right)\propto P_{\text{in}\;}Q_{1}^{\text{non}\;}\ldots Q_{k}^{\text{rad}\;}\ldots Q_{N}^{\text{non}\;}P_{\text{norm}\;}^{T}$$

where $P_{\text{norm}}$ is a row vector of ones.

Until now, we have only assumed a parametric framework with a fixed number of conformational states, mostly assumed to be two in the literature, namely low and high FRET [126]. Here, we abandon the assumption of *a priori* knowing the number of conformational states and extend the formulation above to the non-parametric regime applicable in more realistic situations where the number of states may be unknown. To do so, we assume an infinite number of conformational states with a load $b_{m}$ (see Sec. I B) associated to the *m*th state resulting in an infinite dimensional generator matrix

(34) $$G=\left[\begin{array}{cccccccc} k_{s_{1}} & b_{1}^{2}k_{ex} & 0 & b_{1}b_{2}k_{\xi_{1}\to \xi_{2}} & 0 & 0 & \cdots \\ b_{1}^{2}k_{d} & k_{s_{2}} & b_{1}^{2}k_{FRET}^{\left(1\right)} & 0 & b_{1}b_{2}k_{\xi_{1}\to \xi_{2}} & 0 & \cdots \\ b_{1}^{2}k_{a} & 0 & k_{s_{3}} & 0 & 0 & b_{1}b_{2}k_{\xi_{1}\to \xi_{2}} & & \cdots \\ b_{1}b_{2}k_{\xi_{2}\to \xi_{1}} & 0 & 0 & k_{s_{4}} & b_{2}^{2}k_{ex} & 0 & \cdots \\ 0 & b_{1}b_{2}k_{\xi_{1}\to \xi_{2}} & 0 & b_{2}^{2}k_{d} & k_{s_{5}} & b_{2}^{2}k_{FRET}^{\left(2\right)} & \cdots \\ 0 & 0 & b_{1}b_{2}k_{\xi_{1}\to \xi_{2}} & b_{2}^{2}k_{a} & 0 & k_{s_{6}} & \cdots \\ \vdots & \vdots & \vdots & \vdots & \vdots & \vdots & \ddots \end{array}\right].$$

Now, given the non-parametric generator matrix, we can compute the corresponding propagator matrices and use them to build the likelihood similar to Eqs. 30–33. The non-parametric posterior over the set of unknowns $\vartheta =\left\{\overset{‾}{b},K,P_{\text{in}}\right\}$ is then constructed by including a beta-Bernoulli process prior (see Sec. I B) over the loads and appropriate conjugate priors over the rest of unknowns; see box II C. Strictly speaking, in computational applications, we use large albeit finite *M* and verify that, for large enough M, the conclusions drawn are independent of M.

Finally, the obtained FRET posterior is sampled using Monte Carlo methods to deduce the set of unknowns [115–117]. The sampling procedure for the FRET posterior involves both direct sampling for loads and Metropolis-Hasting for rates; see Sec. I B.

An alternative statistical FRET framework makes use of photon counts over equal time windows, *i.e.*, bins, during the experiment rather than single photons [117, 124]. In this case, the likelihood can be derived using the fact that photon counts over fixed periods are Poisson distributed (ignoring detector noise convoluted with Poisson shot noise required of quantitative analyses) [117]. The derivation of such likelihoods is more straightforward than the single photon case [121, 127] and learning of rates (or, more accurately, transition probabilities) is achieved by invoking the Hidden Markov Model (HMM) paradigm [119]. While the traditional HMM framework has required the number of FRET states as input, more recent iterations have leveraged variational tools to determine states *e.g.*, see vbFRET [128], with recent developments in non-parametric infinite HMMs (iHHMs) now allowing posterior probabilities over states warranted by the data to be sampled simultaneously alongside kinetics [27, 121].

**FIG. 7:**
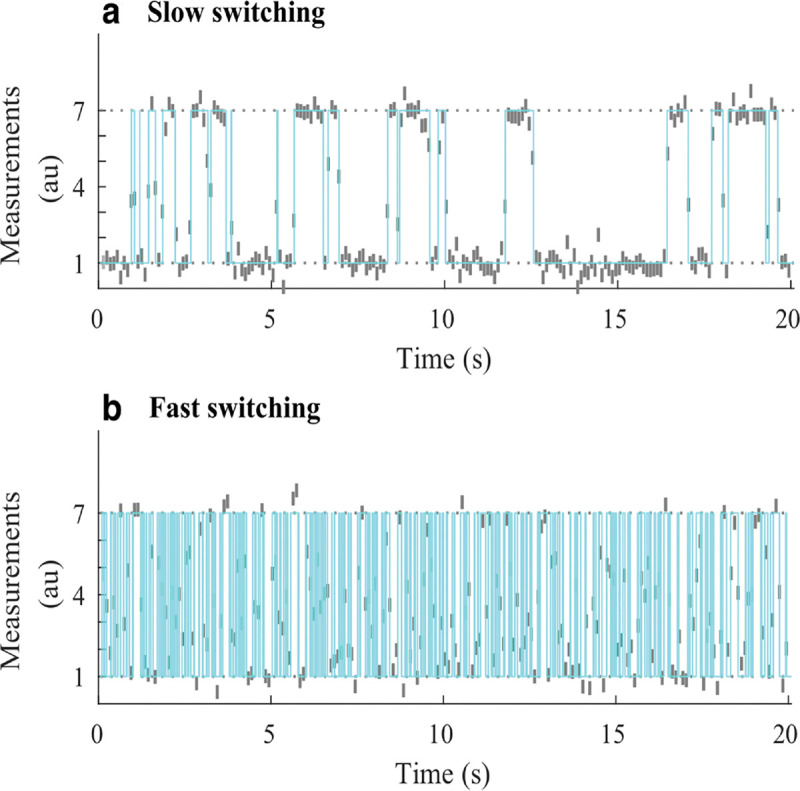
Data simulated for discrete measurements of two state systems with fast and slow transitions depicted in panels a and b, respectively. The system trajectories in the state space, measurements at different times intervals (δT), *i.e.*, bins, and the state signal levels in the absence of noise are, respectively, denoted by cyan, gray, and dotted lines. The measurements between the state signals level coincide with time intervals where the system has switched to a different state at some point during those intervals. In the simulations, data acquisitions take place at every δT=0.1s where the average time spent in each state is, respectively, 0.8 s and 0.066 s for slow and fast kinetics. Figure is adapted from Ref. [122].

However, by virtue of binning photon arrivals, whether by choice or due to the detector used, HMM frameworks naturally compromise our ability to resolve fast kinetics, occurring on timescales at or below the bin size. For this reason, other than the potential for computational speedup, there is no reason to bin single photon data. On the other hand if using detectors that unavoidably bin counts across pixels commonly used in wide-field applications (see Appendix A), then fast transitions may be deduced on timescales exceeding data acquisition by leveraging the fact that the signal amounts to an average of the properties over the state visited [122, 129, 130]; see Fig. 7.

Strategies to deduce dynamics on timescales at or exceeding data acquisition rely on the Markov jump process (MJP) [122, 131] paradigm which assumes that the system evolves in continuous time. This is by contrast to the HMM paradigm which approximates dynamics as occurring discretely and only at the measurement time. Put differently, the MJP accurately pre-supposes a continuous time trajectory 𝒮(t) in the discrete state space of the composite system generated using the same procedure as described by Eqs. 26–27. The observation for the *k*th data acquisition period (bin) is therefore [122, 130]

(35) $$w_{k}∼\;\text{Poisson}\;\left(\int_{t_{k}}^{t_{k}+\delta T}\;\;\mu_{𝒮(t)}dt\right)$$

where $\mu_{𝒮(t)}$ represents the photon emission rate for the instantaneous state occupied at time $t,\;\mu_{𝒮(t)}$.

Having briefly highlighted Markov model applications for FRET, here we turn to how Markov models are employed when enumerating fluorophores [62, 132–134] typically with the intent of determining the stoichiometry of a labeled protein complex within a diffraction-limited spot.

For a single fluorophore we assume, for simplicity of demonstration alone, a state space spanned by 3 photo-physical states, though this treatment is generalized elsewhere [62, 127]. These include the: 1) bright state, $f_{A}$; 2) dark state, $\left.f_{D};3\right)$ photo-bleached state, $f_{P}$. Transitions between these states include: $f_{A}\to f_{A},\;f_{A}\to f_{D},\;f_{A}\to f_{B},\;f_{D}\to f_{D},\;f_{D}\to f_{A},\;f_{B}\to f_{B}$. Here, the photo-bleached state is an absorbing state from which escape is impossible; see Sec. II A.

**FIG. 8:**
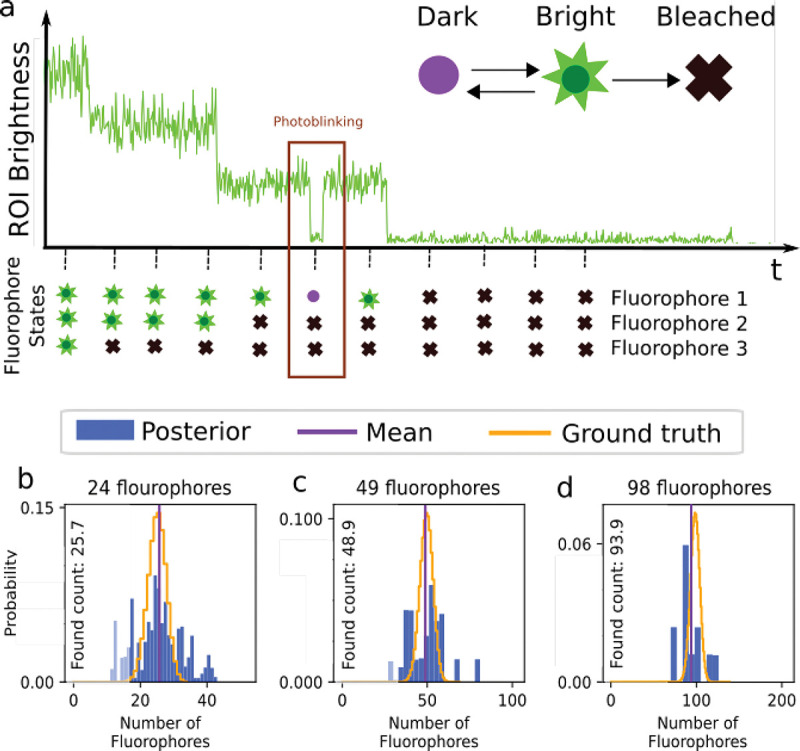
Fluorophore enumeration. (a) Cartoon representation of the enumeration problem where the ROI intensity varies as fluorophores switch between the dark, bright, and photo-bleached states. (b-d) Histogram of the sampled number of fluorophores, *i.e.*, sum of sampled loads, for experimental data with, respectively, 24, 49 and 98 fluorophores using the proposed statistical framework. The described non-parametric framework can count up to 100 fluorophores. Figure is adapted from Ref. [62].

Typically, in such applications, a wide-field detector (see Appendix A) is used to record data from ROIs containing one or multiple putative complexes. The ROIs may contain one or more pixels. The input to the analysis then consists of the sum of the intensity or brightness in each ROI typically obtained by summing the pixel values (Analogue-to-Digital Units or ADUs) in each pixel involved. The sum of ADUs over each ROI is then recorded over K successive frames and is designated by

(36) $${\overset{‾}{w}}_{1:K}=\left\{w_{1:K}^{1},\ldots ,w_{1:K}^{R}\right\}$$

where the overbar represents the set of R ROIs. Typically, the last frame is taken after all fluorophores within the ROI have photo-bleached; see Fig. 8. Assuming only photo-bleaching and ignoring transitions from bright to dark states, the number of discrete intensity drops in the time trace, if all fluorophores are initially bright, should coincide with the number of photo-bleaching events and thus the stoichiometry of the complex. However, not all fluorophores may initially be active. What is more, fluorophores blink; see Sec. II A and Fig. 8.

If our goal is to learn the number of fluorophores, assuming identical complexes across each ROI, then for independent ROIs (*iid* variables), the likelihood reads (see Sec. I B)

(37) $$P\left({\bar{w}}_{1:K}|{\bar{\Lambda }}_{1:K},\text{Ξ}\right)=\prod_{r}\prod_{k}P\left(w_{k}^{r}|{\text{Λ}}_{k}^{r},\text{Ξ}\right)$$

where Ξ and ${\overset{‾}{\Lambda }}_{1:K}$ are, respectively, the camera parameters (see Appendix A) and the elements of ${\overset{‾}{\Lambda }}_{1:K}$, namely $\Lambda_{k}^{r}$, coincide with the expected photon count, *i.e.*, brightness obtained from the emission rate multiplied by the camera exposure time, of the *r*th ROI at frame k.

Decomposed in terms of emission due to background and fluorophores, $\Lambda_{k}^{r}$ reads

(38) $${\text{Λ}}_{k}^{r}={ℬ}^{r}+I_{A}\sum_{m=1}^{M_{r}}\delta_{A,s_{k}^{rm}}$$

where m counts $M_{r}$ fluorophores within the *r*th ROI. Here $I_{A},\;{ℬ}^{r}$, and $s_{k}^{rm}$ respectively, denote the fluorophore’s brightness, background brightness of the *r*th ROI per frame, and the state of the *m*th fluorophore within the *r*th ROI at frame k. The Kronecker delta, $\delta_{A,s_{k}^{rm}}$, assumes fluorophores only emit in the bright state.

This decomposition assumes, perhaps erroneously in some cases, that the fluorphores do not interact [74].

Approximating the fluorophore state as remaining the same over each frame and the state at frame k only depending on its (potentially different) state at frame k-1, *i.e.*, the Markov assumption, we may formulate the problem using transition probabilities between different states as opposed to using transition rates. The transition probabilities for a single fluorophore can be collected as elements of a matrix, designated by Π, which is analogous to the propagator, Q in Eq. 30, for finite time windows

(39) $$\Pi =\exp\left[G\delta T\right]=\left[\begin{array}{ccc} \pi_{A\to A} & \pi_{A\to D} & \pi_{A\to B}\\ \pi_{D\to A} & \pi_{D\to D} & 0\\ 0 & 0 & 1 \end{array}\right].$$

Here, δT is the fixed period of time between measurements (frame exposure time) and each line of the transition matrix gives transition probabilities out of a certain state, for instance, $\pi_{A}=\left[\pi_{A\to A},\pi_{A\to D},\pi_{A\to B}\right]$ for the bright state. The structure of the last row uniquely reflects the nature of the bleached state as an absorbing state.

Now, the state of a single fluorophore at frame k given its states at the previous state k-1 is sampled as follows

(40) $$s_{k}^{mr}|s_{k-1}^{mr}∼Categorical(\pi_{s_{k-1}^{mr}})$$

where $\pi_{s_{k-1}^{mr}}$ collects the set of probabilities of possible transitions out of $s_{k-1}^{mr}$. Finally, as the fluorophore transitions are independent, the transition of the full system is the product of the individual fluorophore transition probabilities.

However, as the aim is to learn the number of fluorophores, we are presented with a model selection challenge. As such, we now extend the developed framework above to the non-parametric case by assuming an infinite number of fluoropbores with loads (see Sec. I B) associated to each fluorophore. This is accomplished by modifying Eq. 38 as follows

(41) $$\Lambda_{s_{k}^{mr}}={ℬ}^{r}+I_{A}\sum b_{m}^{r}\delta_{A,s_{k}^{mr}},$$

where $b_{m}^{r}$ is the load associated to the *m*th fluorophore in the *r*th ROI. In this case, the number of fluorophores is replaced by the loads for each ROI and we collect the set of unknowns in $\vartheta =\left\{\bar{\overset{‾}{b}},I_{A},\bar{ℬ},\Pi ,\bar{\bar{𝒮}}\right\}$.

Finally, to construct the posterior for the set of parameters in ϑ, we introduce priors. The most notable priors are the beta-Bernoulli process on loads and the prior on the transition probabilities, the Dirichlet prior, due to its conjugacy to the categorical distribution Eq. 40. For the remaining priors in box II C, we opt for conjugate priors (see Sec. I B) or physically motivated priors [62].

Now with the posterior at hand, we draw samples using Monte Carlo techniques to make inferences about the set of unknown parameters, ϑ. Particularly, in our sampling scheme, we use direct sampling for transition probabilities as the posterior assumes a closed form in this case. To sample the fluorophore trajectories within the state space, we use the forward filter backward sampling algorithm [22, 62, 135].

Now that we have discussed how to decode temporal data, we turn to spatiotemporal data and for this we need to include optics of fluorescence microscopes and PSFs. As such, in the following sections, we discuss optics of different microscope modalities and derive the corresponding PSFs.

## III. FLUORESCENCE MICROSCOPY: POINT SPREAD FUNCTION

In this section, we develop in a brief but self-contained manner the physical theory of optical imaging within a wide-field fluorescence microscope. We start by deriving the Abbe sine condition subsequently used to describe fundamental properties of electromagnetic wave propagation through optical systems. We then continue by deriving the basic principles of how to find the OTF and PSF of a microscope, discuss in detail the lack of optical sectioning in wide-field microscopes, and illustrate the effect of optical aberrations on PSF.

### A. Fundamental property of microscopic imaging: Abbe’s sine condition

**FIG. 9:**
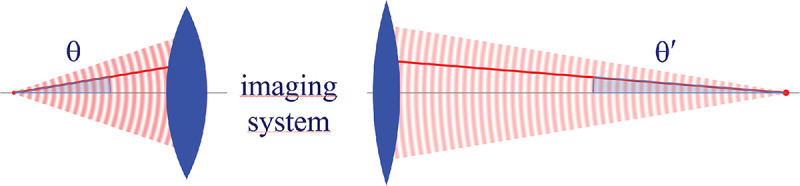
The optical microscope is a wavefront transforming system converting the outgoing spherical wavefront of a point emitter in sample space into a concentric spherical wavefront in image space (left) converging into an image point in the image space (right).

To gain a deeper understanding of how an image is formed by a microscope, and what fundamental principles govern image formation, we start by considering the imaging of a point source into an image point by a microscope as generically shown in Fig. 9. A point source in the focal plane on the optical axis (symmetry axis designated by blue lines) emits concentric (electromagnetic) waves. The segment of the spherical wavefront collected by the objective is then converted by the microscope into a segment of a spherical wavefront converging into the corresponding image point. To facilitate subsequent derivations, we assume that the distance between the sample point and the objective lens is large enough such that the spherical wavefront incident on the objective can be considered as a super-position of planar wavefront segments traveling at different propagation angles θ with respect to the optical axis (Fraunhofer diffraction limit). Correspondingly, the transformed spherical wavefront in image space is also considered to be a super-position of planar wavefront segments traveling at angles $\theta^{\prime }$ with respect to the optical axis.

**FIG. 10:**
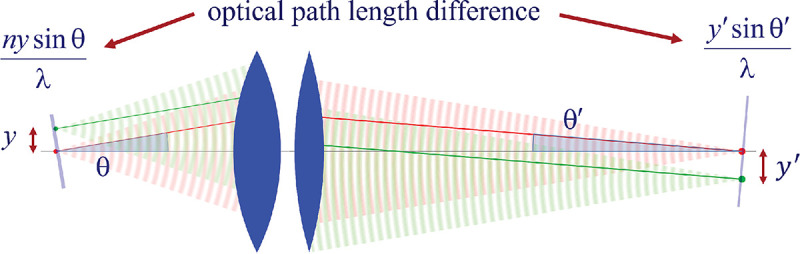
The phase relation between planar wavefront segments propagating along the same angle θ but emanating from two different point sources, where one point source is on the optical axis and the other is laterally shifted by a distance y. The image point (point of convergence of the spherical wavefront segment) corresponding to this shifted source point is shifted by a distance $y^{\prime }$ away from the optical axis. The ratio between $y^{\prime }$ and y is the magnification ℳ. Optical path length differences (phase differences) between wavefront segments traveling along angles θ or $\theta^{\prime }$, respectively, are shown as thin bluish lines at the emitters’ positions and oriented perpendicular to the considered propagation directions θ and $\theta^{\prime }$.

Here, we proceed to obtain a relation between the angle θ of a planar wavefront segment before the objective lens, and the corresponding angle $\theta^{\prime }$ of the planar wavefront segment after the tube lens. To do so, we begin by assuming that the point source is shifted laterally away from the optical axis by some distance y; see Fig. 10. If the microscope is a perfect imaging system, the spherical wavefronts from the shifted point source, shown in green, will be converted into spherical wavefronts converging onto a point shifted a distance $y^{\prime }$ away from the optical axis in the image space. The relation between $y^{\prime }$ and y is given by $y^{\prime }=ℳy$, where ℳ denotes the microscope’s magnification. Now, consider two planar wavefront segments traveling at angle θ from sources located at y and on the optical axis. There is a phase difference between these two planar wavefront segments proportional to nysinθ. The microscope transforms these planar wavefront patches into two planar wavefront patches traveling along angle $\theta^{\prime }$ in the image space with a phase difference of $y^{\prime }\;sin\theta^{\prime }$ between the patches (assuming here and throughout the paper that the refractive index of the image space is always that of air, *i.e.*, ≈ 1.0). Now, to attain perfect focus, all plane wave contributions converging at the focal point must have the same phase at that point (maximum constructive interference). In other words, the phases of all planar wave components constituting the spherical wavefront must be the same in the image point where the spherical wavefront converges. We thus find $ny\sin\theta =y^{\prime }sin\;\theta^{\prime }$. When taking into account that the ratio between $y^{\prime }$ and y is the image magnification, this yields

(42) $$n\sin\theta =ℳ\sin\theta^{\prime },$$

which is the so-called Abbe sine condition [136, 137] for a perfect aplanatic imaging system (*i.e.*, emission from a point at lateral distance x in the focal plane in sample space is converted into a perfect spherical wavefront segment converging into an image point at position $y^{\prime }=ℳy$ in the image plane).

**FIG. 11:**
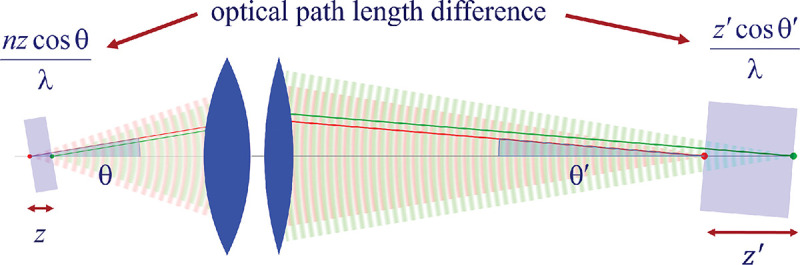
Phase relation between planar wavefront segments propagating along the same angle θ but emanating from two different point sources along the optical axis. Similar to Fig. 10, optical path length differences (phase differences) between wavefront segments traveling along angles θ or $\theta^{\prime }$, respectively, are shown as bluish rectangles.

Invoking similar arguments, we can derive the relation between θ and $\theta^{\prime }$ required for perfect imaging of point sources along the optical axis into corresponding image points in image space. This situation is illustrated in Fig. 11 where we again compare the phase differences between: 1) wavefronts from the point source in the focal plane with the shifted point source; and 2) corresponding wavefronts converging in the image points. As such, we now find the following relation between θ and $\theta^{\prime }$ (up to an additive constant)

(43) $$n\cos\theta ={ℳ}_{z}cos\;\theta^{\prime }$$

where ${ℳ}_{z}$ denotes the *axial* magnification [138, 139]. This implies that for an imaging system that perfectly images points along the optical axis, the axial magnification can only be equal to the refractive index $\left({ℳ}_{z}=n\right)$. As can be easily seen, it is *impossible* for both the Abbe sine condition and Eq. 43 to be simultaneously satisfied. This shows that an optical system which perfectly images points from the focal plane into a conjugate image plane can do that only for these two specific planes. Such an optical system will, however, exhibit aberrations, *i.e.*, deviations of wavefronts from spherical shape, and thus image imperfectly when moving away from the focal plane. Yet, for small values of angle θ, we can expand Eq. 43 into a Taylor series up to the first order, *i.e.*, $n\left(1-\theta^{2}/2\right)\approx {ℳ}_{z}\left(1-\theta^{\prime 2}/2\right)$, which can be satisfied simultaneously with Abbe’s sine condition (up to some constant) if

(44) $$n\;{sin}^{2}\theta /2\approx {ℳ}_{z\;}{sin}^{2}\theta^{\prime }/2$$

and

(45) $${ℳ}_{z}\approx \frac{{ℳ}^{2}}{n}$$

Eq. 44 is also called Herschel’s condition [138–141], and Eq. 45 shows that a system satisfying Abbe’s sine condition (aplantic imaging system) has an axial magnification that is roughly the square of the lateral magnification divided by the refractive index of the sample medium.

### B. Electromagnetic field of image formation

**FIG. 12:**
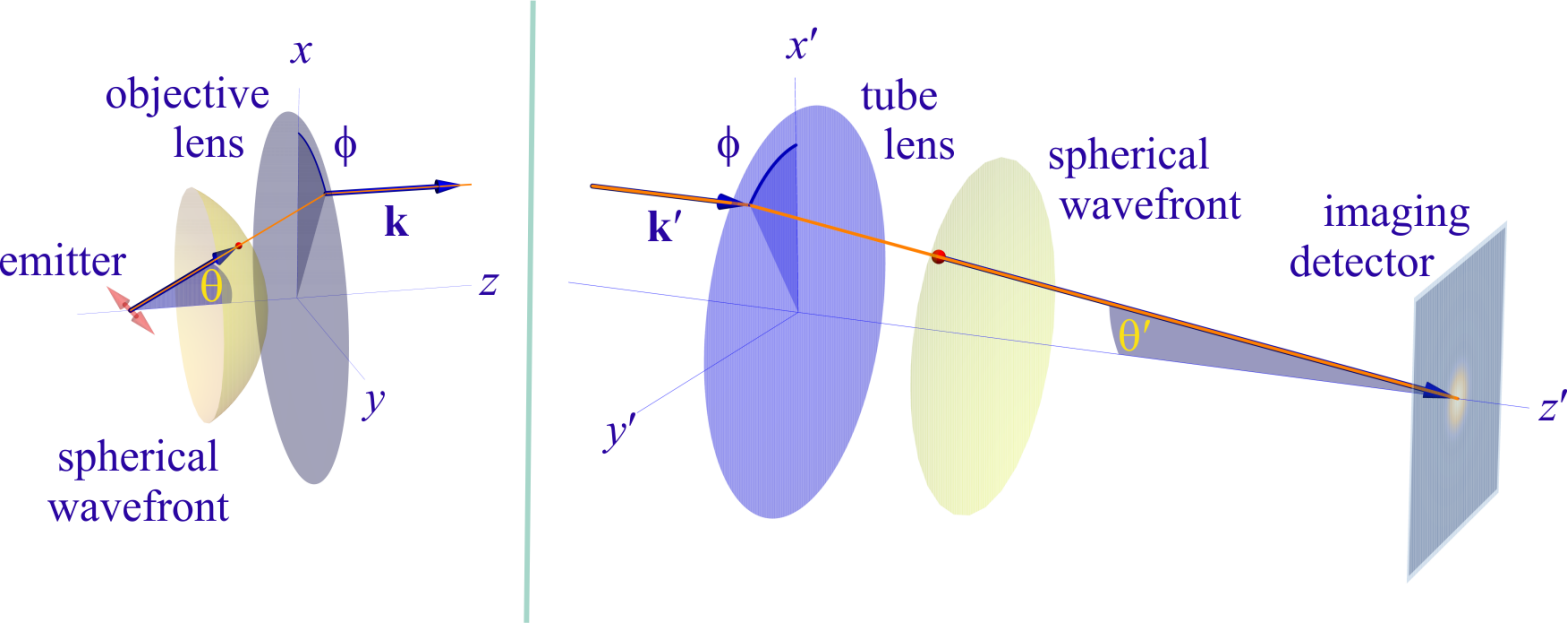
Geometry of propagation of a narrow section of the wavefront from the emitter to the image plane.

In this section, we consider a point emitter with incoherent emission in the sample space and proceed to derive a relation between the corresponding electromagnetic fields in the sample and image spaces. Specifically, we will work in the Fourier domain to derive electric and magnetic field components in the image space in terms of the electric fields of emission in the sample space. To do so, we denote parameters associated to the image and sample spaces with and without prime, respectively, hereafter. We now begin by the plane wave representation (Fourier representation) of the electric field of this emitter in the sample space

(46) $$E\left(r\right)=\int_{0}^{\Theta }\;d\theta \sin\theta \int_{0}^{2\pi }\;d\phi E_{0}\left(\theta ,\phi \right)\exp\left(ik\cdot r\right),$$

where r is the position vector in sample space with respect to the objective focal point. Moreover, $E_{0}(\theta ,\phi )$ is the electric field amplitude for the plane wave traveling along wave vector k with length |k|=2πn/λ and direction $\overset{ˆ}{k}=(cos\;\phi \;sin\;\theta ,sin\;\phi \;sin\;\theta ,cos\;\theta )$ (a hat above a vector always designates a unit vector with components (x,y,z) in Cartesian coordinates); see Fig. 12. Further, the angular integration extends over the whole cone of light with angle Θ detected by the objective (recalling that nsinΘ is the objective’s numerical aperture; see Fig. 2).

Considering a plane on which both the optical axis (z-axis in Fig. 12) and k lie, then it is convenient to split the electric field amplitude $E_{0}(\theta ,\phi )$ into two orthogonal polarization components, both parallel and perpendicular to this plane, as follows

(47) $$E_{0}=E_{0,∥}(\theta ,\phi ){\overset{ˆ}{e}}_{∥}+E_{0,⊥}(\theta ,\phi ){\overset{ˆ}{e}}_{⊥},$$

where $E_{0,∥}$ and $E_{0,⊥}$ are the corresponding electric field amplitudes along the two polarization orientations, and the corresponding unit vectors denoted by ${\overset{ˆ}{e}}_{∥}$ and ${\overset{ˆ}{e}}_{⊥}$. These two unit vectors with the unit vector $\overset{ˆ}{k}$ form an orthonormal set of unit vectors, given as follows in Cartesian coordinates

(48) $$\overset{ˆ}{k}\;=(cos\;\phi \;sin\;\theta ,sin\;\phi \;sin\;\theta ,cos\;\theta ),\;{\overset{ˆ}{e}}_{∥}\;=(-sin\;\phi \;,cos\;\phi \;,0),\;{\overset{ˆ}{e}}_{⊥}\;={\overset{ˆ}{e}}_{∥}\times \overset{ˆ}{k}=(cos\;\phi \;cos\;\theta ,sin\;\phi \;cos\;\theta ,-sin\;\theta ).$$

This representation immediately allows us to write down the magnetic field in sample space. We do so by recalling that for a plane wave with wave vector k and electric field amplitude $E_{0}$ the magnetic field amplitude is $B_{0}=$ $n\overset{ˆ}{k}\times E_{0}$ [142]. Thus, the magnetic field amplitude in sample space reads

(49) $$B_{0}=n\left[-E_{0,∥}(\theta ,\phi ){\overset{ˆ}{e}}_{⊥}+E_{0,⊥}(\theta ,\phi ){\overset{ˆ}{e}}_{∥}\right].$$

The microscope’s optics now converts each of the plane wave components in Eq. 46 into a corresponding plane wave component $E_{0}^{\prime }\left(\theta^{\prime },\phi \right)exp\left(ik^{\prime }\cdot r^{\prime }\right)$ in the image space; see right panel of Fig. 12, Here, $r^{\prime }$ is now centered at the focus of the tube lens, the angle ϕ remains the same, and the propagation angles θ and $\theta^{\prime }$ are connected via Abbe’s sine condition. As before, it is convenient to split the electric field amplitude into two principal polarization directions,

(50) $$E_{0}^{\prime }=E_{0,∥}^{\prime }(\theta ,\phi ){\overset{ˆ}{e}}_{∥}+E_{0,⊥}^{\prime }(\theta ,\phi ){\overset{ˆ}{e}}_{⊥}^{\prime }$$

where the set of unit vectors in the image space is obtained by substituting θ by $\theta^{\prime }$ in Eq. 48. Moreover, we note that ${\overset{ˆ}{e}}_{∥}^{\prime }={\overset{ˆ}{e}}_{∥}$ due to its independence from θ. Now, the corresponding magnetic field amplitude can be obtained as

(51) $$B_{0}^{\prime }=-E_{0,∥}^{\prime }\left(\theta^{\prime },\phi \right){\overset{ˆ}{e}}_{⊥}^{\prime }+E_{0,⊥}^{\prime }\left(\theta^{\prime },\phi \right){\overset{ˆ}{e}}_{∥}$$

assuming that the refractive index in image space is unity (air).

We still have to relate the electric field amplitudes in sample and image spaces. This can be found by considering the conservation of the energy flux density along the optical axis for every plane wave component. This flux density is given by the z-component of the time-averaged Poynting vector P [142]. Therefore, we can write

(52) $$P_{z}=\frac{c}{8\pi }{\overset{ˆ}{e}}_{z}\cdot (E_{0}\times B_{0}^{*})=\frac{c}{8\pi }{\overset{ˆ}{e}}_{z}\cdot (E_{0}^{\prime }\times B_{0}^{\prime *})$$

where a star denotes complex conjugation. Taking into account that $B_{0}=n\overset{ˆ}{k}\times E_{0}$ in sample space and $B_{0}^{\prime }={\overset{ˆ}{k}}^{\prime }\times E_{0}^{\prime }$ in image space, we obtain

(53) $$n{\left|E_{0}\right|}^{2}cos\;\theta ={\left|E_{0}^{\prime }\right|}^{2}cos\;\theta^{\prime }.$$

which yields the following relation between the electric field amplitudes in image and sample spaces

(54) $$\left|E_{0}^{\prime }\right|=\sqrt{n\frac{cos\;\theta }{cos\;\theta^{\prime }}}\left|E_{0}\right|.$$

Furthermore, by using Abbe’s sine condition $\;sin\theta =ℳsin\theta^{\prime }$, Eq. 42 and its differential $n\;cos\;\theta d\theta =ℳ\;cos\;\theta^{\prime }d\theta^{\prime }$, we have

(55) $$\sin\theta d\theta ={\left(\frac{ℳ}{n}\right)}^{2}\frac{\cos\theta^{\prime }}{\cos\theta }\sin\theta^{\prime }d\theta^{\prime }.$$

By substituting the above expression into the plane wave representation of the electric field, Eq. 46, and taking into account that the electric field amplitude is converted according to Eq. 54, we arrive at the following expression for the plane wave representation of the electric field in image space

(56) $$E^{\prime }\left(r^{\prime }\right)=\frac{{ℳ}^{2}}{n^{3/2}}\int_{0}^{\Theta^{\prime }}\;\;d\theta^{\prime }sin\theta^{\prime }\sqrt{\frac{cos\;\theta^{\prime }}{cos\;\theta }}\int_{0}^{2\pi }\;\;d\phi \;\;\left[E_{0,∥}{\overset{ˆ}{e}}_{∥}+E_{0,⊥}{\overset{ˆ}{e}}_{⊥}^{\prime }\right]exp\left(ik^{\prime }\cdot r^{\prime }\right)$$

where the maximum integration angle is now $\Theta^{\prime }=arcsin(n\;sin\;\Theta /ℳ)=arcsin(NA/ℳ)$. Similarly, for the magnetic field, we find

(57) $$\begin{array}{rr} & B^{\prime }\left(r^{\prime }\right)=\frac{{ℳ}^{2}}{\sqrt{n}}\int_{0}^{\Theta^{\prime }}\;\;d\theta^{\prime }sin\theta^{\prime }\sqrt{\frac{cos\;\theta^{\prime }}{cos\;\theta }}\int_{0}^{2\pi }\;\;d\phi \\ & \left[-E_{0,∥}{\overset{ˆ}{e}}_{⊥}^{\prime }+E_{0,⊥}{\overset{ˆ}{e}}_{∥}\right]\exp\left(ik^{\prime }\cdot r^{\prime }\right). \end{array}$$

**FIG. 13:**
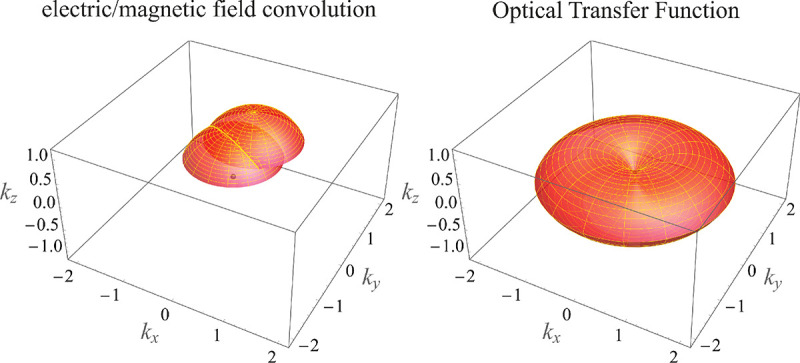
From electric/magnetic field to intensity. The two spherical caps in the left panel show the support of the Fourier representations of electric and magnetic fields given by Eq. 59. The right panel represents the convolution of the two caps on the left yielding the imaging OTF given by Eq. 65. Note that while the extent of the electric and magnetic fields in the left panel are limited to the surface areas, the right panel represents the maximum extent of the imaging OTF and the imaging OTF is also non-zero for all spatial frequencies inside this shape termed butterfly shape. The missing cone of the butterfly shape (in the middle) highlights a wide-field microscope’s inability to collect sufficient axial frequencies and thus lack of optical sectioning.

**FIG. 14:**
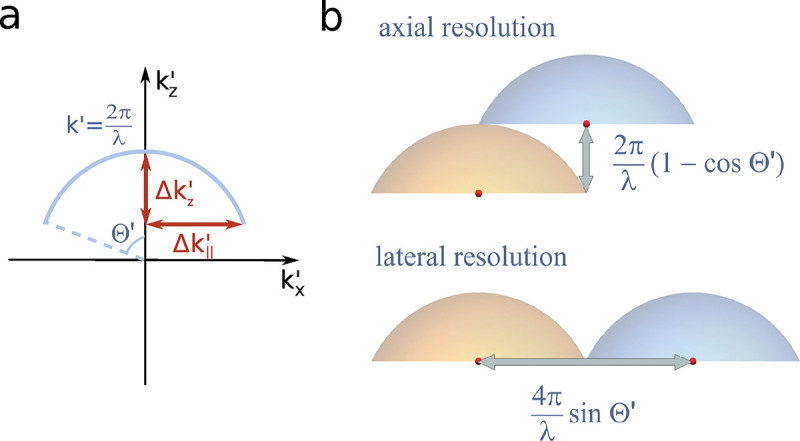
Visualization of the maximum axial and lateral extents of the Fourier representation of the electric field and the imaging OTF. (a) A cross-section of the Fourier representation of the electric field (cap) at $k_{y}^{\prime }=0$. The cross-section is an arc with radius $k^{\prime }=2\pi /\lambda$ and $0\leq \theta^{\prime }<\Theta^{\prime }$. The maximum extents of the cap along the lateral and axial directions are, respectively, given by $\Delta k_{∥}^{\prime }=\frac{2\pi }{\lambda }sin\Theta^{\prime }$ and $\Delta k_{z}^{\prime }=\frac{2\pi }{\lambda }\left(1-cos\Theta^{\prime }\right)$. (b) Here we show the convolution of the caps associated to the electric and magnetic fields along the largest axial and lateral extents beyond which the convolution is zero (see also Fig. 13).

In what follows, it is important to recognize that the above equations for the electric and magnetic field components are nothing other than Fourier representations (expansion into plane waves $\left.exp\left(ik^{\prime }\cdot r\right)\right)$, with a frequency support restricted to the spherical cap in the frequency domain defined by radius $\left|k^{\prime }\right|={\left(k_{x}^{\prime 2}+k_{y}^{\prime 2}+k_{z}^{\prime 2}\right)}^{1/2}=2\pi /\lambda ,\;0\leq \theta^{\prime }\leq \Theta^{\prime }$ (or equivalently $0\leq {\left(k_{x}^{\prime 2}+k_{y}^{\prime 2}\right)}^{1/2}\leq \left.(2\pi /\lambda )sin\;\Theta^{\prime }\right)$ and 0<ϕ≤2π. In other words, the Fourier amplitudes of the electric and magnetic fields are only non-zero on this spherical cap in Fourier space, see also left panel of Fig. 12. To better see this, we rewrite the above equation (Eq. 56) as

(58) $$E^{\prime }\left(r^{\prime }\right)=\int \frac{d^{3}k^{\prime }}{(2\pi {)}^{3}}{\tilde{E}}^{\prime }\left(k^{\prime }\right)exp\left(ik^{\prime }\cdot r^{\prime }\right)$$

where a variable with tilde denotes Fourier representation of the variable hereafter. The three-dimensional integration now extends over the whole k-space, the integration measure in spherical coordinates is $d^{3}k^{\prime }=k^{\prime 2}sin\theta^{\prime }dk^{\prime }d\theta^{\prime }d\phi$, and the electric field Fourier amplitude (integrand in Eq. 58) for angles $0\leq \theta^{\prime }\leq \Theta^{\prime }$ is given by (all constant pre-factors omitted)

(59) $${\tilde{E}}^{\prime }\left(k^{\prime }\right)\propto \delta \left(k^{\prime }-\frac{2\pi }{\lambda }\right)\sqrt{\frac{cos\;\theta^{\prime }}{cos\;\theta }}\left(E_{0,∥}{\overset{ˆ}{e}}_{∥}+E_{0,⊥}{\overset{ˆ}{e}}_{⊥}^{\prime }\right)$$

while it is zero for angles $\theta^{\prime }>\Theta^{\prime }$. Here, δ denotes Dirac’s delta function and guarantees that $k^{\prime }=\left|k^{\prime }\right|=2\pi /\lambda$. As such, the Fourier representation of the electric field is only non-zero for spatial frequencies over a spherical cap of radius 2π/λ and $0\leq \theta^{\prime }\leq \Theta^{\prime }$, which can be also expressed by (see left panels in Figs. 13–14)

(60) $$\left|{\tilde{E}}^{\prime }\right|\propto \left\{ \begin{array}{l} \sqrt{\frac{\cos\theta^{\prime }}{\cos\theta }\left(E_{0,∥}^{2}+E_{0,⊥}^{2}\right)},\;k^{\prime }=\frac{2\pi }{\lambda }\;\&\;0\leq \theta^{\prime }\leq \Theta^{\prime }\\ \;0,\;\mathrm{otherwise}. \end{array}\right.$$

A similar expression holds for the Fourier representation of magnetic field, when replacing $E_{0,⊥}$ by $-nE_{0,∥}$ and $E_{0,∥}$ by $nE_{0,⊥}$. The electric and magnetic field Fourier amplitudes (${\tilde{E}}^{\prime }$ and ${\tilde{B}}^{\prime }$) in Eqs. 59–60 are also called the *Optical Transfer Function* or OTF of the electric and magnetic fields. However, to avoid any confusion with the imaging OTF (Fourier representation of the (imaging) PSF), we simply refer to ${\tilde{E}}^{\prime }$ and ${\tilde{B}}^{\prime }$ as Fourier representations of the electric and magnetic fields hereafter.

### C. Point spread function

Now, we are in a position to calculate the PSF, denoted by $U\left(r^{\prime }\right)$. The PSF is, by its very nature, a probability density over a photon reaching the point $r^{\prime }$ on image plane, *i.e*., detector, where $r^{\prime }$ is a random variable. From this fundamental probabilistic property of light follows most of the statistical concepts inherent to the modeling of fluorescence microscopy imaging. Furthermore, the PSF plays the role of a normalized spatial distribution of light intensity recorded by a detector at the image plane for a point-like emitter located at the focal point in the sample space.

This intensity distribution is again given by the z-component of the Poynting vector a

(61) $$U\left(r^{\prime }\right)\;=\frac{c}{8\pi }{\overset{ˆ}{e}}_{z}\cdot [E^{\prime }\left(r^{\prime }\right)\times B^{\prime *}\left(r^{\prime }\right)]\;\;=\frac{c}{8\pi }[E_{x}^{\prime }\left(r^{\prime }\right)B_{y}^{\prime *}\left(r^{\prime }\right)-E_{y}^{\prime }\left(r^{\prime }\right)B_{x}^{\prime *}\left(r^{\prime }\right)].$$

When knowing this PSF, the image model $\Lambda \left(r^{\prime }\right.$) (expected, *i.e*., mean, spatial distribution of intensity, *i.e*., photon count, in image space) for an arbitrary sample is given by the convolution

(62) $$\Lambda \left(r^{\prime }\right)=I\int d^{3}r_{0}U\left(r^{\prime }-ℳr_{0}\right)S\left(r_{0}\right)$$

where $S\left(r_{0}\right)$ is the so-called sample function describing the fluorophore distribution. We further assumed the PSF, U, to be normalized to unity and I to reflect the total photon emission per fluorophore.

For an aplanatic imaging system, which is shiftinvariant (see Sec. III F), Eq. 62 is exact for all emitters on the focal plane, *i.e*., for $z_{0}=0$. However, it is an approximate expression for emitters outside the focal plane, as should be apparent from the discussion of the Abbe and Herschel conditions in Sec. III A.

Using the Fourier representation of Eq. 59, the lateral components of the electric and magnetic fields in the Fourier domain are explicitly given by (for $\theta^{\prime }\leq \Theta^{\prime }$)

(63) $$\left(\begin{array}{c} {\tilde{E}}_{x}^{\prime }\\ {\tilde{E}}_{y}^{\prime } \end{array}\right)\propto \delta \left(k^{\prime }-\frac{2\pi }{\lambda }\right)\sqrt{\frac{cos\;\theta^{\prime }}{cos\;\theta }}\;\;\left(\begin{array}{c} -E_{0,∥}sin\;\phi +E_{0,⊥}cos\;\theta^{\prime }cos\;\phi \\ E_{0,∥}cos\;\phi +E_{0,⊥}cos\;\theta^{\prime }sin\;\phi \end{array}\right)$$

and

(64) $$\left(\begin{array}{l} {\tilde{B}}_{x}^{\prime }\\ {\tilde{B}}_{y}^{\prime } \end{array}\right)\propto \left(k^{\prime }-\frac{2\pi }{\lambda }\right)\sqrt{\frac{cos\;\theta^{\prime }}{cos\;\theta }}\;\;\left(\begin{array}{c} -E_{0,∥}cos\;\theta^{\prime }cos\;\phi -E_{0,⊥}sin\;\phi \\ -E_{0,∥}cos\;\theta^{\prime }sin\;\phi +E_{0,⊥}cos\;\phi \end{array}\right),$$

where we also used the Cartesian representation of ${\overset{ˆ}{e}}_{∥}$ and ${\overset{ˆ}{e}}_{⊥}^{\prime }$ similar to Eq. 48. Moreover, we remember that the refractive index in image space is assumed to be 1 (air). Thus, no additional prefactor appears in the expression of the magnetic field.

Now, with the Fourier representations of the electric and magnetic fields at hand, we derive the imaging OTF and subsequently the PSF. To start, we note that the PSF is given by products of the electric and magnetic field components in the spatial domain; see Eq. 61. However, to work within the Fourier domain, we use the wellknown convolution theorem: the Fourier representation of the product of two functions is proportional to the convolution of their Fourier representations. As such, the OTF is given by

(65) $$\tilde{U}\left(k^{\prime }\right)\propto {\tilde{E}}_{x}^{\prime }\left(k^{\prime }\right)⊗{\tilde{B}}_{y}^{\prime *}\left(k^{\prime }\right)-{\tilde{E}}_{y}^{\prime }\left(k^{\prime }\right)⊗{\tilde{B}}_{x}^{\prime *}\left(k^{\prime }\right)\;\;=\int d^{3}k^{\prime \prime }[{\tilde{E}}_{x}^{\prime }\left(k^{\prime }-k^{\prime \prime }\right){\tilde{B}}_{y}^{\prime *}\left(k^{\prime \prime }\right)\;\;-{\tilde{E}}_{y}^{\prime }\left(k^{\prime }-k^{\prime \prime }\right){\tilde{B}}_{x}^{\prime *}\left(k^{\prime \prime }\right)],$$

where ⊗ denotes convolution. The obtained OTF is then related to the PSF via the Fourier transformation

(66) $$U\left(r^{\prime }\right)=\int \frac{d^{3}k^{\prime }}{(2\pi {)}^{3}}\tilde{U}\left(k^{\prime }\right)\exp\left(ik^{\prime }\cdot r^{\prime }\right).$$

The convolution of Eq. 65 is visualized in Fig. 13. The two spherical caps (note that it is only the area on the surface) shown in the left panel represent regions where the Fourier amplitudes of the electric and magnetic fields are non-zero. The convolution of these caps results in the butterfly-shaped three-dimensional figure shown in the right panel, where the shown surface represents the maximum extent of frequency support of the imaging OTF. That is, the OTF vanishes for all frequencies outside this region and takes non-zero values only for frequencies inside the three-dimensional shape.

From Fig. 14a, one finds that the lateral and axial extents of the Fourier representations of electric/magnetic fields are $\Delta k_{∥}^{\prime }=2\pi \;sin\;\Theta^{\prime }/\lambda$ and $\Delta k_{z}^{\prime }=2\pi (1-\left.cos\;\Theta^{\prime }\right)/\lambda$, respectively. As the OTF is computed from the auto-convolution of the cap associated to this electric/magnetic fields, the lateral and axial size of the OTF, respectively, is then found to be $4\pi \;sin\;\Theta^{\prime }/\lambda$ and $2\pi \left(1-cos\;\Theta^{\prime }\right)/\lambda$, see Fig. 14b.

Thus, the microscope does not transmit any lateral spatial frequencies beyond $k_{∥}^{\prime }>4\pi \;sin\;\Theta^{\prime }/\lambda$ or any axial spatial frequencies beyond $k_{z}^{\prime }>2\pi (1-\left.cos\;\Theta^{\prime }\right)/\lambda$, where $k_{∥}^{\prime }=\sqrt{k_{x}^{\prime 2}+k_{y}^{\prime 2}}$ is the amplitude of the projection of $k^{\prime }$ in the *xy*-plane. Thus, the three-dimensional intensity distribution in image space does not contain lateral spatial modulations smaller than $2\pi /\;max{\;k}_{∥}^{\prime }=\lambda /\left(2\;sin\;\Theta^{\prime }\right)$, which leads to spatial modulations of ℳλ/(2nsinΘ) in image space using Abbe’s sine condition, and translates into the smallest discernible spatial variation λ/(2nsinΘ) in the sample space when taking into account that the lateral magnification is ℳ. Thus, we recover Abbe’s resolution limit, Eq. 16, as 2π over the largest lateral spatial frequency transmitted by the microscope from sample to image space

(67) $$r_{min}^{l}=\frac{2\pi }{k_{max}^{l}},$$

where $r_{min}^{l}$ and $k_{max}^{l}$, respectively, denote the resolution and maximum extent of the OTF along the *l*th direction. While Eq. 67 provides a measure of resolution for lens-based imaging systems with OTF magnitudes consisting of a single lobe monotonically decaying to zero, *e.g.*, lateral magnitude of wide-field’s OTF (see Fig. 13) microscope, it should be used with care for more complicated OTFs, *e.g.*, axial resolution for wide-field microscope (see Figs. 13 and Sec. III E), SIM (see Sec. IV D), some types of light-sheet microscopes with multiple gaps in their OTF magnitudes (see Sec. IV E), and others.

As such, regarding the wide-field microscope’s axial resolution, the situation is more complicated due to the shape of the imaging OTF in the axial direction. To be more concrete, in the right panel of Fig. 13, one can see that the butterfly shape imaging OTF does not support frequencies within a cone of frequencies defined by $k_{z}^{\prime }/k_{∥}^{\prime }>tan\;\Theta^{\prime }$. This is often called the missing cone of the OTF. One effect of this missing cone is that a widefield microscope does not provide *optical sectioning* (z-sectioning). That is, for $k_{∥}^{\prime }\approx 0$ a wide-field microscope does not collect much axial spatial frequencies. As such, the PSF pattern formed by light collected from a fluorophore using a wide-field microscope varies very slowly with fluorophore’s axial position. Thus, a wide-field microscope is incapable of determining the axial position of this fluorophore with high precision.

Yet, as can also be seen from Fig. 13 axial frequencies $k_{z}^{\prime }$ have non-zero amplitudes for $0<k_{∥}^{\prime }<max{\;k}_{∥}^{\prime }=4\pi \;sin\;\Theta^{\prime }/\lambda$. The maximal value $k_{z}^{\prime }=2\pi \left(1-cos\;\Theta^{\prime }\right)/\lambda$ contained in the OTF shows that the smallest possible spatial modulation of the PSF along the optical axis is approximately $\lambda /\left(1-cos\;\Theta^{\prime }\right)$. For paraxial optics, *i.e.*, for small values of Θ and $\Theta^{\prime }$, where we have approximately an axial magnification ${ℳ}_{z}={ℳ}^{2}/n$ (see Eq. 45), and with the approximation $1-cos\;\Theta^{\prime }\approx \Theta^{\prime 2}/2\approx n^{2}\Theta^{2}/2{ℳ}^{2}$ this translates into a smallest axial modulation of $2\lambda /n\Theta^{2}\approx 2n\lambda /(NA{)}^{2}$ of the sample function that is still transmitted through the microscope. This is in accordance with our previous estimate of the axial resolution limit in Eq. 17. The problem of the missing cone of OTF, *i.e.*, missing z-sectioning, is considered in Sec. IV where we will discuss confocal microscope and other modalities.

### D. Electromagnetic field emission of an oscillating electric dipole

In previous section, we derived integral expressions for the OTF and PSF of a wide-field microscope; see Eqs. 61 and 65–66. In this section, we will perform these integrals and obtain the exact OTF and PSF of a wide-field microscope. To do so, we first need to compute the integrand, which is the electric field $E_{0}(\theta ,\phi )=E_{0,∥}{\overset{ˆ}{e}}_{∥}+E_{0,⊥}{\overset{ˆ}{e}}_{⊥}$ of a fluorescent emitter (point emitter); see Eq. 59. We do so by noting that the electromagnetic emission of basically all fluorescent emitters (organic dyes, fluorescent proteins, luminescent nano-crystals also termed quantum dots), used in fluorescence microscopy are approximated by that of an oscillating electric dipole. Important exceptions include some emission bands of rare earth emitters such as europium complexes, which can show magnetic dipole or electric quadrupole properties [143, 144]. The framework that we will provide herein for the emission of an electric dipole emitter can be readily generalized to these exotic cases as well.

To find the electromagnetic field of such oscillating electric dipole, we start from Maxwell’s equations. We do so by considering these equations for a dipole moment with amplitude p and oscillation frequency ω located at $r_{d}=\left(x_{d},y_{d},z_{d}\right)$ in the sample medium with refractive index $n_{d}$. Moreover, here, we only consider the amplitudes of the electric and magnetic fields given that all fields have the same time dependence exp(-iωt) as the dipole moment oscillations. As such, Maxwell’s equations can be recast as

(68) $$\nabla \times E=\frac{i\omega }{c}B\;\nabla \times B=-\frac{i\omega \epsilon_{d}}{c}E+\frac{4\pi }{c}j,$$

where $\epsilon_{d}=n_{d}^{2}$ is the dielectric constant of the sample solution in which the dipole is embedded and $j=-i\omega p\delta \left(r-r_{d}\right)$ is the electric current generated by the oscillating dipole. Thus, we find the following equation for the electric field $E_{d}$ of the dipole emitter

(69) $$\nabla \times \nabla \times E_{d}-k_{d}^{2}E_{d}=4\pi k_{0}^{2}p\delta \left(r-r_{d}\right),$$

where $k_{0}=\omega /c$ and $k_{d}=n_{d}k_{0}$. Using $\nabla \times \nabla \times E_{d}=\nabla \nabla \cdot E_{d}-\nabla^{2}E_{d}$ [142] and passing to the Fourier space yields for the Fourier amplitude ${\tilde{E}}_{d}$

(70) $$\left(k^{\prime 2}-k_{d}^{2}\right){\tilde{E}}_{d}-k^{\prime }\left(k^{\prime }\cdot {\tilde{E}}_{d}\right)=4\pi k_{0}^{2}p\;exp\left(-ik^{\prime }\cdot r_{d}\right),$$

where $k^{\prime }$ is the Fourier space coordinate. Multiplying the last equation by $k^{\prime }$ yields

(71) $$k^{\prime }\cdot {\tilde{E}}_{d}=-\frac{4\pi }{\epsilon_{d}}k^{\prime }\cdot p\exp\left(-ik^{\prime }\cdot r_{d}\right).$$

Now, by replacing the above equation into Eq. 70, we arrive at

(72) $${\tilde{E}}_{d}=\frac{4\pi \;exp\left(-ik^{\prime }\cdot r_{d}\right)}{\epsilon_{d}\left(k^{\prime 2}-k_{d}^{2}\right)}\left[k_{d}^{2}p-k^{\prime }\left(k^{\prime }\cdot p\right)\right].$$

In real space, the above reads

(73) $$E_{d}=\int \frac{d^{3}k^{\prime }}{2\pi^{2}\epsilon_{d}}\left[k_{d}^{2}p-k^{\prime }\left(k^{\prime }\cdot p\right)\right]\frac{exp\left[ik^{\prime }\cdot \left(r-r_{d}\right)\right]}{k^{\prime 2}-k_{d}^{2}},$$

where the absolute value $\left|r-r_{d}\right|$ is the distance between electric dipole’s location, $r_{d}$, and the observation point, r.

**FIG. 15:**
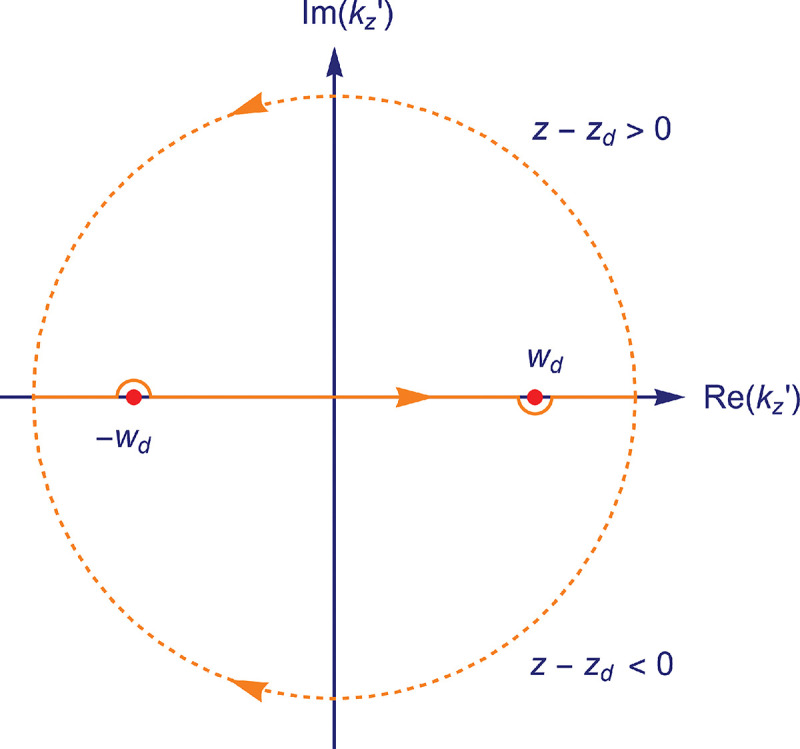
Contour for the integration over $k_{z}^{\prime }$ of Eq. 73 in the complex $k_{z}^{\prime }$-plane. For positive values of $z-z_{d}$, the contour has to be closed, at infinity, over the positive $Im\left(k_{z}^{\prime }\right)$ half-space, while for negative values of $z-z_{d}$ it is over the negative half-space. Along the real axis, the integrand has two poles at $\pm w_{d}=\pm \sqrt{k_{d}^{2}-q^{2}}$.

To obtain an expression well suited in modeling the emission of a dipole in a planar system (*e.g.*, above a flat coverslide), we perform the integration along the $k_{z}^{\prime }$-coordinate in the above expression, using Cauchy’s residue theorem. To do so, we close the integration path along the real axis and complete a semi-circle at infinity over the complex $k_{z}^{\prime }$-plane, as shown in Fig. 15. To make sure that the exponent vanishes when extending the contour into the complex plane, one has to close the contour over the positive imaginary half plane when $z-z_{d}>0$ and over the negative imaginary half plane when $z-z_{d}<0$. Along the real axis, the integrand has two poles at positions $\pm w_{d}=\pm \sqrt{k_{d}^{2}-q^{2}}$, where $q^{2}=k_{x}^{\prime 2}+k_{y}^{\prime 2}$. However, the result of the integration must contain only *outgoing* plane wave contributions (Sommerfeld radiation condition [145]), achieved by deforming the integration contour around the two poles as shown in Fig. 15. Subsequently applying Cauchy’s residue theorem yields the result

(74) $$E_{d}=\frac{i}{2\pi \epsilon_{d}}\int \frac{d^{2}q}{w_{d}}\left[k_{d}^{2}p-k_{d}\left(k_{d}\cdot p\right)\right]\;\;exp\left[iq\cdot \left(\rho -\rho_{d}\right)+iw_{d}\left|z-z_{d}\right|\right],$$

where we used the abbreviations $k_{d}=\left(q,w_{d}\right)$ and $w_{d}=\sqrt{k_{d}^{2}-q^{2}}$ is pole location. The two-dimensional integration over q extends over an infinite (Fourier) plane oriented perpendicular to the optical axis. The z-component $w_{d}$ of $k_{d}$ is assumed to attain either zero or a positive imaginary part, because only solutions that will not diverge towards infinity for large distances $\left|z-z_{d}\right|$ are physically admissible. Eq. 74 is the plane wave representation of the electric field of a free oscillating dipole, also called the Weyl representation (see *e.g.*, Refs. [146, 147]). As we will see, the Weyl representation is particularly suited for modeling the imaging of an emitter through a microscope.

**FIG. 16:**
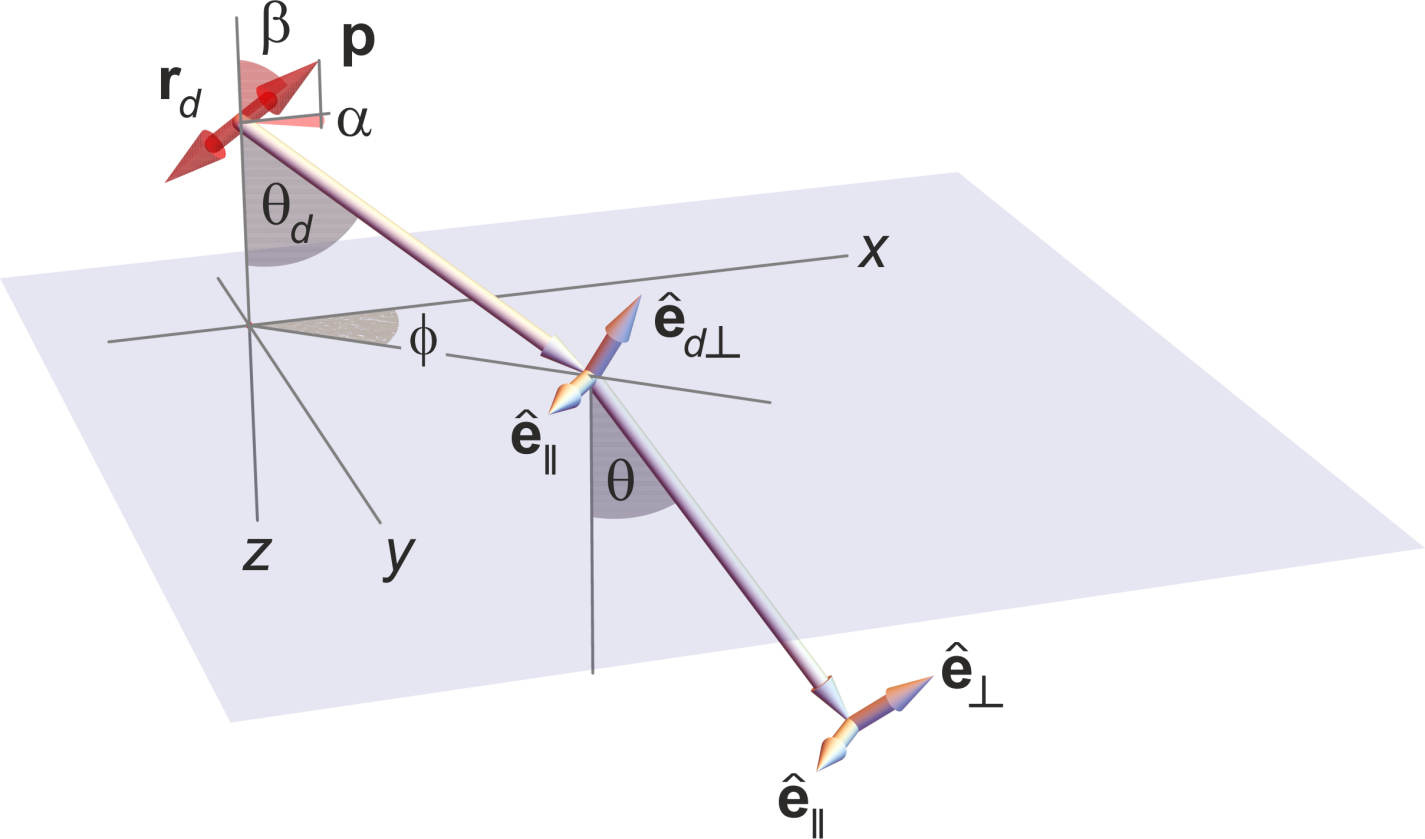
Geometry for deriving the angular distribution of electric field generated by a single dipole emitter. Here, the gray rectangle represents the coverslide which is the interface between the electric dipole’s embedding medium (above the coverslide) and the immersion medium below the coverslide. α and β are, respectively, polar and inclination (azimuthal) angles describing the orientation of the dipole. ϕ is the polar angle of the wave vector. $\theta_{d}$ and θ are the azimuthal angles of the wave vector above and below the interface. It is also common to assume that coverslide coincides with z=0.

Next, we consider the situation that the refractive index of the medium $n_{d}$ where the emitting molecule is situated and the refractive index n of the immersion medium of the microscope’s objective are different (*e.g.*, imaging with an oil immersion objective with an emitter in water). This situation is schematically shown in Fig. 16. To model the propagation of the electric field Eq. 74 through an interface dividing the sample and immersion media, *e.g.*, coverslide surface, it is convenient to split the electric field into its p- and s-polarized components

(75) $$k_{d}^{2}p-k_{d}\left(k_{d}\cdot p\right)=k_{d}^{2}\left[\left(p\cdot {\overset{ˆ}{e}}_{∥}\right){\overset{ˆ}{e}}_{∥}+\left(p\cdot {\overset{ˆ}{e}}_{d⊥}\right){\overset{ˆ}{e}}_{d⊥}\right]$$

where the unit vectors ${\overset{ˆ}{e}}_{∥},\;{\overset{ˆ}{e}}_{d⊥}$ and ${\overset{ˆ}{k}}_{d}$ form an orthogonal set similar to Eq. 48 by ${\overset{ˆ}{e}}_{∥}=(-sin\;\phi ,cos\;\phi ,0)$ and ${\overset{ˆ}{e}}_{d⊥}={\overset{ˆ}{e}}_{∥}\times {\overset{ˆ}{k}}_{d}$, see also Fig. 16. As such, the problem reduces to considering the propagation of s- and p-polarized plane waves through a planar interface.

We now use Eqs. 74–75 to write the electric field after it crosses the interface between the two media and travels a distance before reaching in front of the objective lens (immersion medium with refractive index n) in term of the p– and s-polarized components

(76) $$E_{d}=\frac{ik_{0}^{2}}{2\pi }\int \frac{d^{2}q}{w}\left[t_{∥}\left(p\cdot {\overset{ˆ}{e}}_{∥}\right){\overset{ˆ}{e}}_{∥}+t_{⊥}\left(p\cdot {\overset{ˆ}{e}}_{⊥}\right){\overset{ˆ}{e}}_{⊥}\right]\;\;exp\left[iq\cdot \left(\rho -\rho_{d}\right)-iw_{d}z_{d}+iw(z-f)\right],$$

where the $t_{∥,⊥}$ are the q-dependent Fresnel transmission coefficients, and the axial component w of the wave vector k is given by $w=\sqrt{k^{2}-q^{2}}=\sqrt{n^{2}k_{0}^{2}-q^{2}}$. Here, w refers to the immersion medium (*e.g.*, oil), and $w_{d}$ refers to the molecule’s embedding medium (*e.g.*, water/cytosol). Thus, the unit vector ${\overset{ˆ}{e}}_{⊥}$ is similar to ${\overset{ˆ}{e}}_{d⊥}$ but formed from the wave vector $\left(q,\sqrt{n^{2}k_{0}^{2}-q^{2}}\right)$ instead of $\left(q,\sqrt{n_{d}^{2}k_{0}^{2}-q^{2}}\right)$. Moreover, here, the focal distance, f, is the location of the focal plane with respect to the interface z=0 coinciding with the coverslide surface separating the sample from the immersion medium; see Fig. 16.

Indeed, the formulation above can be readily generalized to arbitrary numbers of interfaces. For instance, if an emitter is imaged through a stack of several layers characterized by different refractive indices, then the Fresnel transmission coefficients for a single interface in the above equation must simply be replaced by those for the stacked structure.

**FIG. 17:**
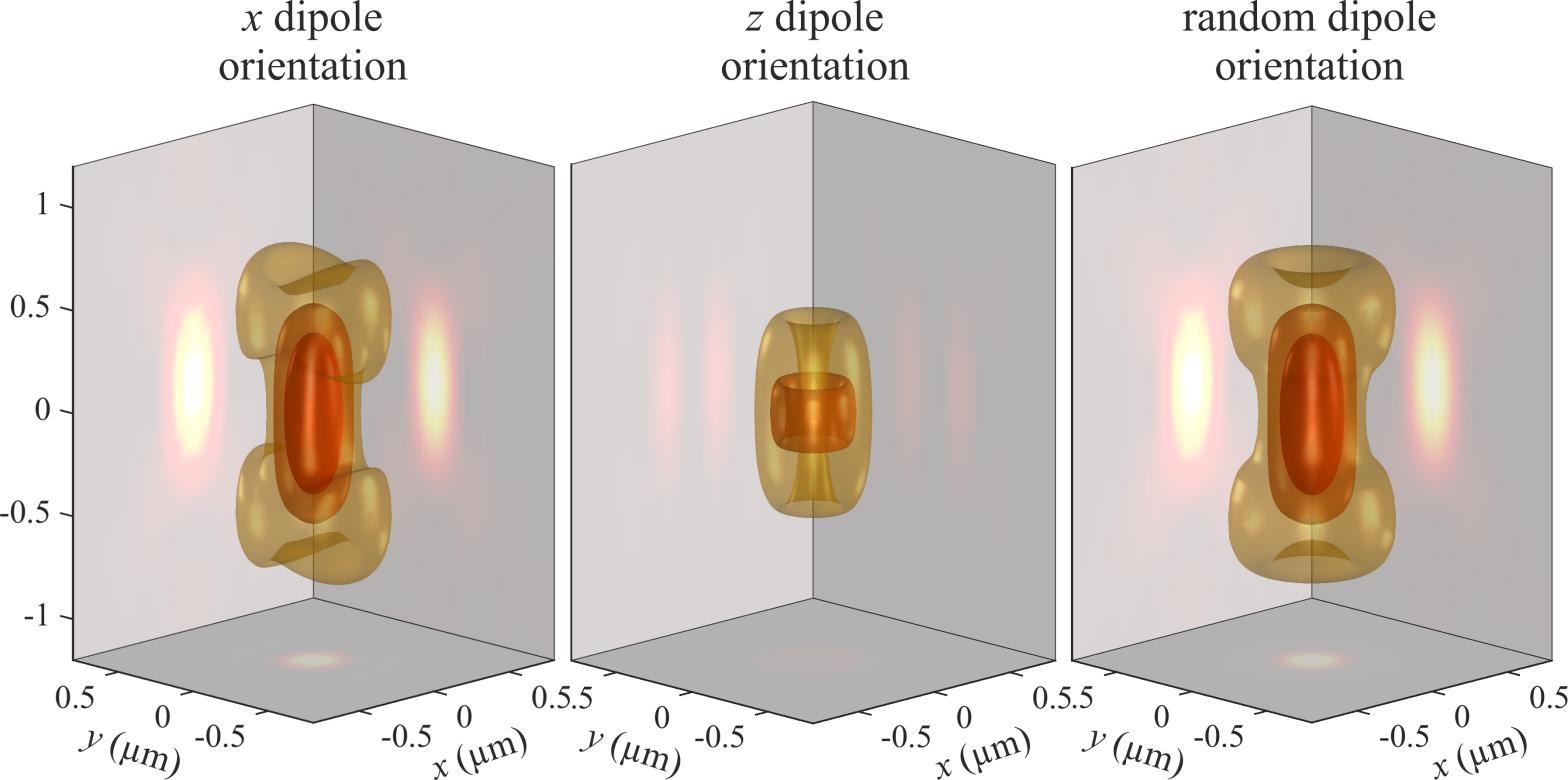
PSF of a wide-field microscope, projected into sample space. Shown are plots of the $1/e,\;1/e^{2}$ and $1/e^{3}$ iso-surfaces of the maximum PSF value. The lateral coordinates refer to back-projected sample space coordinates $(x,y)=\left(x^{\prime },y^{\prime }\right)/ℳ$, whereas the axial coordinate refers to an emitter’s axial position $z_{d}$. We will use this kind of representation of the PSF for all 3D PSF figures in this review. Left panel shows the PSF for an electric dipole emitter with fixed orientation along the *x*-axis, middle panel shows the PSF for a fixed orientation along the *z*-axis (optical axis), and right panels shows the PSF for a rapidly rotating emitter (isotropic emitter, superposition of PSFs for dipoles with x,y and z orientations.) Calculations were done for a water immersion objective with NA=1.2, and a refractive index of water n=1.33. Emission wavelength was set to λ=550nm.

Finally, following the scenario illustrated in Fig. 16. we have $q=nk_{0}(sin\;\theta \;cos\;\phi ,sin\;\theta \;sin\;\phi ,0)$ and $w=nk_{0}cos\;\;\theta \;$ which, in turn, leads to $d^{2}q/w=dq_{x}dq_{y}/w=nk_{0}sin\;\theta \;d\;\theta \;d\phi$ in spherical coordinates. Substituting this result into Eq. 76 and comparing with Eq. 46 shows that the electric field amplitude $E_{0}(\;\theta \;,\phi )$ for a dipole emitter is given by (up to some constant factor)

(77) $$\begin{array}{c} E_{0}\propto \left[t_{∥}\left(p\cdot {\overset{ˆ}{e}}_{∥}\right){\overset{ˆ}{e}}_{∥}+t_{⊥}\left(p\cdot {\overset{ˆ}{e}}_{⊥}\right){\overset{ˆ}{e}}_{⊥}\right]\\ exp\left[-iq\cdot \rho_{d}-iw_{d}z_{d}-iwf\right], \end{array}$$

or more explicitly

(78) $$\left(\begin{array}{c} E_{0,∥}\\ E_{0,⊥} \end{array}\right)=\left(\begin{array}{c} E_{0}\cdot e_{∥}\\ E_{0}\cdot e_{⊥} \end{array}\right)\;\;\propto |p|exp\left(-iq\cdot \rho_{d}-iw_{d}z_{d}-iwf\right)\;\;\left(\begin{array}{c} -t_{∥}sin\;\beta \;sin(\phi -\alpha )\\ t_{⊥}[sin\;\beta \;cos\;\theta \;cos(\phi -\alpha )-cos\;\beta \;sin\;\theta ] \end{array}\right),$$

where α and β are the dipole orientation angles as described in Fig. 16. By inserting these expressions into Eqs. 56, 57 and 61, one can compute the wide-field image PSF of the dipole emitter with arbitrary position and orientation. When doing this, it is convenient to present the result in terms of the lateral sample coordinates $\rho =\rho^{\prime }/ℳ$ instead of the image space coordinates $\rho^{\prime }$, and as a function of the axial position $z_{d}$ (with respect to the coverslide) of the emitter. This notation will be applied to all PSF visualizations throughout this review. Thus, in what follows, when writing the PSF, U(r), as a function of r, it is silently assumed that the lateral coordinates x and y are the coordinates conjugate to $x^{\prime }$ and $y^{\prime }$, *i.e.*, $x=x^{\prime }/ℳ$ and $y=y^{\prime }/ℳ$, and z refers to the axial position $z_{d}$ of the emitter. As a first example, Fig. 17 shows three-dimensional representations of the PSF of a dipole emitter on the optical axis, from left to right, the first panel for an emitter with fixed dipole orientation along the *x*-axis, the second panel for orientation along the optical axis (z-axis), and the last panel for a rapidly rotating emitter, where the isotropic PSF, $U_{\text{iso}}(r)$, is given by an average of the three PSFs calculated for dipole orientations along the x,y and z axes [148]

(79) $$U_{\text{iso}}\left(r\right)=\frac{1}{3}\left[U_{x}\left(r\right)+U_{y}\left(r\right)+U_{z}\left(r\right)\right].$$

The last PSF is the most relevant case in the majority of fluorescence microscopy applications, fluorescent labels are coupled to structures of interest with a sufficiently flexible linker allowing labels to nearly freely rotate in space.

A topic of significant importance for SMLM (Sec. VB2) relates to the effect of emitter dipole orientation on the wide-field image of the emitter, because fixed orientations with an orientation that is neither parallel to the sample surface nor to the optical axis can lead to systematic mislocalization of emitters’ true positions [149–152].

**FIG. 18:**
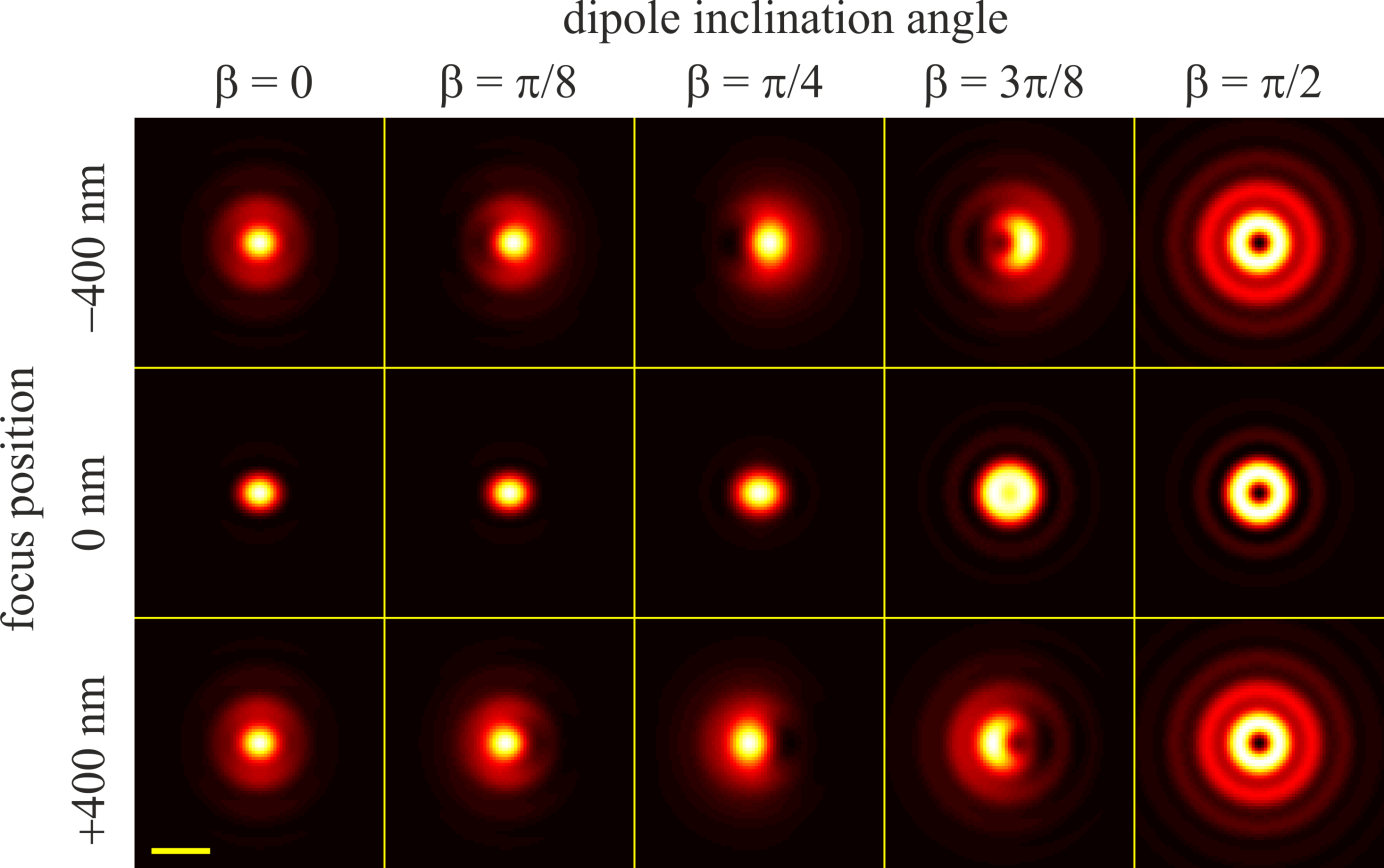
Effect of orientation on molecule image. Top row: Model calculations of images of electric dipole emitters with fixed but different orientations in the *xz*-plane, where the dipole inclination angle β is the angle between the dipole axis and the optical axis. The emitter is situated 400 nm below the focal plane of the water immersion microscope with an 1.2 NA objective. Middle row: same as top row, but for emitter situated in the focal plane of the microscope. Bottom row: Same as top and middle row, but for an emitter situated 400 nm above the focal plane. Yellow scale bar is 0.5μm.

As an example, Fig. 18 shows images of single emitters with different inclination angles towards the optical axis and different axial positions. As can be seen, for out-of-focus emitters intermediate values of the inclination angle β can lead to considerable shifts of the center of mass of an emitter’s image, namely the PSF. The figure presents results for imaging with a water immersion objective with NA=1.2, where such orientation related shifts in the center of mass of an emitter’s image becomes significant only if the emitter is not in the focal plane. The situation only worsens when working with oil-immersion objectives with a larger Total Internal Reflection (TIR) critical angle than water immersion objective, which allows collection of fluorescent light with larger incident angles. In this case, even for in-focus positions, an emitter’s image will dramatically depend on its orientation. However, although this effect can hinder localizations of rigid single molecules, it can be exploited to determine the three-dimensional orientation of these molecules [114, 153–155].

**FIG. 19:**
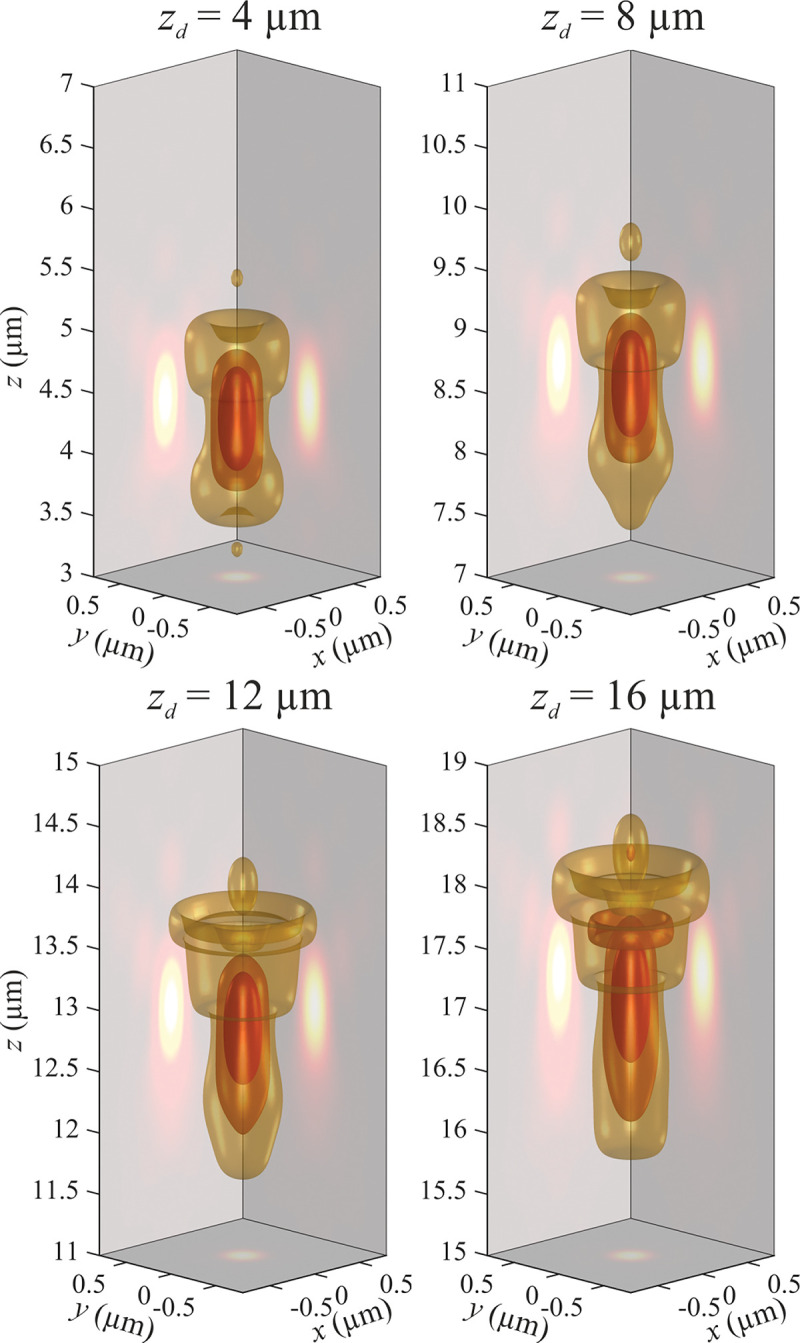
Effect of refractive index mismatch on the PSF. Model calculations of the PSF of a rapidly rotating electric dipole emitter (isotropic emitter) positioned at different distances from a cover slide surface as indicated above each PSF image. Calculations were done for a 1.2 NA water immersion objective corrected for an immersion/sample medium with refractive index n=1.33, while the solution above the cover slide is assumed to have a refractive index value of n=1.38 (*i.e.*, refractive index mismatch Δn=0.05). The bottom of each box shows a density plot of the PSF’s cross-section through its maximum value.

Finally, we briefly consider here the impact of refractive index mismatch. This effect leads to optical aberrations resulting in PSF distortion; see Sec. III F. As an example, Fig. 19 depicts this effect for a slight refractive index mismatch of only Δn=0.05 on the PSF, again for a water immersion objective with NA=1.2. As can be seen, this mismatch primarily results in PSF axial stretching and an axial shift between its center position towards larger z-values with respect to the actual position of the emitter. However, the lateral PSF crosssection at the axial location of its maximum does not change significantly, meaning that the refractive index mismatch will not affect the lateral position of the focused image of an emitter, but will lead to its mislocalization along the optical axis, *i.e.*, axial direction.

### E. Scalar approximation of PSF

In the previous section, we derived the exact electric field of an emitter, *i.e.*, oscillating dipole, (see Eq. 78) and used it to compute the PSF. However, these exact expressions are difficult to computationally manipulate. As such, in this section, we provide a simple approximation to the emitter’s electric field and the resulting approximate PSF.

Along these lines, for many practical applications, we can assume an isotropic emitter, *i.e.*, one with uniform emission amplitude in all directions. In such case, we can ignore the vectorial nature of the electric (and magnetic) fields resulting in an approximate scalar model. To derive such scalar approximation, we start from Eq. 56 and replace the amplitude vector $E_{0,∥}{\overset{ˆ}{e}}_{∥}+E_{0,⊥}{\overset{ˆ}{e}}_{⊥}^{\prime }$ by a scalar constant. Therefore the expression for the now “scalar” electric (magnetic) field in the image plane generated by an isotropic emitter on the optical axis at position $z=z_{d}$ simplifies to (up to a constant factor)

(80) $$E(r)\;\propto \int_{0}^{\Theta^{\prime }}\;d\theta^{\prime }sin\;\theta^{\prime }\sqrt{\frac{cos\;\theta^{\prime }}{cos\;\theta }}\int_{0}^{2\pi }\;d\phi e^{iq^{\prime }\cdot \rho^{\prime }-ik^{\prime }cos\;\theta^{\prime }z^{\prime }}\;\;\propto \int_{0}^{\Theta^{\prime }}\;d\theta^{\prime }sin\;\theta^{\prime }\sqrt{\frac{cos\;\theta^{\prime }}{cos\;\theta }}\int_{0}^{2\pi }\;d\phi e^{i|q|\rho \;cos\;\phi -ik\;cos\;\theta z}\;\;\propto \int_{0}^{\Theta }\;d(sin\;\theta )sin\;\theta \frac{J_{0}(ksin\;\theta \rho )}{\sqrt{cos\;\theta^{\prime }cos\;\theta }}e^{-ik\;cos\;\theta z},$$

where, in the first step, we have used $q^{\prime }\cdot \rho^{\prime }=q\cdot \rho =$ |q|ρcosϕ due to $\rho^{\prime }=ℳ\rho$ and Abbe’s sine condition $\left(sin\;\theta^{\prime }=(n/ℳ)sin\;\theta \right)$, while remembering $\left|q^{\prime }\right|=k_{0}sin\;\theta^{\prime }$ and $|q|=k\;sin\;\theta =nk_{0}\;sin\;\theta$. Moreover, we have similarly used $k_{0}cos\;\theta^{\prime }z^{\prime }=k\;cos\;\theta z$ due to the Herschel condition and $z^{\prime }=ℳz$. In the second step, we performed the integral with respect to ϕ and used the Abbe’s sine condition in conjunction with its differential form (see Eq. 55) and ignored all the prefactors of n and ℳ. Here, $J_{0}$ is the Bessel function of the first kind of order zero [156].

The above expression can be further simplified by neglecting the square root factor approximating unity for small values of $\theta^{\prime }$ and θ (far-field limit). Eq. 80 therefore simplifies to

(81) $$E\left(\rho ,z\right)\approx {\left(\frac{n}{ℳ}\right)}^{2}\int_{0}^{\sin\Theta }d\eta \;\eta J_{0}(k\eta \rho )e^{-ik\sqrt{1-\eta^{2}}z},$$

where η=sinθ. For the special case of z=0 (emitter in the focal plane), this can be analytically integrated to yield

(82) $$E\left(\rho \right)\approx \frac{NA}{{ℳ}^{2}k_{0}\rho }J_{1}\left(NAk_{0}\rho \right),$$

where we have used $k\sin\Theta =NA\;k_{0}$. Here, $J_{1}$ is the Bessel function of the first kind of order one [156]. Finally, using this approximation, the PSF is given by the absolute square of the “scalar” electric field. Therefore, for the 2D PSF of an in-focus isotropic emitter in the far-field limit, we find the well-known Airy pattern

(83) $$U(\rho )\propto {\left[\frac{J_{1}\left(NAk_{0}\rho \right)}{k_{0}\rho }\right]}^{2}$$

where we have omitted a constant factor. It is important to recall that $k_{0}n\;sin\;\Theta =NAk_{0}$ is the maximum lateral wave vector component transmitted by the microscope from the sample to the image plane; see Sec. I C.

**FIG. 20:**
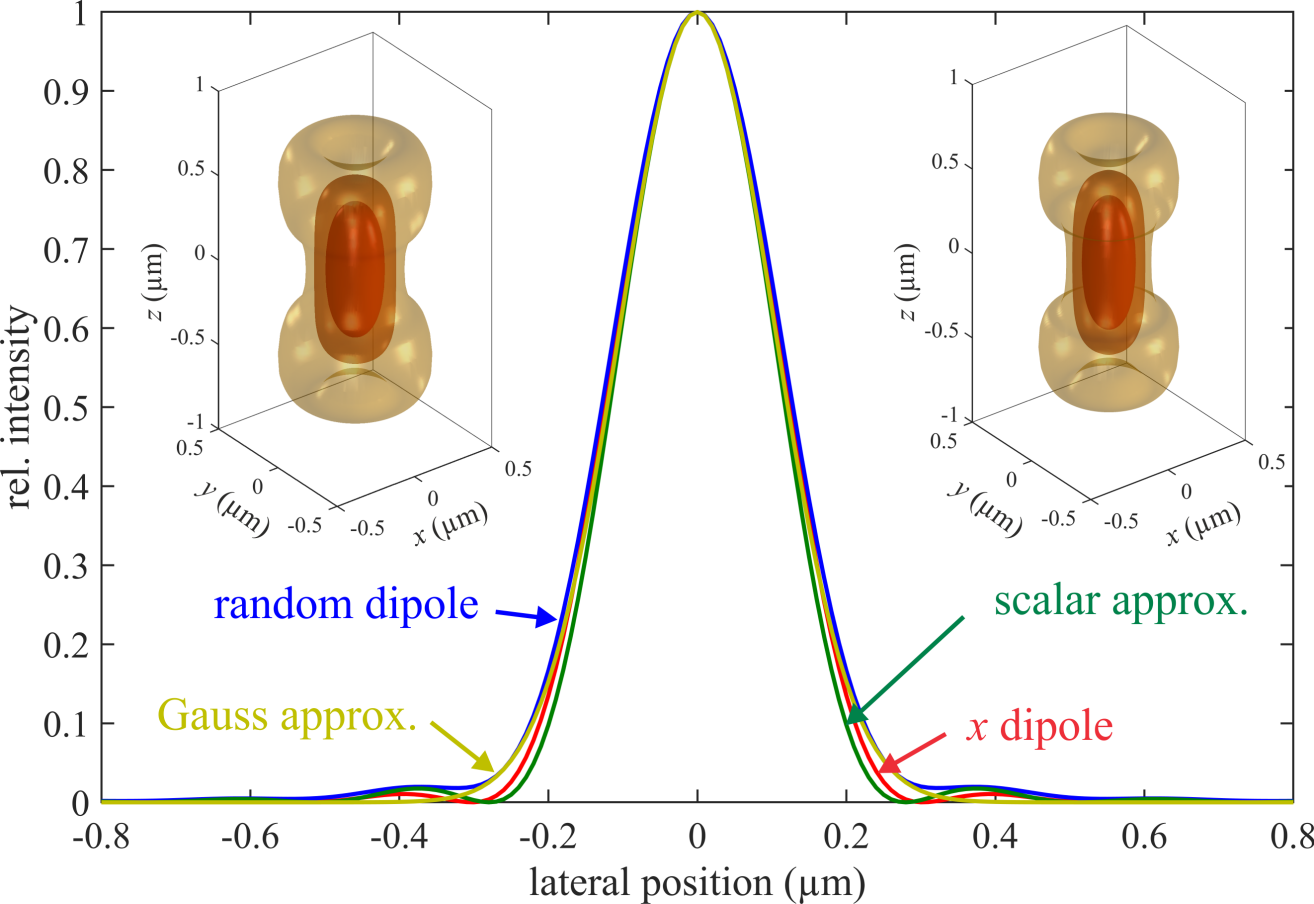
Comparison between scalar and vector calculations of the PSF. Shown are cross-sections of the PSF across the *x*-axis in the focal plane. The red curve shows results of the full wave–vector calculation for the PSF for an electric dipole emitter with fixed orientation along the *x*-axis, the blue curve the same calculation for a rapidly rotating (isotropic or random) emitter, the green curve presents the result of Eq. 83, and the ochre curve shows the Gaussian approximation of Eq. 86. Insets show two three-dimensional iso-surface plots of the PSF, left using the exact vector field calculation for an isotropic emitter, right for the scalar approximation. All calculations were performed for a water immersion objective with 1.2 NA.

In situations where the scalar approximation is suitable (for instance 3D imaging with molecules away from the coverslide interface exceeding a wavelength), this approximate PSF facilitates a computationally lighter model, as calculating Eq. 83 requires a single Fourier transform (integration) while evaluating Eq. 79 requires three Fourier transforms (integration) corresponding to three directions. To check the accuracy of this scalar approximation, Fig. 20 shows a comparison of the line cross-section of the PSF through its center, calculated with the full vectorial model of Eqs. 56, 57 and 61, and calculated in the scalar approximation of Eq. 83. As can be seen, the scalar approximation shows negligible deviations from the accurate model for the system considered (water immersion objective with NA=1.2, emission wavelength 500 nm). In most cases, this approximation is sufficient for quantitative analysis of fluoroscence microscopy data, *e.g.*, fitting single molecule images (see Sec. VB2) provided the molecules rapidly rotate.

**FIG. 21:**
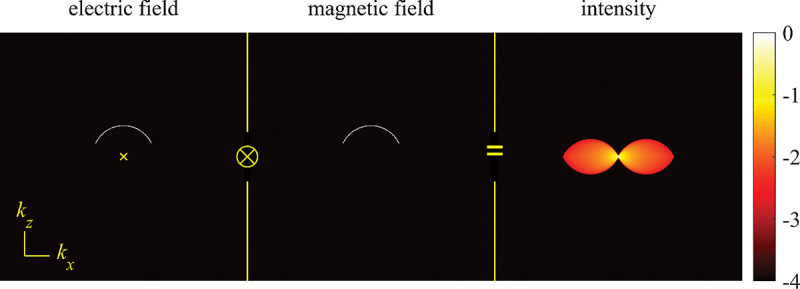
Scalar approximation of the OTF of a widefield microscope. Calculations were done for a 1.2 NA water immersion objective and an emission wavelength of 550 nm. The left panel shows the $k_{x}k_{z}$ cross-section of the electric field amplitudes in *sample space*, having a frequency support (frequencies with non-negative amplitude) in the shape of a spherical cap with radius k=2πn/λ and an opening half angle equal to the maximum half angle Θ of light collection of the objective. The middle panel shows the same distribution for the magnetic field. The right panel is a simple three-dimensional convolution of the left two distributions, yielding the scalar approximation of the OTF. All panels show density plots of the *decadic logarithm* of the absolute value of the Fourier amplitudes (see color bar on the right hand side) normalized by the maximum absolute value of the corresponding distribution. For all panels, the co-ordinate origin $\left(k_{x}=0,k_{z}=0\right)$ is at the center. It is important to emphasize that the frequency support of the OTF (*i.e.*, the frequencies where the OTF is non-zero) is the same when using the exact wave vector calculation or the scalar approximation. Thus, one can always use the scalar approximation of the OTF in determining the spatial resolution of a microscope (because this resolution depends only on the extent of the frequency support, but not the specific OTF values over this support). The length of the $k_{x}$ and $k_{y}$ bars in the lower left are 2πn/λ, where λ is the emission wavelength. Throughout this review, we will always use the same representation for all OTFs shown.

However, the usefulness of the scalar approximation is further evident in considering the OTF of a microscope. When comparing Eqs. 56 (also see Eq. 60) and 80, it is obvious that the *frequency support* of the Fourier transforms for the vector and scalar representations of the electric field are identical, given by a spherical cap centered at $k^{\prime }=0$ with radius 2π/λ and half opening angle $\Theta^{\prime }$. Similar to the visualization of the PSF, it is convenient to show the OTF back-projected to sample space, which is easily done using Abbe’s sine condition as $\left(k_{x}^{\prime },k_{y}^{\prime }\right)=n/ℳ\left(k_{x},k_{y}\right)$ and the relation $k^{\prime }=k/n$. Cross-sections of the corresponding electric (magnetic) field Fourier representation is shown in the left two panels of Fig. 21 at $k_{y}=0$. In the case of vectorial model, for each of the vector fields E and B, one will have two such cross-sections, one for the $E_{∥}\left(B_{∥}\right)$ and one for the $E_{⊥}\;\left(B_{⊥}\right)$ components. Here, Fig. 21 represents the scalar approximation with a uniform field amplitude over the whole spherical cap, *cf*. with Eq. 80. In both the exact vector field description as well as the scalar approximation, the PSF is found by products of the electric and magnetic field (components), which translates in Fourier space to a convolution of the corresponding Fourier representations of the electric and magnetic fields.

A cross-section of the OTF at $k_{y}=0$ is visualized in the right panel of Fig. 21, showing the (auto)convolution of the two Fourier amplitude distributions on the left. Importantly, although the exact amplitude distribution over the butterfly-shaped frequency support of the OTF will be slightly different for the full vector field (see Fig. 13 and Eq. 60) calculation and the scalar approximation (see Eq. 82), the frequency support of the OTF will be identical. This is particularly important to emphasize, because the limits of this frequency support determines the optical resolution of the microscope. Here, again, we emphasize that the resolution, along a given direction, is determined by the maximum frequency $k_{max}$ of this support along the chosen direction by Eq. 67. As such, for the wide-field microscope in Fig. 21, the lateral and axial extents of the frequency support of the OTF are $k_{max,y}=2nk_{0}sin\;\Theta$ and $k_{max,z}=nk_{0}(1-cos\Theta )$, respectively (also see Fig. 14). This leads to the values for the lateral and axial resolutions that we derived before (see Sec. I C, III C and Eq. 67)

(84) $$y_{min}=\frac{2\pi }{k_{max,y}}=\frac{\lambda }{2n\;sin\;\Theta }=\frac{\lambda }{2NA},$$

and

(85) $$z_{min}=\frac{2\pi }{k_{max,z}}=\frac{\lambda }{n(1-cos\;\Theta )}\approx \frac{2n\lambda }{{NA}^{2}}.$$

The first equation is Abbe’s famous lateral resolution limit for a wide-field microscope, while the approximate axial resolution in the second equation obtained is only valid for small numerical apertures.

We can further simplify the PSF by approximating Eq. 83 with a 2D Gaussian function

(86) $$U_{\text{gauss}}\left(\rho -\rho_{0}\right)\propto exp\left(-\frac{{\left|\rho -\rho_{0}\right|}^{2}}{2\sigma_{PSF}^{2}}\right),$$

where $\sigma_{PSF}=\sqrt{2}/\left(NAk_{0}\right)=\lambda /\sqrt{2}\pi n\;sin\;\Theta$, as can be found by requiring the same curvature values at the maximum for both Eq. 86 and Eq. 83; see also Fig. 20. This approximation is useful in creating a simple model, allowing straightforward fitting algorithms for many localization applications [152, 157]. This model fits the main lobe of the PSF and thus is a good approximation when imaging within the depth of focus in an aberration-free microscope. The width $\sigma_{\text{PSF}}$ is usually experimentally fit from a calibration sample or model [158].

### F. Optical aberrations

Finally, we discuss the impact of optical aberrations on the PSF. Optical aberrations refer to any deviation from idealized imaging models earlier presented and can be classified into various groups. The first distinction revolves around the wavelength, *i.e.*, monochromatic aberrations occurring for a single wavelength, by contrast to chromatic aberrations, originating from the chromatic dispersion of the components in the optical system. The second distinction is shift-invariance, *i.e.*, aberrations similar at every point in the Field Of View (FOV) *versus* off-axis aberrations. In the presence of optical aberrations, modeling the PSF as a two-dimensional Fourier transform, ${ℱ}_{2D}$, operation is common as then the aberrations can be treated as part of the system’s OTF. Here, we will focus on the scalar model, *i.e.*, Eq. 80. This approach can however be generalized to the vectorial case [159–161].

As, generally, optical aberrations can be a function of $\left(\phi ,\theta^{\prime }\right)$, we return to Eq. 80 and conveniently recast it as a ${ℱ}_{2D}$ operation prior to the integration over ϕ

(87) $$E\left(\rho ,z;r_{0}\right)=\int_{0}^{\Theta^{\prime }}\;d\theta^{\prime }sin\;\theta^{\prime }\sqrt{\frac{cos\;\theta^{\prime }}{cos\;\theta }}\int_{0}^{2\pi }\;d\phi e^{iq\cdot \rho -ik\;cos\;\theta z}\;\;\propto {ℱ}_{2D}\left(𝒜(\theta^{\prime },\phi )e^{i\left(\Psi \left(\theta^{\prime };z,f\right)+\Phi \left(\theta^{\prime },\phi \right)\right)}\right),$$

where $𝒜\left(\theta^{\prime },\phi \right)$ is the amplitude of the pupil function, which, neglecting all constant factors, simplifies to the Fourier plane support, limited by either the NA or $n_{d}$ as follows

(88) $$𝒜\left(\theta^{\prime },\phi \right)=\left\{\begin{array}{ll} 1, & \;\text{if}\;sin\;\theta^{\prime }\leq min\left(\frac{n_{d}}{n},\frac{NA}{n}\right)\\ 0, & \;\text{otherwise}\; \end{array},\right.$$

where n and $n_{d}$ are, respectively, the refractive index of the objective immersion and the dipole (emitter) medium. Although we set the non-zero part of the amplitude 𝒜 to 1, in full generality this term can be a function of $\theta^{\prime }$ and ϕ, for instance, in the presence of aberrations in the form of attenuation of the transmitted electric and magnetic fields. However, these types of aberrations are rare and often induce negligible changes to the PSF compared to the phase terms [162]. Therefore, it is safe to neglect the effect of amplitude and focus on the phase terms.

The first term in the phase, $\Psi \left(\theta^{\prime };z,f\right)$, is induced by the molecule’s shift off-axis and out-of-focus, *i.e.*, the term $-q\cdot \rho_{d}-w_{d}z_{d}-wfz$ in Eq. 78,

(89) $$\Psi \left(\theta^{\prime };z,f\right)=k_{0}zn_{d}\sqrt{1-sin\;\theta^{\prime 2}}\;\;-k_{0}fn\sqrt{1-{\left(\frac{n_{d}}{n}sin\;\theta^{\prime }\right)}^{2}}.$$

For instance, the phase $-k\sqrt{1-\eta^{2}}z$ in Eq. 81, where η=sinθ, is due to the out-of-focus location of the emitter. The second phase term in Eq. 87, $\Phi \left(\theta^{\prime },\phi \right)$, describes any additional phase terms of the pupil function (can be optical aberration as described in this section or PSF modulating elements described in Sec. VC), otherwise null in perfect aplanatic imaging conditions, as in Eq. 81.

Now, we proceed to describe the phase term due to different aberrations. First, we consider monochromatic shift-invariant aberrations, *i.e.*, not a function of (x,y). In this case, aberration terms can be readily added to Eq. 87 as a phase term $\Phi \left(\theta^{\prime },\phi \right)$. This phase function lives on the disk-like support ϕ∈{0,2π} and $\theta^{\prime }\in \left\{0,\Theta^{\prime }\right\}$ defined by the electric (magnetic) field Fourier amplitude distribution (see Sec. III B and Fig. 13). It is often convenient to expand phase aberrations into a system of orthogonal basis functions, namely Zernike polynomials $Z_{l}^{m}\left(\xi =\theta^{\prime }/\Theta^{\prime },\phi \right)$ (see *e.g.,* [163])

(90) $$\text{Φ}(\xi ,\phi )=\sum_{l}\sum_{m=-l}^{l}v_{lm}Z_{l}^{m}(\xi ,\phi )$$

where $v_{lm}$ are coefficients corresponding to $Z_{l}^{m}$. These polynomials are defined by

(91) $$Z_{l}^{m}(\xi ,\phi )=\left\{\begin{array}{ll} R_{l}^{m}(\xi )sin(m\phi ), & \;\text{if}\;m>0\\ R_{l}^{m}(\xi )cos(m\phi ), & \;\text{if}\;m\leq 0 \end{array}\right.$$

where the radial functions $R_{l}^{m}$ are given by

(92) $$R_{l}^{m}(\xi )=\sum_{k=0}^{(l-|m|)/2}\frac{{\left(-1\right)}^{k}(l-k)!\xi^{l-2k}}{k!\left[\frac{l+m}{2}-k\right]!\left[\frac{l-m}{2}-k\right]!}$$

if l-|m| is an even number, and zero otherwise.

**Table:** 

#	*l*	*m*	$Z_{n}^{m}$	name
1	1	−1	ξcosϕ	horizontal tilt
2	1	1	ξsinϕ	vertical tilt
3	2	0	$2\xi^{2}-1$	defocus
4	2	−2	$\xi^{2}\cos2\phi$	vertical astigmatism
5	2	2	$\xi^{2}\sin2\phi$	oblique astigmatism
6	3	−1	$\left(3\xi^{2}-2\right)\xi \cos\phi$	horizontal coma
7	3	1	$\left(3\xi^{2}-2\right)\xi \sin\phi$	vertical coma
8	4	0	$6\xi^{4}-6\xi^{2}+1)$	primary spherical
9	3	−3	$\xi^{3}\cos3\phi$	oblique trefoil
10	3	3	$\xi^{3}\sin3\phi$	vertical trefoil
11	4	−2	$\left(4\xi^{2}-3\right)\xi^{2}\cos2\phi$	vert. secondary astigmatism
12	4	2	$\left(4\xi^{2}-3\right)\xi^{2}\cos2\phi$	obl. secondary astigmatism

**FIG. 22:**
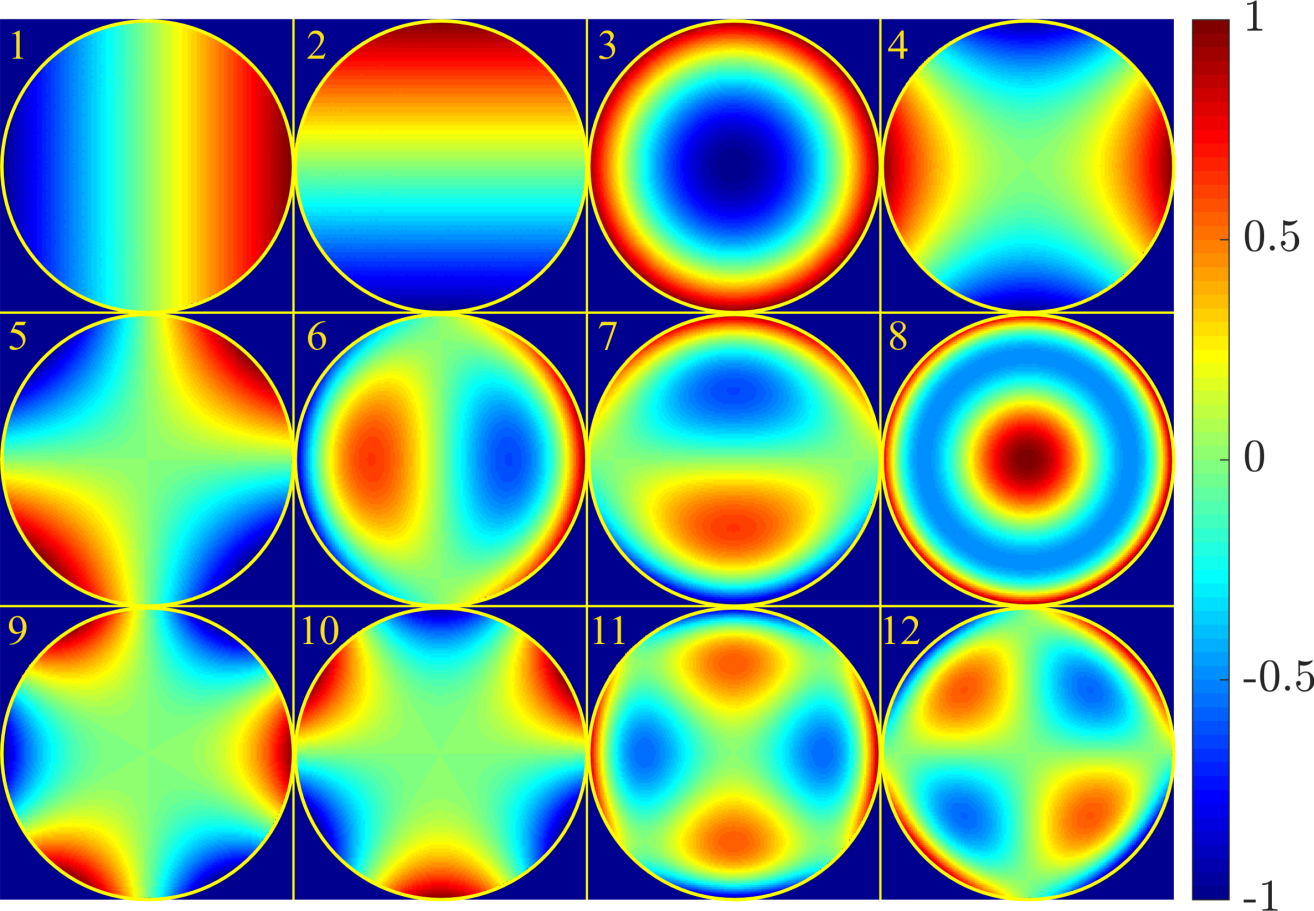
Density plots of the first twelve most important Zernike polynomials as presented in table IIIF. (1) horizontal or x tilt; (2) vertical or y tilt; (3) defocus; (4) vertical astigmatism; (5) oblique astigmatism; (6) horizontal coma; (7) vertical coma; (8) primary spherical aberration; (9) oblique trefoil; (10) vertical trefoil; (11) vertical secondary astigmatism; and (12) oblique secondary astigmatism.

**FIG. 23:**
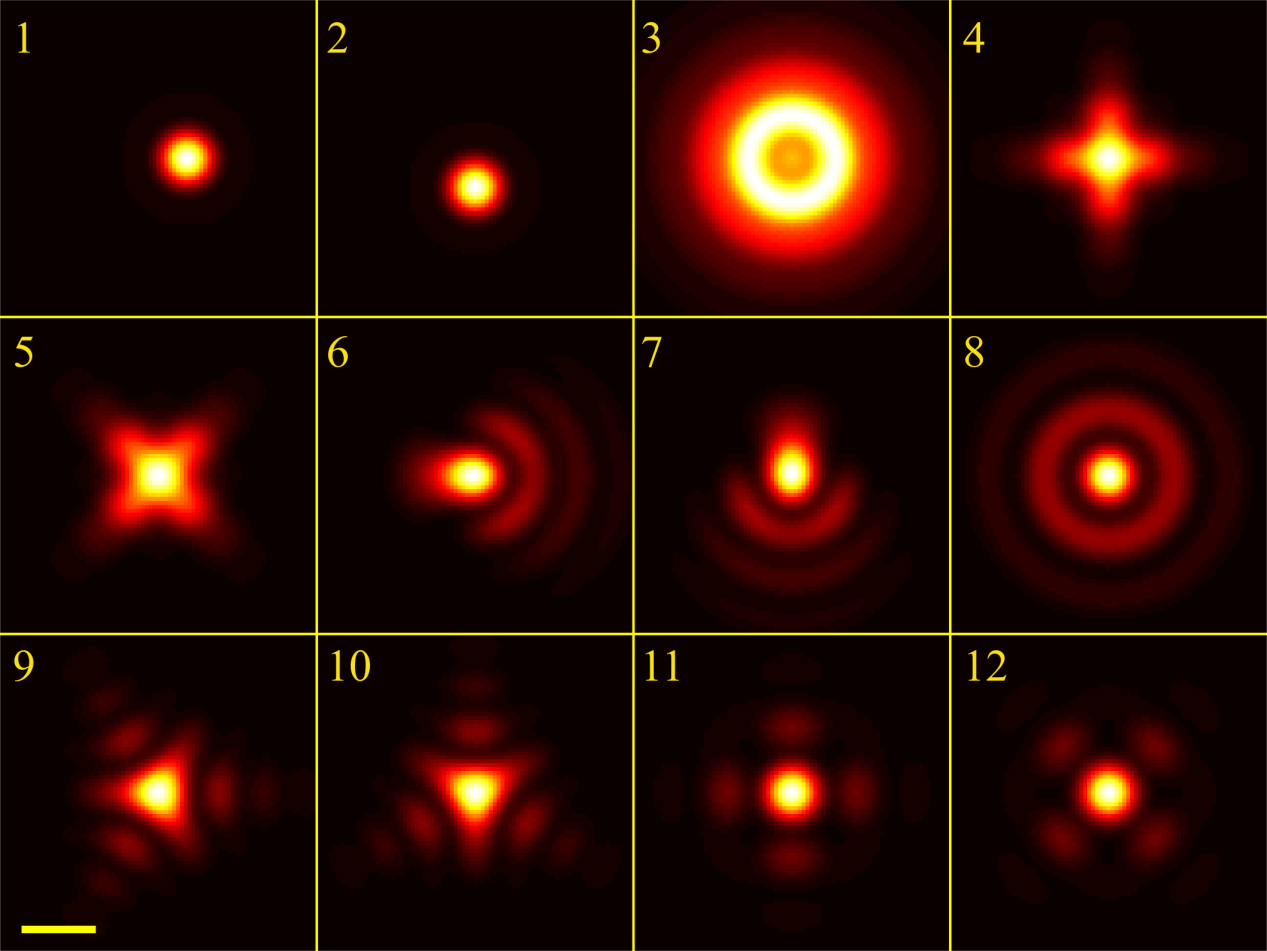
Model calculations of the image of an isotropic emitter (rapidly rotating dipole emitter) under the impact of aberrations defined by a phase function given by the Zernike polynomials shown in Fig. 22. To better visualize the effects of aberration, all Zernike polynomials were multiplied by a factor 2.5. Calculations were again done for a water immersion objective with NA 1.2 and for an emission wavelength of 550 nm. Yellow scale bar is 0.5μm.

Fig. 22 shows density plots of these first 12 Zernike polynomials, and Fig. 23 shows the impact of these aberrations on the PSF of a single isotropic emitter. The first three Zernike polynomials, namely horizontal title, vertical tilt and defocus, coincide with phases due to a lateral, vertical and axial shift in the emitter’s position, respectively. All the other terms describe PSF distortions due to optical aberrations.

In some cases, the aberration can be of higher dimension and not explained well by low order Zernike polynomials. For example, when using Liquid Crystal Spatial Light Modulators (LC-SLM) [164] or in some PSF engineering techniques [165], there might be a sudden phase step in the pupil function which cannot be expressed by a limited number of Zernike polynomials. Therefore, such cases require evaluating the aberration in a pixel-wise manner [159].

The second kind of aberration is chromatic shiftinvariant aberration. In microscopy, it is common to use achromatic objectives and lenses, however, those are never perfect, and furthermore, dispersion from various other components can induce PSF deviations. We have described the imaging model as the forward propagation of a monochromatic wavelength, however, all fluorescent molecules are not narrow-banded sources, having a typical emission spectrum S(λ) (describing the probability to emit at a wavelength λ) with a width of a few tens of nanometers; see Sec. II A. In such a case, the mean measured image will be a super-position integral over the molecule’s spectrum

(93) $$\Lambda \left(x,y;r_{0}\right)=\int_{\lambda }S(\lambda )U\left(x,y;r_{0},\lambda \right)d\lambda ,$$

where the wavelength dependent $U\left(x,y;r_{0},\lambda \right)$ is PSF per λ (as described in Sec. III E as a function of $k_{0}=2\pi /\lambda$). Such an aberration is detrimental in some PSF engineering scenarios, for example in multi-focus microscopy with a phase mask (more details in IV F) where custom chromatic correction gratings must be designed to correct the chromatic shifts in order to achieve a sharp split FOV image [166].

The most challenging aberrations are shift-variant, both chromatic and monochromatic, which cannot be simply described by the proposed model in Eq. 87, as the aberration is now a function of the lateral coordinates. In such a case, the aberration $\Phi \left(\theta^{\prime },\phi \right)$ in Eq. 87 becomes a function of position, *i.e.*, $\Phi \left(\theta^{\prime },\phi ,x,y\right)$. In microscopy, these kind of aberrations can occur either from the sample itself or from off-axis aberrations in the optical system, namely, systematic aberrations. Sample induced aberration occur when the sample structure has significant refractive index variations (*e.g.*, imaging in deep tissue). This issue usually requires the deployment of adaptive optics [167, 168]. On the other hand, offaxis aberrations caused by the optical system are easier to model as they tend to vary smoothly. These aberrations can be modeled as 2D polynomial coefficients over the FOV [169] (which multiply Zernike coefficients for example) or addressed by Nodal Aberration Theory [170].

## IV. FLUORESCENCE MICROSCOPY: MODALITIES

In the previous section, we described the fundamental optics of the wide-field microscope and derived its OTF and PSF. We also tied the lack of optical sectioning in wide-field microscopes to the missing cone of its OTF; see Fig. 13. In this section, we discuss different fluorescence microscopy modalities, *i.e.*, near-field microscopy; point scanning microscopy; SIM; light-sheet microscopy; and multi-plane microscopy, that achieve optical sectioning as well as higher resolutions. Further, we derive their corresponding OTFs and show that they accomplish optical sectioning as they collect more spatial frequencies along the axial direction by modifications to the illumination and/or detection arms.

### A. Near-field methods for enhanced axial resolution

As we saw in previous sections, in classical diffractionlimited microscopy, *i.e.*, wide-field microscope, there is a considerable gap between lateral and axial resolutions due to lack of optical sectioning, which typically differs by a factor of 3 to 5 (*cf.*, Figs. 3–4 and Sec. III C). In this section, we present fluorescence imaging methods using near-field (evanescent field) effects to improve *axial* resolution. Electromagnetic near-fields are non-propagating fields with strong intensity gradients far exceeding those seen in propagating waves. In what follows, we will illustrate microscopy techniques employing such gradients to localize objects with much higher resolution than what can be accomplished using propagating waves.

#### 1. Total internal reflection fluorescence microscopy

The first method to be discussed here leverages TIR occurring when a plane wave is incident on an interface separating two media with different refractive indices. We begin by Fresnel’s reflection and transmission coefficients $r_{⊥},\;r_{∥},\;t_{⊥}$, and $t_{∥}$ for s- and p-polarized plane waves reflected at an interface dividing a medium with refractive index $n_{1}$ (incidence medium) from a medium with refractive index $n_{2}$, which can be written in the following compact forms

(94) $$r_{⊥}\;=\frac{n_{⋆}^{2}-w_{⋆}}{n_{⋆}^{2}+w_{⋆}},r_{∥}\;=\frac{1-w_{⋆}}{1+w_{⋆}}\;t_{⊥}\;=\frac{2n_{⋆}}{n_{⋆}^{2}+w_{⋆}},t_{∥}\;=\frac{2}{1+w_{⋆}}$$

where we have used the abbreviations $n_{⋆}=n_{2}/n_{1}$ and $w_{⋆}=w_{2}/w_{1}=\sqrt{\left(n_{2}^{2}-q^{2}\right)/\left(n_{1}^{2}-q^{2}\right)}$ with $w_{1,2}$ understood as the axial components of the wave vector in the first and second media, respectively. Moreover, $q=2\pi n_{1}\;sin\;\theta_{\text{inc}}/\lambda$ is the length of the wave vector’s lateral component of the plane wave, with $\theta_{\text{inc}}$ being its incidence angle upon the interface with respect to the normal to the interface within the first medium. Here, it is convenient to work in a unit system where the length of the vacuum wave vector is unity. In this unit system, we have $q=n_{1}sin\;\theta_{\text{inc}}$.

Now, remembering that the electric field and wave vector are perpendicular, the electric field amplitude of the transmitted wave can be written as

(95) $$E_{⊥,∥}=E_{0}t_{⊥,∥}\left(-\frac{w_{2}\overset{ˆ}{q}+q\overset{ˆ}{z}}{n_{2}}\right)exp\left[iw_{2}z+iq\cdot \rho \right]$$

where $E_{0}$ is the amplitude of the incident field, with $\overset{ˆ}{q}$ and $\overset{ˆ}{z}$ unit vectors along the lateral wave vector component (parallel to the interface) and along the axial z direction (perpendicular to the interface), respectively.

As can be seen from definitions of $w_{⋆}$ after Eq. 94, for $q=n_{1}sin\;\theta_{\text{inc}\;}>n_{2}$, the axial component $w_{2}$ becomes purely imaginary and the *absolute* values of the reflection coefficients in Eq. 94 become both unity. This means one observes TIR of the incoming plane wave so that the full incident energy is reflected back into the first medium. TIR can only happen if $n_{1}>n_{2}$, and the critical incidence angle (TIR angle) where it starts is $\theta_{TIR}=arcsin\left(n_{2}/n_{1}\right)$. However, as can be seen from Eq. 95, the electric field in medium 2 does not instantly go to zero but decays exponentially with increasing distance z from the interface. This decaying field in the second medium is termed evanescent field or evanescent wave. The characteristic decay length $d_{\text{TIR}\;}$ of the electric field intensity can be directly derived from Eq. 95 and reads

(96) $$d_{TIR}=\frac{1}{2\left|w_{2}\right|}=\frac{1}{2\sqrt{n_{1}^{2}{sin}^{2}\;\theta_{inc}-n_{2}^{2}}}.$$

As such, although evanescent waves do not penetrate far within medium 2, they can still be used, for instance in TIRF microscopy, to excite fluorophores within a distance of $d_{\text{TIR}}$ from the surface [42]. Therefore, fluorophores deeper than $d_{\text{TIR}}$ within the sample (out-offocus fluorophores) are less likely to become excited decreasing the contribution of undesired out-of-focus fluorescent light.

**FIG. 24:**
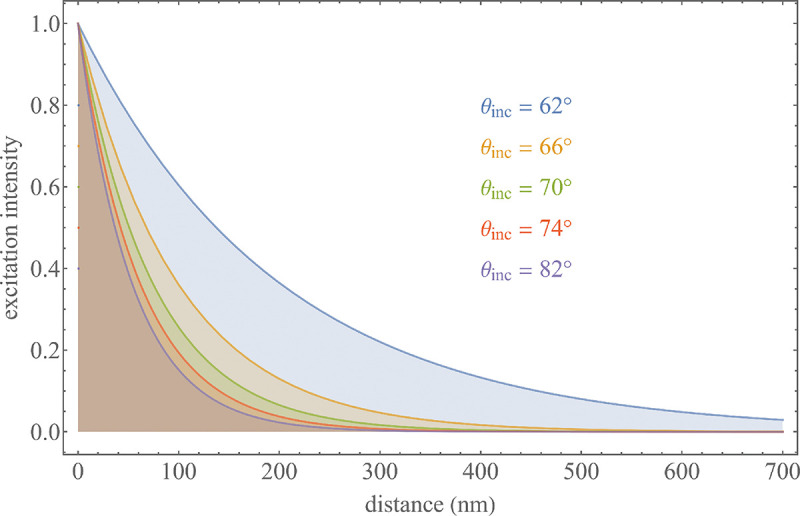
Total Internal Reflection Fluorescence (TIRF) microscopy. Dependence of excitation intensity above a cover slide as a function of incidence angle. The sample solution refractive index and that of the cover slide are assumed to be 1.33 (water) and 1.52, respectively, resulting in a TIR critical angle of $\approx 61^{{}^{\circ}}$. The excitation wavelength is taken as 470 nm.

The exponential decay of the excitation intensity can also be used for localizing fluorescent structures along the optical axis (perpendicular to the interface). This is realized by variable angle TIRF (vaTIRF) [171], where several images are recorded for different incidence angles of the excitation plane wave above the TIR angle. As can be seen in Fig. 24, for increasing incidence angles the decay of the excitation intensity becomes steeper. As such, emitter brightness values vary at different incidence angles which can be used to encode its distance from the interface by applying a deconvolution algorithm [172, 173] with an axial resolution down to a few nanometers, *i.e.*, by *ca.* 2–3 orders of magnitude better than the diffraction-limited resolution of a confocal microscope albeit close to the interface.

#### 2. Super-critical uorescence microscopy

While TIRF uses the evanescent fields generated by an incident excitation plane wave reflected at the glass-sample surface above the critical TIR angle, SAF microscopy employs the coupling of the near-field of a fluorophore’s emission into propagating modes in the coverslide’s glass [43, 44, 174–177]. To be precise, fields due to an oscillating electric dipole has components decaying as $1/r,\;1/r^{2}$ and $1/r^{3}$ where only the first term is a propagating term. The two other terms are non-propagating representing near-field emissions that decay within a short distance (≈λ). However, when the electric dipole is located close to a coverslide, the non-propagating near-field components of the dipole field are converted into propagating modes upon coupling into the glass which can be then collected and imaged by the microscope objective. These modes can be decomposed into a super-position of plane waves traveling along directions *above* the critical TIR angle for the given emission wavelength (super-critical angle fluorescence or SAF emission). The coupling of near-field modes of the fluorophore into propagating modes in the glass decrease with increasing distance from the interface. In contrast, the emission into the angle below the TIR angle (Under-critical Agle Fluorescence (UAF) emission) is due to the propagation of the emitter’s far-field emission into the glass and does not depend on its distance from the surface. Thus, at its core, SAF microscopy leverages the variation in SAF to estimate the distance of an emitter from the surface by measuring the ratio of its SAF to SAF+UAF emission intensity.

**FIG. 25:**
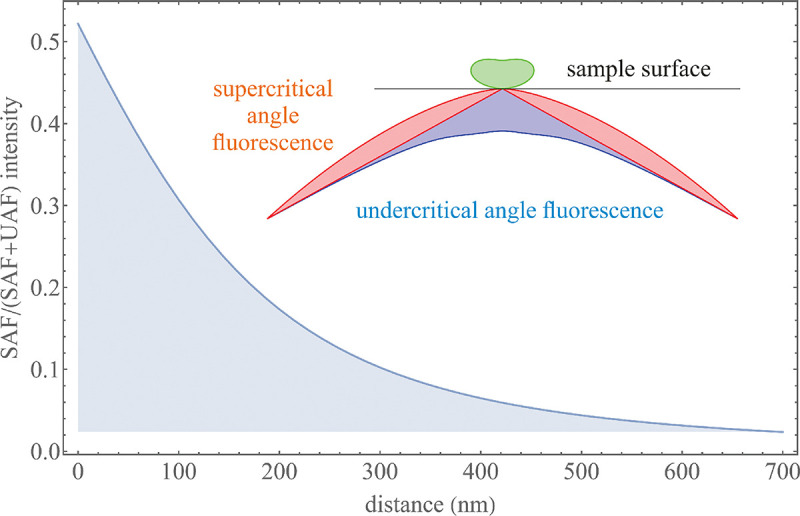
Super-critical Angle Fluorescence (SAF) microscopy. Ratio of super-critical to total downward fluorescence emission for a rapidly rotating molecule as a function of its distance from the surface. Sample solution refractive index and that of the cover slide glass are assumed to be 1.33 (water) and 1.52, respectively, with the emission wavelength taken as 550 nm. The inset shows the angular emission intensity distribution of an emitter directly on the surface (with the blue curve denoting UAF emission, the red curve denoting SAF emission, and the green curve denoting emission towards sample solution). The SAF emission strongly depends on the emitter’s distance to the surface, while the under-critical emission is independent of emitter axial position. By determining the ratio of SAF to SAF+UAF emission, the axial position of an emitter can be determined.

To quantitatively calculate the ratio of super-critical to under-critical angle emissions, we can use the theoretical framework developed in Sec. III. In particular, for calculating SAF emission intensity, one uses Eqs. 56 and 57, but with integration boundaries from $\theta^{\prime }=arcsin\left(n\theta_{TIR}/ℳ\right)$, dictated by the critical TIR angle, to $\theta^{\prime }=\Theta^{\prime }$, dictated by the numerical aperture, then calculates the energy flux density distribution using Eq. 61. The integral of the resulting energy flux density over the *xy*-plane is then proportional to the detectable SAF intensity. The calculation of the UAF intensity is completely analogous but with integration boundaries from $\theta^{\prime }=0$ to $\theta^{\prime }=arcsin\left(n\theta_{\text{TIR}}/ℳ\right)$. As an example, Fig. 25 shows the SAF to SAF+UAF ratio for a glass-water interface as a function of distance, assuming an isotropic emitter with emission wavelength of 550 nm. As can be seen, the dynamic range over which one can use this ratio for determining the emitter’s distance from the surface is very similar to the dynamic range over which vaTIRF is applicable, compare with Fig. 24.

#### 3. Metal-induced energy transfer imaging

**FIG. 26:**
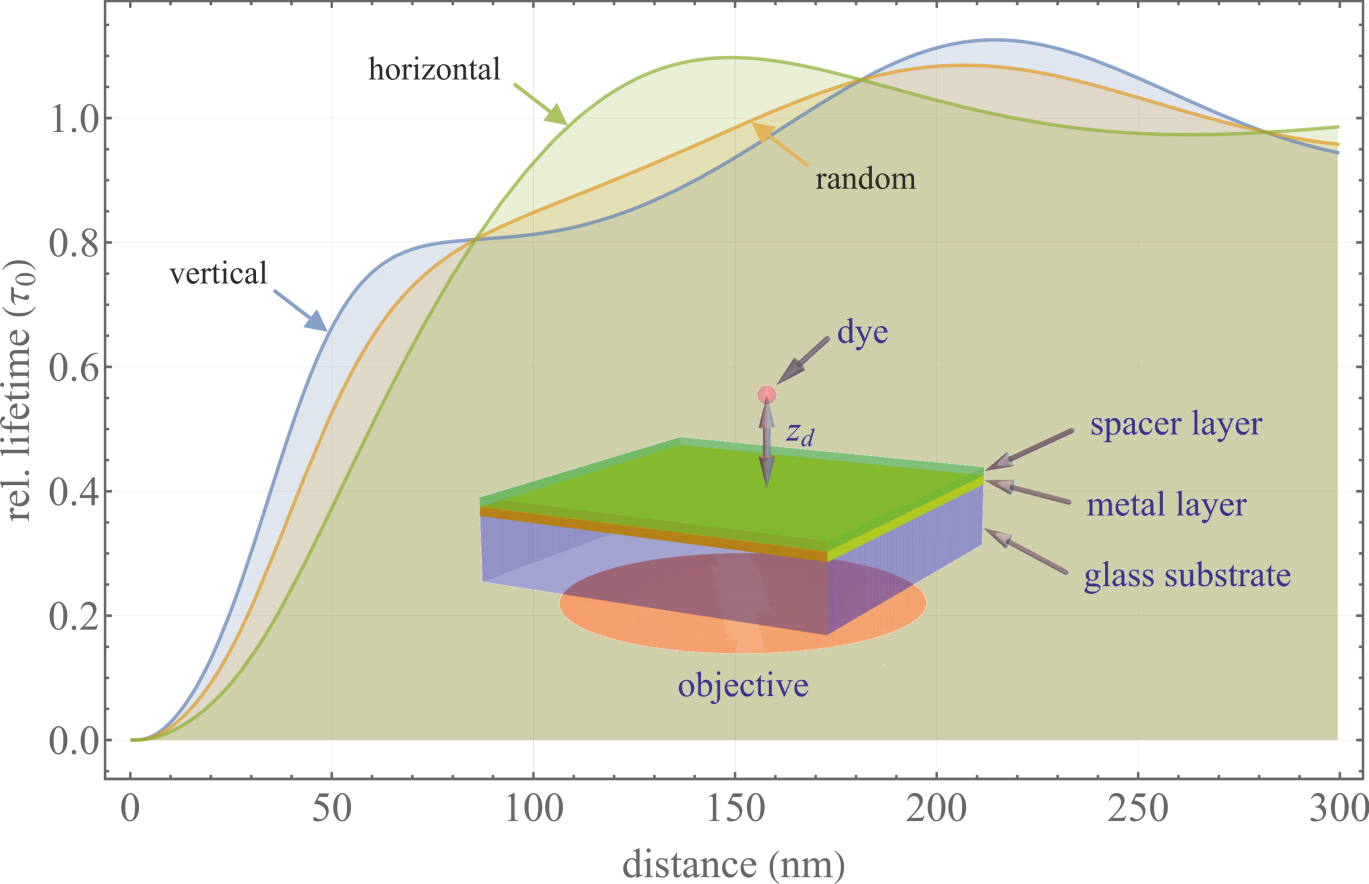
Metal-Induced Energy Transfer (MIET) microscopy: Dependence of the fluorescence lifetime (in terms of free space lifetime $\tau_{0}$) on the emitter’s distance from the glass substrate (cover slide) coated with a 20 nm gold layer. Calculations were done for an emission wavelength of 550 nm, and for a unit fluorescence quantum yield. Here we show the free curves for vertical, horizontal, and random emission dipole orientations. The inset shows MIET sample geometry.

Another near-field method used for axial localization in fluorescence microscopy is MIET imaging [45]. This method is based on near-field coupling similar to SAF microscopy. MIET uses the fact that when a fluorescent emitter (electric dipole emitter) comes close to a metal layer, its electric near-field can excite surface plasmons (coherent metal electron oscillations) in the metal, thereby accelerating de-excitation of fluorescent emitter’s excited state. This is observed as a strong decrease in fluorescence lifetime with decreasing distance from the surface. On the length scale of the dipole’s electric near-field, this leads to an overall monotonic dependence between lifetime and distance, as illustrated in Fig. 26, which can be used to infer distance.

Here, we use the theoretical framework developed in Sec. III to calculate the dependence of lifetime on distance. Briefly the lifetime depends on the emission power which requires the explicit calculation of both electric and magnetic fields.

**FIG. 27:**
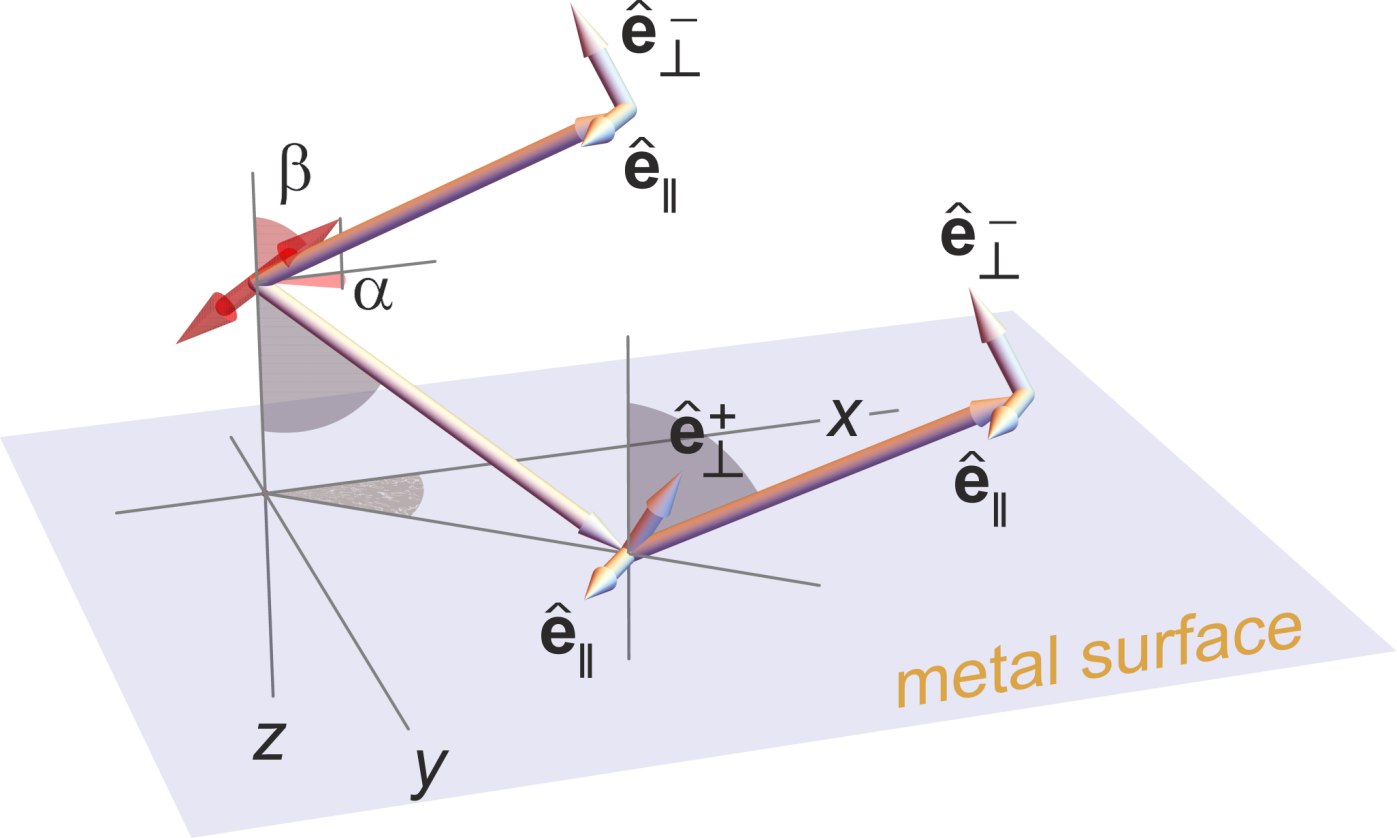
Geometry for deriving the electric field generated by a single dipole emitter above the MIET substrate (metal surface). The red double headed arrow shows a dipole located a distance $z_{d}$ above the metal surface with an orientation of β and α denoting polar and inclination (azimuthal) angles, respectively. The three longer single-headed arrows show plane wave component vectors, with corresponding perpendicular polarization unit vectors ${\overset{ˆ}{e}}_{∥}$ and ${\overset{ˆ}{e}}_{⊥}^{\pm }$. Here ${\overset{ˆ}{e}}_{⊥}^{+}$ is the unit vector associated with the wave vector moving toward the metal surface. Similar conventions hold for the other unit vectors.

Thus we start from the Weyl representation of the electric field of a free dipole emitter obtained in Eq. 74 to derive the electric field distribution above a MIET substrate (denoted by a metal surface in Fig. 27). As shown in Fig. 27, two sources contribute to the electric field above this metal surface: 1) direct emission from the dipole; and 2) emission reflected from the surface (*i.e.*, emission from the emitter’s image)

(97) $$E_{d}^{\pm }=\frac{ik_{0}^{2}}{2\pi }\int \frac{d^{2}q}{w_{d}}\left[\left(p\cdot {\overset{ˆ}{e}}_{∥}\right){\overset{ˆ}{e}}_{∥}\left(1+r_{∥}e^{iw_{d}\left|z+z_{d}\right|}\right)\right.\;\;\left.+\left(\left(p\cdot {\overset{ˆ}{e}}_{⊥}^{\pm }\right){\overset{ˆ}{e}}_{⊥}^{\pm }+\left(p\cdot {\overset{ˆ}{e}}_{⊥}^{+}\right){\overset{ˆ}{e}}_{⊥}^{-}r_{⊥}e^{iw_{d}\left|z+z_{d}\right|}\right)\right]\;\;exp\left[iq\cdot \left(\rho -\rho_{d}\right)+iw_{d}\left|z-z_{d}\right|\right],$$

where terms with the reflection coefficients $r_{∥,⊥}$ describe contribution from the reflected emission. Moreover, the superscripts “+” and “−” refer to plane waves moving towards and away from the metal surface. The $r_{⊥,∥}$ are Fresnel’s q -dependent reflection coefficients for p- and s-waves for the MIET substrate.

For planar structures of arbitrary complexity, these coefficients are readily obtained using propagation matrix formalism in Refs. [138, p. 254] and [178–180]. Here, we now have to distinguish between two p-wave polarization unit vectors: ${\overset{ˆ}{e}}_{⊥}^{+}$ for plane waves traveling towards the substrate, and ${\overset{ˆ}{e}}_{⊥}^{-}$ for plane waves traveling away from the substrate. The corresponding s-waves polarization unit vector ${\overset{ˆ}{e}}_{∥}$ is the same for both waves. We note that the result depends on the three-dimensional orientation of the emitter (given by the Euler angles α and β, see Fig. 27) via the scalar products $p\cdot {\overset{ˆ}{e}}_{⊥}^{\pm }$ and $p\cdot {\overset{ˆ}{e}}_{∥}$.

Analogously, we find a similar result for the magnetic field

(98) $$B_{d}^{\pm }=\frac{in_{d}k_{0}^{2}}{2\pi }\int \frac{d^{2}q}{w_{d}}\;\;\left[\left(p\cdot {\overset{ˆ}{e}}_{∥}\right)\left({\overset{ˆ}{e}}_{⊥}^{\pm }-{\overset{ˆ}{e}}_{⊥}^{+}r_{∥}e^{iw_{d}\left|z+z_{d}\right|}\right)\right.\;\;\left.+\left(\left(p\cdot {\overset{ˆ}{e}}_{⊥}^{\pm }\right)+\left(p\cdot {\overset{ˆ}{e}}_{⊥}^{+}\right)r_{⊥}e^{iw_{d}\left|z+z_{d}\right|}\right){\overset{ˆ}{e}}_{∥}\right]\;\;exp\left[iq\cdot \left(\rho -\rho_{d}\right)+iw_{d}\left|z-z_{d}\right|\right].$$

Now, given both electric and magnetic fields of Eqs. 97–98, the *total* emission power, designated by S(β), of the emitter can be obtained by integrating the outwards component of the Poynting vector over two planar interfaces sandwiching the emitter

(99) $$S(\beta )=\frac{n_{d}c}{8\pi }\int d^{2}\rho \;\;\overset{ˆ}{z}\cdot \left[{\left(E^{+}\times B^{+*}\right)}_{z=0}-{\left(E^{-}\times B^{-*}\right)}_{z<z_{d}}\right].$$

The emission power only depends on the polar orientation angle β of the dipole, but not its azimuthal angle α. The emission power S(β) can now be compared to the emission power $S_{0}$ of a “free” dipole within a homogeneous medium with refractive index $n_{d}$, given by the well-known formula in Ref. [142, p. 410] (which can also be obtained from the above equations by neglecting the contribution from reflected emission which includes coefficients $r_{⊥,∥}$)

(100) $$S_{0}=\frac{cn_{d}p^{2}k_{0}^{4}}{3}.$$

The core idea is to assume that the observable enhancement of the radiative de-excitation rate $k_{f}$ of a fluorescence emitter due to the presence of the metal substrate with respect to the same emitter in a homogeneous environment is given by the ratio $S(\beta )/S_{0}$ [181]. One more technical detail that one has to take into account is that not every de-excitation of a fluorescence emitter is connected with photon emission. Indeed, de-excitation can also occur non-radiatively, with some rate $k_{\text{non–radiative}}$, by collisions with surrounding molecules and thermal dissipation of the excited state energy; see Sec. II. This is quantified by the fluorescence quantum yield, $Q_{f}$, defined as the probability that de-excitation proceeds radiatively with photon emission; see Eq. 19. The observable fluorescence lifetime τ is the inverse of the total de-excitation rate $k_{f}+k_{\text{non–radiative}}$ (see Eq. 20), so that its change in the presence of the metal substrate is given by

(101) $$\frac{\tau }{\tau_{0}}=\frac{S_{0}}{S(\beta )/S_{0}Q_{f}+\left(1-Q_{f}\right)S_{0}}.$$

This is the final equation needed for calculating the dependence of fluorescence lifetime τ on emitter distance $z_{d}$. An example is provided in Fig. 26 for the three cases of a vertically, horizontally, and randomly oriented emitter. In the latter case, the orientation-dependent S(β) is replaced by its orientation-averaged value $\left\langle S\rangle =(1/2)\int_{0}^{\pi }\;d\beta sin\beta S(\beta \right)$.

As can be seen from Fig. 26, for a randomly oriented emitter, within a range of up to 200 nm from the surface the lifetime depends monotonically to distance. Within this range, a measured lifetime can be converted into a unique distance value. Finally, it should be noted that the newest MIET developments substitute the metal layer by other materials such as Indium Tin Oxide (ITO) [182] or single-sheet graphene (graphene induced energy transfer or GIET) [183], leading to a similar distance-dependent modulation of the fluorescence lifetime but on a *ca.* eight times smaller length scale. This has enabled Ångström resolutions along the optical axis.

### B. Point scanning microscopy

As we move our way towards alternates to wide-field imaging, which records an entire image at once using a multi-pixel detector, we move forward with the point scanning microscope. The latter sequentially records an image by scanning the sample over a set of positions and recording fluorescence signal from each scanning position. In contrast to wide-field imaging, point scanning allows for out-of-focus light reduction and thus accomplishes optical sectioning. In this section, we first consider image formation in the most widely used point scanning microscope: the CLSM [184]. We next discuss enhancedresolution ISM, 4pi microscopy, and two-photon excitation microscopy. We finally provide statistical frameworks for analyses of data acquired using such microscopes.

#### 1. Confocal laser scanning microscopy

**FIG. 28:**
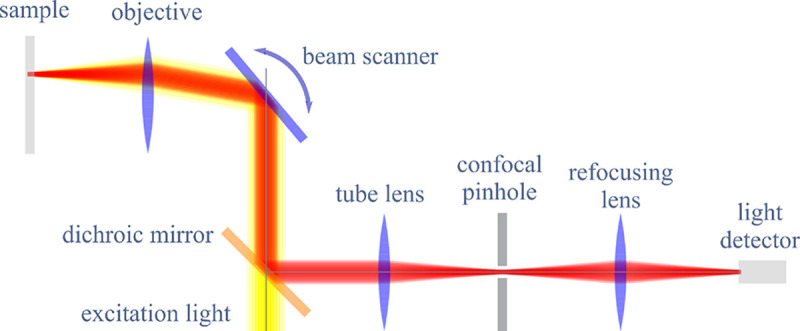
Schematic of a confocal laser scanning microscope (CLSM). Here, the yellow and red beams, respectively, represent the excitation and emission lights. The emission light passes through a confocal pinhole embedded in the setup to supress out-of-focus light. For a detail description of this setup see the text.

A schematic of a point scanning microscope is shown in Fig. 28. An excitation laser beam, shown in yellow, is laterally deflected by a beam scanning unit along the two directions perpendicular to the optical axis. Fig. 28 shows only one of these scanning directions where the excitation beam can be directed up and down upon reflection from the scanner by adjusting the scanner’s orientation. Following deflection, the excitation light is focused by the objective into a diffraction-limited focus within the sample. The emitted fluorescence light from the illuminated spot, designated by a red beam, is then collected by the same objective and guided back through the same beam scanner towards the dichroic mirror. This process is known as de-scanning.

After de-scanning, the fluorescence light is then reflected away from the excitation beam by the dichroic mirror, which only reflects light with certain wavelengths. The fluorescent light is next focused by the tube lens onto the circular aperture of a confocal pinhole obstructing the undesired fluorescent light from out-of-focus fluorophores. After potentially passing additional optical filters for background suppression, the fluorescence light is refocused onto a single-pixel point detector to record the fluorescence intensity from the in-focus fluorophores.

Although the illustration presented for the CLSM is quite different from a wide-field imaging microscope, the image formation can be still described by Eq. 62. The difference in these microscopes is highlighted in their PSFs. The derivation of the PSF for a wide-field microscope was previously described for the most general case and its approximate analytical forms in Secs. IIIC and IIID; see Eqs. 83 and 86. In what follows, we derive the confocal PSF. To avoid notational confusion, PSFs for the wide-field and CLSM are, respectively, denoted by $U_{wf}$ and $U_{cf}$ for the rest of this section.

To derive the confocal PSF (PSF for a single scanning spot), we must consider major differences between this microscope and a wide-field setup including: 1) the spot illumination procedure; and 2) the existence of the confocal pinhole. We do so by assuming an isolated emitter sitting in an excitation focal spot in the sample space. The fluorescent light from this emitter is proportional to the three-dimensional excitation laser intensity at the focal spot $I_{ex}(\rho ,z)$ (excitation is in the sample space and thus described by non-prime coordinates). The fluorescent light is, in turn, collected by the objective and focused onto the confocal pinhole (within the image space). This results in a fluorescent intensity $U_{wf}I_{ex}$ right before the pinhole where $U_{wf}$ is the wide-field PSF of this setup in the absence of the pinhole and spot illumination. In the end, the confocal PSF (imaging PSF of a confocal microscope) is proportional to the fluorescence intensity (ignoring all constant pre-factors) following the pinhole introduced by

(102) $$U_{cf}(\rho ,z)\;\propto \left[A⊗U_{wf}\right]I_{ex}(\rho ,z)\;\;=\int \;d\rho^{\prime }A\left(\rho^{\prime }\right)U_{wf}\left(\rho^{\prime }-\rho ,z\right)I_{ex}\left(\rho ,z\right),$$

where A captures confocal aperture, set to unity for $\rho^{\prime }=\left|\rho^{\prime }\right|$ smaller than the aperture radius a, and zero otherwise. Here, $U_{wf}\left(\rho^{\prime }-\rho ,z\right)$ represents the wide-field PSF when imaging the fluorescence from an emitter at position r=(ρ,z) in sample space onto lateral position $\rho^{\prime }$ in the plane of the confocal aperture within the image space (prime coordinates). Further, we note that the confocal PSF in Eq. 102 is given as product of: $A⊗U_{wf}$ describing the detection, sometimes termed detection PSF; and $I_{ex}$ describing excitation, sometimes termed excitation PSF.

The above integration is performed over the whole $\rho^{\prime }$-plane. The excitation PSF (excitation intensity distribution), $I_{ex}$, that enters the above equation is itself a function of the absorption dipole orientation $p_{ex}$ of a fluorophore via

(103) $$I_{ex}(r)\propto {\left|E_{ex}(r)\cdot p_{ex}\right|}^{2}$$

where $E_{ex}$ denotes the electric field distribution in the focal spot. In most cases of practical interest, one deals with rapidly rotating emitters. In this case, the orientation-averaged excitation intensity is given by

(104) $$I_{ex}(r)\propto {\left|E_{ex,x}\right|}^{2}+{\left|E_{ex,y}\right|}^{2}+{\left|E_{ex,z}\right|}^{2}.$$

Thus, an important part of the PSF calculation for a CLSM is the calculation of the electric field at the laser focus, $E_{ex}(r)$.

**FIG. 29:**
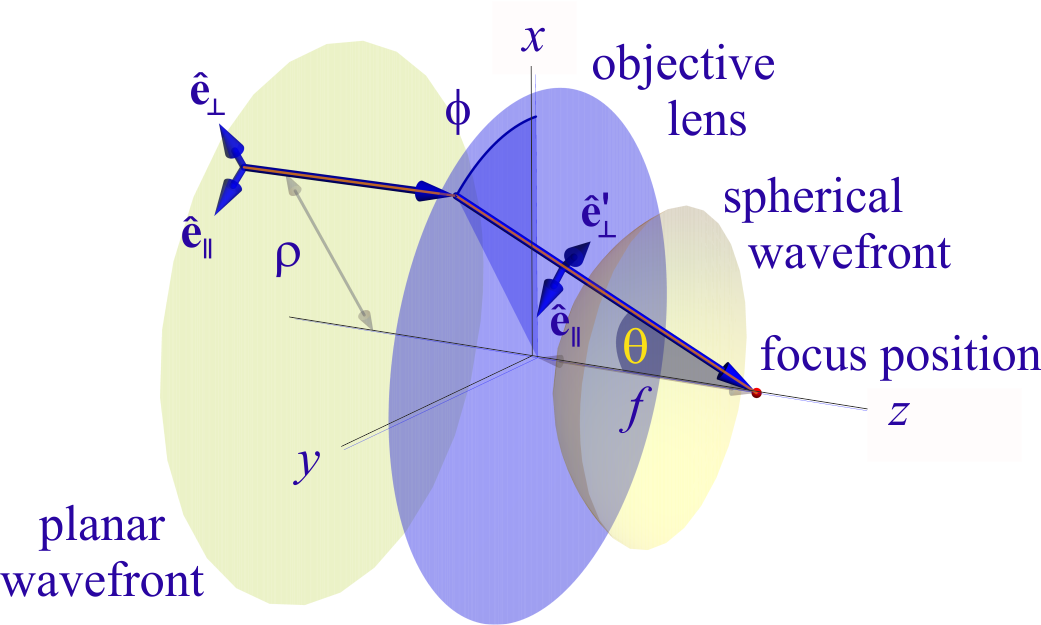
Schematic of the geometry of focusing a planar wavefront through the objective lens into the sample space. Wavefront segments at distance ρ from the optical axis in the back focal plane are converted into spherical wavefront segments traveling at angle θ=arcsin(ρ/nf) with respect to the optical axis z, where f is the focal length of the objective lens; see the main text.

**FIG. 30:**
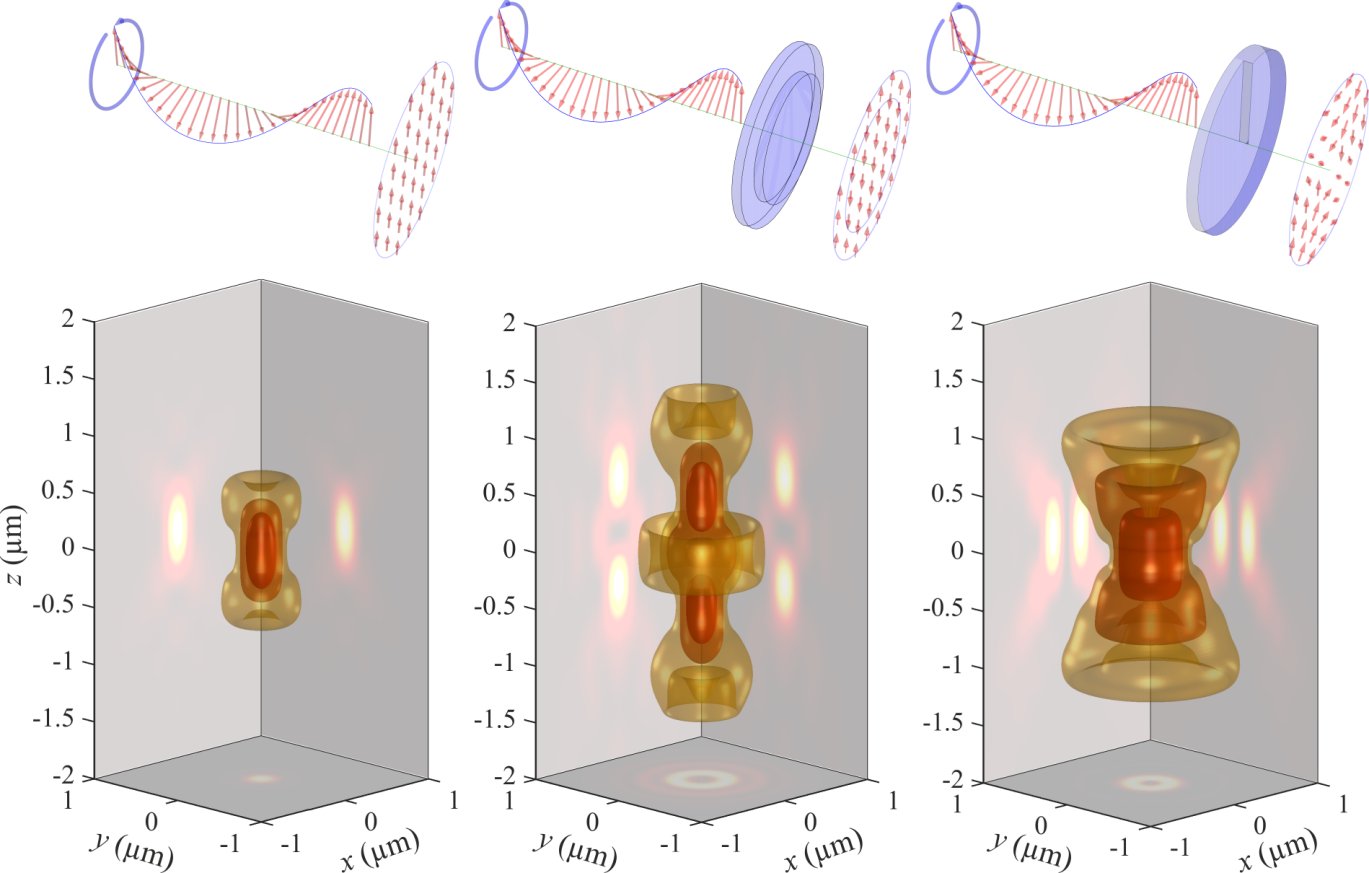
STED excitation. Comparison between conventional excitation focus (left) with z-STED focus (middle) and xy-STED focus (right). Calculations were done for a 1.2 NA water immersion objective at an excitation wavelength of 470 nm. On top of each column, the excitation polarization and its generating phase plate is shown. Bottom panels show 3D contour plots of the $1/e,\;1/e^{2}$ and $1/e^{3}$ intensity iso-surfaces and projections of xy-,xz-, and yz-cross-sections through the center.

To perform this calculation, we first consider the focusing of a planar wavefront through the objective into a diffraction-limited spot; see Fig. 29. Similar to Abbe’s sine condition relating propagation angles of wavefront segments in sample and image spaces, there is a similar relation between the distance from the optical axis of a wavefront patch on the planar wavefront and the propagation angle of the corresponding wavefront patch after focusing through the objective; see Fig. 29. This relation can be found from Abbe’s sine condition when moving the focus point in image space to infinity (*i.e.*, the focal length $f_{\text{tube}}$ of the tube lens tends towards infinity), and remembering that the magnification ℳ is given by the focal distance of the tube lens $f_{\text{tube}}$ divided by the focal distance f of the objective; see Fig. 1. Thus, we find $ℳsin\;\theta^{\prime }=\left(f_{\text{tube}}/f\right)sin\;\theta^{\prime }=nsin\;\theta$. When increasing the value $f_{\text{tube}}$ to infinity, the angle $\theta^{\prime }$ will tend to zero, but the product $\rho =f_{\text{tube}\;}sin\;\theta^{\prime }$ will stay finite and is the distance from the optical axis in the back focal plane. Thus, one finds

(105) ρ=nfsinθ

between the distance ρ of a segment of the incoming laser light wavefront from the optical axis and the propagation angle θ of the corresponding spherical wavefront segment in sample space. Using this relation, one can expand the electric field in sample space into a plane wave superposition, similar to what we have done for deriving the electric field of a point emitter in image space, see Eq. 56. When reading Eq. 56 in reverse, *i.e.*, replacing all primed variable by non-primed and vice versa (thus starting with light coming from the back side of the objective focused through the objective into sample space), and when taking into account that the angles $\theta^{\prime }$ for the incoming light are all zero (plane wavefront), so that $cos\;\theta^{\prime }\approx 1$, one arrives at

(106) $$\begin{array}{ll} & E_{ex}(r)\propto \int_{0}^{\Theta }\;\;d\theta sin\;\theta \sqrt{cos\;\theta }\int_{0}^{2\pi }\;\;d\phi \\ & \left[E_{0,∥}(\rho ,\phi ){\overset{ˆ}{e}}_{∥}+E_{0,⊥}(\rho ,\phi ){\overset{ˆ}{e}}_{⊥}^{\prime }\right]exp\left(ik_{ex}\cdot r\right) \end{array}$$

where $k_{ex}=2\pi n/\lambda_{ex}(\cos\phi sin\;\theta ,\sin\phi sin\;\theta ,cos\;\theta )$ is now the wave vector of a plane wave having wavelength $\lambda_{ex}$ (excitation light wavelength), where the electric field of the incoming laser beam in the back focal plane is expanded into its radially $\left(E_{0,⊥}\right)$ and azimuthally $\left(E_{0,∥}\right)$ polarized parts, see Fig. 29. For example, for a linearly polarized laser beam with polarization direction along x one has $E_{0,⊥}\propto cos\phi$ and $E_{0,∥}\propto -sin\phi$. This equation can now be used for calculating the three-dimensional excitation PSF in sample space, and as an example, the left panel of Fig. 30 shows the calculation result for focusing a 470 nm circularly polarized laser through a water immersion objective into a diffraction-limited spot (planar wavefront at the back focal plane).

However, Eq. 106 is much more general. For instance, it can be also used for calculating the intensity distribution of a donut beam as used in STED microscopy [56]. There are two principal methods for generating a donut intensity distribution with zero intensity on the crossing of the optical axis with the focal plane (focus center).

The first method sends a circularly polarized laser light through a ring-shaped phase plate thicker at its center. This results in retardation of the beams of light closer to optical axis by half a wavelength with respect to the beams that path through the thinner outer part of the plate; see the central panel in Fig. 30. A snapshot of the resulting polarization structure across the back focal plane is depicted in the top middle panel in Fig. 30. Mathematically, this can be described by setting $E_{0,⊥}\propto \cos\phi -i\;sin\phi$ and $E_{0,∥}\propto -sin\phi +icos\phi$ for $\rho \leq \rho_{\Phi }$ and the same expressions but with opposite sign for $\rho_{\Phi }<\rho <fsin\Theta$, where $\rho_{\Phi }=fsin\Theta /\sqrt{2}$ is the radius of the thicker central part of the phase plate. This special choice of $\rho_{\Phi }$ assures that the total excitation intensity in the focus center is indeed zero. The second method for generating a donut-shape laser intensity in the focal plane is to send circularly polarized light through a helical wave plate as shown at top of the right panel in Fig. 30. When choosing an appropriate helical pitch, this leads to an excitation beam with polarization structure $E_{0,⊥}\propto sin2\phi -icos2\phi$ and $E_{0,∥}\propto cos2\phi +isin2\phi$. Three-dimensional representations of the resulting STimulated Emission (STE) intensity distributions and the corresponding cross-sections are shown in the bottom panels of Fig. 30. As can be seen, neither the disk phase plate (middle panel) nor the helical phase plate (right panel) lead to an ideal STE intensity distribution. Whereas the disk phase plate leads to an intensity distribution that achieves excellent axial compression of the STED-PSF, it performs poorly in lateral directions. By contrast, the helical wave plate leads to an excellent compression of the STED-PSF laterally, but not along the optical axis. Thus, 3D-STED systems use a combination of both excitation modalities [185].

Now, having in place an exact description of the excitation PSF (excitation intensity distribution), we can return to the imaging PSF of CLSM and consider its optical resolution. To do so, we consider its OTF, *i.e.*, the Fourier transform of Eq. 102, for which we replace $I_{ex}$ and $U_{wf}$ of Eq. 102 by their Fourier expansions,

(107) $$U_{wf}\left(\rho^{\prime }-r\right)\;=\int \;\frac{dk}{2\pi }{\tilde{U}}_{wf}(k)exp\left[ik\cdot \left(\rho^{\prime }-r\right)\right]\;\;I_{ex}(r)\;=\int \;\frac{dk}{2\pi }{\tilde{I}}_{ex}(k)exp(ik\cdot r)$$

where we recall that a tilde over a symbol denotes its Fourier amplitude. This immediately leads to

(108) $$U_{cf}(r)\;\propto \int \;d\rho^{\prime }\int \;dk\int \;dk^{\prime }A\left(\rho^{\prime }\right){\tilde{U}}_{wf}\left(k^{\prime }\right)\;\;exp\left[ik^{\prime }\cdot \left(\rho^{\prime }-r\right)\right]{\tilde{I}}_{ex}(k)exp(ik\cdot r).$$

The integration over $\rho^{\prime }$ can be now carried out analytically, resulting in

(109) $$\int \;d\rho^{\prime }A\left(\rho^{\prime }\right)exp\left(ik^{\prime }\cdot \rho^{\prime }\right)=\frac{2\pi a}{q^{\prime }}J_{1}\left(aq^{\prime }\right)$$

where a is, as before, the radius of the confocal aperture, $q^{\prime }=\sqrt{k_{x}^{\prime 2}+k_{y}^{\prime 2}}$ is the modulus of the radial part of the vector $k^{\prime }$, and $J_{1}$ is the first order Bessel function of the first kind. Substituting this result into Eq. 108, we write

(110) $$U_{cf}(r)\;\propto \int \;dk\int \;dk^{\prime }\frac{2\pi a}{q^{\prime }}J_{1}\left(aq^{\prime }\right)\;\;{\tilde{U}}_{wf}\left(k^{\prime }\right){\tilde{I}}_{ex}(k)exp\left[i\left(k-k^{\prime }\right)\cdot r\right].$$

After a few substitutions for the integration variables and some algebra, we find for the Fourier transform of $U_{cf}(r)$, *i.e.*, the OTF of a CLSM (up to some constant pre-factor)

(111) $${\tilde{U}}_{cf}(k)\propto \int \;dk^{\prime }\frac{J_{1}\left(aq^{\prime }\right)}{q^{\prime }}{\tilde{U}}_{wf}\left(k^{\prime }\right){\tilde{I}}_{ex}\left(k+k^{\prime }\right).$$

**FIG. 31:**
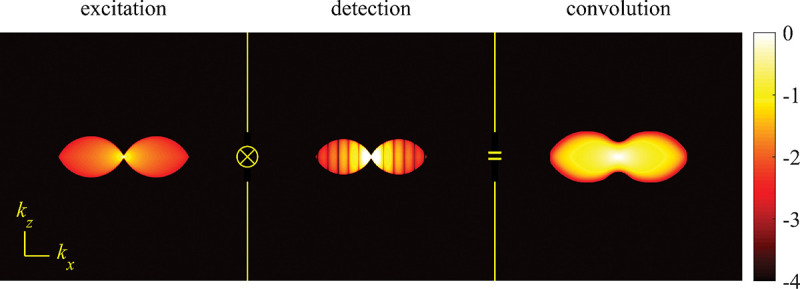
Anatomy of the OTF of a confocal microscope. The left panel shows the excitation OTF. The middle panel shows the detection OTF for a confocal pinhole with 50μm radius and 60 times magnification. The right panel shows the resulting confocal OTF obtained by a 3D convolution of the left two distributions.

Thus, the OTF of the confocal microscope is given by the three-dimensional convolution of a wide-field microscope OTF, ${\tilde{U}}_{wf}(k)$, modulated by the aperture function, $J_{1}\left(aq^{\prime }\right)/q^{\prime }$, (Fourier transform of detection PSF (see Eq. 102) also sometimes termed detection OTF) and the Fourier transform of the excitation PSF, ${\tilde{I}}_{ex}(k)$ (also sometimes termed excitation OTF). This is visualized in Fig. 31, where the left panel shows amplitude of the excitation OTF, ${\tilde{I}}_{ex}(k)$, the middle panel is the detection OTF given by the wide-field OTF ${\tilde{U}}_{wf}(k)$ multiplied by $J_{1}\left(aq^{\prime }\right)/q^{\prime }$, and the right panel represents a cross-section of the confocal OTF obtained by 3D convolution of the previous two panels.

**FIG. 32:**
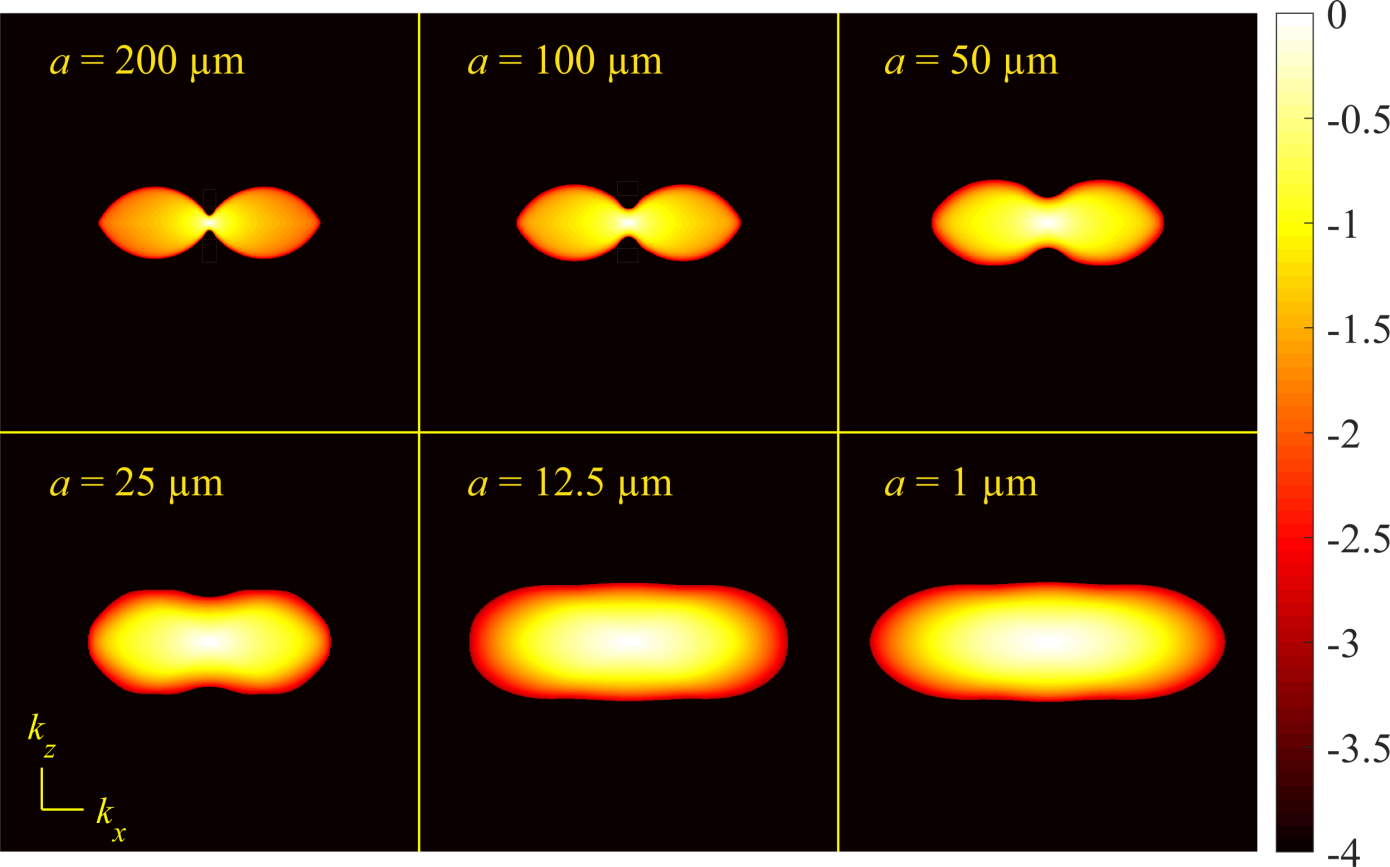
OTF of a confocal microscope as a function of confocal aperture size. The confocal aperture radius is given at the top of each panel. Here we take the excitation wavelength to be 470 nm, emission wavelength to be 550 nm, and assume imaging is achieved with a water immersion objective of 1.2 NA at 60× magnification. The top most left panel shows the limit of an extremely large confocal pinhole so that the OTF approaches that of a wide-field microscope imaging at the same wavelength as the excitation wavelength of the excitation laser. The bottom right panel shows the limit of a nearly zero-size pinhole (a=1μm), so that the OTF approaches that of an ISM, see Sec. IV B 2.

The most noticeable difference between the confocal OTF in Fig. 31 and the wide-field OTF in Fig. 21 is that the confocal OTF has non-zero components along the optical axis (here $k_{x}=0$ with the origin at the center). This signifies that a confocal microscope allows for optical sectioning. The corresponding axial resolution is given by 2π divided by the maximum frequency supported along the $k_{z}$-axis; see Eq. 67. Fig. 32 depicts how the confocal OTF changes with the pinhole size. Here, in the limit of an extreme large confocal pinhole radius of 200μm (top left panel), the confocal OTF approaches that of a widefield microscope at the same wavelength, as can be seen by comparing with the left panel of Fig. 31. In the limit of close to zero pinhole size (a=1μm), optical sectioning and axial resolution are optimized. This is shown in the bottom right panel of Fig. 32. In this case, the confocal aperture can be approximated by a delta function so that the integral Eq. 109 results in a constant. Therefore, the OTF for a very small aperture reduces to the convolution of the wide-field OTF, ${\tilde{U}}_{wf}$, with the excitation OTF, ${\tilde{I}}_{ex}$. Thus the maximum frequency passed by the confocal OTF with a small aperture is given by $k_{max}=k_{max,ex}+k_{max,em}$ where $k_{max,\text{ex}\;}$ and $k_{max,em}$, respectively, denote the maximum extents of ${\tilde{I}}_{ex}$ and ${\tilde{U}}_{wf}$. Now, similar to Eqs. 67 and 84–85, we find that for a confocal microscope with a small aperture, the resolution is given by $2\pi /\left(k_{max}\right)$.

The maximum extents of excitation and detection OTFs in the lateral direction are $k_{max,ex/det}=4\pi n\;sin\Theta /\lambda_{\text{ex/em}}$, which, in turn, results in the following lateral resolution

(112) $$y_{min}=\frac{1}{2NA}{\left(\frac{1}{\lambda_{ex}}+\frac{1}{\lambda_{em}}\right)}^{-1},$$

and similarly for the axial resolution

(113) $$z_{min}=\frac{1}{2n(1-cos\Theta )}{\left(\frac{1}{\lambda_{ex}}+\frac{1}{\lambda_{em}}\right)}^{-1},$$

where $\lambda_{\text{ex}}$ and $\lambda_{em}$ are the excitation and emission wavelengths, respectively. Thus, if one ignores the spectral Stokes shift between excitation and emission, *i.e.*, $\lambda_{em}\approx \lambda_{ex}$, (see Sec. II) then the confocal microscope with infinitely small pinhole has a two times higher lateral resolution than a wide-field microscope as we can see by comparing Eq. 112 to Eq. 84. This improvement in resolution can also be explained in the spatial domain using Eq. 102 when setting the pinhole function A to a delta function $A\left(\rho^{\prime }\right)=\delta \left(\rho^{\prime }-\xi \right)$ (infinitesimally small circular aperture centered at ξ) and adopting Gaussian approximations for both the wide-field PSF (detection PSF) as in Eq. 86 and excitation PSF $I_{ex}$, the resulting confocal PSF would be the product of the two Gaussians. Then, the resulting confocal PSF is a Gaussian as well [186]

(114) $$U_{cf}\left(\rho ,z\right)\propto \exp\left(-\frac{{\left(\rho -\xi_{\rho }\right)}^{2}}{2\sigma_{\rho }^{2}}-\frac{{\left(z-\xi_{z}\right)}^{2}}{2\sigma_{z}^{2}}\right).$$

Here, the widths of the resulting Gaussian PSF, $\sigma_{\rho }$ and $\sigma_{z}$, are smaller than the widths of both excitation and detection PSFs leading to higher resolutions.

**FIG. 33:**
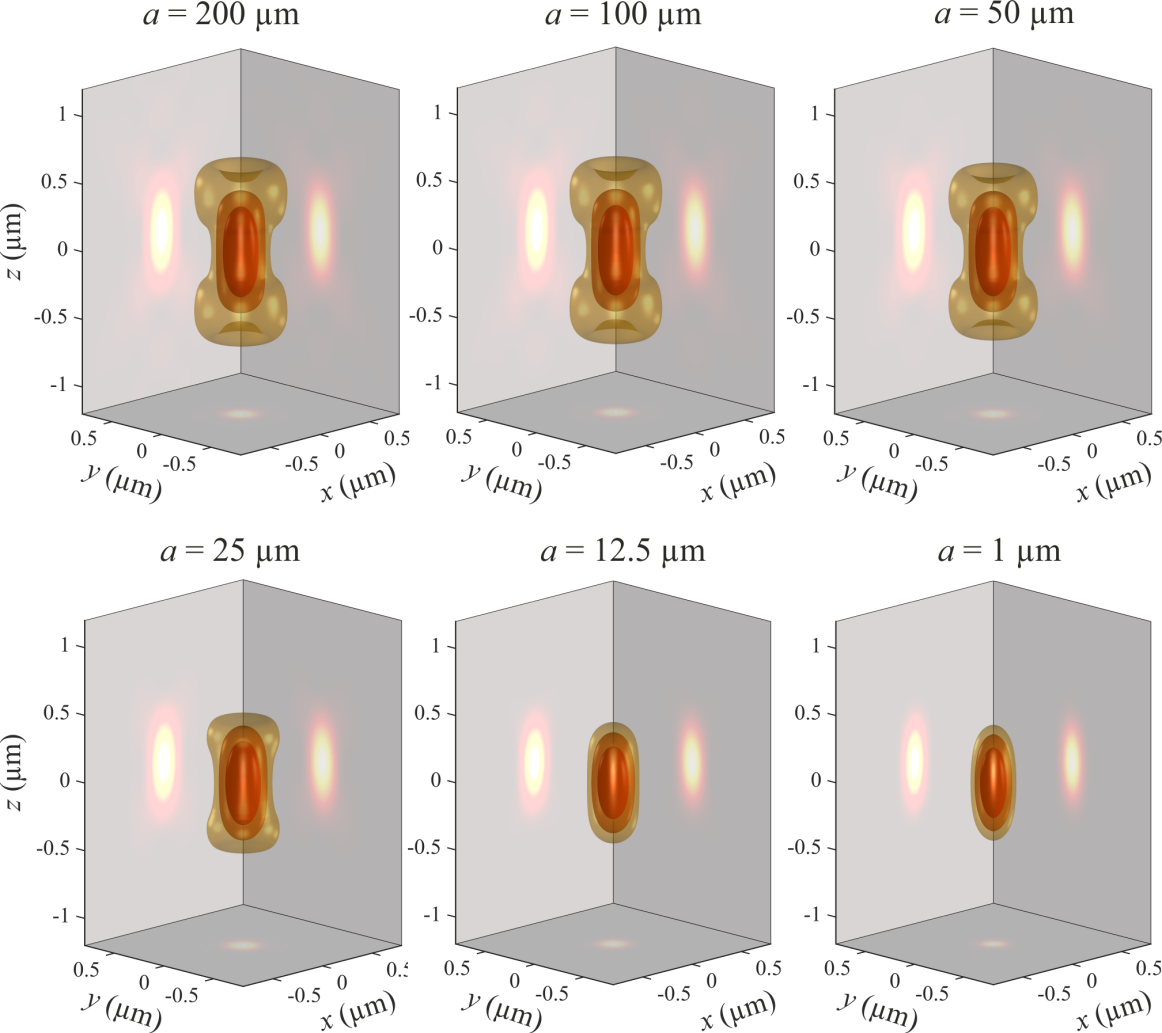
Confocal microscope PSF as a function of confocal aperture size. The confocal aperture radius is given above each panel. As before, the excitation wavelength is taken as 470 nm, emission wavelength taken as 550 nm, and imaging is achieved with a water immersion objective of 1.2 NA at 60 times magnification. PSFs for an isotropic emitter are shown.

**FIG. 34:**
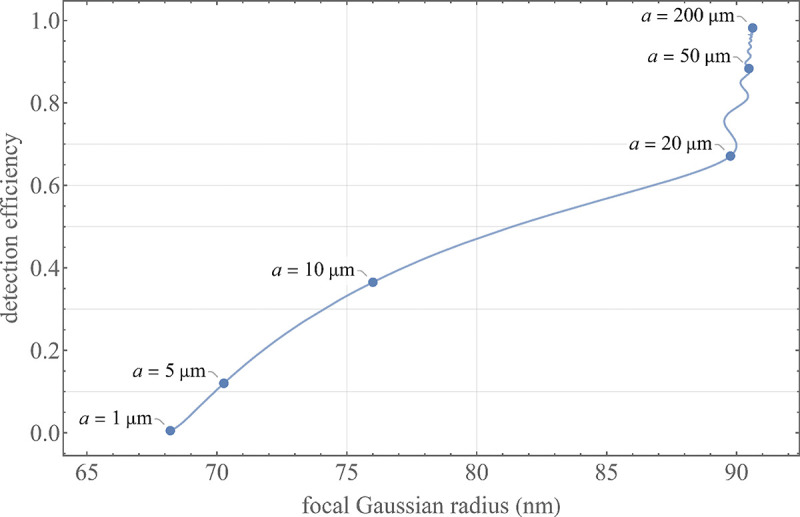
Relation between PSF size and detection efficiency in a CLSM. Here we show the light detection efficiency versus the Gaussian radius σ of the PSF in the focal plane as a function of radius a of the confocal aperture as given by the annotations. Calculations were done for a 1.2 NA water immersion objective and image magnification of 60× (focal plane to pinhole plane). It was assumed that excitation is achieved with 470 nm circular polarized light focused into a diffraction-limited spot, and that the fluorescence emission is of 550 nm wavelength. We determined the focal radius by fitting a radially symmetric Gaussian $exp\left(-\rho^{2}/2/\sigma^{2}\right)$ to the PSF in the focal plane.

The PSFs corresponding to the OTFs shown in Fig. 32 are presented in Fig. 33. This shows how the PSF’s lateral width shrinks with decreasing pinhole size leading to higher lateral resolutions. However, the higher lateral resolution comes with a price: the smaller the confocal pinhole size, the fewer photons reach the detector resulting in low SNR [187]. This is quantified in Fig. 34 which shows the relation between PSF diameter (in the focal plane) and light detection efficiency for different pinhole radii ranging from 1μm to 200μm (assuming 470 nm excitation and 550 nm emission wavelength, and for a water immersion microscope with 1.2 NA objective and 60 times magnification). As can be seen, the light detection efficiency dramatically decreases when the confocal pinhole radius drops below 20μm. An elegant way to achieve optimal resolution (limit of infinitely small pinhole size) without sacrificing light detection efficiency is via so-called ISM discussed in Sec. IV B 2.

#### 2. Image scanning microscopy

As was discussed in Sec. IV B 1 when considering a confocal PSF, the maximum possible spatial resolution was achieved in the limit of an infinitely small confocal pinhole; see Eqs. 112–114. However, as this would reduce light detection efficiency to zero (see Fig. 34), such an option is never used in real confocal microscopes. In order to simultaneously maximize spatial resolution and light detection efficiency, now beyond three decades ago, Colin Sheppard proposed to combine scanning spot illumination of confocal microscopes and the wide-field light detection of an array detector, *e.g.*, EMCCD camera, without pinholes to mitigate light loss and improve resolution [47]. This idea, termed ISM, was first experimentally demonstrated in 2010 by Müller and Enderlein [48]. The core idea of ISM is to replace the confocal pinhole and the single pixel detector of a conventional CLSM by an array detector in the image plane (pinhole plane); see Fig. 28. The fluorescence light from an illumination spot at position r is then spread across multiple pixels of the detector array. In this setup, a pixel located at ξ records photons from the illuminated spot corresponding to a pinhole located at ξ with the same size as the pixel size. The pixel size is often chosen small enough such that each pixel records an image of the illumination spot with a resolution similar to that of CLSM with close to zero pinhole sizes; see Eqs. 112 and 113. Moreover, because ISM builds on a CLSM, it also provides optical z-sectioning.

**FIG. 35:**
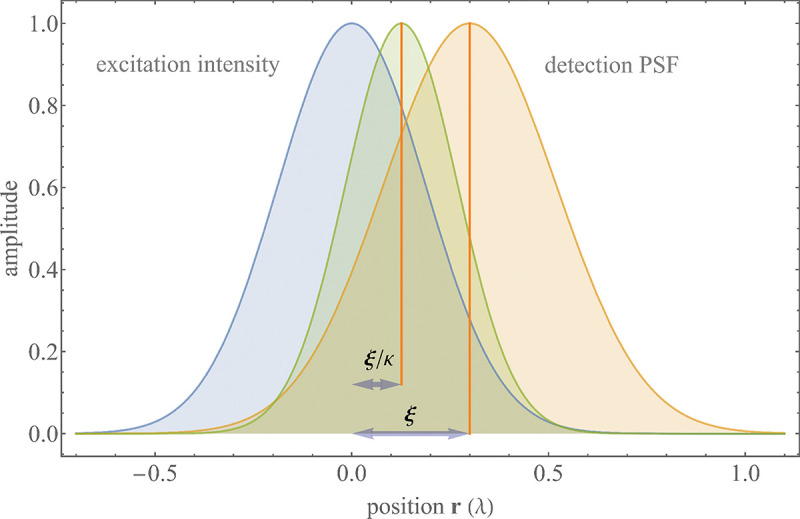
Image formation in ISM. The blue curve represents the excitation intensity distribution $I_{\text{ex}}$ (excitation PSF) with its center at ξ=0 (optical axis). The yellow curve shows the detection PSF $\left(U_{wf}\right)$ for a pixel located at ξ away from the optical axis. The pixel PSF $\left(U_{\text{pix}\;}\right)$, which describes the image formation is, however, given by the product of the excitation PSF and the detection PSF, designated by the green curve and centered at ξ/κ. Therefore, if a fluorophore is located at ξ=0, it will artificially look like to be at ξ/κ.

The ISM setup described here, results in $N_{p}$ recorded images for each illumination spot associated to all $N_{p}$ pixels of the detector array. As such, upon scanning the sample at total $N_{s}$ locations, one acquires $N_{p}\times N_{s}$ images. To combine all the acquired images into a single high resolution image, we first consider the scan image recorded by one pixel at a given position ξ on the array detector. The PSF of this scan image is easily found when replacing the aperture function A(ρ) of Eq. 102 by the pixel area. However, as an idealization, we can consider the pixel area as a delta function δ(ρ-ξ) as compared to the size of features we care to learn. As such, the PSF for the scan image recorded by a pixel at position ξ is

(115) $$U_{pix}(r,\xi )\propto U_{wf}(\xi -r)I_{ex}(r)$$

where, as before, $U_{wf}$ is the wide-field imaging PSF (detection PSF), and $I_{ex}$ is the excitation PSF. This is visualized in Fig. 35] where a cross-section of the excitation PSF $I_{ex}(r)$ is shown together with the detection PSF for a pixel at position ξ (described by $\left.U_{wf}(\xi -r\right)$) and the product of both; see Eq. 115.

When approximating the excitation PSF by a Gaussian with variance $\sigma_{ex}^{2}$ and the detection PSF by a Gaussian with variance $\sigma_{em}^{2}$, we find for the product of both

(116) $$I_{ex}(r)U_{wf}(r-\xi )\propto exp\left[-\frac{(r-\xi /\kappa {)}^{2}}{2\sigma_{PSF}^{2}}\right]$$

with $\sigma_{PSF}^{-2}=\sigma_{ex}^{-2}+\sigma_{em}^{-2}$, and $\kappa =1+\sigma_{em}^{2}/\sigma_{ex}^{2}$. When recalling that $\sigma_{ex}$ and $\sigma_{em}$ linearly scale with wavelength (see Eq. 86), we find the important relation for the factor κ

(117) $$\kappa =1+{\left(\frac{\lambda_{em}}{\lambda_{ex}}\right)}^{2}$$

which equals 2 if one neglects the spectral Stokes shift between excitation and fluorescence emissions. Thus, the maximum of the product of excitation intensity distribution and detection PSF is located between the centers of both at position ξ/κ, such that the scan image is shifted by the same amount with respect to an image recorded by a pixel at position ξ=0; see Fig. 35. This insight yields a recipe for how to super-impose different scan images recorded by different pixels: an image recorded by a pixel at position ξ has to be shifted by ξ/κ towards the optical axis before being added to the final sum image. Mathematically, this is given by

(118) $$\begin{array}{ll} & U_{ISM}(r)\propto \int \;d\xi U_{pix}\left(r+\frac{\xi }{\kappa },\xi \right)\\ & \;=\int \;d\xi U_{wf}\left(\frac{\kappa -1}{\kappa }\xi -r\right)I_{ex}\left(r+\frac{\xi }{\kappa }\right). \end{array}$$

**FIG. 36:**
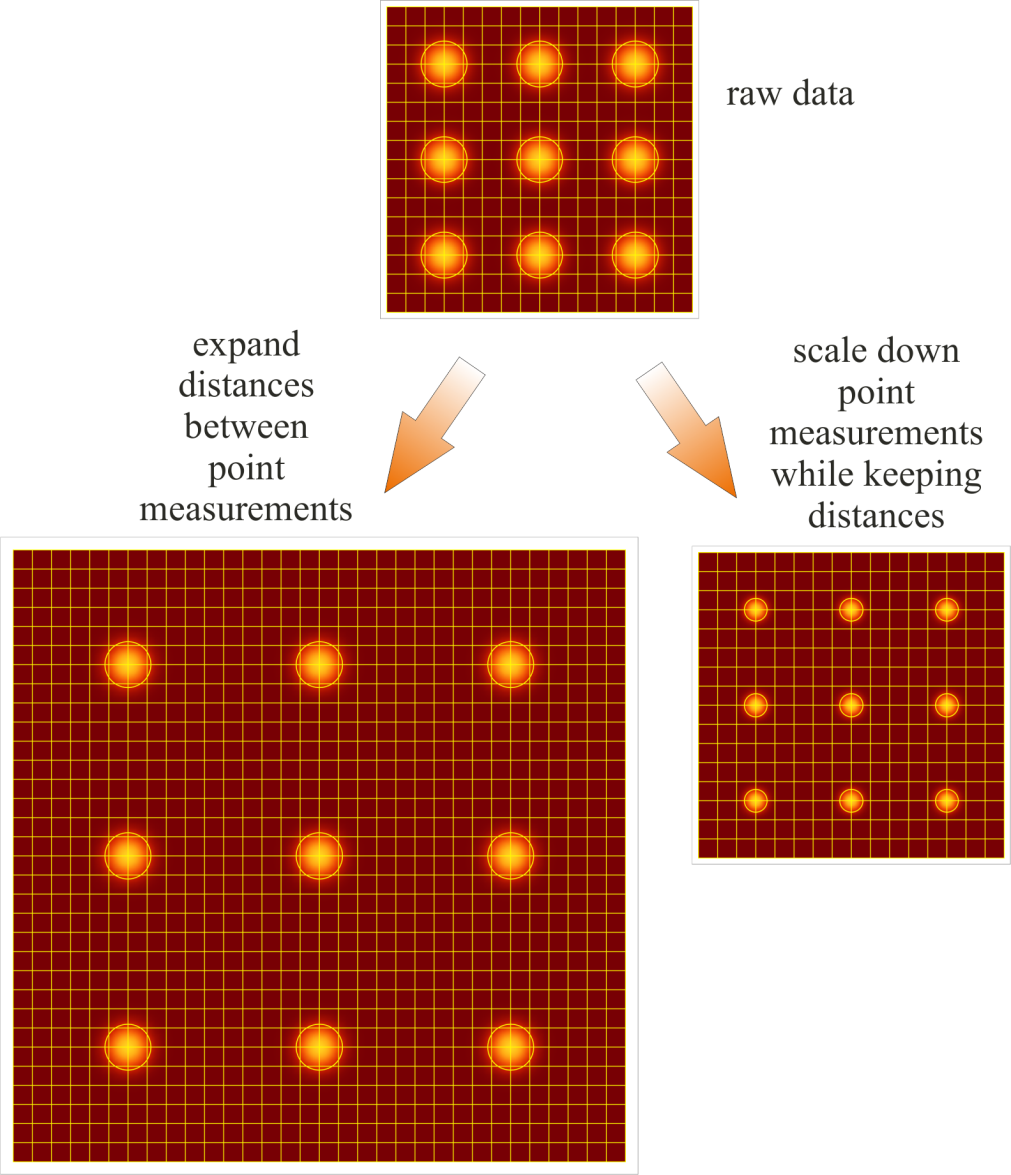
Data evaluation of ISM raw data. At each scan position of the laser focus, the array detector records a small image of the illuminated region (top). For calculating a final ISM image, one can either down-scale each recorded small image by factor κ (bottom right), or one leaves the recorded images unchanged but places them in the final ISM image by the factor κ farther way from each other (bottom left).

There are two ways to realize this in practice. As shown in Fig. 36, one way is to scale down, by factor κ, all images recorded by the array detector at each scan position before adding them to the final image at the corresponding scan position (from top to bottom right in figure). Alternatively, one can leave the recorded array detector images as they are, but place them a factor κ farther away from each other when adding them to the final image (from top to bottom left in figure).

Obviously, both procedures are mathematically equivalent ways to realize the algorithm described by Eq. 118, although the second algorithm is numerically simpler because it does not require any interpolation based downscaling of the images recorded by the array detector. However, as first realized by York and Shroff [188] and by de Luca and Manders [189], both algorithms can be realized in a fully optical way. The first algorithm which down-scales the array detector images can be optically realized by inserting an extra demagnifying lens pair into the detection pathway (as realized by instant SIM [188, 190], Optical Photon Re-Assignment or OPRA [191], or confocal spinning disk ISM [192]), while the second algorithm which up-scales distances between recorded images can be realized by a double mirror re-scan system (re-scan microscopy [189]) or by re-coupling the emission into the excitation scan system (rapid two-photon excitation ISM [193]).

By way of its construction, both OTF and PSF of an ISM are identical to that of a confocal microscope with an infinitely small confocal pinhole; see last panels of Fig. 32 (OTF) and Fig. 33 (PSF), respectively. The corresponding achievable optical lateral and axial resolutions then immediately follow from Eqs. 112 and 113. One important particular property of ISM is that it also “concentrates” the collected fluorescence light into an area of the final image four times smaller than that of a conventional CLSM (“super-concentration of light”, [194], see also top and right panel of Fig. 36), which significantly increases image contrast. Meanwhile, there exist numerous different variants of ISM [195], and several commercial systems are available. It is expected that in the near future, all CLSMs will also offer an ISM option for doubled resolution and high contrast imaging.

#### 3. 4pi microscopy

**FIG. 37:**
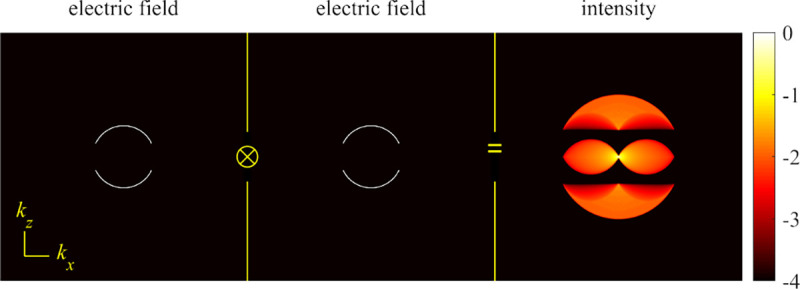
Excitation OTF of a 4pi microscope, generated by the interference of light focused through two opposing objectives. The left and middle panel show the same Fourier transform of the excitation electric field in sample space. The resulting excitation OTF shown in the right panel is the (auto)convolution of this electric field Fourier transform and represents the Fourier transform of the excitation intensity (excitation OTF). Excitation is assumed to be done with a 1.2 NA water immersion objective.

**FIG. 38:**
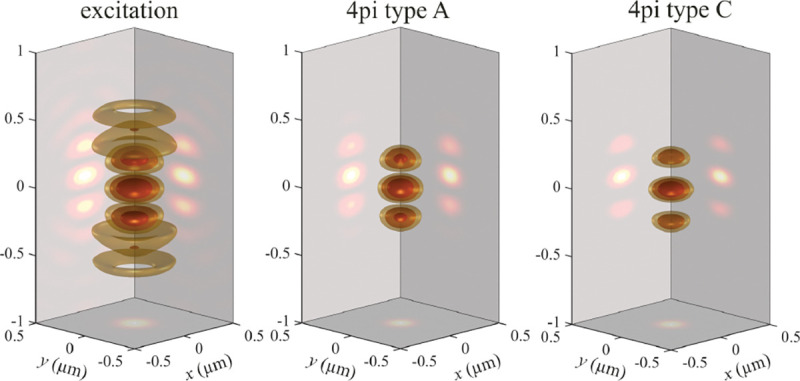
Excitation PSF and (imaging) PSF of 4pi microscopy. The left panel shows the excitation PSF in the focus of a 4pi microscope, the middle panel shows the (imaging) PSF of a 4pi type A microscope, and the right panel that for a 4pi type C microscope. Calculations were done for a 1.2 NA water immersion microscope with 470 nm excitation wavelength and 550 nm fluorescence emission wavelength, and for a confocal detection in the limit of an infinitely small pinhole. PSFs for a rapidly rotating (random or isotropic) emitter are shown.

One peculiarity of conventional CLSM is the disparity between lateral and axial resolution, see Eqs. 112–113. The elongated shape of the PSF along the optical axis (see Fig. 33) leads to 3D CLSM images with an artificial stretching along the optical axis. To overcome this strongly anisotropic resolution, Stelzer and Hell developed 4pi-microscopy using two opposing microscopy objective for focusing (and detecting) light [49]. When sending laser excitation light through both objectives in a *coherent* manner, the resulting interference of both beams generates a multi-peaked interference pattern along the optical axis. The corresponding Fourier representations of the excitation electric and magnetic fields are shown in the left and middle panels of Fig. 37, and the convolution of both, *i.e.*, the 4pi excitation OTF, is shown in the right panel of Fig. 37. By contrast to the CLSM excitation OTF in Fig. 31, its 4pi counterpart populates high frequencies along the optical axis, coinciding with a tight modulation of the excitation intensity along this axis. The corresponding excitation intensity distribution (excitation PSF) in real space is shown in the left panel of Fig. 38.

Detection in a 4pi microscope is done as usual in confocal detection mode, whereby two principal options are possible: 1) fluorescence is collected with both objectives and detected by two detectors resulting in two independent scan images added later to attain a single image (4pi type A microscope [196]); 2) fluorescence is collected with both objectives and coherently super-imposed onto one detector (4pi type C microscope [197]). As a special case is the 4pi type B microscope where excitation is done incoherently (*i.e.*, with no interference pattern generation) but the collected light is super-imposed coherently [198]. In its optical properties, it performs similarly to the type A microscope.

**FIG. 39:**
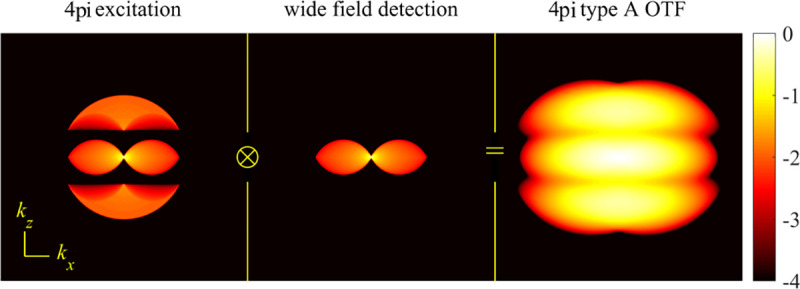
OTF of an A-type 4pi microscope. In this configuration, excitation is done through two opposing objectives, and detection from one side through a confocal pinhole. For simplicity, we consider here only the limiting case of an infinitely small pinhole which would yield maximum possible spatial resolution. The left panel shows the excitation OTF, the middle panel the OTF of detection with an infinitely small pinhole, and the right panel shows the resulting 4pi OTF as a convolution of the two distributions on shown the left. Excitation and detection is assumed to be done with a 1.2 NA water immersion objective, and any Stokes shift between excitation and emission light is neglected.

**FIG. 40:**
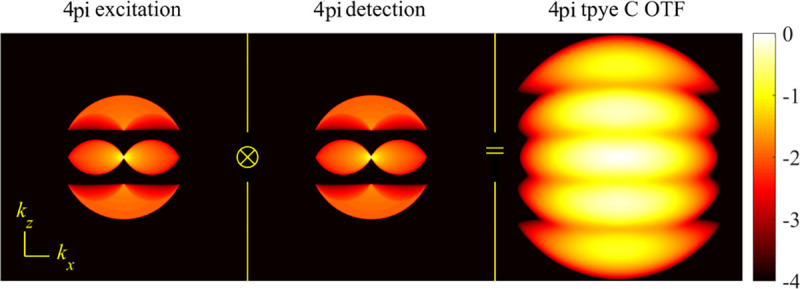
OTF of an C-type 4pi microscope. Similar to Fig. 39, but in this configuration, both excitation and detection is done through two opposing objectives. Again. we consider here only the limiting case of an infinitely small pinhole which would yield maximum possible spatial resolution. The left panel shows the excitation OTF, the middle panel the (identical) Fourier transform for *coherent* confocal detection from both side, and the right panel the resulting OTF as convolution of the two panels shown on the left.

To determine the maximal possible resolution attainable with 4pi microscopy, we show in Figs. 39 and 40 the OTFs for type A and type C microscopes in the limit of an infinitely small confocal pinhole (which could be realized by a combination of 4pi microscopy with ISM). Thus, the OTF of a 4pi type A microscope as shown in Fig. 39 is obtained by a convolution of the 4pi excitation OTF (see Fig. 37) with the OTF of a simple ISM (corresponding to wide-field detection). As mentioned, in a 4pi type C microscope, detection is achieved by coherently superposing fluorescence light from both objectives. In this case, the Fourier representations of the detection electric and magnetic fields can be represented by two incoming counter-propagating spherical wavefronts as shown in the left and middle panels of Fig. 37, so that the OTF of such detection looks similar to that of the excitation shown in Fig. 37, except calculated for the fluorescence emission wavelength. The convolution of such a detection OTF with the excitation OTF then yields the OTF of the 4pi type C microscope; see Fig. 40. The corresponding real space PSFs for both type A and type C 4pi microscopes are shown in the middle and right panels of Fig. 38.

As can be seen in Figs. 39–40, 4pi microscopes collect more spatial frequencies by contrast to CLSM (see Fig. 32) and thus their axial resolutions are indeed much better than CLSM. As before, we can again obtain quantitative numbers for the lateral and axial resolutions by inspecting the OTF and determining the maximum lateral and axial frequencies that are supported by the OTF. The inverse of these maximum frequencies multiplied by 2π then yields approximate values for resolution; see Eq. 67. The lateral resolution of a 4pi microscope (with an infinitely small pinhole) is the same for both type A and C and equal to that of an ISM; see Eq. 112. However, the axial resolution of a type A 4pi microscope now reads

(119) $$z_{min}\approx \frac{1}{2}{\left[\frac{1}{\lambda_{ex}}+\frac{1}{\lambda_{em}}(1-cos\Theta )\right]}^{-1}$$

and similarly for the type C 4pi microscope

(120) $$z_{min}\approx \frac{1}{2}{\left(\frac{1}{\lambda_{ex}}+\frac{1}{\lambda_{em}}\right)}^{-1}.$$

As can be seen from the PSFs in Fig. 38, there are considerable side-lobes next to the central maximum along the optical axis, leading to “ghost” images in a recorded 3D scan image of a sample [197]. These ghost images are much more pronounced for type A than type C, though even for type C they must be eliminated, currently by applying image deconvolution algorithms [199, 200]. Both the technical complexity of a 4pi microscope as well as the difficulties of image deconvolution required for elimination of ghost images have prevented their further distribution. However, the ISM lateral resolution of a 4pi type C (image scanning) microscope together with its extraordinary axial resolution represent the maximum possible spatial resolutions available along the x and z directions possible with a diffraction-limited microscope.

#### 4. Two-photon microscopy

An important variant of the point scanning microscope is the two-(or multi-)photon excitation scanning microscope [201]. Here, a fluorophore is excited by a two-(or multi-)photon absorption process, typically with an excitation wavelength roughly twice (or multiple times) as large as that of one-photon absorption fluorescence excitation. Such two-photon excitation microscopes have several important properties for bio-imaging [202, 203]. First, due to the longer excitation wavelength, typically in the infrared, excitation light can usually penetrate much deeper into tissue than visible light. Thus, two-photon excitation microscopes are ideal for deep-tissue imaging in lipid and water rich tissues with high optical absorption in the visible spectrum. Second, there is a tremendous improvement in signal to background, *i.e.*, undesired light from out-of-focus fluorophores, ratio compared to one-photon absorption fluorescence microscopy due to: 1) fluorophore excitation taking place at much shorter wavelengths than the excitation wavelength. In other words, the probability of simultaneous absorption of two or more photons is only significant at the focal spot with high photon density; 2) light scattering is decreased at longer wavelengths; and 3) two-(or multi-)photon excitation does not require confocal detection for optical sectioning. This is because the two-photon excitation PSF is proportional to the *square* of the excitation light intensity distribution (*square* of the one-photon excitation PSF), represented by an auto-convolution of the excitation OTF in Fourier space. A similar convolution was already considered when discussing the ISM’s OTF (*i.e.*, as idealized by the last panel of Fig. 32), covering higher spatial frequencies by contrast to the OTF of a wide-field microscope or a CLSM with infinitely wide pinhole as shown in the first panel of Fig. 32. Thus, a two-photon excitation microscope has a similar confocal sectioning capability as a one-photon excitation microscope at the same excitation wavelength but with close to infinitely small detection pinhole (when neglecting the spectral Stokes shift between excitation and emission). The downside of two-photon excitation microscopy is the required peak power of the excitation pulses which has to be by orders of magnitude larger than that used in single-photon excitation microscopy. This can lead to substantial photo-damage and photo-bleaching [204].

**FIG. 41:**
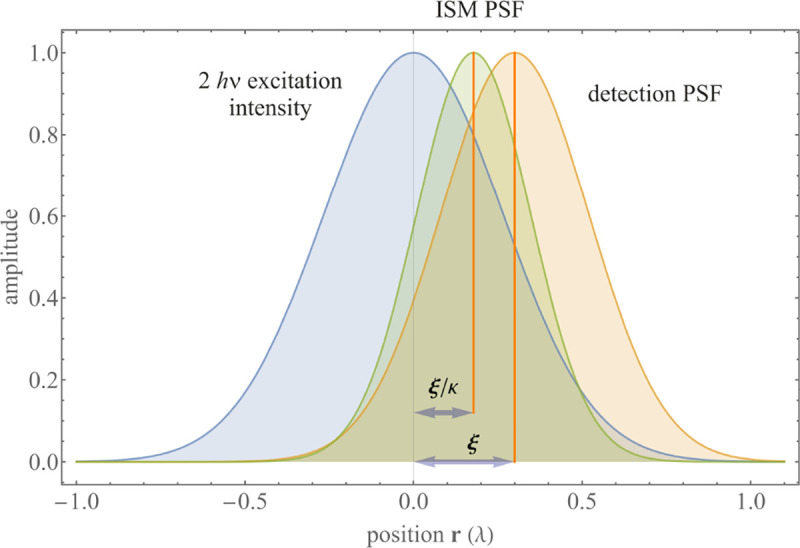
Pixel reassignment in two-photon excitation ISM. By contrast to the ISM in Fig. 35, the excitation intensity distribution (one-photon excitation PSF) in two-photon microscopy has a larger width due to the larger excitation wavelength.

To gain deeper insight into the best possible lateral resolution achievable by a two-photon excitation microscope, we consider two-photon excitation along with the ISM detection, *i.e.*, recording at each scan position a small image of the excited region and performing pixel reassignment to obtain the high resolution ISM image; see Sec. IV B 2. However, the pixel reassignment must take into account the more complicated wavelength relations and that excitation is a two-photon absorption process. Approximating the one-photon excitation PSF as well as the single pixel detection PSF once more by Gaussians with variances $\sigma_{ex}^{2}$ and $\sigma_{em}^{2}$ (see Sec. IV B 2), we can visualize the PSF of the scan image recorded by one pixel at position ξ on the array detector as shown in Fig. 41 (also see Eq. 116).

The new reassignment factor κ (see Sec. IV B 2) is found by looking at the product of detection PSF with square of one-photon excitation PSF, which yields a Gaussian distribution with variance $\sigma^{-2}=2\sigma_{ex}^{-2}+\sigma_{em}^{-2}$ and mid point position ξ/κ with

(121) $$\kappa =1+2{\left(\frac{\lambda_{em}}{\lambda_{ex}}\right)}^{2}$$

which would yield for the case $\lambda_{ex}=2\lambda_{em}$ the value κ=3/2; also see Eq. 117.

**FIG. 42:**
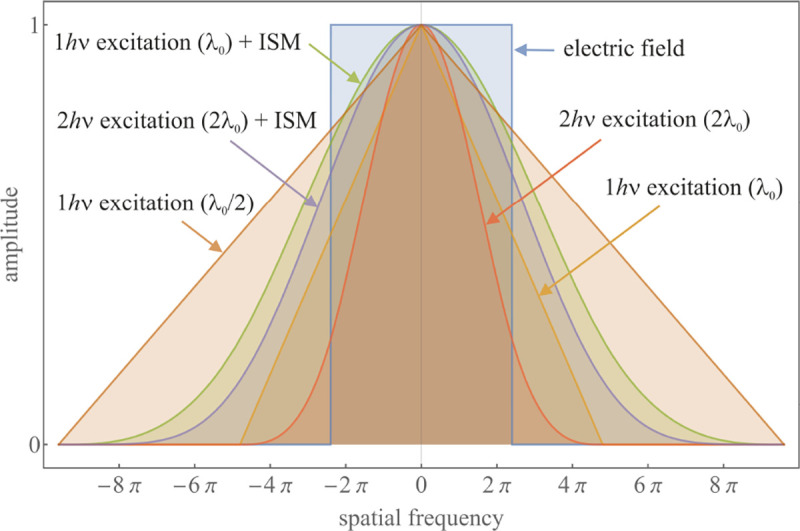
Comparison of one- and two-photon microscopy. For explanation see main text. For explanation see main text.

We now compare the performance of such a twophoton excitation ISM with that of a one-photon excitation CLSM and ISM at *half* the wavelength. For simplicity, we consider the toy model of a one-dimensional microscope. The Fourier representation of the excitation electric field of such a one-dimensional microscope is a uniform amplitude distribution over the frequency range supported by the microscope (maximum lateral frequency transmitted is ${nk}_{0}\;sin\Theta$). This is shown in Fig. 42 by the table-top function (electric field). The auto-convolution of this uniform amplitude distribution yields the excitation OTF and is, for the one-dimensional and one-photon case, the triangular function shown in Fig. 42 and denoted by “1hν excitation $\left(\lambda_{0}\right)$.”

The two-photon excitation PSF for an excitation with $2\lambda_{0}$ wavelength is given by the square of the one-photon excitation PSF. So its OTF corresponds to the autoconvolution of the one-photon OTF shown by “1hν excitation $\left(\lambda_{0}\right)$” in Fig. 42, but scaled down (along the frequency axis) by a factor of 2 (remember that we compare two-photon excitation at $2\lambda_{0}$ with one-photon excitation at $1\lambda_{0}$). The corresponding curve is denoted by “2hν excitation (${2\lambda }_{0}$)”. The OTFs for the extensions of one-photon and two-photon excitation fluorescence microscopy with ISM are also shown in the figure, together with the OTF of one-photon excitation at $\lambda_{0}/2$ for comparison.

As can be seen, the frequency support of two-photon excitation at $2\lambda_{0}$ wavelength is equal to that of the onephoton excitation at $\lambda_{0}$, but with increased amplitudes at low frequencies and decreased amplitudes at large frequencies. In other words, a two-photon microscope transmits high lateral spatial frequencies less efficiently than a one-photon microscope operating at half the wavelength. This is also true when we compare two-photon ISM with one-photon ISM, as shown by the two curves “1hν excitation $\left(\lambda_{0}\right)+\;\text{ISM}$” and “2hν excitation $\left(2\lambda_{0}\right)\;+\;ISM$” in Fig. 42. Both modes have a frequency support equal to that of one-photon excitation at $\lambda_{0}/2$, but with considerably damped amplitudes at high spatial frequencies, with one-photon ISM performing slightly better than two-photon ISM. Thus, two-photon (or multiphoton) excitation generally performs worse, in terms of resolution, than one-photon microscopes at half the wavelength, but their main advantage is that biological tissue is often more transparent and less scattering at long wavelengths, so that two- and multi-photon excitation microscopes allow deeper penetration depths and imaging of thicker tissue samples.

### C. Models for single spot confocal analysis

The point scanning microscope described above, namely confocal and two-photon microscopes, have been used to study both dynamic [205–208] and static [209–213] phenomena with both immobile [206–209, 214] as well as scanning [78, 205, 210, 215] spots under continuous or pulsed illumination [207, 216].

Point scanning microscopes, particularly confocal microscopes, are the basis for a variety of analysis methodologies including FLIM [79, 217], where photon arrival time statistics following pulsed excitation are collected and analyzed, and Fluorescence Correlation Spectroscopy (FCS) [205, 218, 219] where photon arrival times or fluorescence intensities, often collected under constant illumination, are correlated in time in order to extract dynamical parameters [207, 208].

**FIG. 43:**
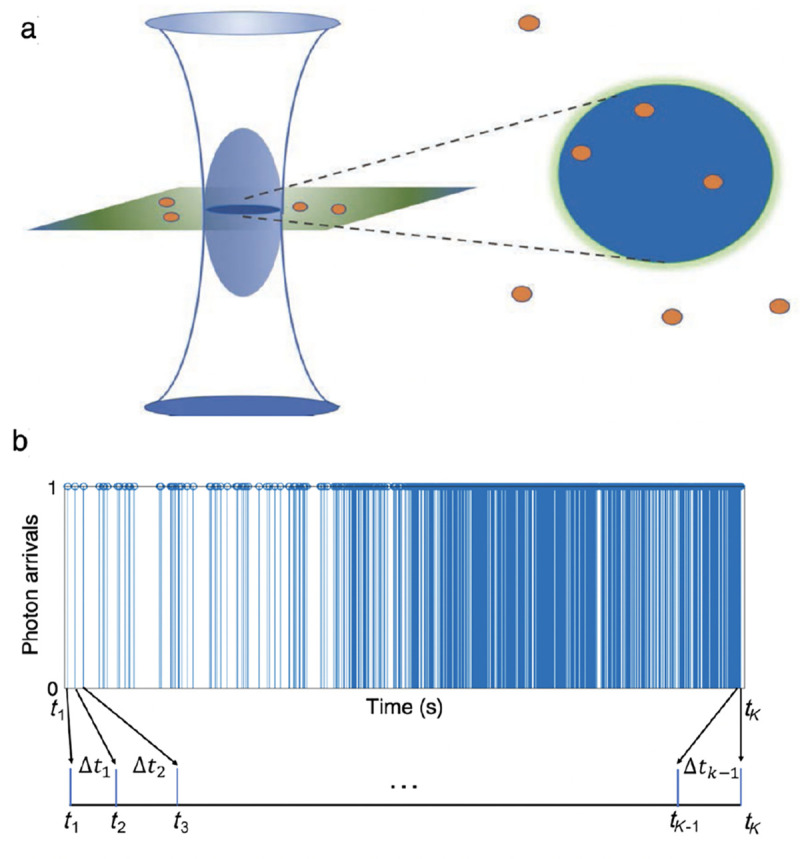
In (a) we show a schematic of confocal volume (in blue) with labeled molecules emitting photons in proportion to their degree of excitation (and thus from the center of) the confocal volume. In (b) we show a synthetic trace with 1500 photons generated assuming four molecules diffusing at 1*μ*m^2^*/s* for 30*ms* using background and molecule photon emission rates of 10^3^ photons/*s* and 4 × 10^4^ photons/*s*, respectively. Figure is adapted from Ref. [222].

Here, we begin with a description of FCS where a static confocal spot is often employed to determine the reaction kinetics and diffusion coefficient of particles freely diffusing through the spot; see Fig. 43a. In particular, this figure illustrates a scenario often analyzed using correlative analysis (FCS) data with labeled molecules freely diffusing through a static confocal spot becoming excited in proportion to the local light intensity. In traditional FCS analysis, a fraction of emitted photons are captured and dynamical properties obtained by autocorrelating in time the emitted light intensity or photon arrival times [218–221].

While auto-correlating photon arrivals is computationally informative, it is data inefficient and eliminates single molecule information already encoded in the signal [208, 222, 223]. Thus, a statistical method directly analyzing photon arrivals is warranted avoiding data post-processing including auto-correlation [207, 208, 222]. Here, we begin by deriving the likelihood for the collection of K photons whose inter-arrival intervals [222] are designated by ${\Delta t}_{1:K}\;=\;\{{\Delta t}_{1},...,{\Delta t}_{K}\}$, see Fig. 43b, under the assumption of continuous illumination.

We begin by considering the confocal PSF derived earlier in this section in Eq. 114 and, for simplicity, immediately adopt Cartesian coordinates where r=(ρ,z). For an arbitrary M molecules located at $r_{k}^{m}$ at time $t_{k}$, we write the following profile

(122) $$S_{k}\left(r\right)=\sum_{m=1}^{M}\delta \left(r-r_{k}^{m}\right).$$

As such, the total expected photon emission rate at time level $k,\;\mu_{k}$, follows from

(123) $$\mu_{k}\left(r\right)=\mu_{ℬ}+\mu_{0}\int \;drU_{\text{cf}}\left(r\right)S_{k}\left(r\right)=\mu_{ℬ}+\sum_{m=1}^{M}\mu_{k}^{m}\left(r_{k}^{m}\right),$$

where $\mu_{k}^{m}\left(r_{k}^{m}\right)=\mu_{0}U_{cf}\left(r_{k}^{m}\right)$ is the expected photon emission rate from the *m*th molecule located at $r_{k}^{m},\;\mu_{0}$ is the maximum photon detection rate associated with a molecule located at the PSF’s center, and $\mu_{ℬ}$ is the background photon emission rate.

Now the photon emission rate, $\mu_{k}$, determines the photon interval time, $\Delta t_{k}$, according to

(124) $$\Delta t_{k}∼Exponential\;\left(\mu_{k}\left(r\right)\right),$$

where we follow notation introduced in Sec. I B (for pulsed illumination see Appendix A). This exponential waiting time follows from the model that photon emission per unit time is Poisson distributed implying that photon inter-arrival times are exponentially distributed.

Finally, as the position, and thus the rate $\mu_{k}(r)$ are stochastic, under the assumption of a diffusion model with open boundary conditions where transitions between positions are normally distributed, we then write

(125) $$r_{k}^{m}|D∼\mathrm{Normal}\left(r_{k-1}^{m},2D\Delta t_{k}\right),$$

where D is the diffusion coefficient assumed to be constant across time and space.

We can now construct the likelihood for K photon inter-arrival times, $\Delta t_{1:K}$, given by Eq. 124. As the photon arrival times are *iid* (see Sec. I B), the likelihood of the trace is simply the product of the likelihood of every individual photon time interval

(126) $$P\left(\text{Δ}t_{1:K}|M,D,\bar{\bar{r}},\mu_{0},\mu_{ℬ}\right)\;\;=\prod_{k}\;\text{Exponential}\;\left(\text{Δ}t_{k};\mu_{k}\left(r\right)\right),$$

where $\mu_{k}(r)$ is an implicit function of $M,\;D,\;\mu_{0}$ and $\mu_{ℬ}$; see Eqs. 123 and 125. Moreover, overbars represent the set of all possible values for m at all time levels $t_{k}$.

**FIG. 44:**
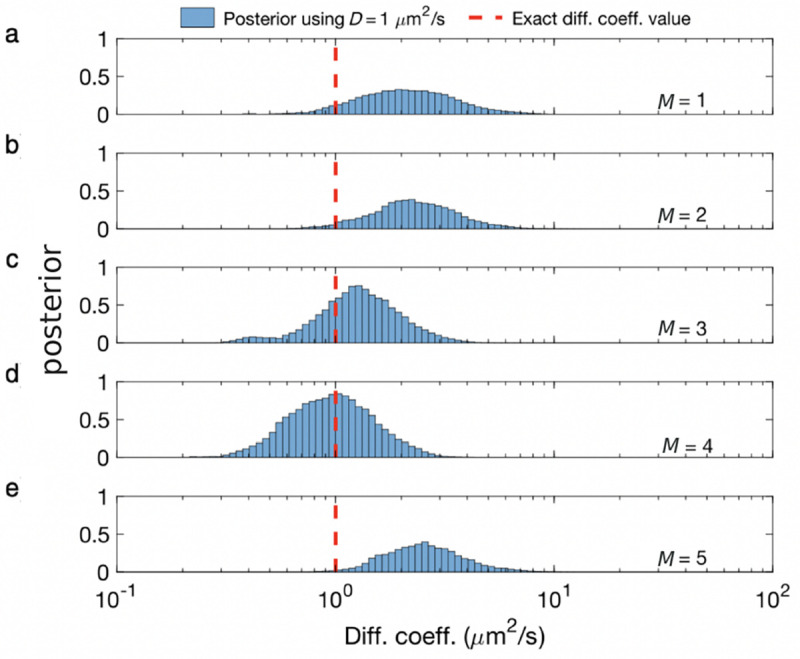
Here we show how strongly posteriors over diffusion coefficients depend on the pre-specified M when operating within a parametric Bayesian paradigm. The trace analyzed contains ≈1800 photons generated from 4 molecules diffusing at $D=1\mu m^{2}/s$ for 30 *ms* with a background and maximum molecule photon emission rate of f 10^3^ and 4 × 10^4^ photons/s, respectively. To deduce the diffusion coefficient within the parametric paradigm, we assumed a fixed number of molecules: (a) M=1; (b) M=2; (c) M=3; (d) M=4; and (e) M=5. The correct estimate in panel d–and the mismatch in all others-highlights why we must use the available photons to simultanesouly learn the number of molecules and diffusion coefficient. Figure is adapted from Ref. [222].

To maximize the likelihood we would need to determine the number of molecules either in advance, *i.e.*, parametric model, or work in a non-parametric paradigm and infer the number of molecules alongside the other parameters. The likelihood above cannot naively be maximized to obtain parameters due to the classical over-fitting favoring more complex models, *i.e.*, larger number of diffusing molecules. However, in the former case, assuming a wrong parametric model with M molecules [208, 222] can result in incorrect estimates of other parameters, *e.g*, diffusion coefficient; see Fig. 44.

As such, we abandon the parametric paradigm and start leveraging tools from BNPs [29, 224, 225]. Of particular interest within the BNP paradigm is the beta-Bernoulli process prior (see Sec. I B) allowing for the inclusion of priors on the number of candidate molecules, M, that we formally allow to tend to infinity, M→∞, prior to considering data via the likelihood. Put differently, each molecule is treated as a Bernoulli random variable (a load), $b_{m}$, learned simultaneously along with the other unknowns; see Sec. I B. The probability of the load being recovered as one, equivalently the probability of the molecule being warranted by the data, is the single parameter of the Bernoulli distribution on which we place a beta prior.

Within this framework, Eq. 123 is modified by replacing $\mu_{k}^{m}$ by $b_{m}\mu_{k}^{m}$ and summing over infinite molecules. As a result, the likelihood adopts the following form

(127) $$P\left(\text{Δ}t_{1:K}|\overset{-}{b},D,\bar{\bar{r}},\mu_{0},\mu_{ℬ}\right)\;\;=\prod_{k}\;\text{Exponential}\;\left(\text{Δ}t_{k};\mu_{k}\left(r\right)\right).$$

Our non-parametric posterior is, therefore, proportional to the likelihood in Eq. 127 and Bernoulli priors on each of the molecules as further detailed in box IV C.

**FIG. 45:**
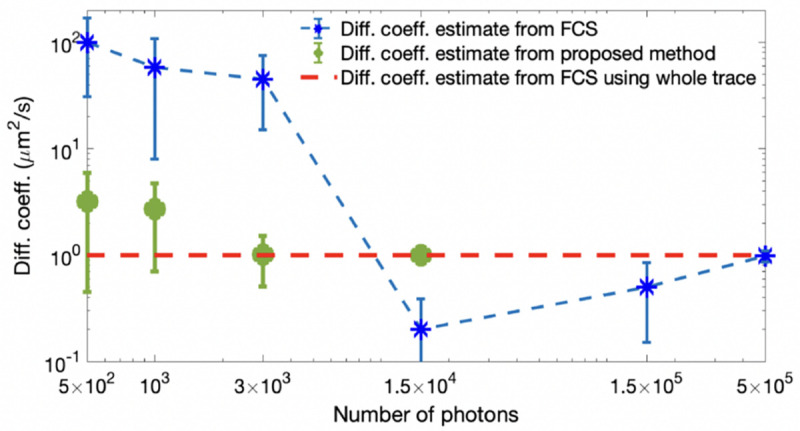
Comparison of diffusion coefficients obtained from the statistical framework and correlative (FCS) methods plotted versus photon numbers used in the analysis. Photon arrival times were simulated using the parameter values listed in Fig. 43b. Figure is adapted from Ref. [222].

Now equipped with the posterior, we draw samples using Monte Carlo methods to learn the set of unknowns ϑ. To learn the trajectories $\bar{\bar{r}}$, we use the forward filtering backward sampling [22, 135, 222], while the rest of parameters can be sampled either directly or using Metropolis-Hasting; see Sec. I B. Fig. 45 benchmarks the diffusion coefficient learned against photon counts and compares the results to correlative techniques.

While the above approach returns a trajectory, the photon emission rate of Eq. 123 and thus the likelihood given by Eq. 127 are invariant under transformationsleaving $\sqrt{{\left(\rho /\sigma_{\rho }\right)}^{2}+{\left(z/\sigma_{z}\right)}^{2}}$ unchanged with respect to the center of the confocal spot, *e.g.*, z to -z. As such, equivalent positions would lead to the same likelihood on account of the symmetry in the confocal PSF, Eq. 114. Therefore molecule locations cannot be uniquely determined using a single confocal setup.

**FIG. 46:**
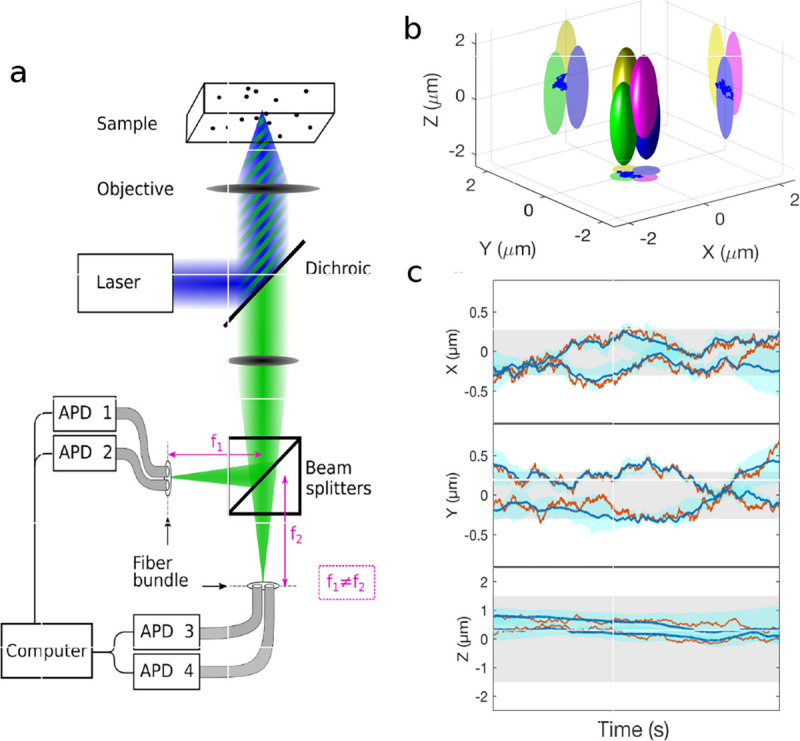
(a) Multi-focal setup. Here, a beam splitter is used to divide the fluorescent emission (designated by green) into two paths which are later coupled into fibers and detected by 4 APDs corresponding to different focal spots. (b) PSFs associated to different light paths. (c) Trajectories for two freely diffusing molecules with $D=1\mu m^{2}/s,\;\mu_{0}=5\times 10^{4}$ photons/s and $\mu_{ℬ}=10^{3}$ photons/s. Here, the orange and blue curves represent the ground truth and median of the learned trajectories. The blue and gray areas, respectively, denote the 95 percent confidence intervals and the width of the PSF. Figure is adapted from Ref. [223].

By contrast, it is possible to determine absolute molecular locations (trajectories) by breaking the spatial symmetry of the confocal spot in considering a multi-focus confocal setup [223, 226, 227]. Such a setup splits the confocal spot by introducing 4 detectors whose detection volume is offset with respect to one another; see Fig. 46a–b. Photons from molecules in such a setup are detected in the *l*th detector with the following rate

(128) $$\mu_{k}^{l}\left(r\right)=\mu_{ℬ}^{l}+\mu_{0}\sum_{m}b_{m}^{l}U_{\text{cf}}^{l}\left(r_{k}^{m}\right)$$

at time k; see Eq. 123. The total photon detection rate is, in turn, the sum of detection rates from all different detectors $\mu_{k}=\sum_{l}\;\mu_{k}^{l}$. Finally, following the same logic as before in constructing the single confocal spot likelihood, we arrive at a likelihood model similar to Eq. 127.

The likelihood derived can then be employed, in conjunction with appropriate priors over unknowns similar to the single spot case (see Box IV C), to construct a posterior. This posterior can then be sampled to learn absolute molecular trajectories; see Fig. 46c.

It is now conceivable to imagine generalizing the treatment above to include multiple diffusing species [228], species with donor and acceptor labels (FCS -FRET) [229, 230], species undergoing reactions which alter their emission rate and kinetics [231].

This brings us to a list of advantages of the statistical approach above as compared to traditional FCS. It is clear such methods are more data efficient, we can deal with any PSF shape, and optical aberrations [232, 233]. But also, fundamentally, by avoiding data post-processing they can learn more. For instance, in contrst to FCS, the statistical methods described above can learn properties of every individual molecule diffusing through the spot providing single molecule resolution albeit at some computational cost.

Having dealt with continuous illumination, we now turn to pulsed illumination and, for simplicity, assume an immobile sample. Under pulsed illumination, the data acquired is a trace of K photon arrival times, $\Delta t_{1:K}$, reported with respect to the immediate preceding pulses. These arrival times are also termed micro-times and encode the excited state lifetimes, $\tau_{m}$ for the *m*th species, of fluorophore species (see Sec. II) present within the confocal spot and the associated photon ratios (weights) shown by $\pi_{m}$ for the *m*th species related to fluorophore densities as we will show later.

Although heuristics exist to determine the lifetimes within a given data set [78], similar to the example we saw in Fig. 45 where the number of molecules introduced by hand dictated the diffusion coefficient recovered, here we encounter a similar problem. To be more precise, the lifetimes we deduce from the data strongly depend on how many lifetime species are assumed to be present in the data [234]. Indeed, existing techniques cannot simultaneously decode: 1) the number of fluorophore species (lifetime components) present in a trace of photon arrival times; 2) operate on a broad range of lifetimes below the Instrument Response Function (IRF) (see Sec. A) to comparable to the laser inter-pulse times and similar lifetimes; 3) provide uncertainties over parameter estimates; 4) inferring smooth fluorophore densities, *i.e.*, lifetime maps given by $\Omega_{m}(r)=\mu_{m}S_{m}(r)$ where $S_{m}$ and $\mu_{m}$ are, respectively, the fluorophore densities (see Eq. 122) and fluorophore excitation probability (for in-focus fluorophores) during a laser pulse for *m*th species, below data pixel size. Here, we review statistical frameworks for FLIM data analysis to address issues above using minimal photon budgets. In doing so, we first discuss a framework for a single pixel (a single confocal spot) and then generalize it to wide-field FLIM with multiple pixels to deduce lifetime maps below the pixel size.

We begin by introducing the likelihood for data set $\Delta t_{1:K}$ collected from a single spot containing M species

(129) $$P\left(\text{Δ}t_{1:K}|\lambda_{1:M},\pi_{1:M}\right)=\prod_{k=1}^{K}P\left(\text{Δ}t_{k}|\lambda_{1:M},\pi_{1:M}\right)$$

where $\lambda_{m}$ denotes the inverse of lifetime $\tau_{m}=1/\lambda_{m}.\;P\left(\Delta t_{k}|\lambda_{1:M},\pi_{1:M}\right)$ is the likelihood of the *k*th arrival time. To derive this likelihood, we must consider the IRF and sum over all possibilities that could give rise to this photon including: all M fluorophore species; and all $N_{pl}$ previous laser pulses. Assuming a Gaussian IRF, this leads to (see Appendix A and Eq. A23) [215, 234]

(130) $$P\left(\text{Δ}t_{k}|\lambda_{1:M},\pi_{1:M}\right)=[\sum_{m=1}^{M}\pi_{m}\sum_{n=0}^{N_{pl}}\frac{\lambda_{m}}{2}\;\;\times \exp\left(\frac{\lambda_{m}}{2}\left(2\left(\tau_{\text{IRF}}-\text{Δ}t_{k}-nT\right)+\lambda_{m}\sigma_{\text{IRF}}^{2}\right)\right)\;\;\times \text{e}rfc\left(\frac{\tau_{\text{IRF}}-\text{Δ}t_{k}-nT+\lambda_{m}\sigma_{\text{IRF}}^{2}}{\sigma_{\text{IRF}}\sqrt{2}}\right)],$$

where $\tau_{IRF},\;\sigma_{IRF}^{2}$ and T, respectively, denote the IRF offset and variance, and the inter-pulse time; see Appendix A. Here, if we ignore excitation by previous pulses, we arrive at the likelihood obtained in Ref. [235].

To summarize, parametrically, we would pre-specify M and often set it to either one or two for simplicity similar to Refs. [235, 236]. In contrast, non-parametrically the number of lifetime components are treated in the same manner as the other parameters [215, 234].

**FIG. 47:**
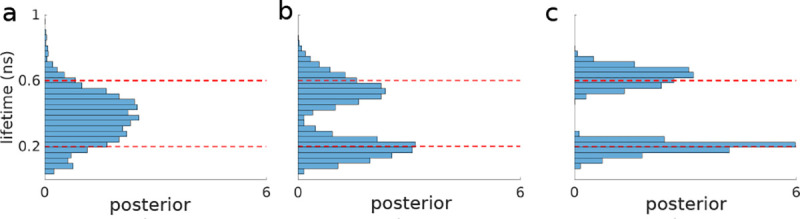
Lifetime histograms from single-pixel FLIM. Here, lifetimes are below IRF and with sub-nanosecond difference. Data sets used in panels (a-c) were simulated with 500, 1K, 2K photons, IRF width of 0.66 ns and ground truth lifetimes of 0.2 ns and 0.6 ns denoted by dotted lines. The correct number of lifetimes are learned when using more than 500 photons in panels (b-c).

In the non-parametric paradigm, the single pixel FLIM posterior is proportional to the likelihood Eq. 130 and the priors over the unknown parameters, namely $\lambda_{1:M}$ and $\pi_{1:M}$. For $\lambda_{m}$, we use a Gamma prior to guarantee nonnegative values. For $\pi_{m}$, we leverage the Dirichlet process prior from the BNP paradigm [25–27] to facilitate inference over the probability in the number of species present warranted by the data, *i.e.*, to address model selection; see Sec. I B. Within this framework, as before when operating non-parametrically, we assume an *a priori* infinite number of species (M→∞) with weights $\left(\pi_{m}\right)$ associated to them. As we sample these weights, the weights ascribed to species not contributing to the data attain negligible values. Fig. 47 shows lifetime histograms for two lifetimes below the IRF and with sub-nanosecond differences using 500, 1K and 2K photons.

**FIG. 48:**
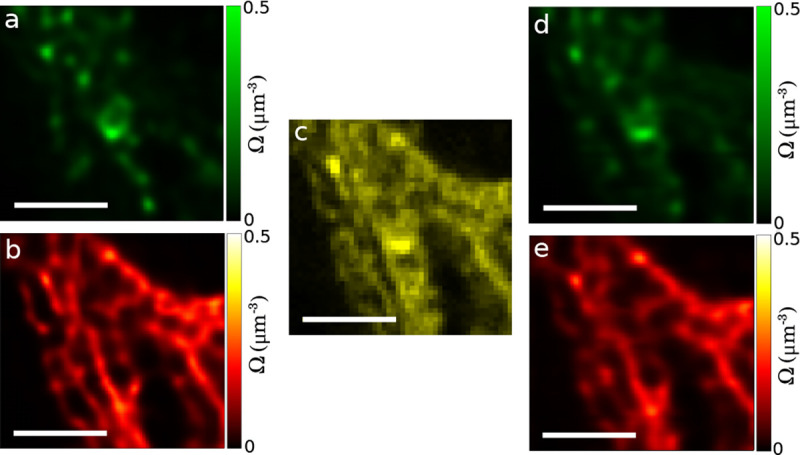
Experimental FLIM data from mixture of two cellular structures (lysosome and mitochondria shown in green and red, respectively) stained with two different fluorophore species. (a-b) Ground truth lifetime maps. (c) Data acquired from mixtures of two ground truth maps. (d-e) Resulting sub-pixel interpolated lifetime maps obtained using the proposed statistical framework. The average absolute difference of the ground truth and learned maps is 4%. Scale bars are 4μm. Figure is adapted from Ref. [215].

After reviewing the framework for single pixel FLIM, we turn to multi-pixel FLIM estimating smooth lifetime maps from limited confocal scanning data; see Fig. 48.

One naive way to process data from such an experiment is analyzing each pixel independently using the above framework. However, this would lead to pixelated lifetime maps. In what follows, we review a framework for multi-pixel FLIM [215, 237] capable of reporting lifetime maps below the data pixel size leveraging spatial correlations across pixels by invoking GPs from the BNP paradigm; see Sec. I B and Fig. 48.

Data from multi-pixel FLIM experiments are typically collected by scanning, spot-by-spot, over the sample using a CLSM setup. Data from such experiments consists of a set of photon arrival times collected over all pixels designated by $\bar{\bar{\Delta t}}$. Another observation that we consider here is whether a laser pulse leads to a photon detection or not designated by a binary variable ${𝒲}_{k_{p}}^{i}$ for the $k_{p}$ th pulse and *i*th pixel. The likelihood for such data is given by

(131) $$P\left(\bar{\bar{𝒲}},\bar{\bar{\text{Δ}t}}|\vartheta \right)=\prod_{i}\prod_{k_{p}}P\left({𝒲}_{k_{p}}^{i}|\vartheta \right)P\left(\text{Δ}t_{k_{p}}^{i}|\vartheta \right),$$

where ϑ collects all the unknowns including inverse of lifetimes $\lambda_{1:M}$, multi-pixel lifetime maps $\Omega_{1:M}$, the loads $b_{1:M}$, and hyper-parameters $\nu_{1:M}$ over each species. Here, the likelihood associated with photon arrival times is similar to Eq. 130 and given by

(132) $$P\left(\text{Δ}t_{k_{p}}^{i}|\vartheta \right)=[\sum_{m=1}^{M}\pi_{m}\sum_{n=0}^{N_{pl}}\frac{\lambda_{m}}{2}\;\;\times \exp\left(\frac{\lambda_{m}}{2}\left(2\left(\tau_{\text{IRF}}-\text{Δ}t_{k_{p}}^{i}-nT\right)+\lambda_{m}\sigma_{\text{IRF}}^{2}\right)\right){\;\;\times \text{erfc}\left(\frac{\tau_{\text{IRF}}-\text{Δ}t_{k_{p}}^{i}-nT+\lambda_{m}\sigma_{\text{IRF}}^{2}}{\sigma_{\text{IRF}}\sqrt{2}}\right)]}^{{𝒲}_{k_{p}}^{i}},$$

which reduces to one for pulses that do not lead to any photon detection (empty pulses with ${𝒲}_{k_{p}}^{i}=0$).

Here, the weights, $\pi_{1:M}$, are directly related to the lifetime maps by [215]

(133) $$\pi_{m}^{i}=\left(1-P_{0m}^{i}\right)\prod_{q\neq m}P_{0q}^{i},$$

where $P_{0m}^{i}$ represents the probability of no photon detection within the *i*th pixel from the *m*th species given by

(134) $$P_{0m}^{i}=exp\left[-b_{m}\int \;\Omega_{m}(r)U_{cf}\left(\xi^{i}-r\right)dr\right],$$

where $\xi^{i}$ is center of the ith pixel. Moreover, $b_{m}$ denotes the loads associated to the *m*th lifetime map as introduced earlier in Sec. I B which we will later learn leveraging the beta-Bernoulli process prior (see box IV C) to deduce the number of lifetime maps present within the data. As a sanity check, we note that for species with $b_{m}=0$, the probability of no photon detection is one.

After illustrating the photon arrival times, we proceed to consider the binary observation ${𝒲}_{k_{p}}^{i}$. This parameter has a Bernoulli distribution with a success probability of $1-\pi_{0}^{i}$

(135) $${𝒲}_{k_{p}}^{n}∼\mathrm{Bernoulli}\left(1-\pi_{0}^{i}\right).$$

Here, $\pi_{0}^{i}$ is the probability of no photon detection from the *i*th pixel given by $\pi_{0}^{i}=\prod_{m=1}^{M}\;P_{0m}^{i}$.

After introducing the likelihoods, we construct the posterior proportional to the product of the likelihood and priors over unknown parameters. The most notable priors are: priors over continuous lifetime maps for which we invoke a GP prior; and the prior over the loads for which we use the beta-Bernoulli process prior both from the BNP paradigm leading to a doubly non-parametric framework; see Sec. I B. The GP priors over lifetime maps are comprised of an infinite set of correlated random variables, *i.e.*, the value of the map at every point in space

(136) $$\Omega_{m}∼GP\left(\nu_{m},K\right)$$

where K and $\nu_{m}$ denote the correlation kernel (also known as covariance matrix) and the mean of the GP prior. The remaining priors are either physically or computationally motivated, see box IV C.

Now with the posterior at hand, we can make inferences about the parameters ϑ by taking samples from the posterior using Monte Carlo techniques. Particularly, as there are two non-parametric priors associated to lifetime maps, *i.e.*, GP prior and beta-Bernoulli process prior, we use elliptical slice sampling [21] to sample the lifetime maps.

### D. Structured illumination microscope

As discussed in Sec. III C, a major drawback of widefield fluorescence imaging is the lack of optical sectioning due to the missing cone of the OTF disallowing axial resolution below certain spatial frequencies; see Fig. 13. This, in turn, leads to contribution of out-of-focus blur resulting in final image degradation. In previous sections, we discussed near-field and point scanning methods where, for example, a conventional confocal microscope achieves optical sectioning using a pinhole to block out-of-focus blur; see Sec. IV B 1. Here, we discuss how SIM achieves optical sectioning and higher resolution [238–242].

An early effort toward attaining optical sectioning introduced patterned illumination with a high stripe contrast only near the focal plane [243]. Thus, the illumination intensity in the focal plane varies strongly across space. This is by contrast to illumination away from the focal plane where it becomes more homogeneous. The pattern is then translated twice yielding three images ${ℐ}_{l}$ with corresponding phase offsets $\phi_{l},\;l=0:2$. One way to attain optical sectioning is to create three images from differences in two images, say $\Delta {ℐ}_{ll^{\prime }}(r)={ℐ}_{l}(r)-{ℐ}_{l^{\prime }}(r)$, and then combining them according to (also see Ref. [244])

(137) $$\Lambda_{sec}(r)\;=\sqrt{\Delta {ℐ}_{01}(r{)}^{2}+\Delta {ℐ}_{12}(r{)}^{2}+\Delta {ℐ}_{20}(r{)}^{2}},\;\;\phi_{l}\;=\frac{2l\pi }{3},l=0:2.$$

In doing so, subtracting images cancels the unmodulated out-of-focus components as they are approximately homogeneously illuminated.

These early efforts ultimately motivated structured illumination to achieve higher resolution [241, 242] that we now discuss.

We begin by considering SIM image formation where SIM images are mathematically given by the product of the fluorophores’ distribution, S(r) (see Sec. IV C), and the illumination intensity pattern, $I_{ex}(r)$, followed by convolution with the microscope’s wide-field detection PSF (also see Eq. 62)

(138) $$\Lambda (r)=ℬ+I\left[S(r)I_{ex}(r)\right]⊗U_{wf}(r)$$

where $U_{wf}(r)$ and I are, respectively, PSF of the widefield setup, (*e.g.*, see Sec. III E) and fluorophore brightness per frame. Here, ℬ is background due to out-of-focus fluorescent features, which we ignore for simplicity here.

**FIG. 49:**
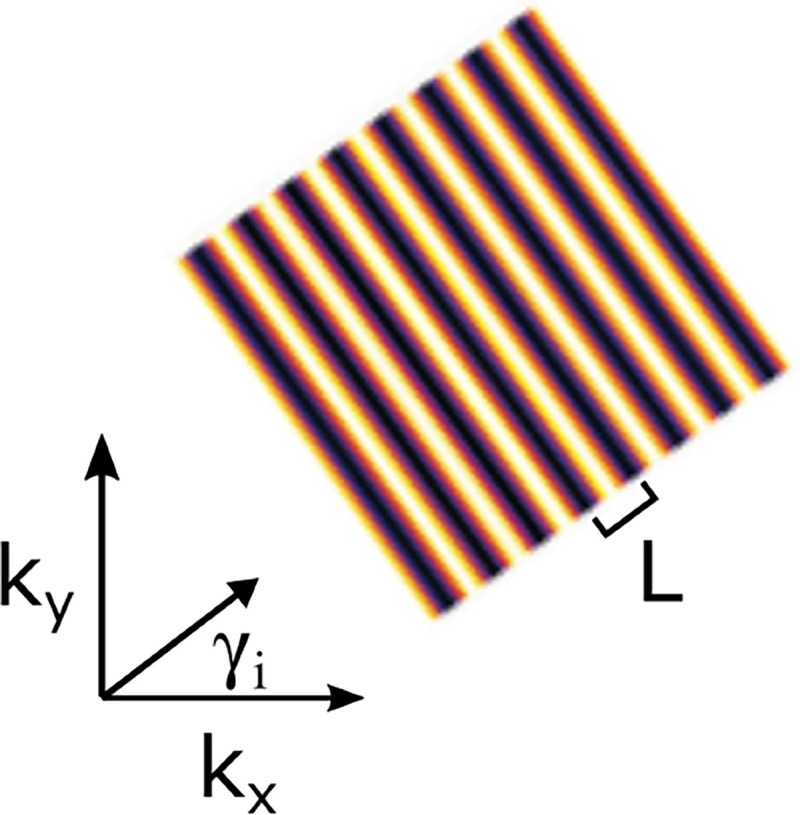
Sinusoidal illumination pattern for SIM microscopy. Here, $k_{i}$ is the wave vector, L is the fringe spacing, $\gamma_{i}$ is the in-plane angle of the illumination. The phase is related to the position of the maxima relative to the optical axis.

While various modulated illumination patterns are conceivable for SIM [245–247], in practice, the sample is typically illuminated using a sinusoidal intensity, $I_{ex}(r)$, with different in-plane phases and angles (see Fig. 49) achieved by either interference-based [248, 249] or laserscanning [250–252] methods. Under the former method, such intensity pattern can be generated by the interference of two to three laser beams, and rotation and translation of a grating embedded within the setup’s illumination arm. For two beam interference, the image formation is described by

(139) $$\Lambda^{li}(r)\;=I[S(r)\frac{1}{2}\left(1+Mcos\left(r\cdot k_{i}+\phi_{l}\right)\right)]⊗U_{wf}\left(r\right),\;\;\gamma_{i}\;=arctan\frac{k_{x}^{i}}{k_{y}^{i}},L=2\pi /\sqrt{k_{x}^{i^{2}}+k_{y}^{i^{2}}}$$

where M is the modulation depth assumes to be one in subsequent calculations for simplicity. $k_{i}$ is the wave vector, with components $k_{x}^{i}$ and $k_{y}^{i}$, defining the period of the oscillating pattern, *i.e.*, the fringe spacing denoted by L; see Fig. 49 Here, $\gamma_{i}$ and $\phi_{l}$ are, respectively, the *l*-th in-plane angle of the illumination and the *i*th phase offset determining the position of the maxima relative to the optical axis; see Fig. 49 and Eq. 137.

**FIG. 50:**
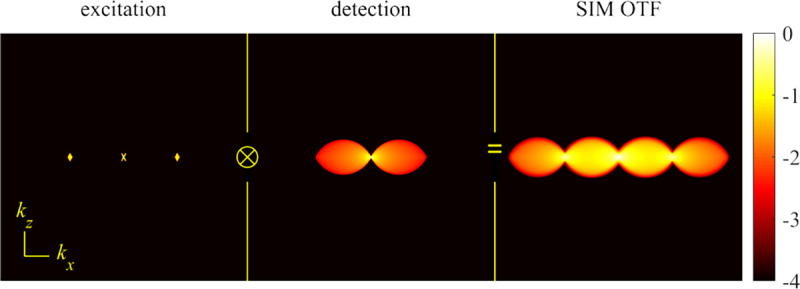
SIM OTF. The left and middle panels, respectively, show the Fourier transforms of the modulated illumination intensity (SIM excitation OTF) and the widefield detection. The right panel shows the SIM OTF given by mapping the wide-field detection OTF (middle panel) on the three positions given by the three deltapeaks of the Foruier representation of the SIM excitation (left panel), see also Eq. 140.

Here, the improved resolution is accomplished by exploiting the moiré effect (frequency mixing) between the excitation pattern and the sample’s spatial frequencies. That is, the previously unobservable high frequency information of the sample, beyond the support of the widefield OTF, is shifted down into the microscope’s passband; see Fig. 21 and 50.

The effect of the structured illumination is most intuitively demonstrated in Fourier space. For the sinusoidal pattern given in Eq. 139, its Fourier representation reads

(140) $${\tilde{\Lambda }}^{li}(k)=I[\tilde{S}(k)⊗{\tilde{I}}_{ex}(k)]{OTF}_{wf}(k)\;\;=I[\tilde{S}(k)⊗(\delta (0)+\frac{1}{2}e^{+i\phi_{l}}\delta \left(k+k_{i}\right)\;\;+\frac{1}{2}e^{-i\phi_{l}}\delta \left(k-k_{i}\right))]{OTF}_{wf}(k)\;\;=I[\tilde{S}(k)+\frac{1}{2}e^{+i\phi_{l}}\tilde{S}\left(k+k_{i}\right)\;\;+\frac{1}{2}e^{-i\phi_{l}}\tilde{S}\left(k-k_{i}\right)]{OTF}_{wf}(k)\;\;={\tilde{ℐ}}_{0}(k)+\frac{1}{2}e^{+i\phi_{l}}{\tilde{ℐ}}_{+}\left(k+k_{i}\right)+\frac{1}{2}e^{-i\phi_{l}}{\tilde{ℐ}}_{-}\left(k-k_{i}\right),$$

where ${OTF}_{wf}(k)$ denotes the wide-field OTF (see Fig. 21 and middle panel of Fig. 50, and the sinusoidal illumination pattern (for a given angle and phase) is described by three different frequencies in the Fourier domain (see the left panel in Fig. 50 yielding the three SIM harmonics ${\tilde{ℐ}}_{0},\;{\tilde{ℐ}}_{+},\;{\tilde{ℐ}}_{-}$. Here, the first delta function within the parenthesis coincides with the Fourier representation of the uniform (wide-field) illumination. However, the two subsequent terms result from the sinusoidal illumination. These additional terms are two copies of the Fourier representation of the sample $\tilde{S}(k)$ phase shifted by a factor $\phi_{l}$ and frequency shifted by $k_{i}$, providing extra information compared to wide-field microscopy.

Now, if we suppose the OTF cut-off frequency is $k_{c}$, we see that the frequency shifted components contain high frequency information not present in the central component (sum frequency $k+k_{i}$ and difference frequency $k-k_{i}$ at each sample frequency of k). When imaged, only frequencies inside the support of the wide-field OTF are captured. However, sample information from different (higher) frequency regions now lie within the microscope’s pass-band; see Fig. 50.

While the three SIM harmonics ${\tilde{ℐ}}_{0},\;{\tilde{ℐ}}_{+},\;{\tilde{ℐ}}_{-}$ (wide-field and ± pattern wave vector) already contain frequencies beyond wide-field pass-band, no sub-diffraction resolution can yet be achieved. This is because these components are overlapping in frequency space. In order to unmix the overlapping parts, we need to acquire at least three images with different pattern phases $\phi_{l}$ designated by ${\tilde{\Lambda }}^{li}(k)$ in Fourier space. The relation between the three SIM harmonics and these images is best shown in matrix form

$$\left[\begin{array}{c} {\tilde{\Lambda }}^{0i}(k)\\ {\tilde{\Lambda }}^{1i}(k)\\ {\tilde{\Lambda }}^{2i}(k) \end{array}\right]=\left(\begin{array}{ccc} 1 & 0.5e^{i\phi_{0}} & 0.5e^{-i\phi_{0}}\\ 1 & 0.5e^{i\phi_{1}} & 0.5e^{-i\phi_{1}}\\ 1 & 0.5e^{i\phi_{2}} & 0.5e^{-i\phi_{2}} \end{array}\right)\left[\begin{array}{c} {\tilde{ℐ}}_{0}(k)\\ {\tilde{ℐ}}_{+}\left(k+k_{i}\right)\\ {\tilde{ℐ}}_{-}\left(k-k_{i}\right) \end{array}\right].$$

Here, we used an idealized mixing matrix, with different phases for the available spectra evenly spaced between 0 and 2π. This allows us to solve for ${\tilde{ℐ}}_{0},\;{\tilde{ℐ}}_{+}$ and ${\tilde{ℐ}}_{-}$, *i.e.*, unmixing the SIM harmonics. The unmixed components are then recombined by shifting them so that their true zero frequency is aligned with their zero frequency in Fourier space, *i.e.*, $k_{0}$ setting. This yields an effective OTF extended to frequencies beyond the original OTF’s support and thus obtain SIM images, *i.e.*, the fluorophores’ density S(r), with high resolution [253, 254].

Several techniques have been put forward that mostly operate within the Fourier domain to unmix the SIM harmonics and reconstruct SIM images [247, 253–264] with multiple practical considerations in SIM image reconstruction. Ideally, we must know properties of the imaging system such as the exact OTF, pattern frequency, phases, and modulation depth (*e.g.*, see Eq. 139). Inaccurate properties can result in imperfect SIM reconstructions typically exhibiting well-known artifacts [265]. For instance, refractive index mismatch inducing property changes may lead to repeated features along the z axis known as “ghosting”; fine hexagonal “honeycomb” pseudo-structures can arise due to neglecting the background ℬ in Eq. 138 in 2D SIM images; a false $k_{0}$ setting impacting the OTF leads to so-called “hatching”, *i.e.*, the appearance of angle-specific stripes in one or more directions, to name only a few.

Here, to avoid artifacts exclusively arising when working within the Fourier domain, such as the $k_{0}$ setting, we review a method directly reconstructing SIM images within the spatial domain while rigorously propagating uncertainties [31]. The total likelihood is the product of likelihood models corresponding to each phase $\phi_{l}$ and wave vector $k_{i}$

(141) $$P\left({\bar{\bar{w}}}_{1:N}|{\bar{\bar{\text{Λ}}}}_{1:N}\right)=\prod_{i=1}^{3}\prod_{l=1}^{3}\prod_{n=1}^{N}P\left(w_{n}^{li}|{\text{Λ}}_{n}^{li}\right),$$

where overbars represent all possible values of i and l and where $P(w_{n}^{li}|\Lambda_{n}^{li})$ is the likelihood over a single pixel. Here, $w_{n}^{li}$ and $\Lambda_{n}^{li}$, respectively, denote the observed (data) and expected photon counts over the *n*th pixel using an illumination with phase $\phi_{l}$ and wave vector $k_{i}$. The expected photon count is given by (see Eqs. 4–5)

(142) $$\Lambda_{n}^{li}=\iint_{{𝒜}_{n}}\;dxdy\Lambda^{li}\left(r\right),$$

where $\Lambda^{li}(r)$ is given by Eq. 139 and ${𝒜}_{n}$ is the pixel area. Assuming high SNR and a Charged Coupled Devices (CCD) camera noise model of Eq. A12 we arrive at the following single pixel likelihood

(143) $$P(w_{n}^{li}|\Lambda_{n}^{li})=Gaussian(w_{n}^{li};g\Lambda_{n}^{li}+o,\sigma_{w}^{2}),$$

where g,o and $\sigma_{w}^{2}$, respectively, are the camera gain, offset and read-out variance; see Appendix A.

Finally, for a rigorous propagation of noise sources present within the problem, we highlight a Bayesian framework [31]. Within this framework, we consider priors over unknowns including the GP priors (Sec. I B) over the fluorophore distribution, S(r), and a prior over the GP’s covariance kernel, ν. These parameters are collectively re-grouped under ϑ={S(r),ν}. The complete framework is described in the following box.

Finally, we numerically sample the posterior to learn the unknowns ϑ. The sampling procedure is particularly straightforward for this SIM framework as the Gaussian likelihood and GP priors are conjugate resulting in a closed form posterior. For low SNR, this procedure fails as it leads to negative values for the fluorophore distributions allowed by the GP prior.

The SIM experiment described combined with image reconstruction typically achieves resolutions up to approximately 100 nm. This is because, in practice, the illumination pattern is also diffraction-limited implying that its corresponding Fourier peaks lie within the support of the system’s wide-field OTF, limiting the resolution improvement to a factor of about two (not considering, *e.g.*, the Stokes shift of fluorescence emission; see Sec. II). The resolution of the SIM image is then approximately $2\pi /\left(k_{c}+k_{i}\right)$ along the direction of $k_{i}$; see Eqs. 67 and 84–85. The process has to be repeated for at least three orientations $\left(k_{i},i=1:3\right)$ to achieve near isotropic lateral resolution enhancement.

Resolution improvement using structured illumination can also be combined with illumination modalities other than wide-field epi-fluorescence providing optical sectioning, such as TIRF [266], grazing incidence illumination [267], or light-sheet microscopy [268–270].

While the above discussion was focused on 2D SIM, here, we continue to outline the principle behind 3D SIM achieved using three (or more) interfering beams to generate an illumination pattern varying both laterally and axially [248, 271, 272]. For three-beam interference, five phase shifts are necessary to unambiguously unmix the frequencies, resulting in five SIM harmonics for each orientation $\left(k_{i}\right)$ as opposed to three for 2D SIM; see Eq. 140. These SIM harmonics need to be determined for each zposition and lateral pattern orientation. Similar to 2D SIM, to achieve isotropic resolution imaging, three different orientations are required for each of the three angles and five pattern phases leading to 15 SIM harmonics. Therefore, to unmix these components 15 images are needed. Although more complicated than 2D SIM, 3D SIM achieves approximately twofold resolution improvement as well as optical sectioning. This is because the OTF copies in 3D SIM overlap and fill the missing cone of the wide-field OTF; see Fig. 13.

All implementations of SIM mentioned so far use linear fluorescence excitation. This has the advantage of being relatively gentle to living samples because low excitation intensities can be used compared to other super-resolution imaging methods employing non-linear response of fluorophores to excitation light; see Secs. II and V. While the SIM resolution improvement is restricted to approximately twofold as the illumination pattern itself is limited by diffraction, a higher resolution is achievable combining SIM with non-linear fluorophore photo-physics [273, 274]; see Secs. II and V A. For instance, resolution improvement beyond two fold was achieved by combining structured illumination with saturation of the excited state emission, *i.e.*, increasing the excitation intensity above a threshold where fluorophores spend a longer time in excited state than the ground state [274], termed Saturated SIM (SSIM). In such regime, fluorophore responses to intensities greater than the saturation threshold remains the same and thus the effective intensity seen by fluorophores is the saturation intensity. As such, the effective intensity pattern seen by fluorophores beyond the saturation threshold start deviating from the sinusoidal pattern. Such distorted patterns contain more than three harmonics shifting more frequencies within the pass-band of the microscope by contrast to sinusoidal patterns; see Fig. 49. However, the frequency unmixing now provides more displaced SIM harmonics in Fourier space that require more images to be separated. When this process is repeated at multiple orientations, SSIM achieves isotropic lateral resolution of approximately 50 nm on fluorescent beads [274]. However, higher computational complexity in unmixing SIM harmonics and high intensities required for saturation prevent its use for biological imaging. Instead, photo-switchable fluorescent proteins (see Sec. II) can be used that cycle between dark and bright states at much lower intensities and are more live-cell compatible. By super-imposing the photo-switching properties of these dyes with structured illumination patterns, resolutions similar to SSIM can be achieved [275, 276].

### E. Light-sheet microscope

Optical sectioning has been an important motivator toward the development of 3D volumetric microscopy such as Light-Sheet Fluorescence Microscopy (LSFM) [52]. LSFM allows optical sectioning, *i.e.*, increase in the OTF’s $k_{z}$ content, by generating a thin sheet of light, *i.e.*, a thin focal volume [277, 278]. In doing so, LSFM both simultaneously minimizes light contribution from outof-focus fluorophores, otherwise present in naive widefield microscopy (see Fig. 13), and reduces sample photodamage [278, 279].

**FIG. 51:**
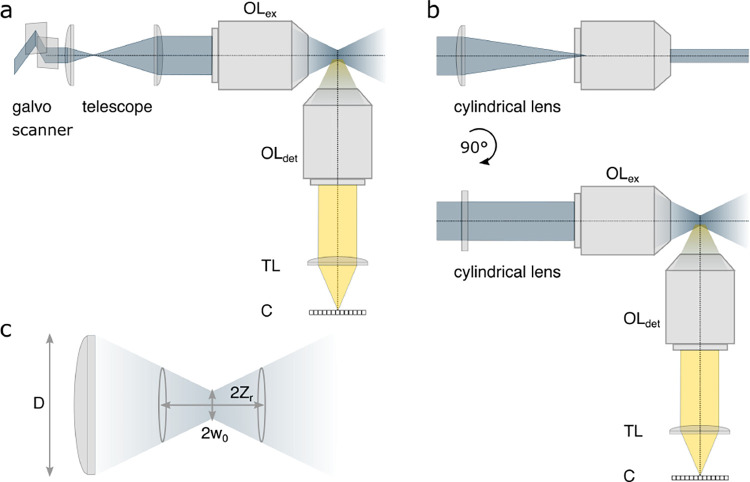
Light-sheet microscopy. (a) A Digitally scanned laser Light-Sheet Microscopy (DLSM) is created using a galvanometric (galvo) scanning unit to rapidly move a Gaussian beam perpendicular to the detection axis focused in the sample through the excitation objective lens $\left(OL_{ex}\right)$. Signal from the excited focal plane is collected through the detection objective lens $\left(OL_{det}\right)$ and tube lens (TL) onto a camera. (b) A static light-sheet is formed by a cylindrical lens in the excitation path, creating a Selective Plane Illumination Microscope (SPIM) with elongated beam in one direction (above) and the same perpendicular detection optics as in panel a. (c) A Gaussian beam is focused through a lens or objective with diameter D, beam waist $\omega_{0}$ and Raleigh length. $z_{r}$.

In LSFM, the illumination and light collection paths are orthogonal providing volumetric information on the sample when the illumination sheet is axially scanned; see Fig. 51 [280]. This facilitates faster volumetric imaging in contrast to previously discussed point scanning microscopy, like confocal (see Sec. IV B 1), scanning the sample point-by-point. Moreover, LSFM achieves optical sectioning through illumination drastically reducing photo-toxicity which, in turn, allows for longer term imaging. This is in contrast to other modalities, *e.g.*, CLSM, providing sectioning only along the detection path while illuminating large portions of the specimen along the excitation path [281]. Indeed, while TIRF (see Sec. IV A) avoids this unnecessary light dose, it is restricted to volumes neighboring the coverslide.

In modern LSFM, there are two main approaches to generate a thin light-sheet. In the first approach, a digitally scanned laser moves rapidly along a direction perpendicular to the detection axis to achieve a thin light-sheet, termed Digitally scanned laser Light-Sheet Microscopy (DLSM) [282], see Fig. 51a.

**FIG. 52:**
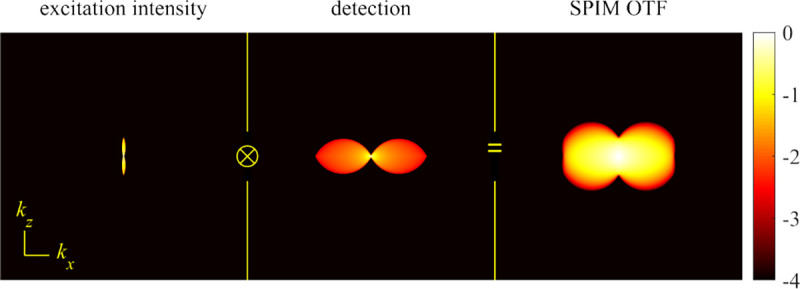
OTF of Selected Plane Imaging Microscopy (SPIM). Here, excitation is achieved by focusing a plane wave through a low-aperture lens (NA=0.4) from the left, resulting in a weakly diverging horizontally elongated excitation region. See further details in the main text.

In the second approach, termed Selected Plane Illumination Microscopy (SPIM) [53], a cylindrical lens is typically used along the excitation path to form an astigmatic Gaussian beam effectively elongating the beam in one dimension to generate a thin, static light-sheet; see Fig. 51b. The SPIM OTF is provided on the right panel of Fig. 52, and obtained by convolving the SPIM light-sheet’s Fourier representation (SPIM excitation OTF) on the left panel with the wide-field detection OTF in the middle panel. Compared to the wide-field OTF in Fig. 21, the resulting SPIM OTF has a larger bandpass along the z-axis facilitating optical sectioning. For Gaussian beams [53, 282], LSFM’s axial resolution is then, as a first approximation, related to the Gaussian beam’s thickness at twice the beam waist $z_{min}=2w_{0}$ see Fig. 51c. Similarly, the FOV is related to the extent of the elongated Gaussian beam given by twice the Raleigh length $2z_{r}$ [278]

(145) $$z_{min}\approx 2w_{0}=4\frac{\lambda f}{\pi D}=\frac{2n\lambda }{\pi NA}$$

(146) $$FOV=2z_{r}=2\frac{\pi w_{0}^{2}}{\lambda }$$

where f and D are, respectively, the focal length and lens diameter, with NA=nD/2f.

The improvement in axial resolution afforded by LSFM can be made clear when comparing LSFM and wide-field axial resolutions approximately given by Eq. 145, and Eq. 17, as well as differently derived in Eq. 85, respectively. Here, we note that for typical LSFM NA<0.8. According to Eqs. 145, and 146, while thinner light-sheets (smaller $w_{0}$) improve axial resolution, they lead to smaller FOVs because of worsening illumination uniformity across the FOV. Such non-uniform illuminations may also result in varying PSFs and OTFs across the FOV [283].

To soften the above trade-off and achieve simultaneous high axial resolutions and large FOVs, a few attempts have been made employing alternatives to Gaussian beams including: Bessel beams [246, 284]; Bessel beam lattices [285]; Airy beams [286, 287]; spherically aberrated beams [288]; and double beams [289]. While these beams achieve a Raleigh length typically larger than the Gaussian beam, it is unclear in practice whether high axial resolutions and contrasts are maintained [290–293]. This is because these alternative beams exhibit strong side-lobes leading to contribution of glare worsening axial resolution and contrast. Moreover, due to these side-lobes, the complex form of the resulting OTF does not lend itself to resolution estimates relying on Eq. 67 or Eq. 146 [290, 292].

Further efforts at rejecting the light contribution from these side-lobes combined LSFM with CLSM, SIM, and two-photon microscopy [246, 294, 295]. Moreover, the concepts of Reversible Saturable OpticaL Fluorescence Transitions (RESOLFT) (later introduced in Sec. VA), and STED have been used in conjunction with SPIM to surpass the diffraction limit in the axial direction [296, 297]. Light-sheet illumination has also been combined with non-linear fluorophore response to light (see Sec. II) for SMLM [298–300].

What is more, since the achieved resolution in the lateral and axial directions differ, to avoid anisotropic resolutions, advanced LSFM configurations use multiple objectives to generate different views of the specimen. These images are then computationally fused yielding improved isotropic resolution in all dimensions [301–304]. An alternative technique involves Axial Swept Light-sheet Microscopy (ASLM) [280, 305, 306] that generates isotropic images by scanning the sample laterally, *i.e.*, perpendicular to the detection arm, using a tightly focused light-sheet synchronized by a moving camera shutter. This only allows fluorescence originating from the well-focused parts of the light-sheet to reach the camera.

On the engineering front, orthogonal detection, and illumination through separate objectives (see Fig. 51) pose technical challenges when using two, bulky, high NA objectives. As such, multiple modification to conventional LSFM have been proposed. For instance, the iSPIM (inverted SPIM) design uses two objectives (NA 0.8–1.1) at 45 angle with respect to the coverslide [307]. More recently, different approaches have been developed achieving illumination, and fluorescent light collection using a single objective allowing use of higher NA objectives [299, 300, 308–310].

### F. Multi-plane microscope

**FIG. 53:**
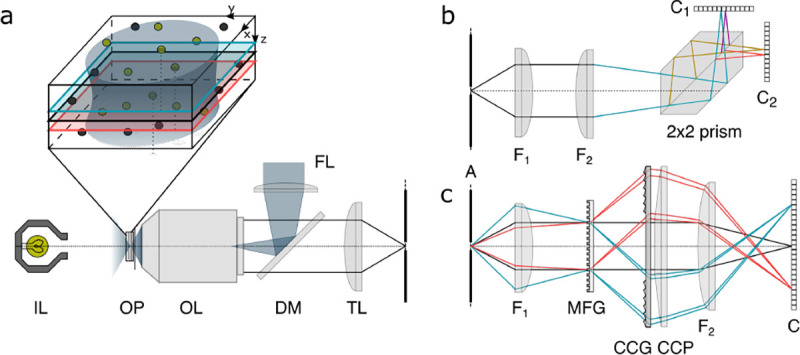
Multi-plane microscopy. (a) A conventional fluorescence microscope with epi-fluorescence (FL) and white light illumination (IL) acquire images of different focal planes across the sample by moving the objective lens (OL), and the sample with respect to each other. Here, the nominal focal plane is indicated in black while the planes shown in red and blue can be also imaged by adjusting the axial positions of, for example, the sample. Here, we, respectively, have object (OP), objective lens (OL), dichroic mirror (DM), and tube lens (YL). (b) A multi-plane microscope relays the optical path from the intermediate image formed in a) via a telescope with lenses of focal lengths F_1_, and F_2_ and uses a beam-splitting prism, *i.e.*, a refractive element, along the detection path to separate fluorescence emission into multiple channels (here four) with different focal planes projected next to each other on two cameras (C1, C2); see Ref. [312]. (c) A multi-focus microscope uses a multi-focus grating (MFG), *i.e.*, diffractive element, chromatic correction grating (CCG) and prism (CCP) to achieve multiple focal planes on one camera; see text for more details.

As discussed in Sec. III C, wide-field microscopy cannot provide axial locations with high resolution due to the missing cone of the OTF; see Fig. 13. One way to remedy this problem is acquiring images from multiple different planes across samples. The simplest approach toward achieving this is by moving the sample and focus plane with respect to each other; see Fig. 53a. However, this involves moving a large inertial object (sample, objective, camera) introducing time lags between planes and mechanical perturbation. Fast, adaptive elements or small moving components in a more complex detection path can speed this up, but do not alleviate the need for axial scanning. Therefore, simultaneous acquisition of multiple focal planes in the sample without moving the sample, or optical components, is desired. This has been achieved by introducing either refractive or diffractive optical elements into the detection arm to split the fluorescent emission into multiple paths leading to simultaneous acquisitions from different focal planes within the sample [54, 55, 311–313]. For a more in-depth review on “snapshot” volumetric microscopy see *e.g.*, Ref. [314].

Multi-plane, also termed multi-focus microscopy imaging, is versatile and can be combined with wide-field fluorescence, or light-sheet excitation [315] for a number of applications. These include: SPT [316, 317], super-resolution microscopy [318, 319] (for statistical modeling of multi-plane super-resolution SMLM data see Sec. VC), Super-resolution Optical Fluctuation Imaging (SOFI) [312, 320], structured illumination [321, 322], as well as single cell and whole organism imaging [311, 323, 324]. Furthermore, phase imaging [323, 325], polarization [326] and dark-field microscopy [323, 324] may also use a multi-plane setup.

In its simplest form, multi-plane microscopes use beam-splitters, *i.e.*, refractive elements, in combination with optical detection paths of different lengths, or tube lenses with different foci [55, 319, 320, 324, 327]. In such setups, the inter-plane distance, and thus axial resolution, can be independently adjusted from the pixel size (related to lateral resolution; see Sec. I C).

However, these versatile implementations are susceptible to misalignment of the detection channels due to opto-mechanical component drift especially relevant in super-resolution microscopy; see Sec. V. A more elegant solution involves a cascade of beam-splitters fused into a single piece, *i.e.*, prism [312, 323], dividing the fluorescent light into multiple beams traveling optical paths with different lengths; see Fig. 53b. Here, increased mechanical stability is afforded by virtue of having all beam-splitting as one optical element, *i.e.*, prism, with minimal chromatic aberration. This setup can also be extended to simultaneously image several colors across planes by the addition of a spectral beam-splitter [328].

An alternative approach uses a Multi-Focus Grating (MFG), *i.e.*, a diffractive element, to split the fluorescent emission into multiple paths corresponding to different diffraction orders. The grating pattern is designed to introduce diffraction order dependent de-focus phase shifts (see Sec. III F) leading to different focal planes for each path [54]; see Fig. 53c. However, the grating introduces chromatic dispersion, improved by introducing a Chromatic Correction Grating (CCG), and a Prism (CCP) to reverse the dispersion due to MFG [311] and seperate the images laterally on the camera chip; see Fig. 53c. While aberration-corrected multi-focus microscopy grating design can further improve imaging of thicker samples [311, 329, 330], gratings have lower transmission, and new gratings are required to alter inter-plane distances.

## V. SUPER-RESOLUTION MICROSCOPY

Resolution across fluorescence microscopy, as described in Sec. IV, is fundamentally limited by the frequency band-pass given by the corresponding OTFs. This restricts the maximum achievable resolution to approximately 100 nm under optimal conditions. This limit can be surpassed by exploiting the non-linearity in fluorophore response to excitation light; see Sec. II. This, in turn, has lead to the development of two main categories of super-resolution, or nanoscopy, methods to which we now turn: 1) targeted switching; and 2) stochastic switching techniques.

### A. Targeted switching super-resolution microscopy

#### 1. Stimulated emission depletion microscopy

Previously introduced fluorescent imaging techniques such as confocal, light-sheet, and multi-plane microscopy improve axial resolution using different optical sectioning strategies. Optical sectioning limits the collected fluorescence to an axial subset of fluorescent molecules preventing interference from fluorophores outside this axial subset. Although these techniques can significantly increase contrast, and improve axial resolution, their resolution remains limited by the diffraction of light. On the other hand, super-resolution methods such as STED microscopy [56, 331], and its generalization, RESOLFT [332, 333], are based upon a traditional point scanning microscope with confocal pinhole in the detection arm allowing higher resolution imaging while retaining the axial sectioning of confocal microscopy.

STED imaging was first achieved in the mid-nineties by Hell and Wichmann [56] and its popularity grew thanks to the high spatial resolution, relatively high imaging speed, and considerable imaging depth. These made possible, for instance, the visualization of biomolecular assemblies and live-cell naoscopy [333, 334].

In terms of temporal resolution, as fast as milli-second imaging times for rapid dynamics in small fields of view was demonstrated by ultrafast STED nanoscopy [335], while spatially, the highest reported 3D isotropic resolution (< 30 nm in x,y,z simultaneously) was validated with the ultra-stable design of 4pi-based isoSTED [336].

In STED, spatial resolution improvement is achieved by adding a second de-excitation (depletion) laser quenching fluorescence around the excitation point confining fluorescence emission to a sub-diffrcation limited spot. Stimulated emission is one means by which to depopulate excited states. In this process, theoretically discovered by Albert Einstein [337], the incoming photon triggers the excited system to decay to its ground state, emitting a photon, with a phase, frequency, polarization, and momentum identical to the incident photon; see Sec. II.

**FIG. 54:**
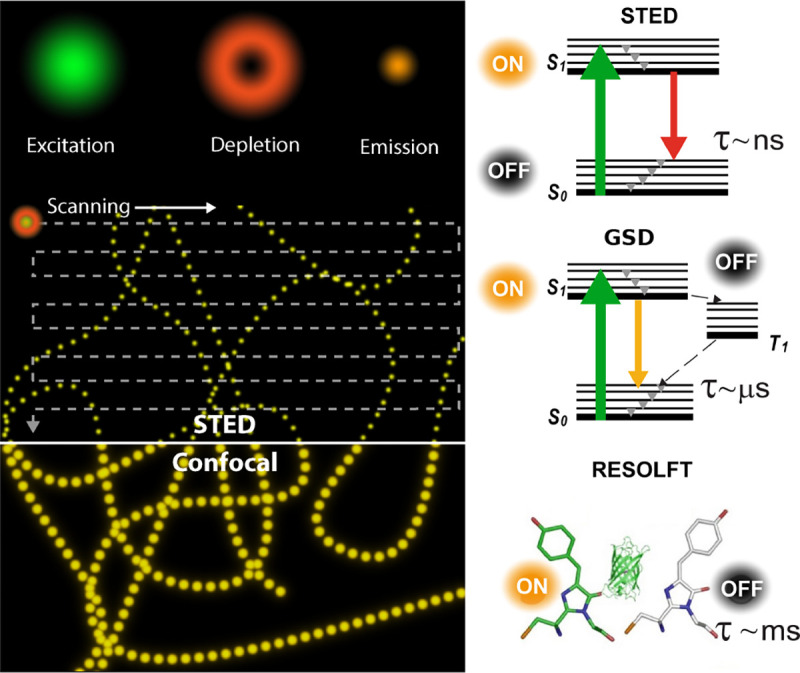
Schematics for STED imaging. a) Excitation and depletion beams are used to acquire a subdiffraction-limited image, formed after raster scanning the full sample. The image can be understood as a convolution between the effective PSF combined from the excitation, and depletion laser beams, and the fluorescent molecule distribution in the sample. Image adapted from Refs. [332, 333]. Schematics on the left hand side compare diffraction-limited confocal images of microtubules with the coinciding STED image. On the right panel we show the electronic transitions of excitation, and stimulated emission in STED (top), ground-state depletion GSD (middle), and RESOLFT (bottom). Figure is adapted from Ref. [339].

In STED imaging, stimulated emission must precede spontaneous emission, requiring the excitation light to excite the sample (≈ 200 ps) prior to laser quenching. The whole imaging protocol can be devised in two steps; see Fig. 54. First, the fluorophores are excited by a diffraction-limited laser beam with a Gaussian waist designated by green in Fig. 54. If we wait until molecules spontaneously decay, and count all photons emitted, the signal will be approximately proportional to the number of molecules present in the laser spot, and no gain in resolution will be achieved. Therefore, it is necessary to introduce the second step where a fraction of the fluorophores are depleted using a torus, or donut-shaped diffraction-limited beam shown in red in Fig. 54, whose central minimum coincides with the Gaussian excitation maximum. As such, the recorded signal only originates from the “donut hole” far narrower than the original Gaussian waist shown in orange in Fig. 54. To understand how STED beams are generated, see Sec. IV B 1 and Fig. 30.

The resolution gain in STED, $y_{STED}$, given below is set by the inner donut radius and may become theoretically infinite

(147) $$y_{STED}=\frac{\lambda }{2NA\sqrt{1+\frac{I}{I_{sat}}}}=\frac{y_{min}}{\sqrt{1+\frac{I}{I_{sat}}}}.$$

Here, $y_{min}$ is the wide-field resolution (see Eq. 16), and I is the depletion laser intensity. Moreover, $I_{\text{sat}\;}$ is the depletion intensity required to outperform the competing transition, *i.e.*, fluorescence emission.

Although the resolution achieved by STED is, in principle, arbitrarily small provided high enough depletion intensity (I→∞) [338], in practice, the achievable resolution is limited by a number of factors. These factors include the nature of the fluorophores used (and their absorption cross-section of the depletion beam), any uncorrected aberration (residual aberration) of the STED pattern, SNR, as well as the STED beam’s relatively high power resulting in potential sample photo-damage.

Photo-damage can be mitigated by working with solid state fluorescent nanodiamonds hosting negatively charged nitrogen-vacancy (NV) point defects. Using such photo-stable labels, resolutions of ≈ 10 nm was demonstrated [340, 341]. However, the complex functionalization of relatively large size 10–15 nm solid-state probes, including issues related to specificity and cell permeability, limit their applications especially for live-cell STED imaging.

While we have focused on 2D thus far, by using interference of two depletion beams (see implementation of 4pi microscopy introduced in Sec. IV B 3), STED super-resolution imaging has been extended to 3D [342, 343] though, in practice, axial resolution gain (≈ 100 nm) comes at the cost of lower lateral resolution.

#### 2. Reversible saturable optically linear fluorescence transition microscopy

Numerous efforts in the last two decades have been undertaken to improve upon the strongest limitation of STED, namely high power depletion beams [334]. Along these lines, a more general method, RESOLFT, (encompassing STED as a special case) was proposed in the early 2000’s [333], leveraging fluorophore photo-physics. This, in turn, renders RESOLFT more appropriate for live-cell, and long-term experiments [332]. Moreover, RESOLFT can also be employed for 3D live-cell imaging using a recent implementation of highly parallelized image acquisition with an interference pattern [344].

In contrast to STED, whose high laser power is required to deplete the excited state back to the ground state, RESOLFT uses donut-shape beams to transition fluorophores into any dark state, not just the ground state; Fig. 54. Thus RESOLFT requires fluorophores controllably switchable between dark (OFF), and bright (ON) states; see Fig. 54. For instance, such fluorophores include reversibly switchable fluorescent proteins, and dyes [345, 346].

One such dark state is the triplet state; see Sec. II. Leveraging transitions to this state forms the basis of ground state depletion (GSD) [347], a special case of RESOLFT requiring less depletion laser powers; see Fig. 54.

#### 3. Minimal photon fluxes

Due to the limited photo-stability of fluorophores, *e.g.*, photo-bleaching, first generation nanoscopy methods such as STED, and RESOLFT reached their practical resolution limit of 20–40 nm. The latter motivated the development of a second generation of fluorescence nanoscopies reaching resolutions ranging from 1–10 nm.

**FIG. 55:**
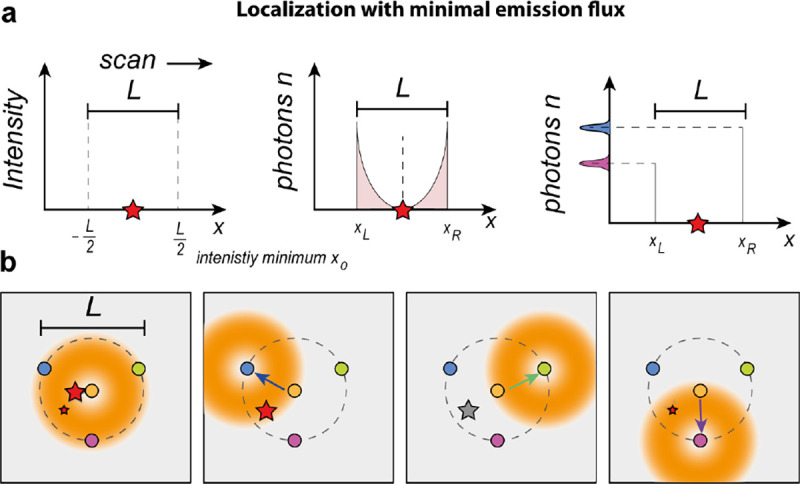
Working principle MINFLUX. (a) 1D MINFLUX employs a well-defined excitation pattern to extract the emitter’s position using only two single measurement points at known separations, L. (b) 2D MINFLUX employs a donut-shape excitation beam, and here uses four measurements with the central minimum of the donut of the excitation beam when displaced at all four locations shown by circles. The red, green, and pink circles are arranged to form an equilateral triangle. Figure is adapted from Ref [348].

An example of such a nanoscopy technique includes MINimal photon FLUXes (MINFLUX) introduced in 2017 [348] which extracts information from a limited photon budget and using minimal laser intensities [348–350]. In this imaging modality, in contrast to STED, it is the excitation beam that has a donut-shape with the intensity minimum at its center. The excitation beam is scanned across the sample and the fluorescence signal is then collected by a confocal microscope. In MINFLUX, the intensity minimum at the excitation beam center is used as the reference coordinate in determining the position of the molecules. In the first MINFLUX implementation, the molecule’s position is triangulated using photons collected from each position of the donut-shaped beam rapidly displaced by a small distance to assume the location of the vertices of an equilateral triangle [348]. The location, $r^{⋆}$, then determined using MLE with data drawn from those displacements of the excitation beam yielding fewer photons ultimately contributing more heavily to the determination of $r^{⋆}$. It then follows that data drawn from more beam displacements, such as four in Fig. 55, provides improved spatial resolution albeit for static structures.

Further implementations of MINFLUX accomplish simultaneous 3D and multi-color imaging [350]. This achieves very high isotropic localization precisions on the order of 1–3 nm resolving nanoscale structures.

MINFLUX concept has also been adapted to wide-field microscopy for faster imaging substituting donut-shaped illumination with structured illumination [351–354]. In these methods, fluorophore locations are realized with respect to the sinusoidal patterns.

While we have focused on spatial resolution here, MINFLUX can easily be translated to SPT experiments [349, 355] localizing with a precision below 20 nm within ≈100 *μ*s [356].

Finally, MINFLUX is fundamentally limited to dilute samples motivating development of the method illustrated in Fig. 46. In this method, BNPs were used to track multiple molecules at once.

### B. Stochastic switching super-resolution microscopy

Previously we described super-resolution methods based on targeted switching of fluorophores. Here, we discuss single molecule based super-resolution methods, a family of super-resolution techniques, achieving sub-diffraction resolution by imaging independent, and stochastically blinking fluorophores over time [357–359]. In these methods, the gain in spatial resolution is traded for temporal resolution as the acquisition of many camera frames is required to computationally reconstruct a single super-resolved image. In such experiments, a conventional wide-field microscope is typically used to collect fluorescent light from (photo)activatable, or switchable probes (see Sec. II A). Moreover, scanning image acquisition have also been successfully used to implement super-resolution microscopy [360].

**FIG. 56:**
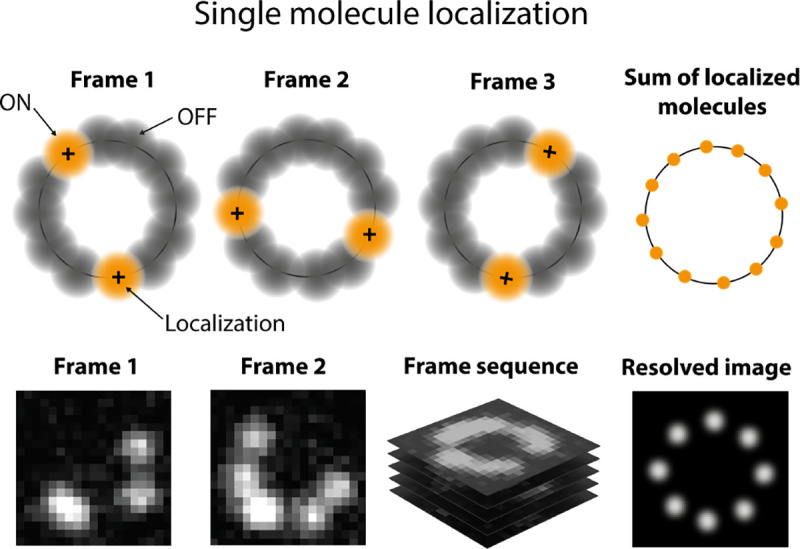
Single emitters are stochastically activated to become fluorescent. The activated emitters can be precisely localized provided they are spaced further apart than the Nyquist limit; see Sec. I C. The process is repeated for tens of thousands of frames. In each frame, single-emitters are identified and fitted to obtain their center of mass, allowing super-resolved pointillistic image reconstruction (see bottom panel right). Repetitive activation, localization, and deactivation temporally separate spatially unresolved structures in a reconstructed image with apparent resolution gain compared to the standard diffraction-limited image of fluorescently labeled microtubules; see bottom left.

The most common use of stochastic switching is applied to techniques summarized as SMLM [359]. In SMLM, spatially overlapping fluorophores are temporally separated by acquiring a sequence of image frames. In each frame, only a few fluorophores emit. Thus localization is achieved by switching on only a small fraction (<1%) of the molecules on at each frame, and localizing only those molecules independently of their immediate neighbors with which their PSF would otherwise overlap; see Fig. 2. The set of nanometer-resolved localizations are then used to reconstruct super-resolved structures; see Fig. 56.

The latter methods however require isolating molecules in determining their positions thereby imposing long data acquisition times. Therefore, more recently, a range of alternative techniques were developed to improve image resolution while avoiding identifying and localizing single molecules [361, 362]. Rather, such methods can analyze fluctuations in fluorescence emission over time, and tolerate a wider range of switching behavior, and imaging conditions including SOFI [363, 364] (see Sec. VB1), and others *e.g.*, SRRF [365], SPARCOM [366], MSSR [367], and 3B [368]. A common feature of fluctuation-based techniques is that they provide lower resolutions compared to SMLM methods but require fewer input frames, and lower laser powers as compared with SMLM, making them more live-cell compatible at the cost of a reduced gain in resolution.

#### 1. Super-resolution optical fluctuation imaging

Super-resolution Optical Fluctuation Imaging (SOFI) [369, 370] is a computational post-processing tool for super-resolution single molecule data akin to FCS. In contrast to localization microscopy (see Sec. VB2), SOFI is not aimed at resolving isolated molecules. Instead, the fluctuations caused by stochastic switching of the underlying molecules only need to be visible and the method thus is robust to the presence of overlapping PSFs in dense regions. The fundamental principle behind SOFI’s resolution improvement is to exploit correlations in the light emitted by single fluorophores. Fluctuations of a molecule only correlate with itself in space and time and not with neighboring molecules.

The data processed in SOFI consists of photon counts (intensity) $w_{n}^{k}$ at pixel n in frame k detected on a wide-field camera, and is described as follows

(148) $$w_{n}^{k}=ℬ+\sum_{m=1}^{M}I_{m}U\left(r_{n}-r_{m}\right)s_{m}^{k}+\varepsilon_{n}^{k}$$

with M denoting the number of fluorophores, $I_{m}$ the molecular brightness per frame, $U\left(r-r_{m}\right)$ the optical system’s PSF, $s_{m}^{k}$ describing the switching of the fluorophore between the on-state and the off-state, ℬ an average background, and $\varepsilon_{n}^{k}$ representing additive noise. Moreover, the sample is assumed to be stationary during image acquisition. Here, we have approximated the integral of the PSF over a pixel by the pixel value at the pixel center.

In its simplest implementation, SOFI computes cumulants, $\kappa \left(w_{1:N}^{1:K}\right)$, of the pixel intensities across frames. For instance, the second order cross-cumulant coincides with the co-variance in signal intensity across frames in one pixel for different time lags. The *l*th order cumulant takes the form

(149) $$\kappa_{l}\left(w_{1:N}^{1:K}\right)\approx I^{l}f_{l}\left(\rho_{\text{on}}\right)\sum_{k=1}^{K}U^{l}\left(r-r_{k}\right),$$

where $f_{l}\left(\rho_{\text{on}\;}\right)$ denotes the *l*th order cumulant of $s_{m}^{k}$ given as an *l*th order polynomial with respect to the probability of the molecule (ratio of molecules) to be on $\rho_{on}$. Moreover, the cumulant of the noise and background are zero. In Eq. 149, critical to SOFI analysis, appears the PSF raised to the *l*th power. This means that the *l*th order cumulant, if plotted instead of the original image, yields a PSF $\sqrt{l}$ narrower than the original PSF and offers an up to l-fold enlarged frequency support in Fourier space. As such, the resolution can be increased up to l-fold with post-processing either by Fourier reweighing [368] or deconvolution [366, 369] as discussed earlier, *e.g.*, see confocal (Sec. IV B 1) and ISM (Sec. IV B 2) microscopy.

This can be further generalized to spatio-temporal cross-cumulants with various time-lags across different pixel combinations to leverage spatial information albeit at higher computational cost [371–373]. A time lag of zero reduces the computational complexity and ensures the maximum of the signal. Using cross-cumulants, shot-noise contributions are eliminated and virtual pixels in between the physical pixels acquired by the camera are generated, which leads to a finer sampling.

One challenge in SOFI is the non-linearity of the post-processing to molecular brightness. For instance, post-processing may amplify signal heterogeneities and mask dimmer structures [372]. Different linearization procedures have been implemented to mitigate this problem [372, 374]. Furthermore, compared to SMLM, SOFI is relatively insensitive to background signal, and only a few hundred to a thousand frames are needed for cumulant calculation. SOFI also tolerates higher labeling densities, higher on-time ratios, and lower SNR [374–376]. This means it can allow more gentle, *i.e.*, less photo-damaging, and faster live-cell imaging. Moreover, SOFI achieves optical sectioning and resolution improvement in the z-direction using simultaneously acquired multi-plane data [312, 377].

#### 2. Single molecule localization microscopy

Almost a decade before its experimental realization [92, 378], the idea underlying SMLM was theoretically proposed by Eric Betzig [379]. The experimental implementations was done employing photo-activatable genetically encoded proteins [380] and quantum dots [378].

An initial iteration was termed (f)PALM [92, 93]. Shortly thereafter, Rust *et al.* developed Stochastic Optical Reconstruction Microscopy (STORM) [381], exploiting photo-switching in organic dyes. In principle, these first generation SMLM methods, PALM and STORM, differ only in their means of achieving temporal separation of spatially overlapping fluorophores. However, as PALM uses photo-activatable or photo-convertible fluorescent proteins [382], it allows for genetic expression and is compatible with live-cell imaging [382], and thus stoichiometric labeling of target proteins used in counting [62, 132].

On the other hand, organic fluorophore photon emission rates are typically higher compared to photo-activatable or photo-convertible fluorescent proteins, resulting in slightly better resolution in STORM. The latter motivated the development of a more general dSTORM modality introducing a pallet of synthetic organic fluorophores as photo-switchable probes [383] later allowing imaging inside living cells when combined with site-specific tagging methods [384].

**FIG. 57:**
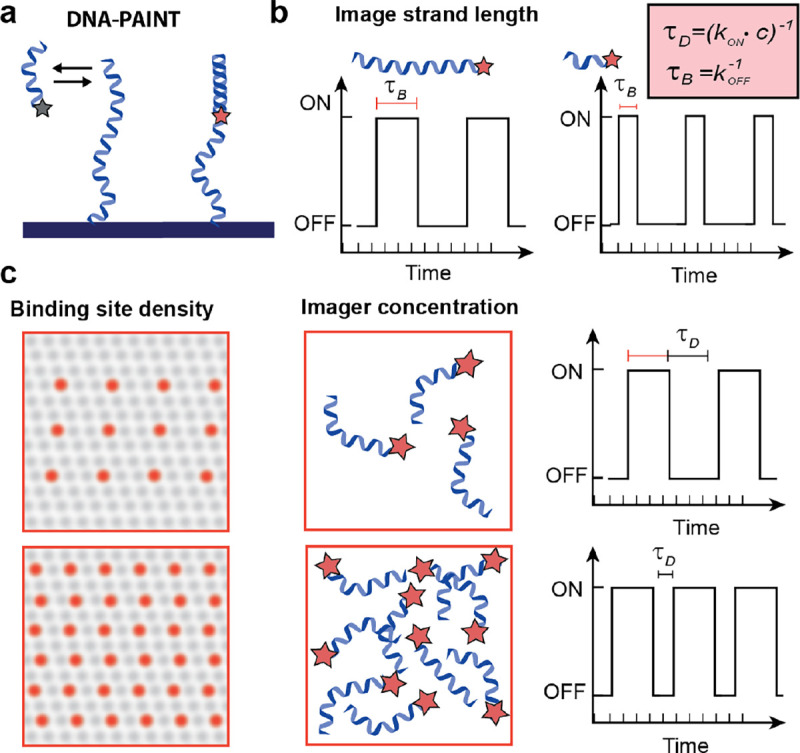
The general principle of DNA-PAINT. (a) Schematics illustrate DNA-PAINT where dye-conjugated oligo (imager oligo) transiently hybridizes with a complementary (docking) oligo. In DNA-PAINT imaging, the complementary/docking oligos are chemically modified to bind to the secondary antibody. (b) The binding time $\tau_{B}$ (or the dissociation rate $1/\tau_{B}$) depends on the length of the imager strand. (c) This panel illustrates how the increase in imager strand concentration and density of docking sites both lead to decrease in dark times, $\tau_{D}$ (inter-event lifetime). Figure is adapted from Ref. [385].

An alternative SMLM method is DNA Point Accumulation for Imaging in Nanoscale Topography (DNA-PAINT) employing stochastic transient binding of diffusing dyes in solution with a complementary molecule bound to the target structure [385]; see Fig. 57. Upon binding, the dye molecule is temporally immobilized, and detected by the camera while the freely diffusing dyes, strongly aliased, are difficult to track, and thus treated as effectively indistinguishable from background.

DNA-PAINT is a photo-bleaching free method as imaging can be continued as long as diffusing dyes are present in solution. The achievable spatiotemporal resolution in DNA-PAINT is set by experimental design; see Fig. 57. For instance, the length of the imager strand increases the binding time leading to a higher number of photons generated during one single binding event, and thus improved SNR resulting in higher spatil resolutions, driven by longer exposures; see Fig. 57b. Furthermore, DNA-PAINT is compatible with multiplexing using color and the binding kinetics [386–388].

#### 3. SMLM data analysis

In SMLM, the acquired data, $w_{1:N}$, typically consists of a set of pixel values (observation) organized as 2D arrays, called image frames. Here, molecular localization is probabilistically related to the pixel values, $w_{n}$, through the likelihood.

To build the likelihood, we begin with the expected photon counts for the *n*th pixel given as

(150) $${\text{Λ}}_{n}=ℬ+\sum_{m=1}^{\infty }b_{m}I_{m}{𝒫}_{m}^{n}.$$

where we have immediately generalized our model to the most practical case with an unknown number of emitters. That is, in this model, we adopt a non-parametric framework with an infinite number of emitters (m=1:∞) with associated load $b_{m}$ for each emitter (see Sec. I B). The loads associated to the emitters not contributing any photons are, as usual, found to be realized to zero. Moreover, $I_{m}$ and ℬ, respectively, represent the intensity of the *m*th emitter and a uniform background. Here, ${𝒫}_{m}^{n}$ is the probability of a photon from the *m*th emitter reaching the *n*th pixel given by (see Eqs. 4–5)

(151) $${𝒫}_{m}^{n}=\iint_{{𝒜}_{n}}\;dxdyU\left(x,y;r_{m}\right),$$

where ${𝒜}_{n}$ is the pixel area and $r_{m}=\left(x_{m},y_{m},z_{m}\right)$ is the emitter position. As a simplification, sometimes this integral is approximated by the PSF value at the middle of the pixel [386].

The integral above can be readily calculated, say, as error functions, for Gaussian PSFs. However, the integral of Eq. 151, for any given axial location, can also be substituted for a sum when the PSF does not assume a simple analytical form as is often the case when dealing with engineered PSFs; see Sec. V C. Moreover, since numerical PSF values typically provided for select axial positions (often uniformly spaced), linear or spline interpolations [390, 391] are used to estimate the PSF at intermediate values.

For concreteness here, we use the CCD detector noise model and arrive at the following likelihood for the *n*th pixel

(152) $$P\left(w_{n}|\vartheta \right)=Gaussian\left(w_{n};g\Lambda_{n}(\vartheta )+o,\sigma_{w}^{2}\right),$$

where g,o and $\sigma_{w}^{2}$ are, respectively, the detector gain, offset, and variance. As before, we collect all unknown parameters in $\vartheta =\{\overset{‾}{b},\overset{-}{r},\overset{‾}{I},ℬ\}$ where the overbar denotes quantities over all emitters. Finally, since pixel values are *iid* (see Sec. I B]), the likelihood of a ROI containing N pixels assumes a product form

153) $$P\left(w_{1:N}|\vartheta \right)=\prod_{n=1}^{N}P\left(w_{n}|\vartheta \right).$$

In parametric frameworks, the likelihood obtained in Eq. 153 can be simplified assuming a known number of emitters M

(154) $${\text{Λ}}_{n}=ℬ+\sum_{m=1}^{M}I_{m}{𝒫}_{m}^{n}.$$

In such parametric frameworks, the number of emitters are typically determined separately using alternate criteria, *e.g.*, Bayesian Information Criteria (BIC) [28], thresholding [392], p-values [393], or other methods [152]. In contrast, non-parametrically, just as before, the number of active emitters are treated as random variables [208, 223].

In a BNP framework, we construct the posterior from the product of the likelihood Eq. 153, and the priors over the unknown parameters; see Sec. I B. We may adopt an empirical prior for the intensity of fluorophores obtained by fitting isolated emitters from sparse regions of the data [394], and assume a beta-Bernoulli process prior for the loads; see Sec. I B.

In SMLM, the localization stage as described above typically generate multiple locations per emitter often with low precision [395–397] hindering the quantification of biological structures. To address this issue, a few methods were put forward [396, 397] relying on photobleaching to enumerate the underlying emitters. To remove the photo-bleaching requirement, a method termed Bayesian Grouping of Localization (BaGoL) was developed to deal with localizations obtained in both presence or absence of photo-bleaching, *e.g.*, dSTORM or DNA-PAINT. Moreover, BaGoL accomplishes sub-nanometer precision under dense labeling conditions by removing nanometer residual drift within the input data and combining multiple identified localizations from each emitter [395].

After focused on static emitters, we now broaden our discussion to mobile emitters, namely tracking emitters across frames. In SPT, data consists of N pixel values for each frame k=1:K denoted by

(155) $$w_{1:N}^{1:K}=\left\{w_{1}^{1},w_{2}^{1},\ldots ,w_{N}^{1},w_{1}^{2},\ldots ,w_{N}^{K}\right\}\text{.}\;$$

The parameter set ϑ is now expanded to include trajectories of particles across time, $r_{m}(t)$ for the *m*th particle. By approximation, these may be reduced to locations across frames, $r_{m}^{1:K}$. However, in full generality positions can be theoretically interpolated at all inter-frame temporal values [122, 398, 399].

To obtain the SPT likelihood, similar to SMLM, we start from the expected photon count per pixel. In SPT, as particles move over each exposure, the expected photon count for the *n*th pixel in the *k*th frame, $\Lambda_{n}^{k}(\vartheta )$, follows from Eq. 150 [398, 399]

(156) $$\Lambda_{n}^{k}(\vartheta )=ℬ+\sum_{m=1}^{\infty }b_{m}\int_{{\text{exposure}}_{k}}dt\mu (t){𝒫}_{m}^{n}(t).$$

Here, ${𝒫}_{m}^{n}(t)$ is adapted from Eq. 151 with time dependent location, and μ(t) is the time dependent fluorescence emission rate, *e.g.*, due to blinking. The time integral of Eq. 156 is stochastic and numerical integration is often used in its evaluation. We may approximate the integrand as a constant and pull it from the integral resulting in Eq. 151 [400]. However, this approximation may fail due to motion blurring artifacts, *i.e.*, aliasing, when particles diffuse rapidly compared to the camera frame rate [401, 402].

As an alternative, an improved approximation is afforded by the trapezoidal rule

(157) $${\text{Λ}}_{n}^{k}\left(\vartheta \right)=ℬ+\sum_{m=1}^{\infty }b_{m}\sum_{l=1}^{L-1}\frac{\delta t}{2}[\mu_{m}\left(t_{l}^{k}\right){𝒫}_{m}^{n}\left(t_{l}^{k}\right)\;\;+\mu_{m}\left(t_{l+1}^{k}\right){𝒫}_{m}^{n}\left(t_{l+1}^{k}\right)]$$

with

(158) $${𝒫}_{m}^{n}\left(t_{l}^{k}\right)=\iint_{{𝒜}_{n}}\;dxdyU\left(x,y;r_{m}\left(t_{l}^{k}\right)\right).$$

In this equation, $t_{1}^{k}$ represents the beginning of the *k*th frame’s exposure while $t_{L}^{k}$ represents the end. The entire exposure period, δT, is divided into L-1 equal panels of length $\delta t=\frac{\delta T}{L-1}$.

A motion model, such as free diffusion, may now be introduced to connect positions

(159) $$r_{m}\left(t_{l+1}^{k}\right)|r_{m}\left(t_{l}^{k}\right)∼\mathrm{Normal}\left(r_{m}\left(t_{l}^{k}\right),2D\delta t\right),$$

where D is the diffusion coefficient of the emitters, assuming they all have the same diffusion dynamics.

The diffusive model is most common though alternative models, such as anomalous diffusion, are also invoked [403]. It remains to be seen however whether alternative models can be useful in light of dramatic approximations often already made in the analysis including, but not limited to, assuming: a number of emitters by hand [404]; a time independent integral of Eq. 156; general corrupting noise from photon count and detectors [404], and many other sources of error.

The emission rates $\mu_{m}$ of the emitters can also be described using Markovian models [62, 132]; see Sec. II. However, for the sake of simplicity, we will assume that all emitters maintain the same brightness throughout the entire frame sequence, resulting in the simplification of Eq. 157 to

(160) $${\text{Λ}}_{n}^{k}\left(\vartheta \right)=ℬ+\mu \sum_{m=1}^{\infty }b_{m}\sum_{l=1}^{L-1}\frac{\delta t}{2}\left[{𝒫}_{m}^{n}\left(t_{l}^{k}\right)+{𝒫}_{m}^{n}\left(t_{l+1}^{k}\right)\right].$$

Again assuming, for simplicity alone, a CCD camera noise model (see Appendix A), the likelihood for the *n*th pixel in the *k*th frame reads

(161) $$P\left(w_{n}|\vartheta \right)=Gaussian(w_{n}^{k};g\Lambda_{n}^{k}(\vartheta )+o,\sigma_{w}^{2}).$$

Now, similar to the SMLM likelihood in Eq. 153, the likelihood of the frame sequence is

(162) $$P\left(\vartheta |w_{1:N}^{1:K}\right)=\prod_{n}\prod_{k}P\left(\vartheta |w_{1:N}^{1:K}\right).$$

By specifying all terms in Eq. 162, we see that the definition of ϑ must be expanded to include $\vartheta =\{\overset{‾}{b},\overset{-}{r}\left(t_{1:L}^{1:K}\right),\mu ,ℬ,D\}$.

To be precise, parametrically, the unknowns would include $\vartheta =\left\{r\left(t_{1:L}^{1:K}\right),\mu ,ℬ,D\right\}$, and the number of trajectories (emitters) might be individually estimated using *ad hoc* methods [404]. In contrast, in the non-parametric paradigm the trajectories and number of emitters would be treated like the other parameters. In this paradigm, the unknowns would include the loads, $\overset{‾}{b}$, as described earlier [208, 223].

Within the non-parametric paradigm, we use the likelihood derived in Eq. 161 alongside appropriate conjugate priors (see Sec. I B), when these can be identified, to construct and sample from the posterior probability distribution. This is outlined in the box below [398, 399].

Now with the posterior at hand, we use Monte Carlo methods to draw samples and infer the set of unknowns ϑ. The posterior for the loads, $\overset{‾}{b}$, and the diffusion coefficient, D, assumes a closed form and can be directly sampled. For the remaining parameters, we use the Metropolis-Hasting sampling procedure; see Sec. I B.

We note that the above tracking reveals the z-position only up to a mirror symmetry above or below the focal plane when using a single plane of illumination. Thus, here, a note is warranted regarding 3D SMLM. In standard SMLM, localizing the molecule’s position along the axial direction is challenging due to the limited depthof-field and symmetry of the wide-field PSF with respect to the focal plane, *i.e.*, lack of optical sectioning; see Sec. III C. To remedy these issues, multiple approaches have been employed including multi-plane microscopy (see Sec. IV F) and PSF engineering [405–408]. Due to its simplicity, the approaches based on PSF engineering, later detailed in Sec. V C, are most commonly implemented for 3D SMLM. Engineered PSFs can be realized by intentionally introducing aberrations in a system by inserting extra optical components to the setup [405] or adaptive optical element, such as a deformable mirrors [409]. This type of aberration breaks the symmetry of the PSF and encodes the axial position in its shape. The shape of engineered PSFs rapidly vary based on its axial position, allowing precise localization of the single molecules in the axial direction over a wide range [410].

### C. PSF engineering

To overcome limited optical sectioning in SMLM imposed by the wide-field microscope PSF, described in Sec. III C, PSF’s have been engineered by adding optical elements, typically at the Fourier plane, also sometimes termed the back focal plane, pupil plane or the aperture plane [161, 408, 411].

One of the earliest PSF engineering applications allowed for the reduction of the in-focus spot size, at the cost of increased side-lobes. This was achieved by implementing a series of amplitude and phase rings in the Fourier plane [412]. As another example, toward achieving Extended Depth Of Field (EDOF), a cubic phase mask was used leading to a PSF minimally changing over a desired axial range [413]. While maintaining EDOF, other improvements were aimed at reducing the required computation and raising the SNR, *e.g.*, the log-asphere lens [414], Bessel Beams [415], and others [416].

The prior examples all coincide with PSFs maintaining their shape throughout de-focus. However, PSFs have been engineered, either heuristically, or algorithmically (more details later), to provide improved axial resolutions across different experimental conditions [411] such as emitter density and wavelength. That is, at the other extreme end of design space where PSFs remain similar throughout de-focus, reside PSFs intentionally sensitive to de-focus. The purpose of such z-encoding PSFs is to encode axial information (depth) in their shape enabling 3D tracking, or imaging [411].

An early instance of z-encoding PSF engineering is induced astigmatism, implemented typically by a cylindrical lens, for the purpose of evaluating de-focus in compact disc players [417]; an idea that adapted for SMLM [405]. The astigmatic PSF provides high axial resolution over approximately 1 *μm*.

**FIG. 58:**
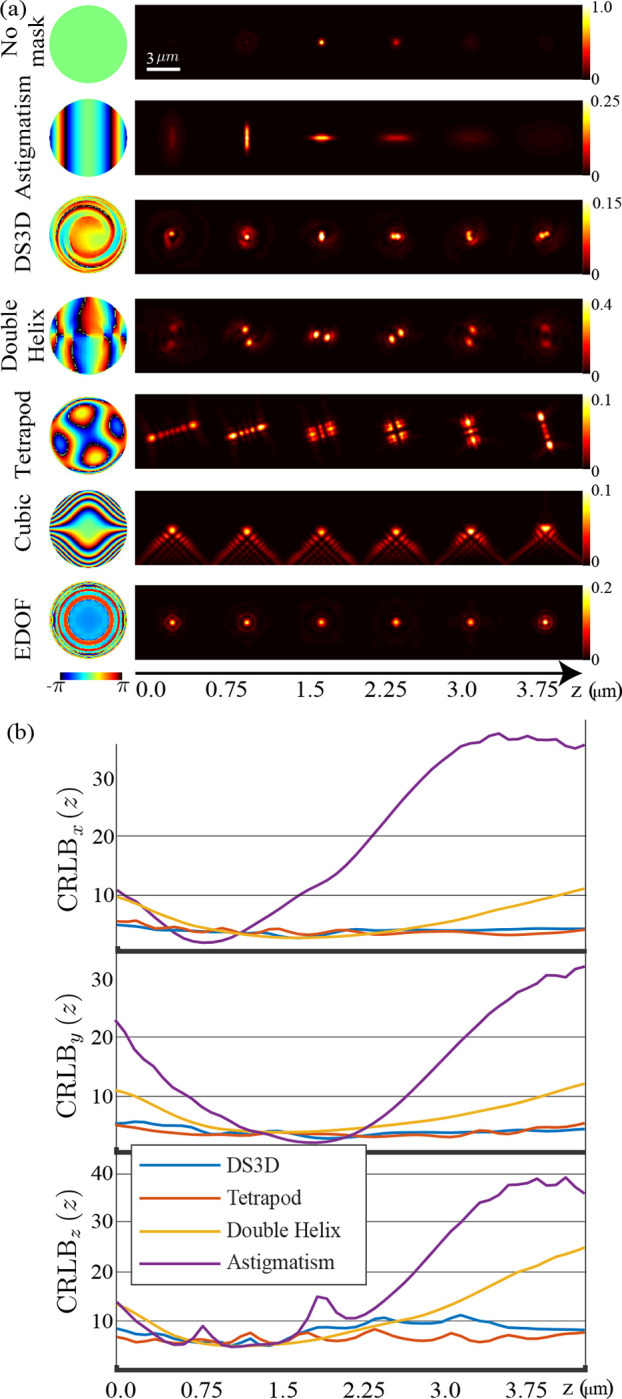
(a) Frequently used engineered PSFs, simulated for objective lens NA 1.49 with pixel size of 110 nm. The top row is the wide-field PSF. Below the wide-field PSF we present commonly used phase masks and their corresponding PSFs over a range of axial positions. (b) CRLB (detailed in Sec. I B) of the 3D position (each axis individually) plotted as a function of the axial position for some of the engineered PSFs, assuming the system is laterally shift-invariant. Here, the subscripts in the axes labels indicate the coordinate for which CRLB was calculated.

Following similar ideas, larger axial ranges were attained using rotating PSFs, based on a linear combination of Laguerre-Gaussian functions [418], later adapted to SMLM introducing the Double Helix PSF [406]. Toward improving axial range and SNR, over the years, various additional engineered PSFs for encoding 3D information have been designed and used, including the corkscrew [407], self-bending beams [419], tetrapods [408], and others [420, 421]. While attaining high resolutions over wide axial ranges, these 3D engineered PSFs also maintain high SNR even for large de-focus. This is in contrast to the wide-field PSF that spreads signal over a large area resulting in low SNRs away from the focus; see first row in Fig. 58.

Several examples of engineered phase masks, *i.e.*, phase intentionally added to the Fourier plane phase (Fourier plane phase is also sometimes termed pupil phase), and associated PSFs are shown in Fig. 58. We show both PSFs maintaining their shapes over a wide axial range and those encoding axial location in their shapes.

Now, we turn to the question of how we can design a phase mask to engineer a desired PSF shape, *e.g.*, a PSF maintaining high axial resolution or high SNR over a wide range. But first, we ask: what is the relation between the measured PSF and the phase mask at the Fourier plane?

To address this, we note the relation between the field at the Fourier plane, and the measured PSF intensity, as described in Eqs. 80 and 87. Indeed, the measured PSF intensity contains a Fourier transform of the electric field, and an absolute value operation, resulting in the loss of image plane, *i.e.*, detector plane, phase information. As such, the problem of recovering Fourier plane phase, which is at the heart of PSF engineering, is known as phase retrieval [422]. The phase retrieval problem in our context, is to estimate the pupil phase $\Psi \left(\theta^{\prime },\phi \right)$ (see Eq. 87) from the measurements $w_{1:N}$ encoding the real space PSF through, for example, detector models such as Eq. A9. This is an ill-posed non-convex optimization problem with various challenges, including degenerate solutions, unstable derivatives, and more [422].

As it is impossible to determine phase using data from one plane, *i.e.*, a single slice of the PSF, we use data from several planes (z-stack) which might be acquired by observing a PSF of a fluorescent bead and scanning the objective, or by using a multi-plane setup; see Sec. IV F.

For example, we may index planes with q=1:Q and model each plane according to Eqs. 154–151. However, here, we envision our PSF as being an explicit function of the phase Ψ and only consider a single fluorophore. This model leads to an expected photon count $\Lambda_{n}^{q}(\vartheta ,\Psi )$, for the *n*th pixel within the *q*th plane of the z-stack, encoding pupil phase information.

Using this model, a likelihood can be constructed given data $w_{n}^{q},\;n=1:N,\;q=1:Q$ similar to Eqs. 153–152. Working, for convenience, with the log-likelihood, we write the z-stack log-likelihood

(163) $$ℒ\left(w_{1:N}^{1:Q}|\vartheta ,\text{Ψ}\right)=\sum_{n=1}^{N}\sum_{q=1}^{Q}\ell \left(w_{n}^{q}|\vartheta ,\text{Ψ}\right),$$

where $\ell \left(w_{n}^{q};\vartheta ,\Psi \right)$ is the log-likelihood of the *n*th pixel within the *q*th plane. In the most general case, detector noise and shot noise would both need to be simultaneously considered as in Eq. A9. However, ignoring detector noise for now, we arrive at the following single pixel log-likelihood

(164) $$\ell \left(w_{n}^{q};\vartheta ,\Psi \right)=\Lambda_{n}^{q}\left(\vartheta ,\Psi \right)-w_{n}^{q}\log\left(\Lambda_{n}^{q}\left(\vartheta ,\Psi \right)\right),$$

which we use to construct the log-likelihood of the z-stack as described in Eq. 163.

To maximize the likelihood in Eqs. 163–164, we employ iterative optimization methods often relying on knowledge likelihood’s gradient with respect to the phase as follows [159, 423]

(165) $$\frac{\partial \ell }{\partial \Psi }=\frac{\partial \ell }{\partial \Lambda_{n}^{q}}\frac{\partial \Lambda_{n}^{q}}{\partial \Psi }.$$

The first term on the right can be analytically evaluated as

(166) $$\frac{\partial \ell }{\partial \Lambda_{n}^{q}}=1-\frac{\Lambda_{n}^{q}}{w_{n}^{q}}.$$

The next term of Eq. 165 involves the derivative of the PSF model $\Lambda_{n}^{q}$ with respect to the phase mask $\Psi_{l}$ where, in computation, we discretize the set of spatial frequencies in the Fourier plane l=1:L and write

(167) $$\frac{\partial \Lambda_{n}^{q}}{\partial \Psi_{l}}=2R\left(\frac{\partial E_{n}}{\partial \Psi_{l}}\cdot E_{n}^{*}\right)$$

where $E_{n}$ is the sampled electric field in the image plane from Eq. 80 and R indicates the real part of the expression within the parenthesis. We note that $\frac{\partial E_{n}}{\partial \Psi_{l}}$ and $\frac{\partial \Lambda_{n}^{q}}{\partial \Psi_{l}}$ are, respectively, complex and real matrices both of size N×L.

Finally, we must evaluate $\frac{\partial E_{n}}{\partial \Psi_{l}}$. The electric field in the image plane is obtained by a Fourier transform of the electric field in the Fourier plane (designated by $E_{\tilde{l}}^{\prime }$) which also contains the phase mask (namely Ψ)

(168) $$\frac{\partial E_{n}}{\partial {\text{Ψ}}_{l}}=\frac{\partial }{\partial {\text{Ψ}}_{l}}{ℱ}_{\tilde{l}}\left[E_{\tilde{l}}^{\prime }\right]=\;\;i\cdot \text{exp}\left(\frac{-i2\pi nl}{M}\right)\cdot E_{\tilde{l}}^{\prime }\cdot \delta_{l,\tilde{l}}$$

where ${ℱ}_{\tilde{l}}$ is a discrete Fourier transform operation over index $\tilde{l}$ and $\delta_{l,\tilde{l}}$ is Kronecker’s delta function. Finally, if L=N the summation over n of Eq. 163 and the exponential function in Eq. 168 can be evaluated as a compact Fourier transform which provides the desired derivative

(169) $$\frac{\partial ℒ}{\partial \Psi_{l}}=2R\left(E_{l}^{\prime }{ℱ}_{n}\left(i\cdot E_{n}^{*}\cdot \frac{\partial ℒ}{\partial \Lambda_{n}^{q}}\right)\right).$$

The approach described above can be used both to learn the phase mask producing a measured PSF or, equivalently, design a PSF and learn the required phase mask.

In the realm of high SNR, it is also common to approximate the likelihood Eq. 163 by a Gaussian distribution and use least squares minimization to determine the phase. The approximate log-likelihood can then be minimized using iterative optimization, *e.g.*, Gerchberg-Saxton or its variants [424, 425], possibly estimated over a constrained Zernike polynomials set [390, 426].

After describing the approach to derive the phase mask for a given PSF shape, we turn to the problem of seeking a PSF optimal by pre-defined metrics, *without* knowing its shape in advance.

The engineered PSFs in Fig. 58 represents the results of various optimization metrics and numerical approaches. For instance, different PSFs exhibit different CRLB behaviors [427]; CRLB optimization on the phase mask expanded in terms of Zernike polynomials yields the tetrapod PSF [408] while optimization on the phase mask expanded in terms over Laguerre-Gaussian functions yields the Double Helix PSF [406, 428]. Similarly, in the panel on DS3D (standing for DeepSTORM3D) [429], the PSF is optimized to localize emitters within a dense environment using a neural network. Finally, for the EDOF PSF, a cost function is optimized to obtain PSFs maintaining their in-focus shape over a wider axial range [430].

As an example of optimization, to attain a PSF achieving optimal localization precision over a wide axial range, we use the Fisher information and CRLB metrics. To derive the CRLB, we start from the Fisher information matrix elements $[𝒬(\vartheta ;\Psi ){]}_{i,j}$ of the log-likelihood given in Eq. 164 (see Sec. I B)

(170) $$\begin{array}{r} {\left[𝒬\left(\vartheta ;\text{Ψ}\right)\right]}_{i,j}=\sum_{n=1}^{\text{N}}\frac{\partial }{\partial \vartheta_{i}}{\text{Λ}}_{n}\left(\vartheta ,\text{Ψ}\right)\frac{\partial }{\partial \vartheta_{j}}{\text{Λ}}_{n}\left(n;\vartheta ,\text{Ψ}\right)\\ \frac{1}{{\text{Λ}}_{n}\left(n;\vartheta ,\text{Ψ}\right)+{ℬ}_{n}}. \end{array}$$

Here, $\vartheta_{j}$ is a parameter within the set of unknowns designated by ϑ. After evaluating the Fisher information entries, we can evaluate the CRLB given by Eq. 12.

In a practical implementation of an iterative optimization procedure, the PSFs are scaled to match realistic signal counts encountered in SMLM imaging, *i.e*, on the scale of a few thousand photons per emitter per frame.

Heuristic and CRLB optimized PSFs, optimized for a *single* emitter, can encounter scenarios that drastically limit their performance. One such case is high labeling density, where engineered PSFs, such as the tetrapod [408], suffer from large PSF overlaps due to their large lateral footprint in the image plane. In such cases, fitting algorithms like MLE designed for sparse cases, exhibit a significant drop in performance with performance slightly improved for the DS3D PSF [429] due to its compact size.

One solution toward axial localization in dense environments is to let a neural net learn the optimal phase mask design [429]. In this case, localization and the encoding mask are simultaneously optimized for successful detection and localization of emitters in 3D.

In a similar vein toward optimizing PSFs for dense localization, similar design strategies have been used in multi-color imaging [423, 431] where neural networks have been used to optimize phase masks to optimally discriminate between colors [432].

## VI. PERSPECTIVES

The world of microscopy, and biology have been intertwined from the onset. As early as humankind could peer at the world beyond its visual range, it peered into life [8] and we continue doing so such as probe nuclear pore complexes [433] key in intra-cellular communication, individual synaptic spines [280], cell adhesion [434] at the basis of tissue formation, actin filaments [435, 436] involved in cell motion and division, and may more.

Yet what we know of life is that events of interest occur at all spatiotemporal scales with no clear means of discriminating between object of interest and background. Discrimination from background motivated fluorescence [437], while probing smaller and faster spatiotemporal scales continues to motivate the development of microscopy techniques.

Along these lines, major improvements in fluorescent microscopy have followed in four main fronts: fluorescent probes; optical setups; detectors; and data analysis.

Regarding fluorescent probes (see Sec. II), the discovery of Green Fluorescent Proteins (GFP) was a milestone in fluorescence microscopy [438, 439]. Next came the ability to switch biomarkers from dark and bright states [56, 440] leading to the development of super-resolution microscopy and nanometer spatial resolutions [64, 359]; see Sec. V.

Concerning optical setups (see Sec. III and IV), the invention of the confocal microscope [46] marked a milestone accomplishing optical sectioning by inserting a pinhole in the detection arm to filter out-of-focus light. Research in this area is ongoing leading to development of different microscopy modalities, *e.g.*, light-sheet, SIM and others, discussed in Sec. IV, yielding unprecedented optical sectioning as well as high lateral resolutions.

On the detector front (see Appendix A), the coming of fast cameras, including CCDs, EMCCDs, and CMOSs, have revolutionized the field of fluoroscence microscopy and enabled rapid wide-field imaging. Indeed, the need to amplify signal lead to the development of EMCCDs capable of imaging dim fluorescent probes [441]. Moreover, recently, the advent of CMOS cameras has accelerated data acquisition up to hundreds of frames per second, over large FOVs with reduced read-out noise [442]. While we mostly focused on integrative detectors, increasingly available Single Photon Avalanche Diodes (SPAD) arrays [443, 444], have the potential to herald an era of unparalleled spatiotemporal resolution.

Finally data analysis methods grounded in statistics are naturally suited to process fluorescent microscopy data while considering all sources of uncertainty; see Sec. I B. Moreover, considering the fundamental problem of model selection, BNP frameworks (see Sec. I B) have equipped us with the ability to assign probabilities over candidate models especially relevant given the minimal photon numbers provided as we keep pushing to faster and smaller spatiotemporal scales. Deep learning methods [445–447] have also recently gained popularity for fluorescent data analyses due to their speed though these tools are necessarily supervised and require model training.

Despite the continued progress and promise of fluorescence microscopy [184, 448, 449], the community still faces multiple challenges including potentially perturbative effects of fluorescent probes on the systems they label; uncontrolled probe interaction with themselves and their environment; photo-toxic effects naturally arising from any form of illumination; as well as labeling and detection challenges in complex environments; and many others.

Indeed, as we move to complex environments complementary read-outs beyond fluorescence are often desired and, along these fronts, a number of other methods continue to be developed. These include refractive index tomography [450, 451], Raman imaging [452, 453], phase imaging [454, 455], lens-free imaging [456], ghost imaging [457, 458], expansion microscopy [459, 460] and others proven useful at the nanoscale.

Together, these approaches, alongside the development of theoretical and numerical tools, may help us visualize life’s events otherwise unfolding in environments that remain impenetrable and at scales still beyond our reach.

## Figures and Tables

**FIG. 59:**
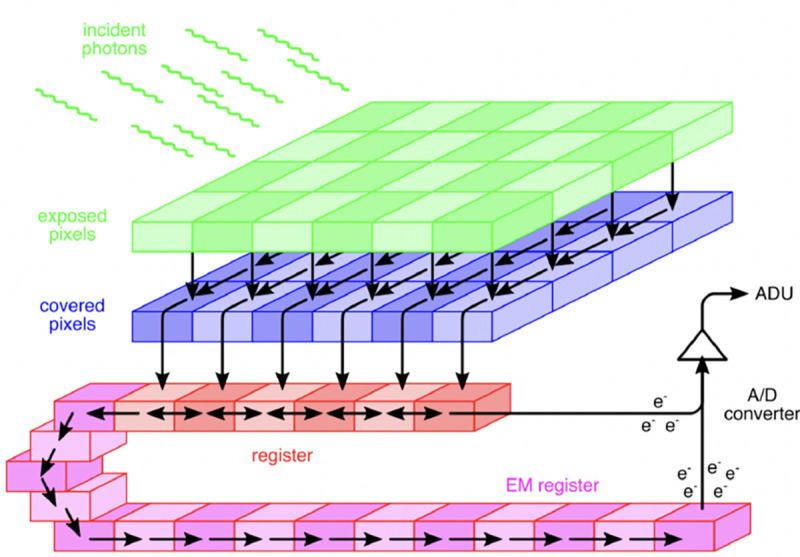
A cartoon illustration of CCD/EMCCD detector design. For details see the main text.

**FIG. 60:**
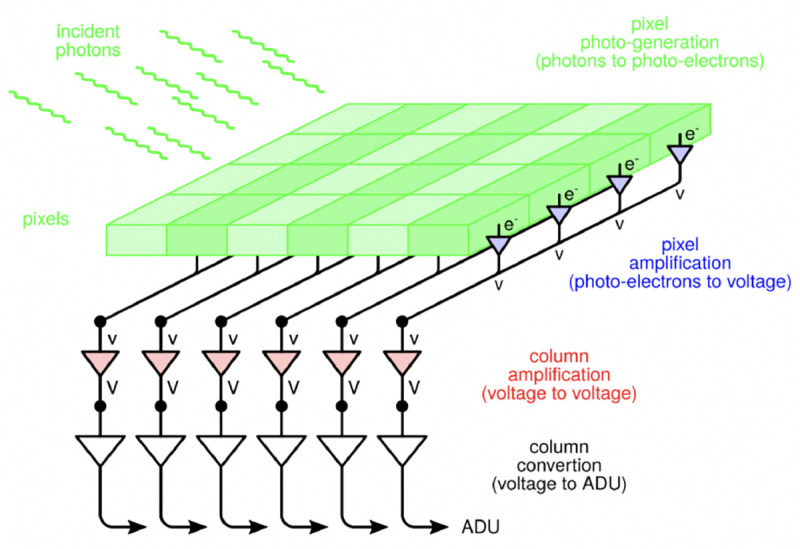
A cartoon illustration of CMOS detector design.

**FIG. 61:**
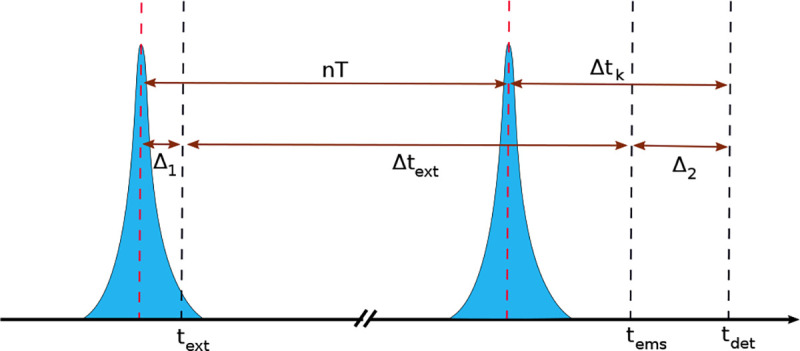
Single photon detector. The laser pulses and their centers are, respectively, shown by blues spikes and red dashed lines with inter-pulse window T. The fluorophore excitation, photon emission and photon detection events take place, respectively, at $t_{ext},\;t_{ems}$ and $t_{det}$ designated by black dashed lines. The fluorophore spends time $\Delta t_{ext}$ in the excited state and emits a photon after n pulses. The reported photon arrival time, $\Delta t_{k}$, is measured with respect to the immediate previous pulse center. Moreover, $\Delta_{1}$ and $\Delta_{2}$ denote the difference of the excitation pulse center and the detector delay in reporting the photon arrival time.

## Statistical Framework II C: FRET

Data: Photon inter-arrival times

$$\Delta t_{1:K}=\left\{\Delta t_{1},\ldots ,\Delta t_{K}\right\}.$$

Parameters: loads, transition rates, initial probability vector

$$\vartheta =\left\{\overset{‾}{b},K,P_{\text{in}\;}\right\}.$$

Likelihood:

$$P(\bar{\Delta t}|K,P_{\text{in}\;})\propto P_{\text{in}\;}Q_{1}^{\text{non}\;}\ldots Q_{k}^{\text{rad}\;}\ldots Q_{N}^{\text{non}\;}P_{\text{norm}}^{T}.$$

Priors:

$$q_{m}∼\mathrm{Beta}\left(A_{q},B_{q}\right),m=1:\infty ,$$

$$b_{m}∼\mathrm{Bernoulli}\left(q_{m}\right),$$

$$K∼\mathrm{Gamma}\left(\alpha_{K},\beta_{K}\right),$$

$$P_{\text{in}\;}∼\mathrm{Dirichlet}\left(\alpha_{\Pi }\right).$$

Posterior:

$$P\left(\vartheta |\bar{\Delta t}\right)\propto P\left(\bar{\Delta t}|\vartheta \right)P\left(\vartheta \right).$$

## Statistical Framework II C: Counting

Data: Sum of ROIs’ pixel values in ADUs

$${\overset{‾}{w}}_{1:K}=\left\{w_{1:K}^{1},\ldots ,w_{1:K}^{R}\right\}.$$

Parameters: loads, fluorophore intensity, background, transition probabilities, fluorophores’ trajectories in the state space

$$\vartheta =\left\{\bar{\overset{‾}{b}},I_{A},\bar{ℬ},\Pi ,\bar{\bar{𝒮}}\right\}.$$

Likelihood:

$$P\left({\overset{‾}{w}}_{1:K}|\vartheta \right)=\prod_{k,r}\;P\left(w_{k}^{r}|\Lambda_{k}^{r},\Xi \right).$$

Priors:

$$q_{m}∼Beta\left(A_{q},B_{q}\right),m=1:\infty ,$$

$$b_{m}∼Bernoulli\left(q_{m}\right),$$

$$I_{A}∼Gamma\left(\alpha_{A},\beta_{A}\right),$$

$$ℬ∼Gamma\left(\alpha_{ℬ},\beta_{ℬ}\right),$$

$$\Pi ∼Dirichlet\left(\alpha_{\Pi }\right),$$

$$s_{k}^{mr}|s_{k-1}^{mr},\Pi ∼Categorical(\pi_{s_{k-1}^{mr}}).$$

Posterior:

$$P\left(\vartheta |{\overset{‾}{w}}_{1:K}\right)\propto P\left({\overset{‾}{w}}_{1:K}|\vartheta \right)P(\vartheta ).$$

## Statistical Framework IV C: Confocal under continuous illumination

Data: photon inter-arrival times

$$\Delta t_{1:K}=\left\{\Delta t_{1},\ldots ,\Delta t_{K}\right\}.$$

Parameters: loads, diffusion coefficient, molecule trajectories, molecule maximum emission rate, background emission rate

$$\vartheta =\left\{\overset{‾}{b},D,\bar{\bar{r}},\mu_{0},\mu_{ℬ}\right\}.$$

Likelihood:

$$P\left(\Delta t_{1:K}|\vartheta \right)=\prod_{k}\;\text{Exponential}\;\left(\Delta t_{k};\mu_{k}(r)\right).$$

Priors:

$$q_{m}∼Beta\left(A_{q},B_{q}\right),m=1:\infty ,$$

$$b_{m}∼Bernoulli\left(q_{m}\right),$$

$$D∼InvGamma\left(\alpha_{D},\beta_{D}\right),$$

$$r_{k}^{m}∼Normal\left(r_{k-1}^{m},2D\Delta t_{k}\right),$$

$$\mu_{0}∼Gamma\left(\alpha_{\mu },\beta_{\mu }\right),$$

$$\mu_{ℬ}∼Gamma\left(\alpha_{b},\beta_{ℬ}\right).$$

Posterior:

$$P\left(\vartheta |\Delta t_{1:K}\right)\propto P\left(\Delta t_{1:K}|\vartheta \right)P(\vartheta ).$$

## Statistical Framework IV C: single pixel FLIM

Data: photon arrival times

$$\Delta t_{1:K}=\left\{\Delta t_{1},\ldots ,\Delta t_{K}\right\}.$$

Parameters: inverse lifetimes, weights

$$\vartheta =\left\{\lambda_{1:M},\pi_{1:M}\right\}.$$

Likelihood:

$$P\left(\Delta t_{1:K}|\vartheta \right)=\prod_{k}\;P\left(\Delta t_{k}|\vartheta \right).$$

Priors:

$$\lambda_{m}∼\mathrm{Gamma}\left(\alpha_{\lambda },\beta_{\lambda }\right),$$

$$\pi_{m}∼{Dirichlet}_{1:M}\left(\frac{\alpha_{\pi }}{M},\ldots ,\frac{\alpha_{\pi }}{M}\right),M\to \infty$$

Posterior:

$$P\left(\vartheta |\Delta t_{1:K}\right)\propto P\left(\Delta t_{1:K}|\vartheta \right)P(\vartheta ).$$

## Statistical Framework IV C: Wide-field FLIM

Data: photon arrival times and pulses being empty or not

$$\bar{\bar{\Delta t}}=\left\{{\bar{\Delta t}}^{i},\ldots ,{\bar{\Delta t}}^{N}\right\},$$

$$\bar{\bar{𝒲}}=\left\{{\bar{𝒲}}^{i},\ldots ,{\bar{𝒲}}^{N}\right\}.$$

Parameters: loads, inverse lifetimes, lifetime maps, average of GP priors

$$\vartheta =\left\{b_{1:M},\lambda_{1:M},\Omega_{1:M},\nu_{1:M}\right\}.$$

Likelihood:

$$P\left(\bar{\bar{𝒲}},\bar{\bar{\Delta t}}|\vartheta \right)=\prod_{k}\;\prod_{n}\;P\left({𝒲}_{k}^{n};\vartheta \right)P\left(\Delta t_{k}^{n};\vartheta \right).$$

Priors:

$$q_{m}∼Beta\left(A_{q},B_{q}\right),m=1:\infty ,$$

$$b_{m}∼Bernoulli\left(q_{m}\right),$$

$$\lambda_{m}∼Gamma\left(\alpha_{\lambda },\beta_{\lambda }\right),$$

$$\Omega_{m}∼\mathrm{GP}\left(\nu_{m},K\right),$$

$$\nu_{m}∼Normal\left(0,\sigma_{\chi }^{2}\right).$$

Posterior:

$$P\left(\vartheta |\bar{\bar{𝒲}},\bar{\bar{\Delta t}}\right)\propto P\left(\bar{\bar{𝒲}},\bar{\bar{\Delta t}}|\vartheta \right)P\left(\vartheta \right).$$

## Statistical Framework IV D: SIM

Data: pixel values in ADUs

$${\bar{\bar{w}}}_{1:N}=\left\{{\bar{\bar{w}}}_{1},\ldots ,{\bar{\bar{w}}}_{N}\right\}.$$

Parameters: fluorophore distribution, a hyperparameter

$$\vartheta =\left\{S\left(r\right),\nu \right\}.$$

Likelihood:

$$p\left({\bar{\bar{w}}}_{1:N}|\vartheta \right)=\prod_{i=1}^{3}\prod_{l=1}^{3}\prod_{n=1}^{N}\mathrm{Gaussian}\left(w_{n}^{li};g\Lambda_{n}^{li}+o,\sigma_{w}^{2}\right).$$

Priors:

(144)
$$S(r)\;∼GP(0,\Sigma (\nu )),\;\nu \;∼Gamma\left(\alpha_{\nu },\beta_{nu}\right).$$

Posterior:

$$P\left(\vartheta |{\bar{\bar{w}}}_{1:N}\right)\propto P\left({\bar{\bar{w}}}_{1:N}|\vartheta \right)P(\vartheta ).$$

## Statistical Framework V B 3: SMLM

Data: pixel values in ADUs

$$w_{1:N}=\left\{w_{1},\ldots ,w_{N}\right\}.$$

Parameters: loads, fluorophore locations, intensities, background

$$\vartheta =\left\{\overset{‾}{b},\bar{r},\overset{‾}{I},ℬ\right\}.$$

Likelihood:

$$P\left(w_{1:N}|\vartheta \right)=\prod_{n}\;Gaussian\left(w_{n};g\Lambda_{n}(\vartheta )+o,\sigma_{w}^{2}\right).$$

Priors:

$$q_{m}∼Beta\left(A_{q},B_{q}\right),m=1:\infty ,$$

$$b_{m}∼\mathrm{Bernoulli}\left(q_{m}\right),$$

$$r_{m}∼Uniform,$$

$$I_{m}∼Empirical,$$

$$ℬ∼Gamma\left(\alpha_{ℬ},\beta_{ℬ}\right).$$

Posterior:

$$P\left(\vartheta |w_{1:N}\right)\propto P\left(w_{1:N}|\vartheta \right)P\left(\vartheta \right).$$

## Statistical Framework V B 3: Tracking

Data: pixel values in ADUs

$$w_{1:N}^{1:K}=\left\{w_{1}^{1},\ldots ,w_{N}^{K}\right\}.$$

Parameters: loads, fluorophore trajectories, emission rate, background, diffusion coefficient

$$\vartheta =\left\{\overset{‾}{b},\bar{r}\left(t_{1:L}^{1:K}\right),\mu ,ℬ,D\right\}$$

Likelihood:

$$P\left(w_{1:N}^{1:K}|\vartheta \right)=\prod_{n=1}^{N}\prod_{k=1}^{K}\mathrm{Gaussian}\left(w_{n}^{k};g\Lambda_{n}^{k}(\vartheta )+o,\sigma_{w}^{2}\right).$$

Priors:

$$q_{m}∼\mathrm{Beta}\left(A_{q},B_{q}\right),m=1:\infty$$

$$b_{m}∼\mathrm{Bernoulli}\left(q_{m}\right),$$

$$r_{m}\left(t_{1}^{1}\right)∼\mathrm{Normal}\left(r_{0},\sigma_{r}^{2}\right),$$

$$r_{m}\left(t_{l+1}^{k}\right)|r_{m}\left(t_{l}^{k}\right)∼\mathrm{Normal}\left(r_{m}\left(t_{l}^{k}\right),2D\delta t\right),$$

$$\mu ∼Gamma\left(\alpha_{\mu },\beta_{\mu }\right)$$

$$ℬ∼Gamma\left(\alpha_{ℬ},\beta_{ℬ}\right)$$

$$D∼InvGamma\left(\alpha_{D},\beta_{D}\right)$$

Posterior:

$$P\left(\vartheta |w_{1:N}^{1:K}\right)\propto P\left(w_{1:N}^{1:K}|\vartheta \right)P(\vartheta ).$$

## Appendix A: Detectors physics

Every photon carries with it information that can be recorded by detectors and later employed to draw inferences. These detectors may comprise of either a single or many pixels arranged into 2D arrays. The former is mostly employed in point scanning microscopy techniques to record single photon arrival times in experiments such as FLIM and FRET. The latter is suitable in wide-field fluorescence microscopy. Instead of recording single photons, in wide-field detectors, ideally, pixel values would simply histogram the photon counts incident on a particular pixel over the course of a given exposure time. However, due to the stochastic noise inherent to the device, the reported arrival times and pixel values are not the direct photon emission times and photon counts [215, 461, 462]. Rather, each of the reported values is a random variable sampled from a probability distribution (see Sec. I B) dependent on the photon emission time and the number of incident photons for single pixel and wide-field detectors, respectively. This section lays out noise models for reported values by different detectors motivated by the physics of the detector types used in experiments. Once the model is formulated, its parameters can be estimated for specific detectors using data from calibration experiments [218, 219, 463–466]. In what follows, we first describe wide-field detectors (integrative detectors) and next turn to single photon detectors.

There are three common types of wide-field detectors used in single molecule fluorescence microscopy: CCDs [467–469]; EMCCDs [441, 470, 471]; and CMOS [469, 472]. In what follows, we describe the architecture and physics of each detector and, in turn, derive the appropriate noise model.

We begin with a cartoon of detector devices. Fig. 59 depicts the main components of a CCD/EMCCD detector. The green pixel grid represents photo-active capacitors that accumulate photo-electrons proportional to the incident photon counts. The blue grid is a set of capacitors that temporarily hold the photo-electrons. The blue grid then transfers its electrons to the red register, one row at a time. In CCD cameras there is no Electron Multiplier (EM) stage and the transferred electrons follow the arrows to the right in Fig. 59 and go to the charge-to-voltage converter. The voltages are then converted into ADUs and recorded as pixel values.

By contrast, in EMCCD detectors, the transferred electrons follow the arrows to the left in the red register and undergo an amplification stage shown in pink before going to the charge-to-voltage converter; see Fig. 59. In the EM stage, electrons are fed through a chain of avalanche EMs where an electric field is applied to the electrons, giving them sufficient kinetic to knock other electrons into the material’s conducting band. This creates new electron-hole pairs and amplifies the current. Each stage in the EM process has a small expected gain (~ 1%) but the device has many stages thereby dramatically amplifying the current prior to reaching the charge-to-voltage converter.

While the amplification process and the charge-to-voltage conversion in the CMOS is similar to EMCCD, every pixel of the CMOS has its own capacitor and amplifier independent of other pixels; see Fig. 60. This allows for faster data acquisition, a larger FOV and lower noise. However, such architecture imposes pixel-dependent noise thereby complicating the noise model.

### Noise models

Every stage involved in detecting and converting incident photons to ADUs in detectors introduces a type of noise to the final reported pixel values. In this section, we will discuss noise models introduced at every stage:

1) The first source of noise stems from the discrete nature of photons. Given the expected photon count for pixel $n,\;\Lambda_{n}$, over a fixed exposure time (*e.g.*, see Eq. 150), the measured photon count, $N_{Ph,n}$, is Poisson distributed, *i.e.*, shot noise limited [464, 473–475]

(A1)
$$N_{Ph,n}∼Poisson\left(\Lambda_{n}\right)$$

where we use notation introduced in Sec. IB. 2) Only a fraction of the photons incident on the detector generate photo-electrons where the expected number of photo-electrons per incident photon is called the quantum efficiency β [474, 476, 477]. The number of generated photo-electrons, $N_{pe,n}$, therefore follows a Binomial distribution [476]

(A2)
$$N_{pe,n}∼\mathrm{Binomial}\left(N_{Ph,n},\beta \right).$$

The distribution over the number of photo-electrons given the expected number of photons, $\Lambda_{n}$, can then be obtained by marginalizing over the incident number of photons (see Sec. IB), as follows

(A3)
$$\text{Poisson}\left(N_{\text{pe},n};\beta {\text{Λ}}_{n}\right)=\sum_{N_{\text{Ph},n}=N_{\text{pe},n}}^{\infty }\text{Poisson}\left(N_{\text{Ph},n};{\text{Λ}}_{i}\right)\;\;\times \text{Binomial}\left(N_{\text{pe},n};N_{\text{Ph},n},\beta \right).$$

3) The third source of noise is due to spurious charge consisting of unwanted electrons generated during the transfer process, termed Clock Induced Charge (CIC) [476, 478]. The CIC noise follows a Poisson distribution and gives rise to additional electrons while transferring to the register

(A4)
$$N_{\text{te},n}∼Poisson\left(\beta \Lambda_{n}+C\right)$$

where $N_{\text{te},n}$ and C are the number of electrons after the transferring stage and the mean value of the CIC. This noise is small but can be greatly amplified during the electron multiplier step of EMCCD detectors.

4) The EM process consists of many stages in which new electrons are excited through impact ionisation, which can be considered a cascade of stochastic events. These steps are approximately identical thus the EM process can be modeled as a cascade of Poisson processes, or Bernoulli trials [476, 479], or a geometric model of multiplication [480, 481].

The number of electrons after the EM stage, $N_{ae,n}$, is a random variable that approximately follows a Gamma distribution [471, 475, 476]

(A5)
$$N_{ae,n}∼Gamma\left(N_{te,n},\overset{ˆ}{g}\right)$$

where $\overset{ˆ}{g}$ denotes the amplification gain given by the ratio of the output and input electrons to the EM stage. For large values of $N_{\text{te},n}$, this process is further approximated by a Gaussian, which is computationally more efficient [471, 476]

(A6)
$$N_{ae,n}∼Gaussian\left(\overset{ˆ}{g}N_{te,n},{\overset{ˆ}{g}}^{2}N_{te,n}\right).$$

5) The last stage is called the read-out process where the input electrons to the converter, $N_{ae,n}$, are converted into discrete pixel values, reported as the data $w_{n}$ in ADUs. This stage introduces another gain γ (ADUs per electron, which is also sometimes refereed to as sensitivity and is typically smaller than one) and offset μ (output ADU at zero input electron often added to avoid negative pixel values) modeled by a Gaussian distribution and termed “read-out noise”

(A7)
$$w_{n}∼Gaussian\left(\gamma N_{ae,n}+\mu ,\sigma_{ro}^{2}\right)$$

where $\sigma_{\text{ro}}^{2}$ is the read-out noise variance.

The combination of the noises introduced via the amplification and read-out stages is obtained by marginalizing the intermediate parameter $N_{ae,n}$ (namely the number of electrons after the EM stge) between Eqs. A6 and A7, resulting in

(A8)
$$w_{n}∼Gaussian\left(\tilde{g}N_{te,n}+\mu ,\sigma_{w}^{2}\right)$$

where $\tilde{g}=\gamma \overset{ˆ}{g}$ and $\sigma_{w}^{2}=\gamma^{2}{\overset{ˆ}{g}}^{2}N_{\text{te}\;,n}+\sigma_{\text{ro}\;}^{2}$ denote total gain and variance, respectively. Finally, the entire detector model, which relates the expected photon count $\left(\Lambda_{n}\right)$ to the reported pixel value $\left(w_{n}\right)$ is obtained by marginalizing the other intermediate parameter $N_{\text{te}\;,n}$ (namely the number of electrons after the transferring stage) between Eqs. A4 and A8

(A9)
$$P\left(w_{n}|{\text{Λ}}_{n}\right)\;=\sum_{N_{\text{te},n}=0}^{\infty }\text{Poisson}\left(N_{\text{te},n};\beta {\text{Λ}}_{n}+C\right)\;\;\times \text{Gaussian}\left(w_{n};\tilde{g}N_{\text{te},n}+\mu ,\sigma_{w}^{2}\right).$$

Since we did not make any assumptions about the gain, offset and other parameters to derive the noise model above, it is valid for both CCD and EMCCD detectors. Moreover, if we assume pixel-dependent parameters, *e.g.*, gain, offset and others, this model would be valid for CMOS detectors as well. However, the noise model in Eq. A9 has a complicated form adding computational complexity. Therefore, in what follows, we make appropriate approximations to derive simpler noise models specialized for each detector.

We start by CCD detectors. The CCD detectors do not have an EM amplification stage $\left(\overset{ˆ}{g}\approx 1\right.$ and $\left.\sigma_{w}^{2}\approx \sigma_{ro}^{2}\right)$ and therefore are suitable for detecting large input signals compared to the read-out noise variance. This can be quantitatively expressed as

(A10)
$$SNR=\frac{\Lambda_{n}}{\sigma_{ro}}\gg 1,$$

which implies that the signal will not be buried under read-out noise. Therefore the noise associated to a pixel value $w_{n}$ is given by Eq. A9 when $\tilde{g}=\gamma$ and ${\sigma_{w}^{2}=\sigma }_{\text{ro}}^{2}$. Under the large signal $\left(\Lambda_{n}\right)$ assumption, the Poisson distribution Eq. A4 can be approximated by a Gaussian distribution where both mean and variance are given by the mean of the Poisson distribution

(A11)
$$P\left(w_{n}|{\text{Λ}}_{n}\right)\;\approx \sum_{N_{\text{te},n}=0}^{\infty }\text{Gaussian}\left(N_{\text{te},n};\beta {\text{Λ}}_{n},\beta {\text{Λ}}_{n}\right)\;\;\;\times \text{Gaussian}\left(w_{n};\gamma N_{\text{te},n}+\mu ,\sigma_{w}^{2}\right)$$

where we neglected the spurious charge *C* due to the absence of the amplification stage in CCD cameras. Therefore, Eq. A11 results in

(A12)
$$w_{n}|\Lambda_{n}∼Gaussian\left(g\Lambda_{n}+o,\sigma_{w}^{2}\right)$$

where g=γβ,o=μ and $\sigma_{w}^{2}=\sigma_{\text{ro}}^{2}$, respectively, denote gain, offset, and variance for CCD detectors. It is also common to apply the offset and gain to the pixel values (data) and write the above equation for gain and offset corrected pixel values

(A13)
$$\left(w_{n}-o\right)/g|\Lambda_{n}∼Gaussian\left(\Lambda_{n},\sigma_{w}^{2}/g^{2}\right).$$

Next, we consider EMCCD detectors. These detectors are suitable to record signals when SNR (given in Eq. A10) is low. These detectors amplify the signal in the EM stage above the read-out noise $\left(\overset{ˆ}{g}\gg \sigma_{w}^{2}\right)$ and allow for the detection of weak signals. To simplify Eq. A9 for EMCCD cameras, we write the explicit form of this equation

(A14)
$$P\left(w_{n}|{\text{Λ}}_{n}\right)=\sum_{N_{\text{te},n}=0}^{\infty }\frac{{\left(\beta {\text{Λ}}_{n}\right)}^{N_{\text{te},n}}e^{-\beta {\text{Λ}}_{n}}}{N_{\text{te},n}!}\;\;\;\;\;\;\;\;\;\;\;\;\;\;\;\;\;\;\;\;\;\;\;\times \frac{1}{\sqrt{2\pi \sigma_{D}^{2}}}\text{exp}\left[-\frac{{\left(N_{\text{te},n}-\frac{w_{n}-\mu }{\tilde{g}}\right)}^{2}}{2\sigma_{w}^{2}/{\tilde{g}}^{2}}\right]$$

where we have factorized $\tilde{g}$ in the exponent. For large amplifications the standard deviation $\sigma_{w}^{2}/{\tilde{g}}^{2}$ becomes very small and results in a very narrow Gaussian which can be approximated by a delta function. Therefore, with marginalization and some additional algebra we recover [464]

(A15)
$$\left(w_{n}-o\right)/g|\Lambda_{n}∼\frac{1}{\Gamma \left(1+\frac{w_{n}-o}{g}\right)}e^{-\beta \Lambda_{n}}{\left(\beta \Lambda_{n}\right)}^{\frac{w_{n}-o}{g}}$$

where o=μ and $g=\tilde{g}$ denote offset and gain, respectively. The above EMCCD model for the corrected pixel values is similar to a Poisson noise model where the corrected pixel values do not need to be integer [464]. An alternative EMCCD camera noise model could be obtained by convolution of the Poisson distribution Eq. A4 and the Gamma noise model for EM amplification Eq. A5 resulting in an approximate Gamma noise model for EMCCD detectors [62, 471, 476]. Both of these noise models asymptotically converge to the same model for large gain g.

After deriving noise models for CCD and EMCCD detectors with pixel-independent gain, g, offset, o and variance, $\sigma_{w}^{2}$, we continue to derive the noise model for CMOS detectors. However, in the case of CMOS detectors, the gain, variance and offset are pixel-dependent and have to be characterized for all pixels [464, 482, 483]. Therefore, every pixel follows a different noise model similar to Eq. A9

(A16)
$$\begin{array}{l} P\left(w_{n}|\Lambda_{n}\right)=\sum_{N_{\text{te},n}=0}^{\infty }\mathrm{Poisson}\left(N_{\text{te},n};\beta \Lambda_{n}\right)\\ \;\;\;\;\;\;\;\;\;\;\;\;\;\;\times \mathrm{Gaussian}\left(w_{n};{\tilde{g}}_{n}N_{\text{te}\;,n}+\mu_{n},\sigma_{w,n}^{2}\right), \end{array}$$

where n counts pixels. Provided that the gain ${\tilde{g}}_{n}$ is small for CMOS detectors, the above convolution can be approximated by a Poisson distribution assuming an extra source of photon for each n pixel contributing $\sigma_{w,n}^{2}/g_{n}^{2}$ photons [464]

(A17)
$${\overset{ˆ}{w}}_{n}|\Lambda_{n}∼Poisson\left(\Lambda_{n}+\sigma_{w,n}^{2}/g_{n}^{2}\right)$$

where ${\overset{ˆ}{w}}_{n}=\left(w_{n}-o_{n}\right)/g_{n}+\sigma_{w,n}^{2}/g_{n}^{2},g_{n}$ is the gain, characterized from calibration experiments, and $o_{n}=\mu_{n}$ is the pixel-dependent offset.

After considering wide-field detectors, we proceed to describe noise models for single photon detector. We do so by assuming fluorophore excitation using a pulsed laser. This is illustrated in Fig. 61 where the laser pulses are designated by blue spikes with inter-pulse window T. Here, the fluorophore gets excited during a pulse at time $t_{\text{ext}\;}$, spends $\Delta t_{\text{ext}\;}$ time in the excited state and emits a photon, in the most general case, after n pulses at $t_{ems}$. However, the photon arrival time is recorded as $t_{\text{det}\;}$ by a $\Delta_{2}$ delay in the detector and is reported with respect to the immediate previous pulse given by $\Delta t_{k}$ for the *k*th photon.

From Fig. 61, we can write the following relation for the reported photon arrival time

(A18)
$$\Delta t_{k}=\Delta t_{ext}+\Delta t_{IRF}-nT$$

where $\Delta t_{IRF}=\Delta_{1}+\Delta_{2}$ is the noise introduced by the IRF due to the finite size of the laser pulses and the stochastic delay in the detector. Here, the reported arrival time is the sum of three random variables. As such, the noise model is given by the convolution of probability distributions for these three variables

(A19)
$$P\left(\Delta t_{k}|\lambda \right)\;=P(n|N)\;\;⊗\left[P\left(\Delta t_{ext}|\lambda \right)⊗P\left(\Delta t_{IRF}|\tau_{IRF},\sigma_{IRF}^{2}\right)\right]$$

where $\lambda ,\;\tau_{IRF},\;\sigma_{IRF}^{2}$ and N are, respectively, the rate of excited state decay (inverse of the excited state lifetime; see Eq. 20), the offset of the IRF, the variance of the IRF, and the maximum possible number of pulses after which the fluorophore emits. These distributions are given by

(A20)
$$\Delta t_{ext}|\lambda ∼Exponential\left(\Delta t_{ext};\lambda \right)$$

(A21)
$$\Delta t_{IRF}|\tau_{IRF},\sigma_{IRF}^{2}∼Normal\left(\Delta t_{IRF};\tau_{IRF},\sigma_{IRF}^{2}\right)$$

(A22)
$$n|N∼Categorical\left(A_{0},\ldots ,A_{N}\right)$$

where the time spent in the excited state and the IRF time are sampled from Exponential and Normal distributions. Moreover, the pulse at which the fluorophore emits is sampled from a categorical distribution where $A_{n}$ is given by the integral of the term inside brackets in Eq. A19 over the *n*th pulse [215]. Finally, calculating the convolutions in Eq. A19, we obtain the following noise model for single photon detectors under pulsed illumination [215]

(A23)
$$P\left(\text{Δ}t_{k}|\lambda \right)=[\sum_{n=0}^{N}\frac{\lambda }{2}\text{erfc}\left(\frac{\tau_{\text{IRF}}-\text{Δ}t_{k}-nT+\lambda \sigma_{\text{IRF}}^{2}}{\sigma_{\text{IRF}}\sqrt{2}}\right)\;\;\times \text{exp}\left(\frac{\lambda }{2}\left(2\left(\tau_{\text{IRF}}-\text{Δ}t_{k}-nT\right)+\lambda \sigma_{\text{IRF}}^{2}\right)\right)],$$

where erfc(.) denotes the complementary error function [156]. In many practical cases, the inter-pulse time is much larger than the fluorophore lifetime (inverse of fluorophore radiative decay, T≫1/λ) where the fluorophore emits before the next pulse. In such case, the noise model can be simplified by setting N=0 [461].
